# Peptidic Catalysts Conformationally Tuned for Fluoride Binding and Delivery

**DOI:** 10.1021/jacs.6c01667

**Published:** 2026-02-24

**Authors:** Gabija Poškaitė, Thomas Schlatzer, Zijun Chen, Mihai V. Popescu, Robert S. Paton, Véronique Gouverneur

**Affiliations:** † Chemistry Research Laboratory, 6396 University of Oxford, 12 Mansfield Road, Oxford, OX1 3TA, United Kingdom; ‡ Department of Chemistry, 3447 Colorado State University, Fort Collins, Colorado 80528, United States

## Abstract

Knowledge on how fluoride interacts with peptides is currently limited to *in silico* studies. Here, we report an experimental investigation on the ability of peptidic scaffolds to bind fluoride using TBAF·3H_2_O or CsF. For CsF, in-depth NMR and GOAT-DFT studies shed light on peptides acting as chelators to both fluoride and cesium ions. This finding led to the development of the first peptide-catalyzed fluorination reactions. These advances open a new avenue to investigate fluorination chemistry with peptide-based catalysts that are considered as the possible ancestors to enzymes.

## Graphical abstract

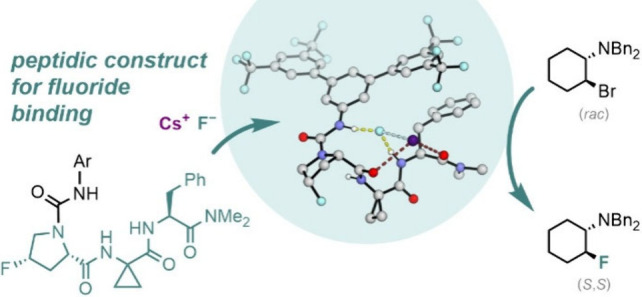

The remarkable ability of enzymes to catalyze a variety of reactions under mild conditions has been a constant source of inspiration for the discovery of new organocatalysts,^1^ including catalytic fluorination processes.^2^ In nature, fluorine chemistry is limited to the nucleophilic substitution of *S*-adenosyl methionine with fluoride (F^–^) to generate 5′-fluoro-5′-deoxyadenosine and l-methionine, so inspiration stemmed from this unique enzyme. In-depth studies of the 5′-fluoro-5′-deoxyadenosine synthase enzyme, also known as fluorinase, have revealed that precise tuning of the local environment around fluoride through hydrogen bonding (H-bonding) in the active site is key for desolvation, binding, and catalysis (Figure 1, *middle*).^3^

**Figure 1:**
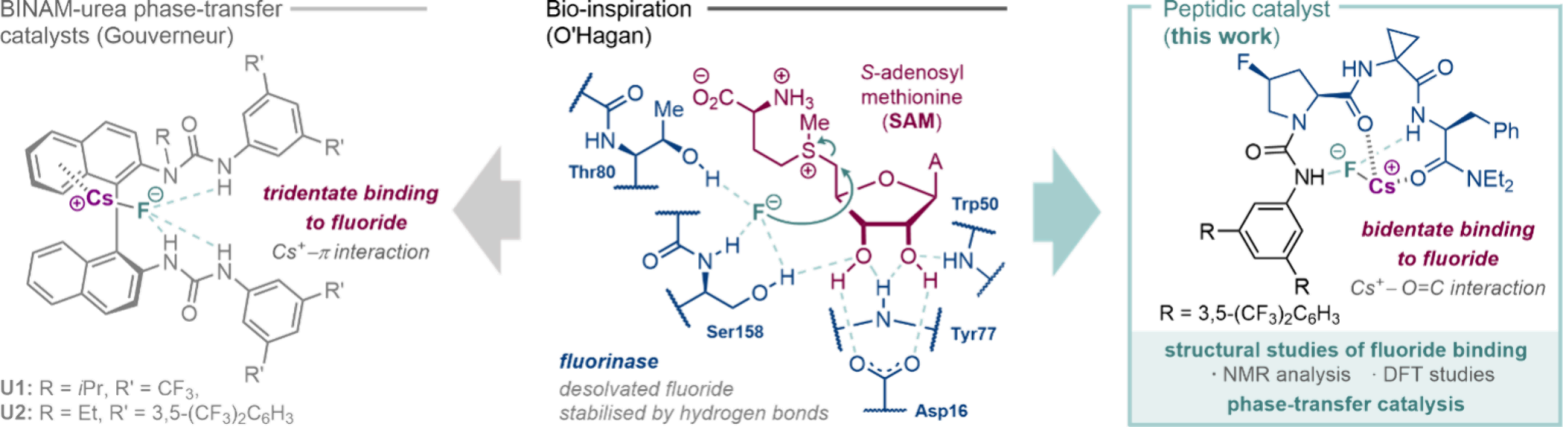
Inspiration from enzymatic fluorination (*middle*). BINAM-urea catalysts for asymmetric nucleophilic fluorination via HB-PTC (*left*). Peptide-based platform for fluoride binding and catalytic fluorination (this work, *right*).

In previous work inspired by the fluorinase enzyme, we reported the design and synthesis of *N*-alkylated BINAM-derived *bis*-urea catalysts for enantioselective fluoride delivery onto various classes of alkyl halides.^4^ In this chemistry, the *bis*-urea acts as a phase-transfer catalyst and brings solid alkali metal fluoride (CsF or KF) into solution through H-bonding interactions with fluoride (Figure 1, *left*). In search for new catalysts to expand the scope of nucleophilic fluorination via H-bonding phase-transfer catalysis (HB-PTC), we prioritized peptides as these structures allow for considerable structural variability while conserving the advantages of a small molecule catalyst. The design of a peptide for catalytic nucleophilic fluorination would be synthetically valuable and highly instructive because our knowledge on peptide-fluoride binding is currently limited to *in silico* studies.^5^ This state of play is astonishing, and in stark contrast to various studies on the interactions of halides other than fluoride with peptides^6^ and proteins,^7^ aimed at understanding biological processes such as pH regulation, protein structure and assembly, or neuron signaling. Here, we report an experimental study investigating how peptidic scaffolds can be conformationally tuned to bind fluoride. We also provide preliminary data demonstrating that peptide-based constructs can serve as phase-transfer catalysts for asymmetric nucleophilic substitution with CsF (Figure 1, *right*).

In exploratory studies, we considered short tetramers with both *N*- and *C*-terminal caps intended to ensure solubility in organic solvents for in-depth NMR spectroscopic studies. We reasoned that a closely positioned H-bonding network may be suited for fluoride chelation via H-bonds. To this end, amino acid sequence motifs that provoke secondary peptide structures such as β-turn-inducing Pro-Acpc^8^ were selected, aiming at controlling the microenvironment around fluoride. Based on the aforementioned considerations, the proline-containing tetrapeptide **1a** served as lead structure in our study on binding to soluble fluoride source TBAF·3H_2_O, as well as insoluble CsF with application to HB-PTC in mind (Figure 2A).

**Figure 2:**
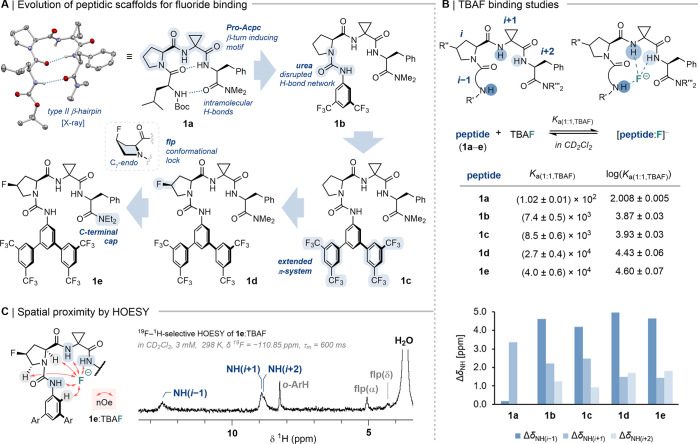
(*A*) Structural editing of peptidic fluoride chelators. (*B*) NMR titrations with TBAF·3H_2_O: Binding affinities for 1:1 HBD:TBAF complexes calculated using DynaFit 4 ^11^ (*n* = 2), and Δδ_NH_ between unbound and TBAF·3H_2_O-saturated **1a**–**e** (∼10 equiv TBAF·3H_2_O for **1a**). (*C*) ^19^F–^1^H-selective HOESY NMR spectrum of **1e**:TBAF.

Titrations^9^ of **1a** with TBAF·3H_2_O in CD_2_Cl_2_ revealed a 1:1 binding mode to fluoride with moderate affinity (*K*_a(1:1,TBAF)_ = (1.02 ± 0.01) × 10^2^ M^–1^). Over the course of the titration, δ_NH(*i*+1)_ has shifted downfield by +3.37 ppm at ∼ 10 equiv of fluoride added, while δ_NH(*i*–1)_ and δ_NH(*i*+2)_ remained largely unperturbed, suggesting no significant interactions for these two NH groups with fluoride (Δδ_NH_ = +0.19 and +0.02 ppm, respectively; Figure 2B). The monodentate binding mode for **1a**:TBAF implies retention of a type II β-hairpin featuring intramolecular H-bonds between Leu­(*i*–1) and Phe­(*i*+2). This secondary structure was observed in the solid-state for unbound **1a** as determined by single-crystal X-ray diffraction (Figure 2A). To enhance fluoride binding, we considered **1b** and **1c** featuring an electron-deficient *N*-terminal urea cap to disrupt intramolecular H-bonding, thereby favoring multidentate binding to fluoride. We selected the urea motifs 3,5-(CF_3_)_2_C_6_H_3_NHC­(O)- (**1b**) and 3,5-[3,5-(CF_3_)_2_­C_6_­H_3_]_2_­C_6_­H_3_­NHC­(O)- (**1c**), effective for BINAM-urea catalysts;^4a,10^ the terphenyl group imparting conformational rigidity through π-π stacking. Pleasingly, the *N*-terminal urea cap resulted in tridentate fluoride binding for both **1b** and **1c**, as suggested by deshielding of all three NH contacts (Δδ_NH_ = 0.96–4.31 ppm; Figure 2B and Table S8) upon saturation with TBAF·3H_2_O (2.0 and 3.0 equiv, respectively). The binding profiles were consistent with 1:1 HBD:TBAF complexation, exhibiting binding constants *K*_a(1:1,TBAF)_ = (7.4 ± 0.5) × 10^3^ M^–1^ and (8.5 ± 0.6) × 10^3^ M^–1^ for **1b** and **1c**, respectively. Further conformational tuning took advantage of (4*S*)-fluoroproline (flp),^12^ known to reinforce β-turns due to C_γ_-*endo* ring puckering and disfavored n_O_(*i–*1)→π_C=O_*­(*i*) interactions between consecutive carbonyls.^13^ This structural editing of Pro­(*i*) (**1d**) as well as *C*-terminal cap modification to –NEt_2_ (**1e**) retained tridentate fluoride binding with *K*_a(1:1,TBAF)_ = (2.7 ± 0.4) × 10^4^ M^–1^ and (4.0 ± 0.6) × 10^4^ M^–1^, and proved fruitful for catalysis (*vide infra*). ^1h^ *J*_NH···F_– couplings were not detected for TBAF-bound complexes studied herein, suggesting fast dynamic exchange. In order to probe NH···F^–^ contacts in solution, nuclear Overhauser effects (nOe) developed between fluoride and NH groups of 1:1 **1e**:TBAF complex (3 mM, CD_2_Cl_2_) were studied by ^19^F–^1^H HOESY NMR.^14^ The spectrum revealed three NH···F^–^ correlations in addition to spatial correlations of fluoride with ureido *ortho*-aryl protons (*o*-ArH), and with α- and δ-protons of the flp residue (Figure 2C).

With the knowledge gained from TBAF-binding studies, we investigated the ability of **1a–e** to act as H-bond donor (HBD) phase-transfer catalysts for the enantioselective fluorination of β-haloamines. This substrate class was selected for their propensity to ionize into *meso*-aziridinium ions (R_4_N^+^), a process leading to the reactive ion-pair [R_4_N^+^HBD·F^–^] for fluoride delivery;^10^ such ion-pair should bear resemblance to the [*n*Bu_4_N^+^HBD·F^–^] complex derived from HBD binding to TBAF. The catalytic performance of **1a–e** was assessed with racemic *trans*-*N*,*N*-dibenzyl-2-bromocyclohexan-1-amine (*rac*-**2a**) (Figure 3A), a challenging substrate for fluorination with CsF and BINAM-urea catalysts under HB-PTC conditions.^4a,10^ In the absence of HBD catalyst, no fluorination was observed because CsF is insoluble in 1,2-dichloroethane (1,2-DCE). Addition of 10 mol % tetrapeptide **1a** gave the fluorinated product **3a** in 18% yield with no enantiocontrol (50:50 *e.r.*). Considering that this tetrapeptide was soluble in 1,2-DCE, the low yield may stem from weak monodentate binding of fluoride (*vide supra*). Peptidic scaffolds **1b** and **1c** showed improved enantiocontrol (65:35 and 71:29 *e.r.*, respectively), suggesting that fluoride sits in a chiral H-bonding network. The low yields of 22% and 18%, respectively, for these catalysts were attributed to their low solubility in 1,2-DCE (4.4 g·L^–1^ for **1b** and 1.1 g·L^–1^ for **1c**, at 20 *°C;* Supporting Information). Indeed, the Gly­(*i*+1) analogue of **1c**, which was found to be soluble under the reaction conditions, provided (*S*,*S*)-**3a** in 76% yield with a similar level of enantioinduction (70:30 *e.r.*, Table S2, **1c-Gly­(** *i* **+1)**). The importance of the stereochemistry of the Pro­(*i*) residue was highlighted when comparing **1c-Gly­(** *i* **+1)** to its d-Pro­(*i*) epimer (**1c-** d **-Pro­(** *i* **)-Gly­(** *i* **+1)**), the latter leading to near-racemic product **3a** in 42% yield (46:54 *e.r.*, Table S2). Installation of conformationally locked (C_γ_-*endo*) (4*S*)-fluoroproline (flp) in **1d** increased both enantioselectivity (85:15 *e.r.*) and yield of fluorination (84%). Comparative studies under similar conditions with **1d** **-Gly­(** *i* **+1)** or **1d-Flp­(** *i* **)-Gly­(** *i* **+1)** (Flp = (4*R*)-fluoroproline) led to lower enantioselectivity of 80:20 and 59:41 *e.r.*, respectively (Table S2). Modification of the *C*-terminal cap (−NEt_2_; **1e**) resulted in (*S*,*S*)-**3a** in 86% yield and 88:12 *e.r.* Pleasingly, performing the reaction on gram-scale afforded (*S*,*S*)-**3a** isolated in 83% yield with retained enantiocontrol (89:11 *e.r.*, 5 mol % **1e**, 120 h; Figure 3B).

**Figure 3:**
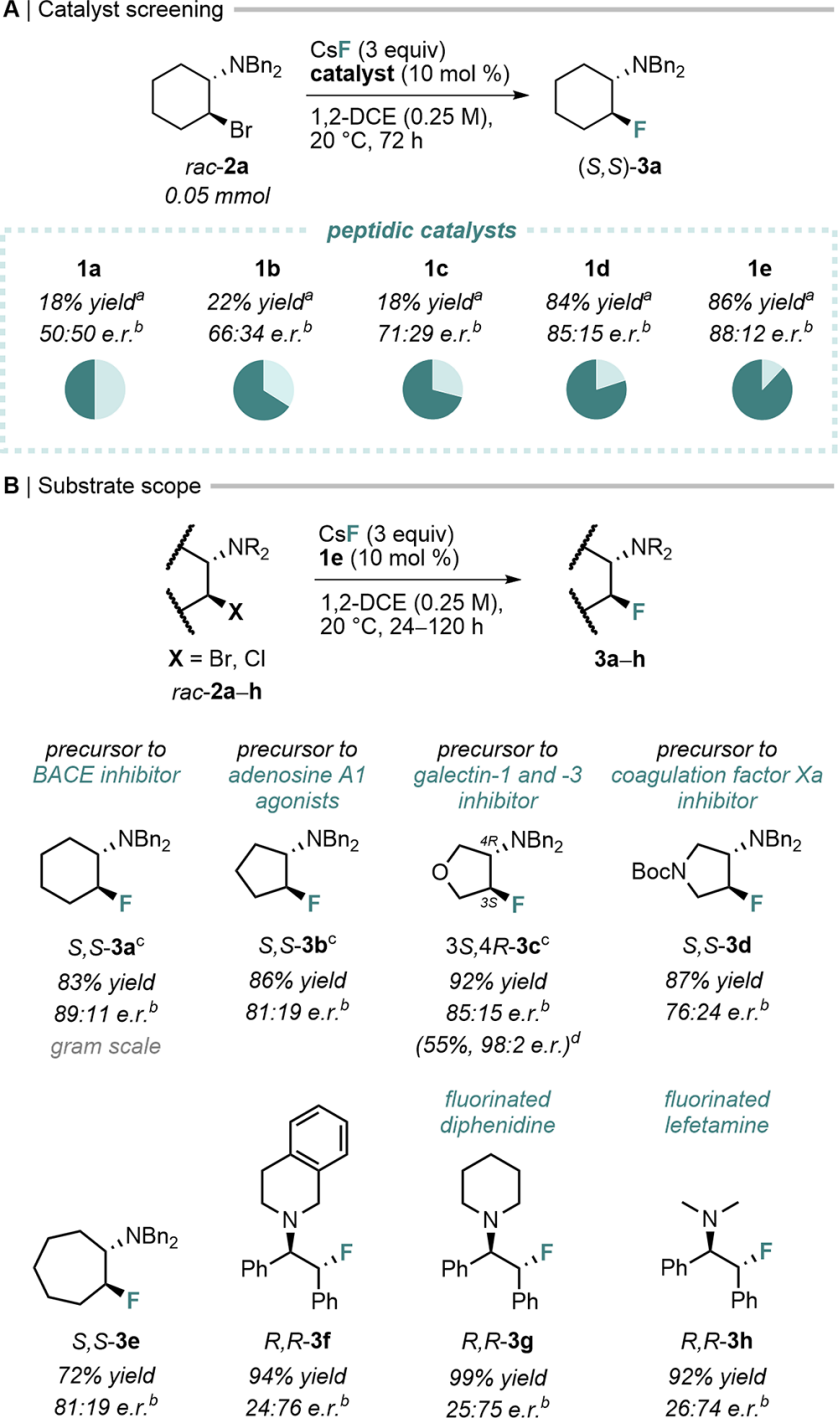
(*A*) Catalytic evaluation of peptidic catalysts for asymmetric nucleophilic fluorination, and (*B*) substrate scope. ^*a*^Determined by ^19^F qNMR; ^*b*^enantiomeric ratio (*e.r.*) measured by chiral HPLC; ^*c*^5 mol % **1e**; ^*d*^recrystallized from *i*PrOH as trichloroacetate salt.

Various β-haloamines were subjected to fluorination conditions with peptidic catalyst **1e**. Overall, cyclic (**2a**–**e**) performed better than stilbene-based substrates (**2f**–**h**), a trend contrasting with BINAM-urea catalysts^10^ (Figure S1). Precursors to bioactive compounds such as β-secretase enzyme (BACE) inhibitor^15^ (**3a**), adenosine A1 agonists^16^ (**3b**), galectin-1 and -3 inhibitor^17^ (**3c**), and coagulation factor Xa inhibitor^18^ (**3d**) were obtained in high yields (up to 92%) and moderate enantioselectivities (Figure 3B). Notably, **3c** was successfully recrystallized as trichloroacetate (TCA) salt to provide (3*S,*4*R*)-**3c**·TCA in 98:2 *e.r.*, 55% yield over two steps.

The superiority of **1e** as a catalyst for asymmetric nucleophilic fluorination prompted a study on its ability to bind CsF. Saturation of **1e** (25 mM) with CsF in CD_2_Cl_2_ showed extensive line broadening in the ^1^H NMR spectrum at 298 K, indicating solubilization and complexation of CsF with fast equilibration of several species (Figure 4A). Disappearance of NH­(*i*–1) and NH­(*i*+2) peaks was observed, while the resonance of NH­(*i*+1) remained unperturbed, suggesting bidentate binding to fluoride. At lower temperature (233 K), the ^1^H NMR spectrum revealed two diagnostic doublets indicating the presence of two H-bonds to fluoride. The observation of a single broad peak by both ^19^F (−60.7 ppm) and ^133^Cs NMR (+43.1 ppm) suggested the presence of one HBD:fluoride complex observable by NMR.

**Figure 4:**
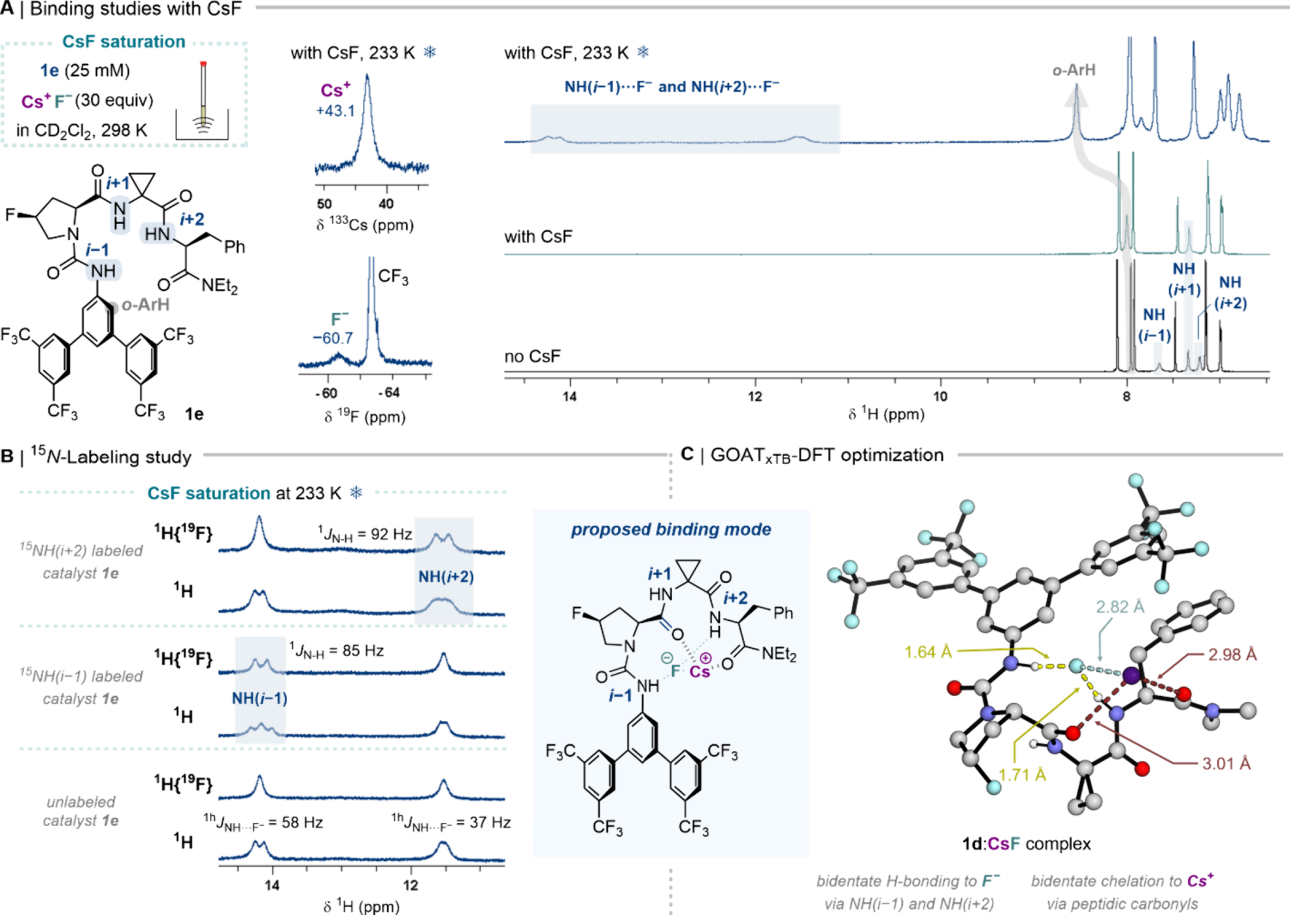
(*A*) ^1^H, ^19^F, and ^133^Cs NMR studies for **1e** under CsF saturation. (*B*) ^15^ *N*-Labeling study of **1e** for identification of NH contacts. (*C*) Computational model of the **1d**:CsF complex at the M06–2X-D3/def2-TZVP; def2-TZVPPD­[Cs^+^,F^–^]­(SMD = CH_2_Cl_2_)//M06-L-D3/def2-SVP;def2-TZVPPD­[Cs^+^,F^–^]­(SMD = CH_2_Cl_2_) level of theory.

The assignment of doublets in the ^1^H NMR spectrum was established by ^15^ *N*-labeled variants **1e-** ^**15**^ **NH­(** *i*–**1)** and **1e-** ^**15**^ **NH­(** *i*+**2** **)** (Figure 4B). ^1^H­{^19^F} NMR experiments of the corresponding CsF complexes revealed ^1^ *J*_H–N_ heteronuclear couplings, which enabled unambiguous assignment of the resonances as NH­(*i*–1) (13.64 ppm, ^1h^ *J*_NH···F_– = 55 Hz, ^1^ *J*_H–N_ = 85 Hz) and NH­(*i*+2) (11.81 ppm, ^1h^ *J*_NH···F_– = 40 Hz, ^1^ *J*_H–N_ = 92 Hz). It has been previously shown that ^1h^ *J*_NH···F_– values for HBD:CsF complexes correlate with NH···F^–^ internuclear distances measured from solid-state structures,^9^ suggesting relative H-bonding distances for **1e** as NH­(*i*–1)···F^–^ < NH­(*i*+2)···F^–^, and no H-bonding interaction with NH­(*i*+1).

Bidentate fluoride binding within complex **1e**:CsF differs from tridentate binding of TBAF·3H_2_O, highlighting the impact of the countercation (TBA^+^ versus Cs^+^) on fluoride chelation.^9^ The Cs^+^-chelating ability of cyclic peptides,^19^ or large proteins,^20^ via cation-carbonyl and cation-π interactions are well-documented. Further investigations of the effect of Cs^+^ involved computational studies of the solution-phase CsF-complex of **1d** (computationally less demanding analog of **1e**). Experimentally, **1d** displayed similar binding to TBAF (tridentate fluoride coordination) and CsF (bidentate fluoride binding) compared to **1e** (Supporting Information). Initial conformational search via a Global Optimization Algorithm (GOAT)^21^ for **1d**:CsF resulted in GFN2-xTB conformers ranging from no chelation of fluoride to tridentate coordination. Subsequent DFT calculations reinforced the energetic preference for bidentate H-bonding to fluoride with NH­(*i*–1)···F^–^(1.64 Å) < NH­(*i*+2)···F^–^(1.71 Å), in line with experimental NMR data (Figure 4C). The lowest-energy conformer of **1d**:CsF complex featured bidentate chelation to Cs^+^ via two carbonyls (2.98 and 3.01 Å), with the Cs^+^···F^–^ pair remaining in close proximity (2.82 Å). An alternative conformation with tridentate binding to fluoride resulted in a complex that is 11.6 kcal·mol^–1^ higher in energy, which could be attributed to a dissociated Cs^+^···F^–^ pair (4.73 Å; Figure S22).

Our previous work revealed that BINAM-ureas exhibited tridentate binding to fluoride as determined by single-crystal X-ray crystallography and MD-DFT computational studies, irrespective of the cation.^4a,9,10^ DFT structural studies also indicated that the cation (Cs^+^ or TBA^+^) does not significantly alter the structure of the **U1**:F^–^ complex, with tridentate fluoride binding maintained. Additionally, Cs^+^···F^–^ ion-pairing and Cs^+^···π interaction^22^ were observed for the **U1**:CsF complex (Figure 5, *left*).^4a^ For comparison, **1d**:CsF displayed Cs^+^ chelation to peptidic carbonyl groups, and no Cs^+^···π interactions, based on DFT calculations (Figure 5, *right*).

**Figure 5:**
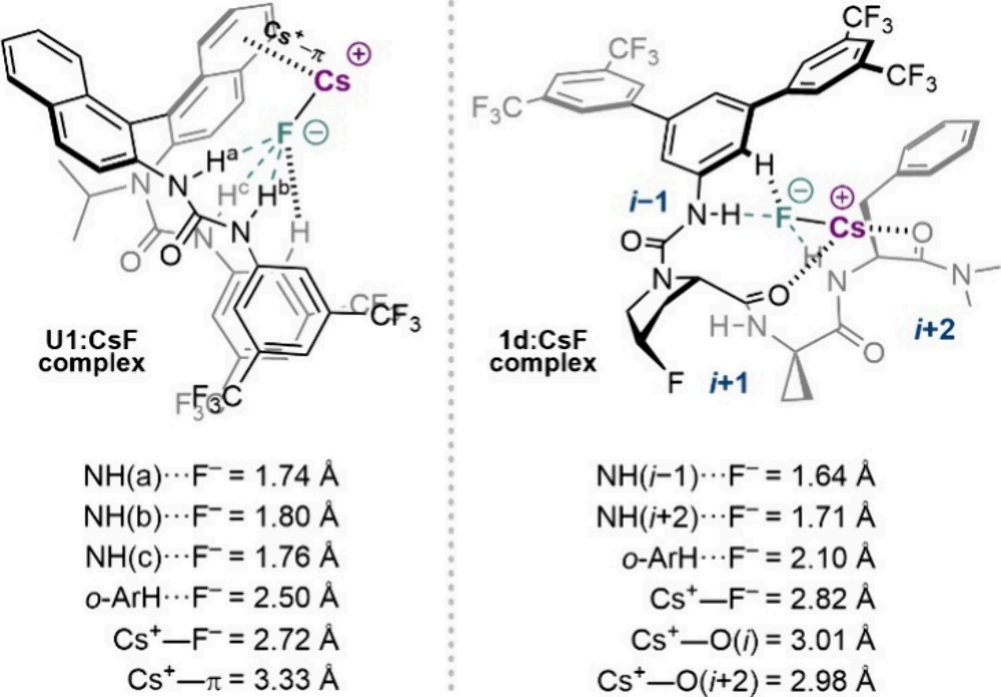
Comparison of CsF binding to BINAM-urea (**U1**)^4a^ and peptide-based (**1d**) catalysts in CH_2_Cl_2_ via DFT calculations.

Taken together, these results are instructive at various levels. For the first time, peptidic HBDs are studied for their ability to bind fluoride in an organic solvent via backbone NH groups. NMR titrations with TBAF of a short conformationally rigid model peptide highlighted the competition between intramolecular H-bonding and fluoride binding, the latter being favored upon structural editing with a urea motif. Further conformational tuning upon replacement of l-proline with (4*S*)-fluoro-l-proline enabled control over the denticity of peptidyl ureas for TBAF complexation from mono- to tridentate, and enhanced binding affinity. This work also highlights the importance of the countercation for the fluoride binding mode as illustrated with the preferential formation of a bidentate complex when TBAF is replaced with CsF. Increased binding affinity of peptide-based catalysts to fluoride, combined with a suitable solubility profile proved fruitful for the fluorination of β-haloamines with CsF (up to 99% yield, 89:11 *e.r.*). Peptidic HBDs are suitable phase-transfer agents for CsF because they serve as chelators of both F^–^ (via NH groups) and Cs^+^ (via carbonyls), as evidenced by NMR spectroscopic studies and GOAT-DFT calculations.

This study suggests that the inherent modularity of peptides shows prospect for targeted optimization for fluoride salt chelation and substrate specificity. More broadly, this work represents a new departure for catalytic fluorination considering the wide chemical space of peptidic catalysts, especially when combined with expert knowledge, innovative screening approaches, and machine-learning workflow.^23^

## Supplementary Material



## Notes

Additional reaction optimization, catalyst synthesis and characterization, NMR methods and characterization, binding studies, computational methods and relevant energies with *xyz* coordinates (PDF)
