# Supplementary material for: Peptidic Catalysts Conformationally Tuned for Fluoride Binding and Delivery
Source: J Am Chem Soc. 2026 Feb 24;148(9):9238–43. doi: 10.1021/jacs.6c01667 (PMC12983308; doi:10.1021/jacs.6c01667)
Supplement: Supplementary file 1 [file ja6c01667_si_001.pdf]

Supporting Information

Peptidic Catalysts Conformationally Tuned for Fluoride Binding and Delivery

Gabija Poškaitė,[a] Thomas Schlatzer,[a] Zijun Chen,[a] Mihai V. Popescu,[b] Robert S. Paton,[b] and Véronique Gouverneur[a,*]

[a] *Chemistry Research Laboratory, University of Oxford, 12 Mansfield Road, Oxford, OX1 3TA, United Kingdom*

[b] *Department of Chemistry, Colorado State University, Fort Collins, Colorado 80528, United States.*

*Correspondence to: veronique.gouverneur@chem.ox.ac.uk

Table of Contents

1. General information 3
2. Catalyst synthesis and characterization 4
   2.1. General procedures for solution phase catalyst synthesis 5
   2.2. Representative synthesis of ureido-peptides 6
   2.3. Preparation and characterization of intermediates 10
   2.4. Characterization of catalysts 14
3. Optimization of reaction conditions 21
   3.1. General reaction conditions for screenings 21
   3.2. Reaction screenings 22
4. Substrate synthesis and characterization 25
   4.1. Alcohols 25
   4.2. Bromides 27
5. Asymmetric nucleophilic fluorination 29
   5.1. General Procedure VI 29
   5.2. Characterization of products 30
   5.3. Comparison with catalyst **U2** 35
6. NMR studies 36
   6.1. Solubility determination for catalyst **1b**, **1c** and **1d**. 36
   6.2. ^{1}H and ^{13}C NMR spectra assignments for **1e** 37
   6.3. NMR titrations of catalysts **1a−1e** 41
   6.4. **1e**–TBAF Complex: HOESY NMR 49
   6.5. **1e**–CsF Complex: NMR at low temperature 50
   6.6. CsF Complexation with **1a−1d** 53
7. Computational studies 58
   7.1. Computational methods 58
   7.2. Binding modes of **1d:CsF** 59
   7.3. Binding modes of **1a:CsF** 61
   7.4. Cartesian coordinates of **1d:CsF (C)**, **1d:CsF (A)**, and **1a:CsF (F)** 63
8. Crystallographic information: structure of **1a** 67
9. NMR spectra 69
   9.1. NMR spectra of catalysts and peptide intermediates 69
   9.2. NMR spectra of substrates, their intermediates and products 93
10. HPLC traces 104
11. References 113

1. General information

The three-letter abbreviations in accord with the *IUPAC-IUB* were used for proteinogenic amino acids. Modified amino acids were abbreviated as follows: 1-amino-1-cyclopropane carboxylic acid (Acpc), (2*S*,4*S*)-4-fluoropyrrolidine-2-carboxylic acid (flp) and (2*S*,4*R*)-4-fluoropyrrolidine-2-carboxylic acid (Flp).

Unless otherwise stated, all reagents were purchased from commercial suppliers and used without further purification. Unless otherwise stated, solvents were used without prior drying/degassing. Reactions requiring anhydrous conditions are clearly stated and were conducted after flame-drying of reaction flasks under an inert atmosphere of nitrogen. CsF (99.9 % trace metal basis from Sigma-Aldrich) was ground prior to reactions and used without pre-drying. Reactions were monitored by thin layer chromatography (TLC) on silica gel pre-coated aluminium sheets (Merck Kieselgel 60 F_{254} plates). Visualization was accomplished by irradiation with UV light at 254 nm, and/or ninhydrin stain, and/or permanganate stain. Column chromatography was performed on Merck silica gel (60, particle size 0.040-0.062 mm). Optical rotations were measured on an Autopol L 2000 (Schmidt-Haensh) at 589 nm, 25 °C. Data are reported as $[\alpha]_D^{25℃}$, concentration (c in g/100 mL) and solvent. High resolution mass spectra (HRMS, *m/z*) were recorded on an ACQUITY I-Class PLUS UPLC System (Waters, Milford, MA, USA) coupled to an ACQUITY RDa mass spectrometer (Waters, Milford, MA, USA) equipped with an ESI probe, in positive ion mode. Infrared spectra were recorded for neat compounds or in solution using a Bruker Tensor 27 FT-IR spectrometer. Absorptions (ν) are reported in wavenumber (cm^{-1}). Melting points (m.p.) of solids were measured on a Griffin apparatus and are uncorrected. The enantiomeric ratios (*e.r.*) were determined by HPLC analysis on a Shimadzu *i*-Prominence LC-2030 (PDA detector) using a chiral stationary phase column as specified.

All NMR spectra were recorded on Bruker AVIIIHD 400, NEO 400, AVIIIHD 500 or AVIII 600. The spectra were acquired at 298 K or at low temperature (stated where applicable) using a BCU-I/II fridge units with liquid nitrogen heat exchanger on AVIIIHD 500. CD_2Cl_2 was stored at 4 ˚C. $CDCl_3$ was stored at room temperature. MeOD-d_4 was stored at room temperature under nitrogen environment. Spectra were processed with Topspin 4.0.7, spectral data are reported as follows: chemical shift, multiplicity (singlet

(s), doublet (d), triplet (t), quartet (q), quintet (quint), sextet (sx), septet (sept), multiplet (m), or combination of those), coupling constant (Hz) and relative integration (for ^{1}H NMR only), br (broad) indicates significant signal broadening. Coupling constants, *J*, are reported in Hz to the nearest 0.1 Hz. ^{1}H and ^{13}C NMR spectral data are reported as chemical shifts (*δ*) in parts per million (ppm) relative to the solvent peak using the Bruker internal referencing procedure (*edlock*). ^{19}F NMR spectra are referenced relative to $CFCl_3$ using the Bruker internal referencing procedure. Non-standard pulse sequences and acquisition parameters are clearly stated in the text according to the following naming convention. NS = number of scans, DS = number of dummy scans, TD = time domain points [pt], SI = spectrum data size, SW = spectral window [ppm], o1p = spectrum offset, centre of spectrum [ppm], o2p = selection of second nuclei [ppm].

2. Catalyst synthesis and characterization

Catalyst **U2**[1] was prepared following published procedures. The solution phase peptide synthesis of catalysts **1a**–**1e** (listed in **Table S1**) was accomplished using the *N*-*tert*-butoxycarbonyl (Boc) group strategy.[2] Once synthesized, peptidic catalysts were stored at 0 °C to prevent epimerization and other adverse side-reactivity. Yields were not optimized. A representative synthesis procedure for catalyst **1e**, full characterization data for catalysts **1a**–**1e**, and preparation of not commercially available intermediates **S1**–**S9**, are provided below.

Table S1. Synthesized catalysts **1a**-**1e**.

N-cap	*i-1*	*i*	*i+1*	*i+2*	*C*-cap	Compound no.
Boc-	Leu	Pro	Acpc	Phe	NMe_2	**1a**
**3,5-$(CF_3)_2C_6H_3$NHC(O)-**	-	Pro	Acpc	Phe	NMe_2	**1b**
**3,5-[3,5-$(CF_3)_2C_6H_3]_2C_6H_3$NHC(O)-**	-	Pro	Acpc	Phe	NMe_2	**1c**
3,5-[3,5-$(CF_3)_2C_6H_3]_2C_6H_3$NHC(O)-	-	Pro	**Gly**	Phe	NMe_2	**1c-Gly(*i*+1)**
3,5-[3,5-$(CF_3)_2C_6H_3]_2C_6H_3$NHC(O)-	-	**D-Pro**	Gly	Phe	NMe_2	**1c-D-Pro(*i*)-Gly(*i*+1)**
3,5-[3,5-$(CF_3)_2C_6H_3]_2C_6H_3$NHC(O)-	-	**flp**	**Acpc**	Phe	NMe_2	**1d**
3,5-[3,5-$(CF_3)_2C_6H_3]_2C_6H_3$NHC(O)-	-	flp	Gly	Phe	NMe_2	**1d-Gly(*i*+1)**
3,5-[3,5-$(CF_3)_2C_6H_3]_2C_6H_3$NHC(O)-	-	**Flp**	Gly	Phe	NMe_2	**1d-Flp(*i*)-Gly(*i*+1)**
3,5-[3,5-$(CF_3)_2C_6H_3]_2C_6H_3$NHC(O)-	-	flp	Acpc	Phe	**NEt_2**	**1e**
3,5-[3,5-$(CF_3)_2C_6H_3]_2C_6H_3$**^{15}N**HC(O)-	-	flp	Acpc	Phe	NEt_2	**1e-^{15}NH(*i*–1)**
3,5-[3,5-$(CF_3)_2C_6H_3]_2C_6H_3$NHC(O)-	-	flp	Acpc	Phe(**^{15}N**)	NEt_2	**1e-^{15}NH(*i*+2)**

2.1. General procedures for solution phase catalyst synthesis

General Procedure I: peptide coupling

H-peptide-NR_2·HCl (1.0 mmol), Boc-peptide-OH (1.2 mmol) and HOBt·H_2O (1.2 mmol) were dissolved in CH_2Cl_2 (5 mL) at 0 °C and allowed to stir for 5 min, followed by addition of EDC·HCl (1.2 mmol) and drop-wise addition of DIPEA (2.5 mmol). The reaction mixture was allowed to stir for 2–24 hours at room temperature (monitored *via* TLC or LC-MS). The solution was diluted with CH_2Cl_2 (10 mL) and the organic layer was washed sequentially with distilled water (3 × 15 mL), 1 M HCl (2 × 15 mL), sat. aq. $NaHCO_3$ (2 × 15 mL), and brine (1 × 15 mL). The organic layer was dried with Na_2SO_4, filtered and concentrated *in vacuo*, which was carried forward without further purification, unless otherwise stated.

General Procedure II: Boc-deprotection

Boc-peptide-NR_2 (1.0 mmol) was dissolved in 4.0 M HCl in dioxane (3 mL) and stirred vigorously for 1 h at r.t. Then the solvent was removed *in vacuo*, followed by co-evaporation with CH_2Cl_2 (3 × 10 mL). The resulting crude product was thoroughly dried under reduced pressure and carried forward without further purification.

General Procedure IIIa: urea formation

H-peptide-NR_2 (1.0 mmol) was dissolved in anhydrous CH_2Cl_2 (5 mL) followed by the addition of NEt_3 (1.5 mmol) under an atmosphere of N_2. The mixture was cooled to 0 °C and Ar-NCO (1.2 mmol) was added dropwise. The reaction mixture was stirred at 0 °C until complete consumption of the starting material as indicated by TLC. Excess isocyanate was quenched by the addition of MeOH (0.5 mL) before the reaction mixture was allowed to warm to r.t. Solvents were removed under reduced pressure and the crude mixture was purified by column chromatography.

General Procedure IIIb: urea formation

H-peptide-NR_2 (1.0 mmol) was dissolved in a mixture of anhydrous THF and pyridine (10 mL, v/v 25:3) under an atmosphere of N_2. The mixture was cooled to 0 °C followed by addition of Ar-NCO (1.1 mmol). The reaction mixture was stirred at 0 °C until complete consumption of the starting material as indicated by TLC. Excess isocyanate was quenched by the addition of MeOH (0.5 mL) before the reaction mixture was

allowed to warm to r.t. Solvents were removed under reduced pressure and the crude mixture was purified by column chromatography.

2.2. Representative synthesis of ureido-peptides

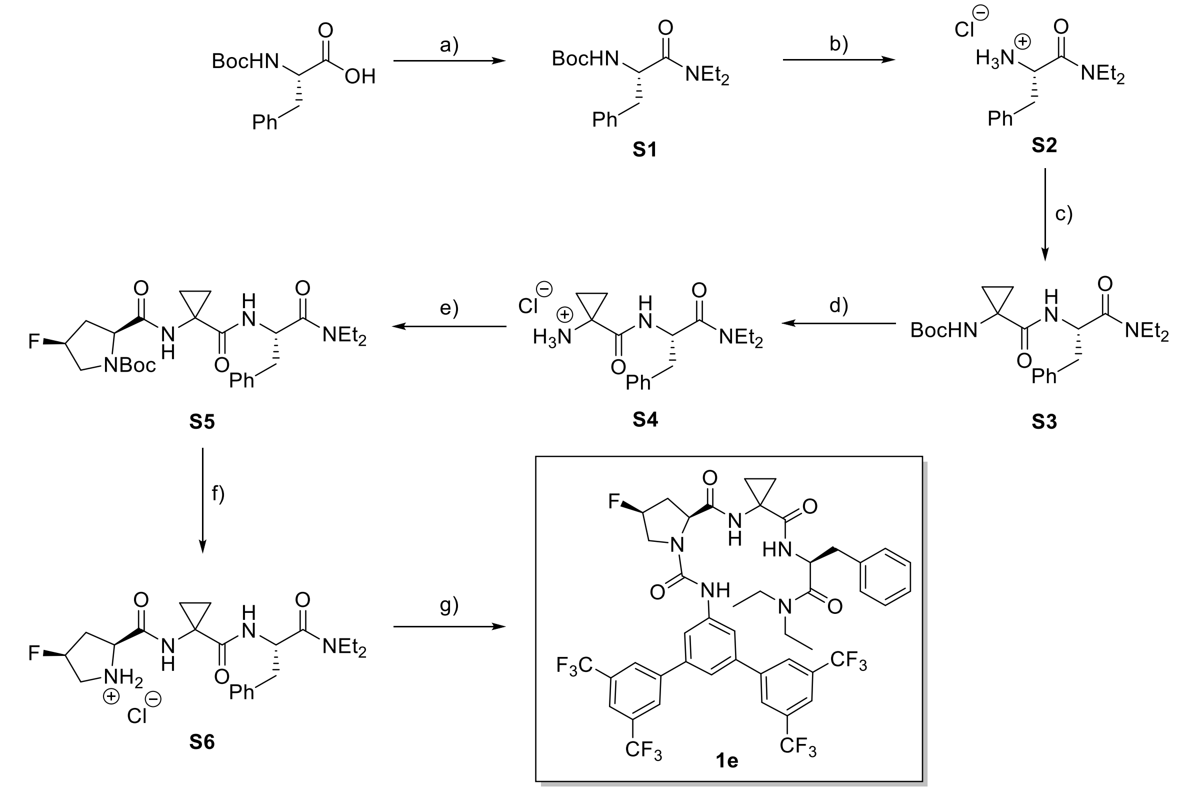

Scheme 1. Representative synthesis for the preparation of lead catalyst 3,5-[3,5-$(CF_3)_2C_6H_3]_2C_6H_3$NHCO-flp-Acpc-Phe-NEt_2 (**1e**). a) $HNEt_2$ (2.0 equiv.), EDC·HCl (1.2 equiv.), HOBt·H_2O (1.2 equiv.), DIPEA (2.5 equiv.), CH_2Cl_2 (0.2 M), 2 h; b) HCl (4.0 M in dioxane), 1 h; c) Boc-Acpc-OH (1.2 equiv.), EDC·HCl (1.2 equiv.), HOBt·H_2O (1.2 equiv.), DIPEA (2.5 equiv.), CH_2Cl_2 (0.2 M), 2 h; d) HCl (4.0 M in dioxane), 1 h; e) Boc-flp-OH (1.2 equiv.), EDC·HCl (1.2 equiv.), HOBt·H_2O (1.2 equiv.), DIPEA (2.5 equiv.), CH_2Cl_2 (0.2 M), 24 h; f) HCl (4.0 M in dioxane), 1 h; g) 3,5-[3,5-$(CF_3)_2C_6H_3]_2C_6H_3$NCO (1.2 equiv.), THF/pyridine 25:3 (0.1 M), 0 °C, overnight.

Boc-Phe-NEt_2 (S1)

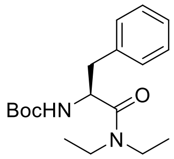

The title compound was prepared following *General Procedure I* using commercially available Boc-Phe-OH (5.32 g, 20.0 mmol) and diethylamine (4.1 mL, 40.0 mmol). The title compound was obtained as a yellow oil (6.40 g, quant.). **^{1}H NMR** (400 MHz, $CDCl_3$): δ 7.27–7.19 (m, 5H), 5.36 (d, J = 8.6 Hz, 1H), 4.74 (q, J = 8.3 Hz, 1H), 3.52 (sx, J = 6.9 Hz, 1H), 3.08 (sx, J = 7.1 Hz, 1H), 3.04–2.90 (m, 4H), 1.41 (s, 9H), 1.04 (t, J = 7.1 Hz, 3H), 0.97 (t, J = 6.9 Hz, 3H) ppm; **^{13}C NMR** (101 MHz, $CDCl_3$): δ 170.9, 155.0, 136.6, 129.6, 128.4, 126.8, 79.6, 51.4, 41.6, 40.5, 40.4, 28.3, 14.2, 12.8 ppm; **IR** (thin layer film): ν 3291, 2978, 2936, 1708, 1635, 1526, 1494, 1454, 1391, 1366, 1221, 1173, 1081, 1051, 1020, 978, 914, 888, 867, 800, 783, 752, 737, 701, 662, 649, 625 cm^{-1}; $[\alpha]_D^{20°C}$ = +7.5 (c 0.9,

$CHCl_3$); **HRMS** (ESI+) = *m/z* calculated for $C_{18}H_{28}N_2O_3Na^+$ $[M+Na]^+$ 343.1992, found 343.1991. Spectroscopic data were in agreement with the literature values.[3]

Note: Some low intensity broad peaks were observed in ^{1}H NMR indicating the presence of Boc-rotamers (5.08, 4.56, 1.37 ppm); 100:1 ratio.

H-Phe-NEt₂·HCl (S2)

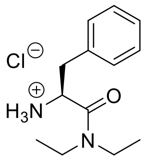

The title compound was prepared following *General Procedure II* from **S1** (6.37 g, 19.9 mmol) using 4 M HCl in dioxane (40 mL). Compound **S2** was obtained as a white solid (5.10 g, quant). **^{1}H NMR** (400 MHz, $CDCl_3$): δ 8.74 (s, 3H), 7.33–7.23 (m, 5H), 4.68 (br quint, *J* = 4.7 Hz, 1H), 3.64–3.55 (m, 2H), 3.24 (dd, *J* = 12.6, 10.3 Hz, 1H), 2.99 (sx, *J* = 6.8 Hz, 1H), 2.89 (sx, *J* = 7.1 Hz, 1H), 2.69 (sx, *J* = 7.1 Hz, 1H), 1.04 (t, *J* = 7.1 Hz, 3H), 0.95 (t, *J* = 7.0 Hz, 3H) ppm; **^{13}C NMR** (101 MHz, $CDCl_3$): δ 167.4, 134.4, 130.0, 128.6, 127.5, 51.3, 41.5, 40.6, 38.1, 13.7, 12.6 ppm; **IR** (thin layer film): *v* 3505, 3460, 2976, 2935, 2877, 1646, 1496, 1457, 1383, 1362, 1304, 1268, 1215, 1150, 1100, 912, 762, 732, 701, 646 cm^{-1}. $[\boldsymbol{\alpha}]_{\mathbf{D}}^{\mathbf{20}°C}$ = +68.6 (*c* 0.9, $CHCl_3$); **HRMS** (ESI+) = *m/z* calculated for $C_{13}H_{21}N_2O^+$ $[M+H]^+$ 221.1648, found 221.1654.

Note: Highly hydroscopic solid.

Boc-Acpc-Phe-NEt₂ (S3)

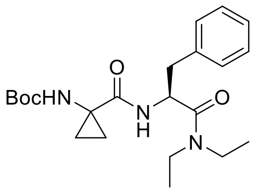

The title compound was prepared following *General Procedure I* using **S2** (4.40 g, 17.1 mmol) and Boc-Acpc-OH (4.13 g, 20.5 mmol). The reaction yielded **S3** as a white gum (6.74 g, 98%). **^{1}H NMR** (400 MHz, $CDCl_3$): δ 7.28–7.19 (m, 6H), 5.17 (s, 1H), 5.03 (q, *J* = 8.2 Hz, 1H), 3.50 (sx, *J* = 7.2 Hz, 1H), 3.07 (sx, *J* = 7.2 Hz, 1H), 3.06–2.87 (m, 4H), 1.61–1.55 (m, 1H), 1.53–1.46 (m, 1H) 1.46 (s, 9H), 1.07–0.97 (m, 2H), 1.03 (t, *J* = 7.2 Hz, 3H), 0.92 (t, *J* = 6.9 Hz, 3H) ppm; **^{13}C NMR** (101 MHz, $CDCl_3$): δ 171.2 (br), 170.4, 155.3 (br), 136.4, 129.6, 128.4, 126.9, 80.6 (br), 50.6, 41.6, 40.4, 40.2 (br), 35.4 (br), 28.3, 18.3 (br), 17.7 (br), 14.1, 12.8 ppm; **IR** (thin layer film): *v* 3414, 3301, 2979, 2934, 1719, 1635, 1sx 509, 1456, 1413, 1366, 1315, 1250, 1218, 1168, 1073, 1033, 1010, 956, 914, 784, 737, 702, 669, 647, 632 cm^{-1}; $[\boldsymbol{\alpha}]_{\mathbf{D}}^{\mathbf{20}°C}$ = –6.2 (*c* 0.9, $CHCl_3$); **HRMS** (ESI+) = *m/z* calculated for $C_{22}H_{34}N_3O_4^+$ $[M+H]^+$ 404.2544, found 404.2553.

Note: Resonances in ^{13}C NMR spectrum corresponding to Acpc residue appear broad due to presence of Boc-rotamers.

H-Acpc-Phe-NEt$_2$·HCl (S4)

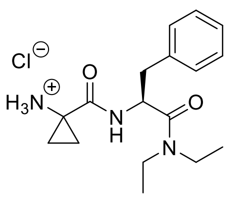

The title compound was prepared following *General Procedure II* from **S3** (6.68 g, 16.5 mmol). H-Acpc-Phe-NEt$_2$·HCl was obtained as a white powder (5.53 g, 99%). **^{1}H NMR** (400 MHz, D_2O): δ 7.40–7.32 (m, 3H), 7.29–7.27 (m, 2H), 5.03 (t, J = 7.6 Hz, 1H), 3.45 (sx, J = 7.1 Hz, 1H), 3.26 (sx, J = 7.2 Hz, 1H), 3.16 (sx, J = 7.1 Hz, 2H), 3.06 (dd, J = 13.8, 7.7 Hz, 1H), 2.99 (dd, J = 13.8, 7.6 Hz, 1H), 1.61–1.57 (m, 1H), 1.48–1.43 (m, 3H), 1.05 (t, J = 7.1 Hz, 3H), 1.03 (t, J = 7.2 Hz, 3H) ppm; **^{13}C NMR** (101 MHz, D_2O): 171.4, 170.1, 136.0, 129.4, 128.8, 127.3, 51.4, 42.4, 41.1, 37.4, 35.0, 13.0, 12.8, 12.7, 11.9 ppm; **m.p.** 233 ˚C; $[\alpha]_D^{20℃}$ = 16.0 (*c* 0.5, H_2O); **HRMS** (ESI+) = *m/z* calculated for $C_{17}H_{26}N_3O_2^+$ $[M+H]^+$ 304.2020, found 304.2014; **IR** (neat): v 3460, 2693, 2648, 1438, 1376, 1308, 1283, 1261, 1094, 971, 748, 701, 634 cm^{-1}.

Boc-flp-Acpc-Phe-NEt$_2$ (S5)

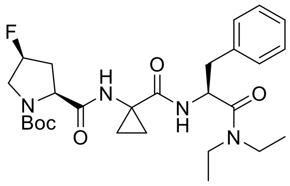

The title compound was prepared following *General Procedure I* using **S4** (5.50 g, 16.2 mmol) and Boc-flp-OH (4.52 g, 19.4 mmol). The crude product was purified via flash column chromatography (98:2 then 90:10, *i*PrOH in CH_2Cl_2) to yield **S5** as a white foam (5.72 g, 68 %). **^{1}H NMR** (500 MHz, $CDCl_3$, **at 333 K**): δ 7.28–7.19 (m, 5H), 7.03 (br s, 1H), 6.86 (br s, 1H), 5.31 (br d, $^2J_{HF}$ = 52.4 Hz, 1H), 5.02 (appt q, J_{HH} = 8.6 Hz, 1H), 4.41 (d, J_{HH} = 10.1 Hz, 1H), 3.90 (ddd, $^3J_{HF}$ = 22.7 Hz, J_{HH} = 13.4, 1.3 Hz, 1H), 3.62 (ddd, $^3J_{HF}$ = 39.3 Hz, J_{HH} = 13.4, 2.7 Hz, 1H), 3.49 (sx, J_{HH} = 7.1 Hz, 1H), 3.11 (sx, J_{HH} = 7.1 Hz, 1H), 3.08–3.00 (m, 3H), 2.92 (dd, J_{HH} = 13.0, 5.5 Hz, 1H), 2.64 (br t, $^3J_{HF}$ = 15.9 Hz, J_{HH} = 14.9 Hz, 1H), 3.08 (br dt, $^3J_{HF}$ = 44.2 Hz, J_{HH} = 11.8 Hz, 1H), 1.71 (br s, 1H), 1.49 (s, 9H), 1.49–1.45 (m, 1H), 1.13 (ddd, J_{HH} = 10.0, 8.1, 4.9 Hz, 1H), 1.04 (t, J_{HH} = 7.1 Hz, 3H), 0.93 (t, J_{HH} = 7.1 Hz, 3H), 0.89 (ddd, J_{HH} = 10.0, 8.0, 4.5 Hz, 1H) ppm; **^{13}C** (126 MHz, $CDCl_3$, **at 333 K**): δ 172.3 (br s), 170.6, 170.4, 154.6 (br s), 136.8, 129.6, 128.3, 126.7, 92.7 (d, $^1J_{CF}$ = 174 Hz), 81.5 (br), 59.9, 53.9 (d, $^2J_{CF}$ = 23 Hz), 50.6, 41.6, 40.4, 39.7, 36.7 (br), 34.5, 28.3, 17.6, 17.2, 13.9, 12.7 ppm; **^{19}F{^{1}H} NMR** (377 MHz, $CDCl_3$, at 298 K, 1.0:0.8 ratio of rotamers): –171.35 (s, major), –172.96 (s, minor) ppm; $[\alpha]_D^{20℃}$ = –43.3 (*c* 0.9, $CHCl_3$); **HRMS** (ESI+) = *m/z*

calculated for $C_{27}H_{40}FN_4O_5^+$ $[M+H]^+$ 519.2977, found 519.2992; **IR** (thin layer film): *v* 3488, 3294, 2979, 2936, 1688, 1640, 1520, 1496, 1455, 1393, 1367, 1317, 1258, 1217, 1169, 1122, 1073, 758 cm^{-1}.

Note: NMR spectra appeared broad due to the presence of rotamers. Hence, spectra were collected at 333 K.

H-flp-Acpc-Phe-NEt₂·HCl (S6)

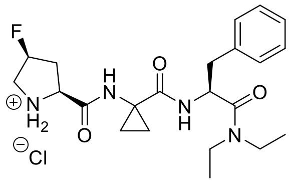

The title compound was prepared following *General Procedure II* from **S5** (5.90 g, 11.4 mmol). **S6** was obtained a white powder (2.27 g, 99% yield). **^{1}H NMR** (500 MHz, $CDCl_3$): *δ* 10.59 (br s, 1H), 9.42 (s, 1H), 8.91 (br s, 1H), 7.59 (br s, 1H), 7.17–7.16 (m, 3H), 6.94 (br s, 2H), 5.31 (d, $^2J_{HF}$ = 52.0 Hz, 1H), 5.30 (br s, 1H), 4.80–4.75 (m, 1H), 3.90–3.75 (m, 2H), 3.41 (sx, J_{HH} = 7.2 Hz, 1H), 3.08 (br t, J_{HH} = 11.5 Hz, 1H), 2.99 (sx, J_{HH} = 7.2 Hz, 1H), 2.92–2.75 (m, 3H), 2.77 (dd, J_{HH} = 12.7, 4.7 Hz, 1H), 2.64 (br t, $^3J_{HF}$ = 18.6 Hz, J_{HH} = 15.0 Hz, 1H), 1.60–1.56 (m, 1H), 1.41–1.38 (m, 1H), 1.18–1.14 (m, 1H), 1.03–0.99 (m, 1H), 0.98 (t, J_{HH} = 7.2 Hz, 3H), 0.79 (t, J_{HH} = 7.2 Hz, 3H) ppm; **^{13}C NMR** (126 MHz, $CDCl_3$): 171.5, 170.9, 170.0, 136.3, 129.7, 128.4, 126.9, 91.5 (*d*, $^1J_{CF}$ = 179 Hz), 59.9, 52.5 (*d*, $^2J_{CF}$ = 24 Hz), 51.4, 41.9, 40.9, 39.3, 37.8 (*d*, $^2J_{CF}$ = 22 Hz), 34.9, 17.4, 17.1, 13.4, 12.6 ppm; **^{19}F{^{1}H} NMR** (471 MHz, $CDCl_3$): –172.74 (s) ppm; $[\alpha]_D^{20℃}$ = 3.5 (*c* 0.9, $CHCl_3$); **HRMS** (ESI+) = *m/z* calculated for $C_{22}H_{32}FN_4O_3^+$ $[M+H]^+$ 419.2453, found 419.2451; **IR** (thin layer film): *v* 3445, 3013, 2936, 1696, 1633, 1519, 1454, 1413, 1383, 1358, 1315, 1217, 1074, 1034, 758 cm^{-1}.

3,5-[3,5-(CF₃)₂C₆H₃]₂C₆H₃NHC(O)-flp-Acpc-Phe-NEt₂ (1e)

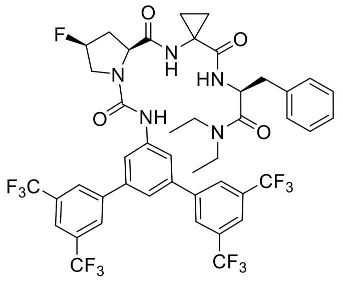

The title compound was prepared following *General Procedure IIIb* from **S6** (455 mg, 1.00 mmol, 1 equiv.). The crude product was purified *via* flash column chromatography (10–40% (v/v) acetone in toluene) to give the title compound **1e** as a white solid (809 mg, 84%). **^{1}H NMR** (500 MHz, CD_2Cl_2): *δ* 8.10 (s, 4H), 7.95 (s, 2H), 7.92 (s, 2H), 7.65 (br s, 1H), 7.48 (s, 1H), 7.32 (s, 1H), 7.20 (d, *J* = 8.2 Hz, 1H), 7.15–7.14 (m, 3H), 7.00–6.98 (m, 2H), 5.46 (dt, *J* = 52.4, 3.5 Hz, 1H), 4.87 (q, *J* = 7.9 Hz, 1H), 4.75 (d, *J* = 9.6 Hz, 1H), 4.16 (dd, *J* = 22.4, 11.6 Hz, 1H), 3.93 (ddd, *J* = 35.2, 11.8, 3.7 Hz, 1H), 3.44 (sx, *J* = 7.0 Hz, 1H), 3.00–2.91 (m, 3H), 2.82–2.76 (m, 2H), 2.71 (dd, *J* = 13.3, 6.5 Hz, 1H),

2.47 (dddd, J = 41.0, 14.7, 10.0, 3.8 Hz, 1H), 1.49–1.40 (m, 2H), 1.09 (ddd, J = 10.2, 7.2, 4.1 Hz, 1H), 0.98 (ddd, J = 10.2, 6.5, 3.2 Hz, 1H), 0.91 (appt q, J = 7.3 Hz, 6H) ppm; **^{13}C NMR** (151 MHz, CD_2Cl_2): δ 172.3, 170.6, 170.3, 155.2, 142.6, 140.6, 139.9, 136.5, 132.0 (q, J = 33 Hz), 129.3, 128.1, 127.5 (br d, J = 3 Hz), 126.6, 122.3 (q, J = 273 Hz), 121.5 (br t, J = 4 Hz), 121.1, 119.6, 92.4 (d, J = 173 Hz), 59.9, 53.9, 50.6, 41.5, 40.3, 39.6, 35.6 (br d, J = 2 Hz), 34.6, 16.5, 16.3, 13.6, 12.3 ppm; **^{19}F{^{1}H} NMR** (471 MHz, CD_2Cl_2): δ –63.07 (12F), –173.39 (1F) ppm; **HRMS** (ESI+) = *m/z* calculated for $C_{45}H_{40}F_{13}N_5O_4Na^+$ $[M+Na]^+$ 984.2765, found 984.2743; $[\boldsymbol{\alpha}]_{\mathbf{D}}^{\mathbf{20^{\circ}C}}$ = –31.2 (*c* 0.9, acetone); **m.p.** 151−152 °C; **IR** (thin layer film): ν 3290, 2926, 2855, 1717, 1649, 1631, 1542, 1468, 1432, 1371, 1280, 1178, 1139, 844, 683 cm^{-1}.

2.3. Preparation and characterization of intermediates

Unless otherwise stated, all amino acid derivatives were purchased from commercial suppliers. Isocyanate 5′-isocyanato-3,3″,5,5″-tetrakis(trifluoromethyl)-1,1′:3′,1″-terphenyl (**S7**) was prepared according to a literature procedure.[4] Boc-[^{15}N]Phe-OH (**S8**) was prepared by adapting a previously published procedure from [^{15}N]Phe-OH.[5] The syntheses of **S8** and [^{15}N]-isocyanate **S9** are described below.

Boc-[^{15}N]Phe-OH (S8)

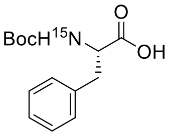

H-[^{15}N]Phe-OH (500 mg, 3.01 mmol) was dissolved in 1,4-dioxane (4.6 mL) and water (2.3 mL), and NaOH (122 mg, 3.04 mmol) was added. The solution was cooled to 0 °C, followed by portion-wise addition of Boc_2O (720 mg, 3.31 mmol). The solution was allowed to warm to r.t. and stirred for 1 h (monitored *via* TLC). The volatiles were evaporated *in vacuo* and the resulting residue was dissolved in water (15 mL), washed with EtOAc (2 × 10 mL). The aqueous phase was then carefully acidified to pH 1–2 using 6 M HCl and extracted with EtOAc (3 x 10 mL). The organic fraction was dried over $MgSO_4$, filtered and evaporated under reduced pressure to give **S8** as colorless crystals (790 mg, 99%). **^{1}H NMR** (400 MHz, $CDCl_3$): δ 7.22–7.11 (m, 5H), 5.35 (dd, $^1J_{H\text{–}N}$ = 91.7 Hz, $J_{H\text{–}H}$ = 8.7 Hz, 1H), 4.75 (br q, J = 7.8 Hz, 1H), 2.93–2.83 (m, 2H), 2.78 (s, 3H), 2.54 (s, 3H), 1.35 (s, 9H) ppm; **^{13}C NMR** (101 MHz, $CDCl_3$): δ 171.6, 155.1 (d, $^1J_{C\text{–}N}$ = 26 Hz), 136.5, 129.4, 128.4, 126.9, 79.6, 51.4 (d, $^1J_{C\text{–}N}$ = 13 Hz), 40.3, 36.8, 35.5, 28.4 ppm; $[\boldsymbol{\alpha}]_{\mathbf{D}}^{\mathbf{20^{\circ}C}}$ = +49.5 (*c* 0.9, $CHCl_3$); **^{15}N NMR** (51 MHz, $CDCl_3$): δ 85.0 (d, $^1J_{N\text{–}H}$ = 92 Hz) ppm;

HRMS (ESI+) = *m/z* calculated for $C_{14}H_{19}[^{15}N]O_4Na^+$ $[M+Na]^+$ 289.1177, found 289.1184. Spectroscopic data consistent with literature reported data.[6]

[^{15}*N*]-5'-(Isocyanato)-3,3'',5,5''-tetrakis(trifluoromethyl)-1,1':3',1''-terphenyl (S9)

S9 was prepared following the route depicted below; compounds **S9a-S9** were prepared following literature procedures for non-labelled analogues.[4, 7]

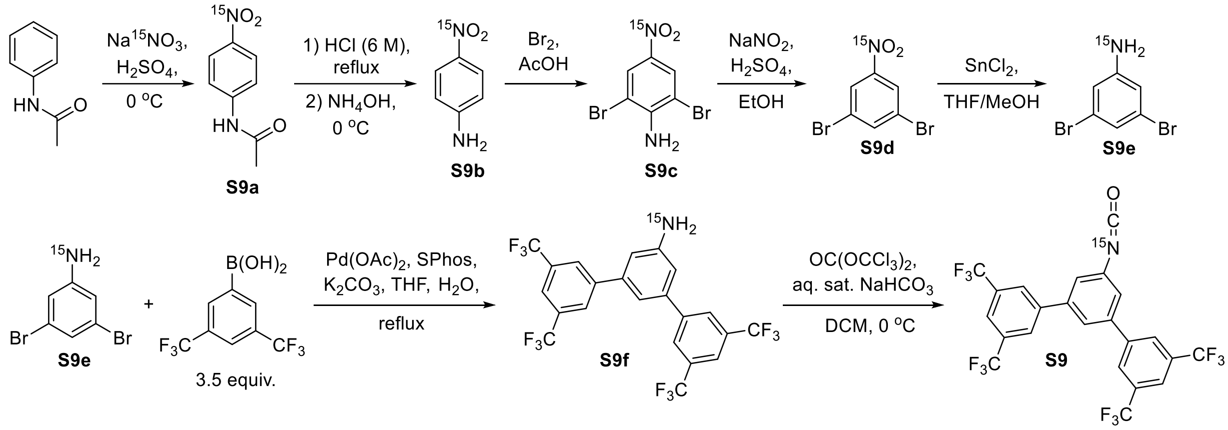

Scheme 2. Multi-step synthesis of **S9**.

N-(4-(Nitro-^{15}*N*)phenyl)acetamide (S9a)

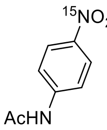

In a 150 mL round-bottom flask, acetanilide (3.00 g, 22.2 mmol) was dissolved in conc. H_2SO_4 (35 mL) and cooled to 0 ˚C, followed by addition of a solution of $Na^{15}NO_3$ (1.91 g, 22.2 mmol) in conc. H_2SO_4 (25 mL) over 20 min. The reaction mixture was stirred at 0 ˚C for 3 h (reaction monitoring *via* GC-MS and TLC) and quenched onto ice (200 mL). The resulting precipitate was collected by filtration, washed with ice-cold H_2O (2 × 50 mL) and dried under a stream of N_2. **S9a** was obtained as a colorless solid (3.87 g, 96%) and was used without further purification. **^{1}H NMR** (400 MHz, DMSO-d_6): δ 10.55 (s, 1H), 8.25–8.16 (m, 2H), 7.86–7.77 (m, 2H), 2.12 (s, 3H) ppm; **^{13}C NMR** (101 MHz, DMSO-d_6): δ 169.4, 145.5, 142.0 (d, J_{CN} = 15 Hz), 125.0 (d, J_{CN} = 2 Hz), 118.5 (d, J_{CN} = 2 Hz), 24.2 ppm; **HRMS** (ESI-) = *m/z* calculated for $C_8H_7N[^{15}N]O_3^-$ $[M-H]^-$ 180.0433, found 180.0417. Spectroscopic data consistent with literature reported data for unlabeled analogue.[8]

4-(Nitro-^{15}*N*)aniline (S9b)

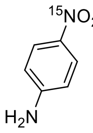

In a 250 mL round-bottom flask, **S9a** (3.80 g, 21.0 mmol) was suspended in 6 M HCl (135 mL) and heated under reflux for 62 h (reaction monitoring *via* GC-MS and TLC). The solution was allowed to cool to r.t. and the pH was

adjusted to 10 by addition of conc. NH_4OH (80 mL). The resulting precipitate was collected by filtration, washed with ice-cold H_2O (3 × 20 mL) and dried *in vacuo*. **S9b** was obtained as a yellow solid (2.50 g, 86%) and was used without further purification. **^{1}H NMR** (400 MHz, DMSO-d_6): δ 7.94 (dd, J = 9.1, 1.7 Hz, 2H), 6.71 (s, 2H), 6.59 (d, J = 9.1 Hz, 2H) ppm; **^{13}C NMR** (101 MHz, DMSO-d_6): δ 155.7, 135.6 (d, J_{CN} = 16 Hz), 126.4 (d, J_{CN} = 2 Hz), 112.4 (d, J_{CN} = 3 Hz) ppm; **HRMS** (ESI+) = *m/z* calculated for $C_6H_7N[^{15}N]O_2^+$ $[M+H]^+$ 140.0472, found 140.0471. Spectroscopic data consistent with literature reported data for unlabeled analogue.[9]

2,6-Dibromo-4-(nitro-^{15}*N*)aniline (S9c)

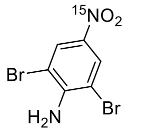

In a 150 mL round-bottom flask, **S9b** (2.41 g, 17.3 mmol) was suspended in AcOH (30 mL) and heated to 65 °C before a solution of Br_2 (1.95 mL, 38.0) in AcOH (15 mL) was added over 30 min. After 24 h (reaction monitoring *via* GC-MS), the reaction mixture was quenched onto ice (150 mL) and the resulting precipitate was collected by filtration, washed with ice-cold H_2O (3 × 25 mL) and dried *in vacuo*. **S9c** was obtained as a yellow solid (4.87 g, 95%) and was used without further purification. **^{1}H NMR** (400 MHz, DMSO-d_6): δ 8.28 (d, J = 1.8 Hz, 2H), 6.79 (s, 2H) ppm; **^{13}C NMR** (101 MHz, DMSO-d_6): δ 149.2, 136.4 (d, $J_{C–N}$ = 17 Hz), 128.0 (d, $J_{C–N}$ = 2 Hz), 105.3 (d, $J_{C–N}$ = 3 Hz) ppm; **HRMS** (ESI+) = *m/z* calculated for $C_6H_4Br_2N[^{15}N]O_2Na^+$ $[M+Na]^+$ 317.8502, found 317.9446. Spectroscopic data consistent with literature reported data for unlabeled analogue.[10]

1,3-Dibromo-5-(nitro-^{15}*N*)benzene (S9d)

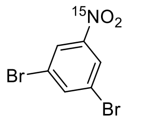

In a 150 mL round-bottom flask, **S9c** (4.76 g, 16.0 mmol) was suspended in a mixture of EtOH (50 mL) and conc. H_2SO_4 (6 mL). The yellow suspension was heated to 80 °C before $NaNO_2$ (3.32 g, 48.1 mmol) was added in portions. After 17 h (reaction monitoring *via* GC-MS), the reaction mixture was quenched onto ice (150 mL) and the resulting precipitate was collected by filtration, washed with ice-cold H_2O (3 × 25 mL) and dried *in vacuo*. The crude material was recrystallized from hot EtOH and **S9d** was obtained as a tan crystalline solid (2.92 g, 65%). **^{1}H NMR** (400 MHz, $CDCl_3$): δ 8.32 (t, J = 1.8 Hz, 2H), 8.00 (t, J = 1.7 Hz, 1H) ppm; **^{13}C NMR** (101 MHz, $CDCl_3$): δ 149.2 (d, $J_{C–N}$ = 16 Hz), 140.2, 125.7 (d, $J_{C–N}$ = 2 Hz), 123.6 (d, $J_{C–N}$ = 3 Hz) ppm; **HRMS** (ESI+) = *m/z* calculated for $C_6H_5Br_2$

$[^{15}N]O_2^+$ $[M+H]^+$ 280.8574, found 280.8900. Spectroscopic data consistent with literature reported data for unlabeled analogue.[11]

3,5-Dibromoaniline-^{15}*N* (S9e)

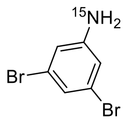

In a 250 mL round-bottom flask, **S9d** (2.84 g, 10.1 mmol) and $SnCl_2·2H_2O$ (11.38 g, 50.5 mmol) were suspended in a mixture of MeOH (35 mL) and THF (10 mL) and heated to 60 ˚C for 20 h (reaction monitoring *via* GC-MS). The reaction mixture was concentrated under reduced pressure, followed by the addition of NaOH (9.83 g) dissolved in H_2O (120 mL). The resulting suspension was stirred at r.t. for 2 h, extracted with CH_2Cl_2 (3 × 50 mL) and the combined organic layers were dried over Na_2SO_4, filtered and concentrated under reduced pressure. **S9e** was obtained as a beige solid (2.49 g, 98%) and was used without further purification. **^{1}H NMR** (400 MHz, CD_3CN): δ 6.92 (t, J = 1.7 Hz, 1H), 6.77 (t, J = 1.8 Hz, 2H), 4.49 (d, J_{HN} = 85.0 Hz, 2H) ppm; **^{13}C NMR** (101 MHz, CD_3CN): δ 151.8 (d, $J_{C–N}$ = 13 Hz), 123.8 (d, $J_{C–N}$ = 2 Hz), 122.4, 116.6 (d, $J_{C–N}$ = 3 Hz); **HRMS** (ESI+) = *m/z* calculated for $C_6H_56Br_2[^{15}N]^+$ $[M+H]^+$ 250.8832, found 250.8828. Spectroscopic data consistent with literature reported data for unlabeled analogue.[12]

3,3'',5,5''-Tetrakis(trifluoromethyl)-[1,1':3',1''-terphenyl]-5'-amine-^{15}*N* (S9f)

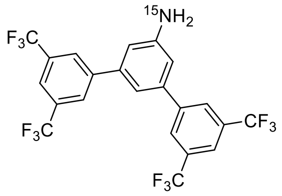

In a Schlenk flask, **S9e** (501 mg, 1.98 mmol), 3,5-bis(trifluoromethyl)phenylboronic acid (1.79 g, 6.95 mmol), SPhos (81.5 mg, 0.20 mmol), and K_2CO_3 (1.38 g, 9.92 mmol) were suspended in a THF/H_2O (25 mL, 4/1). The mixture was degassed (evacuate and backfill with N_2 three times) before $Pd(OAc)_2$ (22.3 mg, 0.10 mmol) was added and heated to 80 ˚C for 21 h (reaction monitoring *via* GC-MS). The reaction mixture was cooled to r.t. and the THF was removed under reduced pressure. The residue was taken up in H_2O (20 mL), extracted with CH_2Cl_2 (3 × 20 mL). The combined organic layers were dried over Na_2SO_4, filtered through a plug of Celite and concentrated under reduced pressure. The crude product was purified *via* flash column chromatography (0% to 10% (v/v) EtOAc in pentane) to give **S9f** as an off-white solid (864 mg, 84%). **^{1}H NMR** (400 MHz, CD_3CN): δ 8.29–8.23 (m, 3H), 8.00 (tt, J = 1.7, 0.9 Hz, 2H), 7.33 (t, J = 1.7 Hz, 1H), 7.09 (t, J = 1.7 Hz, 2H), 4.51 (d, $J_{H–N}$ = 82.8 Hz, 2H) ppm; **^{19}F NMR** (377 Hz, CD_3CN): δ -63.23 ppm; **^{13}C NMR** (101 MHz, CD_3CN): δ 150.6 (d, $J_{C–N}$ = 13 Hz), 144.3, 140.7, 132.5 (q, $J_{C–F}$ = 33 Hz), 128.6 (d,

J_{C-N} = 4 Hz), 124.7 (q, J_{C-F} = 272 Hz), 122.1 (sept, J_{C-F} = 4 Hz), 116.2, 114.6 (d, J_{C-N} = 3 Hz) ppm; **HRMS** (ESI+) = *m/z* calculated for $C_{22}H_{11}F_{12}[^{15}N]K^+$ $[M+K]^+$ 608.3419, found 608.3428. Spectroscopic data consistent with literature reported data for unlabeled analogue.[1]

5'-(Isocyanato-^{15}*N*)-3,3'',5,5''-tetrakis(trifluoromethyl)-1,1':3',1''-terphenyl (S9)

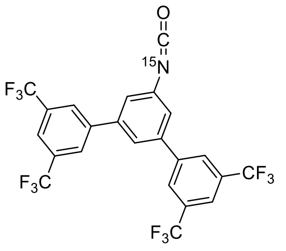

In a Schlenk flask, aniline **S9f** (122 mg, 0.24 mmol) was dissolved in anhydrous CH_2Cl_2 (1.5 mL) under an atmosphere of N_2. The solution was cooled to 0 °C, followed by addition of triphosgene (24 mg, 0.08 mmol), and dropwise addition of sat. aq. $NaHCO_3$ (1.5 mL), which resulted in the formation of a slurry. The reaction mixture was allowed vigorously stirred for 15 min. The organic phase was separated and extracted with CH_2Cl_2 (2 × 5 mL), washed with brine (5 mL), dried over $MgSO_4$, filtered over Celite and concentrated *in vacuo*. **S9** was obtained as an off-white solid (127 mg, 99%) and was used without further purification. **^{1}H NMR** (400 MHz, $CDCl_3$): δ 7.95 (s, 4H), 7.85 (s, 2H), 7.50 (s, 1H), 7.32 (s, 2H) ppm; **^{13}C NMR** (126 MHz, $CDCl_3$): δ 141.5, 141.2 (d, $^3J_{C-N}$ = 2 Hz), 135.5 (d, $^1J_{C-N}$ = 18 Hz), 132.6 (q, $^2J_{C-F}$ = 34 Hz), 127.5–127.4 (m), 125.3 (d, $^1J_{C-N}$ = 46 Hz), 123.2 (q, $^1J_{C-F}$ = 273 Hz), 123.9 (d, $^2J_{C-N}$ = 3 Hz), 123.6, 122.1–122.0 (m) ppm; **^{15}N NMR** (51 MHz, $CDCl_3$): δ 48.4 (s) ppm; **^{19}F NMR** (377 MHz, $CDCl_3$): δ -62.82 (s) ppm. Spectroscopic data consistent with literature reported data for unlabeled analogue.[4]

2.4. Characterization of catalysts

Boc-Leu-Pro-Acpc-Phe-NMe$_2$ (1a)

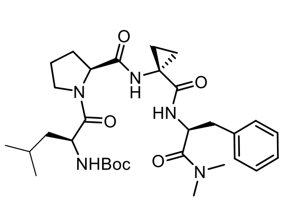

The title compound was prepared following *General Procedure I*, allowing the reaction to stir for 24 hours. The crude product was purified *via* flash column chromatography (5% then 10% (v/v) *i*PrOH in CH_2Cl_2), giving **1a** as a white powder (127 mg, 72%). **^{1}H NMR** (500 MHz, CD_2Cl_2): δ 7.87 (d, *J* = 7.8 Hz, 1H), 7.30–7.21 (m, 5H), 6.92 (s, 1H), 5.80 (d, *J* = 9.0 Hz, 1H), 4.97 (td, *J* = 8.4, 5.3 Hz, 1H), 4.45 (br td, *J* = 10.3, 2.3 Hz, 1H), 4.22 (t, *J* = 7.1 Hz, 1H), 3.90–3.76 (m, 1H), 3.62 (dt, *J* = 9.6, 7.1 Hz), 3.05 (dd, *J* = 13.2, 8.7 Hz, 1H), 2.90 (s, 3H), 2.89 (dd, *J* = 13.2, 5.5 Hz, 1H), 2.79 (s, 3H), 2.20–2.12 (m, 1H), 2.11 (q, *J* = 6.1 Hz, 2H), 2.03–1.96 (m, 1H), 1.86–1.78 (m, 1H), 1.63 (ddd, J = 13.4, 11.4, 3.9 Hz, 1H), 1.52 (ddd, *J* = 10.2, 7.7, 4.4 Hz, 1H), 1.49–1.46

(m, 1H), 1.44 (s), 1.42 (ddd, J = 10.2, 7.5, 4.1 Hz, 1H), 1.04 (ddd, J = 11.5, 7.5, 4.4 Hz, 1H), 1.02 (d, J = 6.5 Hz, 3H), 0.97 (d, J = 6.6 Hz, 3H), 0.93 (ddd, J = 11.5, 7.7, 4.1 Hz, 1H) ppm; **^{13}C NMR** (126 MHz, CD_2Cl_2): δ 173.3, 172.8, 170.6, 155.8, 137.8, 129.5, 128.1, 126.4, 79.0, 61.4, 51.5, 50.8, 47.4, 41.1, 38.7, 36.7, 35.5, 34.5, 28.2, 28.1, 25.6, 24.7, 23.2, 22.1, 17.3, 16.3 ppm; **HRMS** (ESI+) = *m/z* calculated for $C_{31}H_{47}N_5O_6Na^+$ $[M+Na]^+$ 608.3419, found 608.3428; **IR** (thin layer film): v 3305, 3296, 3013, 2962, 1702, 1638, 1525, 1453, 1392, 1367, 1046, 1027, 760, 702 cm^{-1}; $[\boldsymbol{\alpha}]_{\mathbf{D}}^{\mathbf{20℃}}$ = -38.5 (*c* 0.9, $CHCl_3$); **m.p.** 98 ˚C.

3,5-$(CF_3)_2C_6H_3$NHC(O)-Pro-Acpc-Phe-NMe_2 (1b)

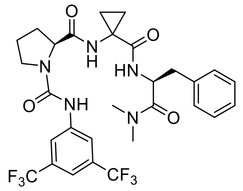

The title compound was prepared following *General Procedure IIIa.* The crude product was purified *via* flash column chromatography (3/1 to 2/3 (v/v) toluene/acetone), giving **1b** as a white solid (119 mg, 78%). **^{1}H NMR** (500 MHz, CD_3OD): δ 8.05 (s, 2H), 7.30 (s, 1H), 7.18–7.10 (m, 3H), 6.94–6.86 (m, 2H), 4.74 (dd, J = 8.2, 6.5 Hz, 1H), 4.35 (dd, J = 7.6, 6.4 Hz, 1H), 3.76–3.55 (m, 2H), 2.80 (s, 3H), 2.79–2.68 (m, 2H), 2.63 (s, 3H), 2.28–2.12 (m, 2H), 2.08–1.90 (m, 2H), 1.53–1.38 (m, 1H), 1.37–1.25 (m, 1H), 1.06–0.97 (m, 2H) ppm; **^{13}C NMR** (126 MHz, CD_3OD): δ 175.8, 172.2, 172.0, 154.6, 141.6, 136.6, 131.3 (q, J = 33 Hz), 128.7, 127.8, 126.3, 123.4 (q, J = 272 Hz), 120.0 (br), 114.9 (br), 61.0, 51.9, 46.5, 37.7, 35.9, 34.6, 33.9, 29.2, 24.9, 16.2, 15.7 ppm; **^{19}F NMR** (471 MHz, CD_3OD): δ −64.40 ppm; **HRMS** (ESI−) = *m/z* calculated for $C_{29}H_{31}F_6N_5O_4^-$ $[M-H]^-$ 626.2207, found 626.2199; **IR** (thin layer film): v 3247, 2975, 2461, 2377, 1651, 1633, 1538, 1472, 1441, 1393, 1345, 1322, 1299, 1278, 1217, 1178, 1132, 1106, 1003, 935, 883, 757, 730, 702, 682, 626 cm^{-1}; $[\boldsymbol{\alpha}]_{\mathbf{D}}^{\mathbf{25℃}}$ = −14.0 (*c* 0.7, MeOH); **m.p.** 273 °C.

3,5-[3,5-$(CF_3)_2C_6H_3]_2C_6H_3$NHC(O)-Pro-Acpc-Phe-NMe_2 (1c)

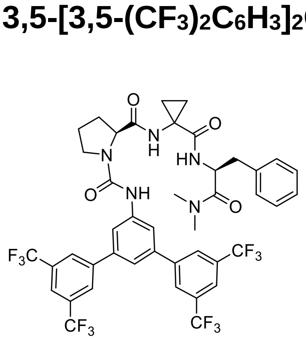

The title compound was prepared following *General Procedure IIIa.* The crude product was purified *via* flash column chromatography (9/1 to 1/1 (v/v) toluene/acetone), giving **1c** as a white solid (180 mg, 99%). **^{1}H NMR** (500 MHz, $(CD_3)_2SO$): δ 8.97 (s, 1H), 8.67 (s, 1H), 8.26 (s, 4H), 8.09 (s, 2H), 8.05 (d, J = 1.3 Hz, 2H), 7.90 (d, J = 7.4 Hz, 1H), 7.69 (t, J = 1.5 Hz, 1H), 7.00 (tt, J = 7.2, 1.2 Hz, 1H), 6.94 (br t, J = 7.3 Hz, 2H), 6.61 (br d, J = 7.1 Hz, 2H), 4.58 (ddd,

J = 9.1, 7.5, 6.0 Hz, 1H), 4.27 (br t, *J* = 6.7 Hz, 1H), 3.68 (td, *J* = 9.2, 6.6 Hz, 1H), 3.59–3.54 (m, 1H), 2.53 (dd, *J* = 12.9, 9.2 Hz, 1H), 2.49 (s, 3H), 2.42 (s, 3H), 2.53 (dd, *J* = 12.9, 6.0 Hz, 1H), 2.12–2.05 (m, 2H), 1.95–1.89 (m, 1H), 1.85–1.78 (m, 1H), 1.09 (ddd, *J* = 9.8, 7.2, 3.4 Hz, 1H), 1.28 (ddd, *J* = 10.0, 7.2, 3.8 Hz, 1H), 0.86 (ddd, *J* = 10.0, 6.9, 3.4 Hz, 1H), 0.80 (ddd, *J* = 9.8, 6.9, 3.8 Hz, 1H) ppm; **^{19}F NMR** (470 MHz, $(CD_3)_2SO$): δ -61.00 ppm; **^{13}C NMR** (151 MHz, $(CD_3)_2SO$): δ 173.7, 170.5, 170.2, 154.2, 142.6, 141.6, 138.3, 137.0, 130.8 (q, $^2J_{CF}$ = 33 Hz), 128.6, 127.7 (br), 127.5, 126.0, 123.4 (q, $^1J_{CF}$ = 273 Hz), 121.1 (t, $^3J_{CF}$ = 3 Hz), 120.3, 119.2, 60.3, 50.5, 46.6, 37.7, 36.0, 34.6, 33.5, 29.1 (br), 24.7 (br), 16.1, 15.6 ppm; **HRMS** (ESI+) = *m/z* calculated for $C_{43}H_{37}F_{12}N_5O_4Na^+$ $[M+Na]^+$ 938.2546, found 938.2524; **IR** (thin layer film): *v* 3272, 2926, 2856, 1719, 1663, 1641, 1536, 1372, 1281, 1180, 1139, 900, 846, 706 cm^{-1}; $[\alpha]_D^{20°C}$ = –45.9 (*c* 0.9, acetone); **m.p.** 241−243 ˚C.

3,5-[3,5-$(CF_3)_2C_6H_3]_2C_6H_3$NHC(O)-Pro-Gly-Phe-NMe$_2$ (1c-Gly(*i*+1))

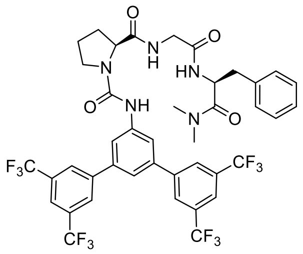

The title compound was prepared following *General Procedure IIIa*. The crude product was purified *via* flash column chromatography (5/1 to 1/5 (v/v) toluene/acetone), giving **1c-Gly** as a white solid (459 mg, 79%). **^{1}H NMR** (400 MHz, $CDCl_3$): δ 7.93 (s, 4H), 7.84 (s, 2H), 7.72 (d, *J* = 1.6 Hz, 2H), 7.51 (t, *J* = 6.1 Hz, 1H), 7.45–7.34 (m, 1H), 7.30 (d, *J* = 7.4 Hz, 1H), 7.25 (t, *J* = 1.6 Hz, 1H), 7.20–7.10 (m, 3H), 6.99–6.88 (m, 2H), 4.92 (td, *J* = 7.9, 5.8 Hz, 1H), 4.67–4.59 (m, 1H), 4.09 (dd, *J* = 17.0, 6.8 Hz, 1H), 3.79–3.68 (m, 2H), 3.59 (q, *J* = 7.8 Hz, 1H), 2.88–2.73 (m, 2H), 2.70 (s, 3H), 2.50 (s, 3H), 2.43–2.31 (m, 1H), 2.22–2.01 (m, 3H); **^{13}C NMR** (126 MHz, $CDCl_3$): δ 173.1, 171.3, 168.3, 155.5, 142.6, 140.9, 139.9, 136.0, 132.3 (q, *J* = 33 Hz), 129.4, 128.5, 127.5–127.3 (m), 127.2, 123.4 (q, *J* = 273 Hz), 121.7–121.5 (m), 120.8, 119.7, 60.7, 50.7, 47.2, 42.9, 39.2, 36.8, 35.6, 29.0, 25.1 ppm; **^{19}F NMR** (377 MHz, $CDCl_3$): δ –62.72 ppm; **HRMS** (ESI+) = *m*/*z* calculated for $C_{41}H_{35}F_{12}N_5O_4Na^+$ $[M+Na]^+$ 912.2390, found 912.2410; **IR** (thin layer film): *v* 3291, 2932, 1727, 1642, 1605, 1535, 1374, 1283, 1178, 1141, 924, 684, 665 cm^{-1}; **m.p.** 126–128 °C; $[\alpha]_D^{20°C}$ = –24.8 (*c* 0.9, $CHCl_3$).

3,5-[3,5-$(CF_3)_2C_6H_3]_2C_6H_3$NHC(O)-D-Pro-Gly-Phe-NMe_2 (1c-D-Pro(*i*)-Gly(*i*+1))

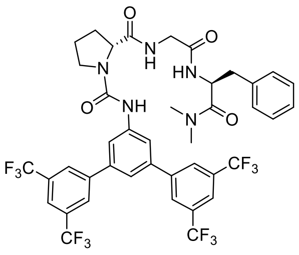

The title compound was prepared following *General Procedure IIIa.* The crude product was purified *via* flash column chromatography (3/2 to 2/3 (v/v) toluene/acetone), giving **1c-Gly-D-Pro** as a white solid (532 mg, 78%). **^{1}H NMR** (400 MHz, $CDCl_3$): δ 7.95 (s, 4H), 7.80 (br s, 4H), 7.64 (br t, J = 5.1 Hz. 1H), 7.51 (s, 1H), 7.37 (d, J = 6.7 Hz, 1H), 7.25 (t, J = 1.5 Hz, 1H), 7.13–7.07 (m, 3H), 6.92 (dd, J = 7.3, 1.4 Hz, 2H), 4.83 (ddd**,** J = 9.2, 6.9, 5.6 Hz, 1H), 4.63 (dd, J = 7.1, 3.6 Hz, 1H), 3.97–3.91 (m, 2H), 3.88–3.81 (m, 1H), 3.73 (q, J = 7.8 Hz, 1H), 2.85 (dd, J = 13.0, 5.6 Hz, 1H), 2.68 (dd, J = 13.0, 9.2 Hz, 1H), 2.65 (s, 3H), 2.37 (s, 3H), 2.33–2.06 (m, 4H) ppm; **^{19}F NMR** (470 MHz, $CDCl_3$): δ –62.75 ppm; **^{13}C NMR** (101 MHz, $CDCl_3$): δ 172.5, 171.0, 168.3, 155.4, 142.5, 140.8, 139.8, 135.9, 132.1 (q, J_{CF} = 33 Hz), 129.1, 128.2, 127.3 (br s), 126.9, 123.0 (q, J_{CF} = 273 Hz), 121.4 (br s), 120.6, 118.5, 61.2, 50.7, 47.0, 42.5, 39.2, 36.6, 35.4, 29.1, 25.1 ppm; **HRMS** (ESI+) = *m*/*z* calculated for $C_{41}H_{36}F_{12}N_5O_4^+$ $[M+H]^+$ 890.2570, found 890.2488; **IR** (thin layer film): *v* 3307, 1646, 1547, 1475, 1443, 1359, 1279, 1177, 1130, 883, 702, 682, 628 cm^{-1}; $[\boldsymbol{\alpha}]_{\mathbf{D}}^{25℃}$ = 93.7 (*c* 1.0, MeOH); **m.p.** 143–145 °C.

3,5-[3,5-$(CF_3)_2C_6H_3]_2C_6H_3$NHC(O)-flp-Gly-Phe-NMe_2 (1d-Gly(*i*+1))

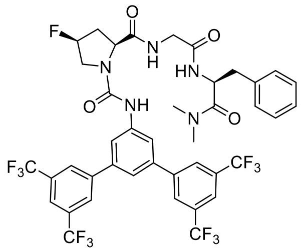

The title compound was prepared following *General Procedure IIIa*. The crude product was purified *via* flash column chromatography (4/1 to 2/3 (v/v) toluene/acetone), giving **1d-Gly(*i*+1)** as a white solid (799 mg, 89%). **^{1}H NMR** (400 MHz, CD_2Cl_2): δ 8.07 (s, 1H), 8.04 (s, 4H), 7.91 (s, 2H), 7.89 (d, J = 1.5 Hz, 2H), 7.51 (t, J = 5.5 Hz, 1H), 7.40 (t, J = 1.5 Hz, 1H), 7.31 (d, J = 7.9 Hz, 1H), 7.23–7.20 (m, 3H), 7.06–7.04 (m, 2H), 5.42 (dt, J_{HF} = 51.8 Hz, J_{HH} = 3.2 Hz, 1H), 4.99 (q, J = 7.6 Hz, 1H), 4.78 (d, J = 10.0 Hz, 1H), 4.07 (br dd, J_{HF} = 23.1 Hz, J_{HH} = 12.4 Hz, 1H), 3.99–3.86 (m, 3H), 2.89 (dd, J = 13.4, 7.6 Hz, 1H), 2.82 (dd, J = 13.4, 6.4 Hz, 1H), 2.77 (br t, J_{HF} = 16.0 Hz, J_{HH} = 16.0 Hz, 1H), 2.74 (s, 3H), 2.59 (s, 3H), 2.52 (dddd, J_{HF} = 42.9 Hz, J_{HH} = 14.8, 10.3, 3.7 Hz, 1H) ppm; **^{13}C NMR** (126 MHz, CD_2Cl_2): δ 172.2, 170.9, 167.9, 155.4, 142.5, 140.9, 139.6, 136.1, 132.0 (q, J = 33 Hz), 129.3, 128.2, 127.3 (br s), 127.9, 123.4 (q, J = 273 Hz), 121.4 (br s), 120.5, 119.5, 92.4 (d, J = 118 Hz), 60.2, 50.4, 47.6, 38.7, 36.6, 36.5, 35.2, 29.7 ppm; **^{19}F NMR** (470

MHz, CD_2Cl_2): δ −63.11 (12F), −172.93 (1F) ppm; **HRMS** (ESI+) = *m/z* calculated for $C_{41}H_{34}F_{13}N_5O_4Na^+$ $[M+Na]^+$ 930.2295, found 930.2339; **IR** (thin layer film): v 3315, 2928, 1656, 1561, 1371, 1232, 1178, 1137, 757, 870, 845, 683 cm^{-1}; **m.p.** 152 °C; $[\alpha]_D^{20℃}$ = −23.7 (*c* 0.9, $CHCl_3$).

3,5-[3,5-(CF3)2C6H3]2C6H3NHC(O)-Flp-Gly-Phe-NMe2 (1d-Flp(*i*)-Gly(*i*+1))

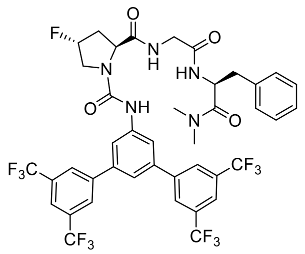

Prepared following *General Procedure IIIa*. The crude product was purified *via* flash column chromatography (5-10% *i*PrOH in toluene), giving **1d-Flp(*i*)-Gly(*i*+1)** as a white solid (241 mg, 90%). **^{1}H NMR** (400 MHz, CD_2Cl_2): δ 8.00 (s, 4H), 7.89 (s, 2H), 7.78 (br t, J = 5.9 Hz, 1H) 7.75 (br s, 1H), 7.72 (s, 2H), 7.42 (d, J = 6.1 Hz, 1H), 7.27 (s, 1H), 7.15–7.10 (m, 3H), 6.85 (d, J = 7.1 Hz, 2H), 5.36 (br d, J = 52.5 Hz, 1H), 4.87–4.80 (m, 2H), 4.12 (dd, J = 17.6, 6.8 Hz, 1H), 4.02 (dd, J = 22.3, 12.0 Hz, 1H), 3.86 (br ddd, J = 35.1, 12.0, 2.3 Hz, 1H), 3.64 (dd, J = 17.4, 5.0 Hz, 1H), 2.73 (d, J = 6.6 Hz, 2H), 2.67 (s, 3H), 2.54 (s, 3H), 2.62–2.45 (m, 2H) ppm; **^{13}C NMR** (101 MHz, CD_2Cl_2): δ 172.9, 171.3, 168.2, 155.2, 142.4, 140.7, 139.4, 136.1, 131.9 (q, J = 33 Hz), 129.1, 128.0, 127.3 (br s), 126.8, 123.4 (q, J = 273 Hz), 121.3 (br s), 120.5, 119.9, 92.4 (d, J = 178 Hz), 59.2, 53.6, 51.0, 43.1, 38.3, 36.4, 35.8 (d, J = 22 Hz), 35.2 ppm; **^{19}F NMR** (376 MHz, CD_2Cl_2): δ −63.06 (s, 12F), −176.54 (br s, 1F) ppm; **HRMS** (ESI+) = *m/z* calculated for $C_{41}H_{34}F_{13}N_5O_4Na^+$ $[M+Na]^+$ 930.2295, found 930.2265; **m.p.** 133 °C; $[\alpha]_D^{20℃}$ = −15.8 (*c* 0.9, $CHCl_3$).

3,5-[3,5-(CF3)2C6H3]2C6H3NHC(O)-flp-Acpc-Phe-NMe2 (1d)

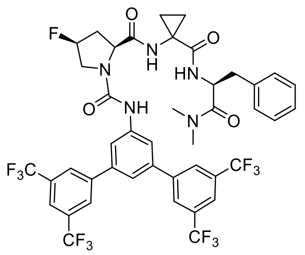

The title compound was prepared following *General Procedure IIIb.* The crude product was purified *via* flash column chromatography (4/1 to 1/1 (v/v) toluene/acetone), giving **1d** as a white solid (98 mg, 65%). **^{1}H NMR** (600 MHz, $(CD_3)_2SO$): δ 8.77 (s, 1H), 8.72 (s, 1H), 8.29 (s, 4H), 8.10 (d, J = 1.6 Hz, 2H), 8.08 (s, 2H), 7.76 (t, J = 1.1 Hz, 1H), 7.71 (d, J = 7.7 Hz, 1H), 7.04–6.97 (m, 3H), 6.73 (br d, J = 7.1 Hz, 2H), 5.42 (br dt, $^2J_{HF}$ = 53.8 Hz, J = 3.9 Hz, 1H), 4.63 (dt, J = 8.1, 6.5 Hz, 1H), 4.49 (br d, J = 7.9 Hz, 1H), 3.95 (br dd, $^3J_{HF}$ = 24.1 Hz, J = 12.1 Hz, 1H), 3.84 (ddd, $^3J_{HF}$ = 32.5 Hz, J = 12.1, 4.1 Hz, 1H), 2.62 (dd, J = 13.1, 8.8 Hz, 1H), 2.63–2.54 (m, 1H), 2.59 (s, 3H), 2.54 (s, 3H), 2.45 (dd, J = 13.1, 6.4 Hz, 1H), 2.20 (br t, $^3J_{HF}$ = 18.0 Hz, 1H), 1.22 (ddd, J = 10.1, 7.4, 3.9 Hz, 1H), 1.14 (ddd, J = 10.1, 7.5, 3.9 Hz,

1H), 0.87 (ddd, J = 9.8, 7.5, 3.9 Hz, 1H), 0.79 (ddd, J = 9.8, 7.5, 3.9 Hz, 1H) ppm; **^{19}F NMR** (377 MHz, $CDCl_3$): δ –62.70, –172.96 (br s) ppm; **^{13}C NMR** (151 MHz, $(CD_3)_2SO$): δ 173.0, 171.2, 170.7, 154.8, 143.1, 142.2, 138.8, 137.4, 131.3 (q, $^2J_{CF}$ = 33 Hz), 129.2, 128.1, 126.5, 123.8 (q, $^1J_{CF}$ = 271 Hz), 121.7 (br), 120.8, 119.4, 92.8 (d, $^1J_{CF}$ = 175 Hz), 59.6, 53.3 (d, $^2J_{CF}$ = 24 Hz), 51.0, 38.3, 36.6 (*two peaks overlapping; based on HSQC*), 35.3, 34.1, 16.6, 16.5 ppm; **HRMS** (ESI+) = *m/z* calculated for $C_{43}H_{37}F_{13}N_5O_4^+$ $[M+H]^+$ 934.2632, found 934.2674; **IR** (thin layer film): v 3269, 3065, 2938, 1715, 1642, 1608, 1430, 1180, 1137, 900, 845, 706, 683 cm^{-1}; $[\alpha]_D^{20℃}$ = –32.3 (*c* 0.9, acetone); **m.p.** 241−242 ˚C.

3,5-[3,5-$(CF_3)_2C_6H_3]_2C_6H_3$^{15}NHC(O)-flp-Acpc-Phe-NEt_2 (1e-^{15}NH(*i*–1))

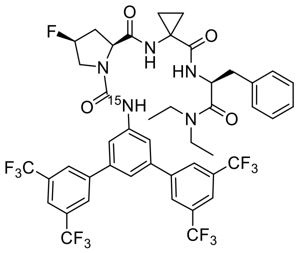

The title compound was prepared following *General Procedure IIIb.* The crude product was purified *via* flash column chromatography (9/1 to 3/2 (v/v) toluene/acetone) followed by (2% to 5% *i*PrOH in CH_2Cl_2), giving **1e-^{15}NH(*i*–1)** as a white solid (122 mg, 62%). **^{1}H NMR** (500 MHz, CD_2Cl_2): δ 8.10 (s, 4H), 7.95 (s, 2H), 7.92 (s, 2H), 7.65 (br d, $^1J_{NH}$ = 88.5 Hz, 1H), 7.48 (s, 1H), 7.32 (s, 1H), 7.20 (d, J = 8.2 Hz, 1H), 7.15–7.14 (m, 3H), 7.00–6.98 (m, 2H), 5.46 (dt, J = 52.4, 3.5 Hz, 1H), 4.87 (q, J = 7.9 Hz, 1H), 4.75 (d, J = 9.6 Hz, 1H), 4.16 (dd, J = 22.4, 11.6 Hz, 1H), 3.93 (ddd, J = 35.2, 11.8, 3.7 Hz, 1H), 3.44 (sx, J = 7.0 Hz, 1H), 3.00–2.91 (m, 3H), 2.82–2.76 (m, 2H), 2.71 (dd, J = 13.3, 6.5 Hz, 1H), 2.47 (dddd, J = 41.0, 14.7, 10.0, 3.8 Hz, 1H), 1.49–1.40 (m, 2H), 1.09 (ddd, J = 10.2, 7.2, 4.1 Hz, 1H), 0.98 (ddd, J = 10.2, 6.5, 3.2 Hz, 1H), 0.91 (appt q, J = 7.3 Hz, 6H) ppm; **^{13}C NMR** (151 MHz, CD_2Cl_2): δ 172.3, 170.6, 170.3, 155.2, 142.6 (d, J_{CN} = 21 Hz), 140.6 (d, J_{CN} = 17 Hz), 139.9, 136.5, 132.0 (q, J_{CF} = 33 Hz), 129.3, 128.1, 127.5 (br d, J_{CF} = 3 Hz), 126.6, 122.3 (q, J_{CF} = 273 Hz), 121.5 (br t, J = 4 Hz), 121.1, 119.6, 92.4 (d, J_{CF} = 173 Hz), 59.9, 53.9 (*overlapping with solvent peak*), 50.6, 41.5, 40.3, 39.6, 35.6 (br d, J_{CF} = 2 Hz), 34.6, 16.5, 16.3, 13.6, 12.3 ppm; **^{19}F{^{1}H} NMR** (470 MHz, $CDCl_3$): δ –62.69, –172.74 ppm; **^{15}N NMR** (120 MHz, CD_2Cl_2): δ 105.5 (d, $J_{N–H}$ = 89 Hz) ppm; **HRMS** (ESI+) = *m/z* calculated for $C_{45}H_{40}F_{13}N_4[^{15}N]O_4Na^+$ $[M+Na]^+$ 985.2735, found 985.2737; **IR** (powder): v 3290, 2926, 2855, 1662, 1636, 1540, 1462, 1432, 1367, 1280, 1177, 1135, 843, 703, 634 cm$^{–1}$; $[\alpha]_D^{20℃}$ = –32.1 (*c* 0.9, acetone); **m.p.** 152 °C.

3,5-[3,5-$(CF_3)_2C_6H_3$]$_2C_6H_3$NHC(O)-flp-Acpc-[^{15}N]Phe-NEt_2 (1e-^{15}NH(*i*+2))

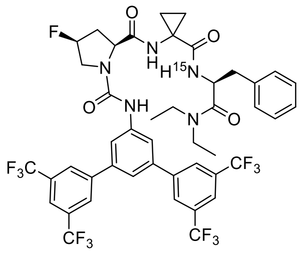

The title compound was prepared following *General Procedure IIIb.* The crude product was purified *via* flash column chromatography (9/1 to 3/2 (v/v) toluene/acetone), giving **1e-^{15}NH(*i*+2)** as a white solid (253 mg, 60%). **^{1}H NMR** (500 MHz, CD_2Cl_2): δ 8.10 (s, 4H), 7.95 (s, 2H), 7.92 (s, 2H), 7.65 (br s, 1H), 7.48 (s, 1H), 7.32 (s, 1H), 7.20 (dd, $^1J_{NH}$ = 92.8 Hz, $^1J_{HH}$ = 8.2 Hz, 1H), 7.15–7.14 (m, 3H), 7.00–6.98 (m, 2H), 5.46 (dt, J = 52.4, 3.5 Hz, 1H), 4.87 (q, J = 7.9 Hz, 1H), 4.75 (d, J = 9.6 Hz, 1H), 4.16 (dd, J = 22.4, 11.6 Hz, 1H), 3.93 (ddd, J = 35.2, 11.8, 3.7 Hz, 1H), 3.44 (sx, J = 7.0 Hz, 1H), 3.00–2.91 (m, 3H), 2.82–2.76 (m, 2H), 2.71 (dd, J = 13.3, 6.5 Hz, 1H), 2.47 (dddd, J = 41.0, 14.7, 10.0, 3.8 Hz, 1H), 1.49–1.40 (m, 2H), 1.09 (ddd, J = 10.2, 7.2, 4.1 Hz, 1H), 0.98 (ddd, J = 10.2, 6.5, 3.2 Hz, 1H), 0.91 (appt q, J = 7.3 Hz, 6H) ppm; **^{13}C NMR** (151 MHz, CD_2Cl_2): δ 172.3, 170.6, 170.3 (d, J_{CN} = 17 Hz,), 155.2, 142.6, 140.6, 139.9, 136.5, 132.0 (q, J_{CF} = 33 Hz), 129.3, 128.1 127.5 (br d, J_{CF} = 3 Hz), 126.6, 122.3 (q, J_{CF} = 273 Hz), 121.5 (br t, J_{CF} = 4 Hz), 121.1, 119.6, 92.4 (d, J_{CF} = 173 Hz), 59.9, 53.9 (*overlapping with solvent peak*), 50.6 (d, J_{CN} = 12 Hz), 41.5, 40.3, 39.6, 35.6 (br d, J_{CF} = 2 Hz), 34.6 (d, J_{CN} = 9 Hz), 16.5, 16.3, 13.6, 12.3 ppm; **^{19}F{^{1}H} NMR** (470 MHz, $CDCl_3$): δ –62.70, –172.74 ppm; **^{15}N NMR** (120 MHz, CD_2Cl_2): δ 115.9 (d, $J_{N\text{–}H}$ = 92 Hz) ppm; **HRMS** (ESI+) = *m/z* calculated for $C_{45}H_{40}F_{13}N_4[^{15}N]O_4Na^+$ $[M+Na]^+$ 985.2735, found 985.2722; **IR** (powder): ν 3243, 3062, 2973, 2937, 1658, 1639, 1546, 1511, 1464, 1432, 1366, 1280, 1177, 1135, 870, 704, 683 cm^{-1}; $[\alpha]_D^{20°C}$ = –31.6 (*c* 0.9, acetone); **m.p.** 154 °C.

3. Optimization of reaction conditions

3.1. General reaction conditions for screenings

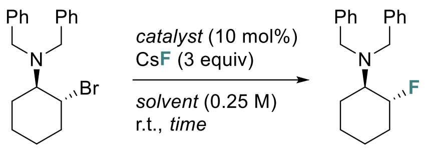

rac-**2a** *S,S*-**3a**

A 1.75 mL glass vial equipped with a stirring bar was charged with *rac*-*N*,*N*-dibenzyl-2-bromocyclohexan-1-amine (*rac*-**2a**)[1] (1.0 equiv., 0.050 mmol), catalyst (0.1 equiv., 0.005 mmol), CsF (3.0 equiv., 0.150 mmol) and anhydrous solvent (0.25 M, 200 µL). The reaction mixture was stirred at 900 rpm for the indicated period of time at r.t. The crude mixture was filtered through a small plug of Celite, which was washed with CH_2Cl_2. The filtrate was evaporated to dryness, dissolved in $CDCl_3$ (0.7 mL) and analyzed by ^{1}H and ^{19}F NMR (400 MHz and 377 MHz respectively) using 4-fluoroanisole as internal standard (5.0 µL, 0.044 mmol) to determine the yield. ^{19}F *q*NMR parameters were set-up as o1p = –150 ppm, d_1 = 35 s. To determine the *e.r.* (enantiomeric ratio) an aliquot of the reaction mixture was purified by preparative TLC (500 micron; 99.8:0.2 hexane:Et_2O) and analyzed by chiral analytical HPLC: DAICEL CHIRALPAK® IB-3, Heptane:*i*PrOH = 99.5:0.5, 1 mL/min; t_1 = 3.92 min (major), t_2 = 5.44 min (minor). The results presented in this section are averages of two experiments.

3.2. Reaction screenings

Table S2. Catalyst screening.

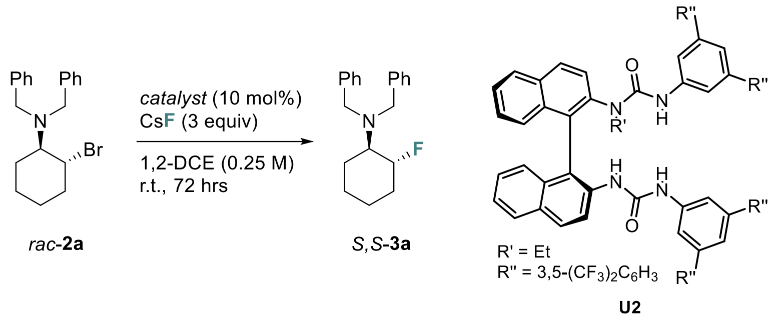

Catalyst	NMR Yield (%)	Cat. Deg. (%)	***e.r.*** (*S*,*S*):(*R*,*R*)
-	<1	n/a	n/a
**1a**	18	0	50:50
**1b**	22	11	66:34
**1c**	18	0	71:29
**1d**	84	13	85:15
**1e**	86	20	88:12
**1c-Gly(*i*+1)**	76	5	70:30
**1c-D-Pro(*i*)-Gly(*i*+1)**	42	15	46:54
**1d-Gly(*i*+1)**	86	24	80:20
**1d-Flp(*i*)-Gly(*i*+1)**	73	34	59:41
**U2**	*quantitative*	0	20:80

Table S3. Reaction with TBAF$(H_2O)_3$.

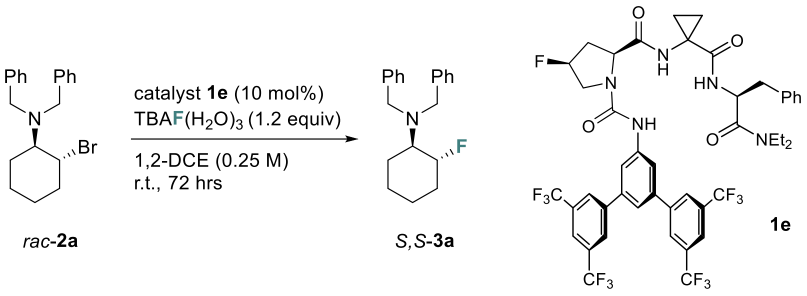

Catalyst loading (**1e**)	Yield, *e.r.*[a]	Alcohol	RSM[b]
-	22%, 50:50	36%	42%
10 mol%	20%, 50:50	34%	46%

[a]Determined by chiral-HPLC, [b]RSM = remaining starting material.

Table S4. Solvent screening.

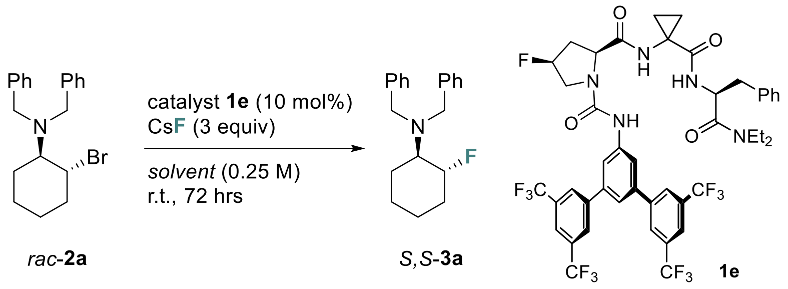

Solvent	NMR Yield (%)	Cat. Deg. (%)	*e.r.* (*S*,*S*):(*R*,*R*)
α,α,α-trifluorotoluene	24	17	83:17
1,2-DCB	28	*trace*	85:15
$CHCl_3$[a]	*quant.*	0	51:49
1,2-DFB	34	8	85:15
CH_2Cl_2	98	32	81:19
1,2-DCE	81	20	88:12
1,2-DCE ("wet")[b]	86	24	88:12
MeCN	42	13	87:13
THF	*trace*	0	n.d.
EtOAc	8	0	n.d.
toluene	4	0	n.d.
acetone	28	0	90:10

[a]$CHCl_3$ filtered over basic alumina to remove residual HCl; [b]1,2-DCE was saturated with water.

Table S5. Temperature screening.

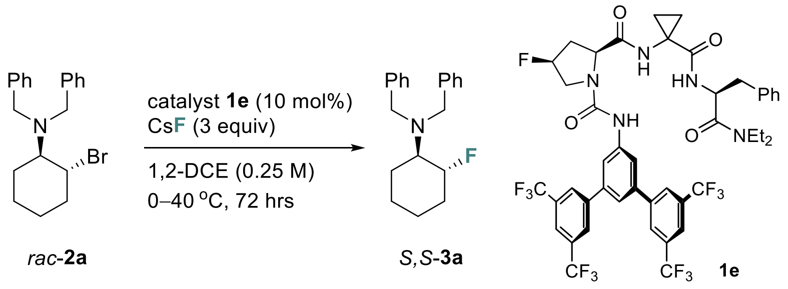

Temperature	NMR Yield (%)	Cat. Deg. (%)	*e.r.* (*S*,*S*):(*R*,*R*)
0 °C	<1	0	n.d.
6 °C	16	0	90:10
r.t.	88	24	88:12
r.t.[a]	<1	n.a.	n.d.
40 °C	>99	37	75:25
40 °C[a]	60	n.a.	n.d.

[a]No catalyst added; n.a. = not applicable; n.d. = not determined.

Table S6. Time course at 6 °C.

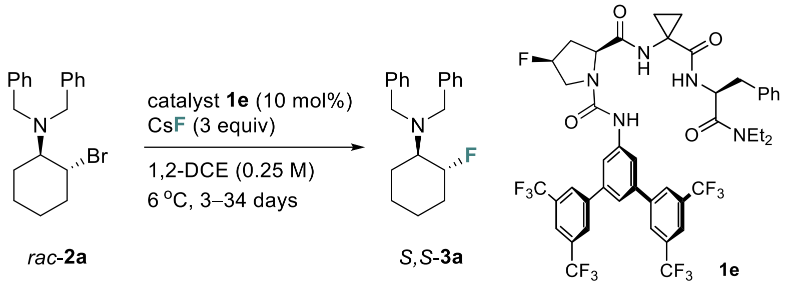

Time	NMR Yield (%)	Cat. Deg. (%)	*e.r.* (*S*,*S*):(*R*,*R*)
3 days	16	0	90:10
7 days	34	6	90:10
16 days	60	12	90:10
34 days	97	31	90:10

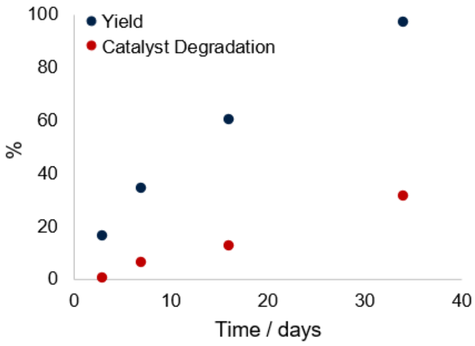

Table S7. Effect of catalyst loading and concentration.

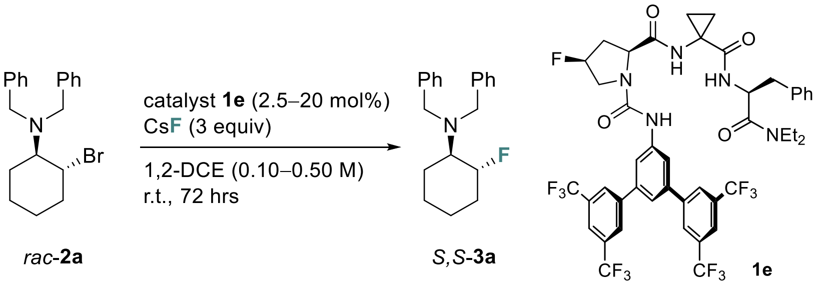

Catalyst loading (mol%)	Concentration (M)	NMR Yield (%)	Cat. Deg. (%)	*e.r.* (*S*,*S*):(*R*,*R*)
2.5	0.25	56	50	89:11
5	0.25	70	33	88:12
10	0.25	86	24	88:12
20	0.25	84	8	85:15
10	0.10	73	16	89:11
10	0.50	78	22	86:14
5[a]	0.25	76	37	88:12
5[b]	0.25	88	43	88:12

[a]96 hours; [b]120 hours.

4. Substrate synthesis and characterization

β-Bromoamines *trans-N*,*N*-dibenzyl-2-bromocyclohexan-1-amine (*rac*-**2a**), *trans-N,N*-dibenzyl-2-bromocyclopentan-1-amine (*rac*-**2b**)**,** 2-(*trans*-2-bromo-1,2-diphenylethyl)-1,2,3,4-tetrahydroisoquinoline (*rac*-**2f**), 1-(*trans*-2-bromo-1,2-diphenylethyl)piperidine (*rac*-**2g**) and *trans*-2-bromo-*N*,*N*-dimethyl-1,2-diphenylethan-1-amine (*rac*-**2h**), were prepared and characterized in the group previously.[1] *rac*-**2c−2e** are novel compounds and their preparation and full characterization is provided below.

4.1. Alcohols

General Procedure IV: Synthesis of β-Aminoalcohols:

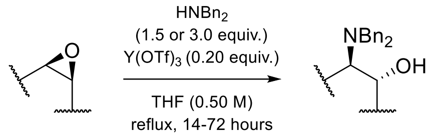

rac *rac*-**S10**-**12**

cis-Epoxide (1.0 mmol), the corresponding amine (3.0 mmol) and $Y(OTf)_3$ (0.20 mmol) were heated to reflux in anhydrous THF (2.0 mL) under N_2 atmosphere until complete conversion of the epoxide (14–72 h, monitored by TLC) or for 48 hours if epoxide is not visible on TLC (relevant to epoxides *cis*-3,6-dioxabicyclo[3.1.0]hexane and *cis*-8-oxabicyclo[5.1.0]octane). The reaction mixture was then cooled to r.t. and the solvent was evaporated *in vacuo*. The crude product was purified by flash column chromatography over silica gel.

***trans*-4-(Dibenzylamino)tetrahydrofuran-3-ol (*rac*-S10)**

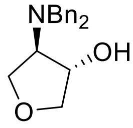

rac-**S10**

Alcohol *rac*-**S10** was prepared following *General Procedure IV* from *cis*-3,6-dioxabicyclo[3.1.0]hexane[13] (0.86 g, 10 mmol). Purification by flash column chromatography (100:0 to 90:10 (v/v) pentane/EtOAc + 1% NEt_3, then 3:2 (v/v) pentane/EtOAc) afforded the title compound as a yellow oil (2.19 g, 77% yield).

^{1}H NMR (400 MHz, $CDCl_3$): δ 7.40–7.25 (m, 10H), 4.53–4.50 (m, 1H), 4.03 (dd, J = 9.6, 6.0 Hz, 1H), 4.01 (dd, J = 10.0, 7.4 Hz, 1H), 3.83 (dd, J = 9.6, 5.3 Hz, 1H), 3.75 (d, J = 14.1 Hz, 2H), 3.67 (d, J = 14.1 Hz, 2H), 3.63 (dd, J = 10.0, 4.2 Hz, 1H), 1.77 (br s, 1H) ppm; **^{13}C NMR** (101 MHz, $CDCl_3$): δ 139.3, 128.6, 128.4, 127.1, 75.0, 73.8, 69.8, 69.4, 55.5 ppm; **HRMS** (ESI+) = m/z calculated for $C_{18}H_{22}NO_2^+$ $[M+H]^+$ 284.1645,

found 284.1634; **IR** (thin layer film): *v* 3413, 3028, 2917, 2842, 1493, 1452, 1076, 1028, 980, 917, 741, 698 cm^{-1}.

tert-Butyl trans-3-(dibenzylamino)-4-hydroxypyrrolidine-1-carboxylate (rac-S11)

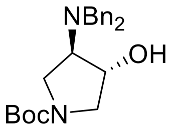

rac-**S11**

Alcohol *rac*-**S11** was prepared following *General Procedure IV* from *tert*-butyl *cis*-6-oxa-3-azabicyclo[3.1.0]hexane-3-carboxylate[14] (1.00 g, 5.4 mmol, 1 equiv.). Purification by flash column chromatography (80:20 (v/v) pentane/EtOAc) afforded colorless clear crystals (2.03 g, 97% yield). **^{1}H NMR** (400 MHz, $CDCl_3$): δ 7.37–7.23 (m, 10H), 4.33–4.28 (m, 1H), 3.82–3.49 (m, 7H), 3.37–3.23 (m, 2H), 3.09–3.00 (m, 1H), 1.45 (s, 9H) ppm; **^{13}C NMR** (101 MHz, $CDCl_3$, *mixture of rotamers*): δ 154.6 (major), 154.4 (minor), 139.4, 128.52 (major), 128.50 (br), 128.4 (minor), 127.3 (major), 127.2 (minor), 79.6, 70.9 (major), 70.3 (minor), 66.1 (minor), 65.4 (major), 55.1, 52.8 (minor), 51.0 (major), 44.6 (minor), 43.2 (major), 28.5 ppm; **HRMS** (ESI+) = *m/z* calculated for $C_{23}H_{31}N_2O_3^+$ [M+H]$^+$ 383.2329, found 383.2328. Spectroscopic data consistent with literature reported data.[15]

***trans*-2-(Dibenzylamino)cycloheptan-1-ol (*rac*-S12)**

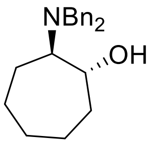

rac-**S12**

Alcohol *rac*-**S12** was prepared following *General Procedure IV* from *cis*-8-oxabicyclo[5.1.0]octane[16] (560 mg, 5.00 mmol, 1 equiv.). Purification by flash column chromatography (80:20 (v/v) pentane/EtOAc) afforded white clear crystals (0.80 g, 52% yield).

^{1}H NMR (400 MHz, $CDCl_3$): δ 7.35–7.23 (m, 10H), 4.08 (s, 1H), 3.84 (d, *J* = 13.1 Hz, 2H), 3.49 (ddd, *J* = 13.3, 8.6, 3.9 Hz, 1H), 3.33 (d, *J* = 13.1 Hz, 2H), 2.42 (td, *J* = 9.4, 2.7 Hz, 1H), 1.97–1.90 (m, 2H), 1.82–1.75 (m, 1H), 1.61–1.49 (m, 3H), 1.41–1.27 (m, 4H) ppm; **^{13}C NMR** (101 MHz, $CDCl_3$): δ 139.3, 129.2, 128.6, 127.4, 71.7, 65.6, 53.7, 33.1, 27.4, 24.9, 21.9, 21.6 ppm; **HRMS** (ESI+) = *m/z* calculated for $C_{21}H_{28}NO^+$ [M+H]$^+$ 310.2165, found 310.2163; **IR** (thin layer film): *v* 3458, 2930, 2859, 1494, 1454, 748, 699 cm^{-1}.

4.2. Bromides

General Procedure V: Synthesis of β-bromoamines:

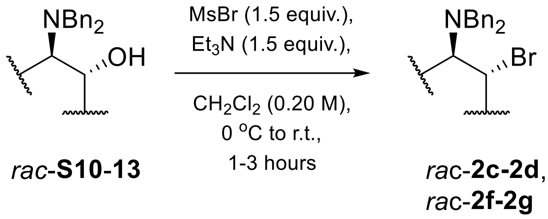

rac-**S10-13** → *rac*-**2c-2d**, *rac*-**2f-2g**

The corresponding β-aminoalcohol (1.0 mmol) was dissolved in anhydrous CH_2Cl_2 (0.20 M) under N_2 atmosphere, and the solution was cooled to 0 °C. NEt_3 (1.5 equiv.) was added at once, followed by dropwise addition of MsBr (1.5 equiv.) at 0 °C. The reaction mixture was allowed to warm to r.t. and stirred for 1–3 h. After disappearance of both the starting material and the mesylate intermediate (monitored by ^{1}H NMR), the reaction mixture was washed with sat. aq. $NaHCO_3$ (10 mL) and with brine (10 mL). The organic layer was dried over $MgSO_4$, filtered and concentrated *in vacuo*. The crude product was then purified with a short silica plug, due to instability of products on silica leading to hydrolysis.

N.B. β-bromoamines were unstable on silica gel and proved to be unstable for MS analysis with a variety of ionization methods (HRMS of the molecular ion was therefore not recorded). To prevent any decomposition, bromides were always stored in the freezer.

trans-N,N-Dibenzyl-4-bromotetrahydrofuran-3-amine (_rac_-2c)

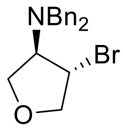

rac-**2c**

Title compound *rac*-**2c** was prepared following *General Procedure V* from **S10** (283 mg, 1.00 mmol, 1 equiv.). The crude product was purified with a short silica plug (9:1 (v/v) pentane/Et_2O) affording a colorless oil (212 mg, 61%).

^{1}H NMR (400 MHz, $CDCl_3$): δ 7.31–7.16 (m, 10H), 4.40–4.36 (m, 1H), 4.25 (dd, J = 10.3, 6.8 Hz, 1H), 3.89 (dd, J = 9.4, 7.5 Hz, 1H), 3.80–3.76 (m, 3H), 3.73 (d, J = 13.8 Hz, 2H), 3.58 (d, J = 13.8 Hz, 2H) ppm; **^{13}C NMR** (101 MHz, $CDCl_3$): δ 138.8, 128.7, 128.4, 127.3, 76.1, 69.9, 69.2, 54.7, 44.8 ppm; **HRMS** (ESI$^+$) = *m/z* calculated for $C_{18}H_{21}BrNO^+$ [M+H]$^+$ 346.0801, found 346.0800; **IR** (thin layer film): ν 3028, 2862, 1493, 1453, 1136, 1087, 1026, 922, 746, 697 cm^{-1}.

tert-Butyl trans-3-bromo-4-(dibenzylamino)pyrrolidine-1-carboxylate (rac-2d)

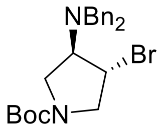

rac-**2d**

Title compound *rac*-**2d** was prepared following *General Procedure V* from **S11** (382 mg, 1.00 mmol, 1 equiv.). The crude product was purified with a short silica plug (7:3 (v/v) pentane/Et_2O) affording a light-yellow solid (330 mg, 73%).

^{1}H NMR (500 MHz, $CDCl_3$, *mixture of rotamers*): δ 7.40 (br d, J = 7.2 Hz, 4H), 7.33 (br t, J = 6.8 Hz, 4H), 7.27–7.24 (br m, 2H), 4.38 (br s, 1H), 3.99 (br t, J = 8.6 Hz, 0.4H, minor), 3.91 (br t, J = 8.6 Hz, 0.6H, major), 3.84–3.80 (m, 2H), 3.72–3.42 (m, 5H), 3.34 (br s, 1H), 1.44 (br s, 9H) ppm; **^{13}C NMR** (101 MHz, $CDCl_3$, *mixture of rotamers*): δ 154.0, 139.0, 128.8, 128.5, 127.4, 80.1, 66.7 (minor), 65.7 (major), 54.4, 53.5, 46.0 (major), 44.6 (major), 44.6 (minor), 44.2 (major), 28.6 ppm; **IR** (thin layer film): ν 3028, 2976, 2886, 1696, 1454, 1403, 1366, 1166, 1136, 1115, 767, 698 cm^{-1}.

Note: ^{1}H NMR spectrum appeared broad due to mixture of rotamers.

trans-N,N-Dibenzyl-2-bromocycloheptan-1-amine (rac-2e)

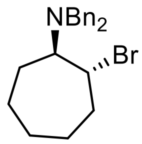

rac-**2e**

Alcohol **S12** (226 mg, 0.73 mmol, 1 equiv.), CBr_4 (1.39 g, 0.77 mmol) and PPh_3 (202 mg, 0.77 mmol) were stirred in anhydrous CH_3CN (3.5 mL) at r.t. for 18 h. Following, Et_2O (5 mL) was added causing the precipitation of a white solid. The mixture was filtered, washed with Et_2O (2 x 3 mL), and the filtrate was concentrated *in vacuo*. The crude product was then purified with a short silica plug (95:5 (v/v) pentane/EtOAc) affording a white waxy solid (128 mg, 47%). Note: Product was unstable on silica.

^{1}H NMR (400 MHz, $CDCl_3$): δ 7.37 (br d, J = 7.5 Hz, 4H), 7.23 (br t, J = 7.6 Hz, 4H), 7.16 (br t, J = 7.3 Hz, 2H), 4.37 (dt, J = 8.7, 4.5 1H), 3.71 (d, J = 14.1 Hz, 2H), 3.31 (d, J = 14.1 Hz, 2H), 2.92 (td, J = 8.7, 1.6 Hz, 1H), 2.19–2.12 (m, 1H), 2.04–1.96 (m, 1H), 1.87–1.78 (m, 2H), 1.66–1.60 (m, 1H), 1.52–1.49 (m, 1H), 1.36–1.11 (m, 4H) ppm; **^{13}C NMR** (126 MHz, $CDCl_3$): δ 139.7, 129.3, 128.2, 127.0, 68.1, 59.9, 54.5, 37.2, 29.3, 27.6, 24.8, 23.7 ppm; **IR** (thin layer film): ν 3061, 3027, 2908, 2855, 2800, 1494, 1454, 1148, 744, 698 cm^{-1}.

5. Asymmetric nucleophilic fluorination

5.1. General Procedure VI

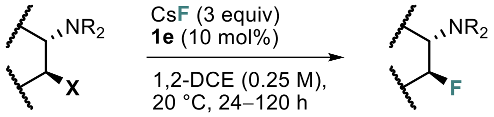

X = Br, Cl *rac*-**2a**–**h** *S,S*-**3a**–**e** *R,R*-**3f**–**h**

To an RBF with a magnetic stirring bar were sequentially added substrate (2.79–0.15 mmol, 1.0 equiv.), catalyst **1e** (10 mol% or 5 mol%), CsF (2.0 or 3.0 equiv.) and dry 1,2-DCE (0.25 M). The reaction mixture was stirred at 900 rpm at r.t. for 24−120 hours. The crude mixture was then filtered through a plug of silica and eluted with CH_2Cl_2, and the filtrate was evaporated to dryness under reduced pressure followed by purification of the crude material by FCC if necessary. The silica plug was further eluted with acetone to recover the catalyst.

Racemate synthesis: The racemic reference products for HPLC analysis were obtained from β-amino-alcohols using DAST (2 equiv.) in DCM (0.2 M) over 24 h at r.t., or from β-bromo-amines using AgF (0.12 equiv.) in MeCN (0.1 M) over 2 h at 60 °C.[1]

5.2. Characterization of products

(*S*,*S*)-*N*,*N*-Dibenzyl-2-fluorocyclohexan-1-amine (*S*,*S*-3a)

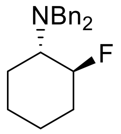

S,*S*-**3a** was prepared from *rac*-**2a** (1.00 g, 2.79 mmol, 1.0 equiv) according to *General Procedure VI* using catalyst **1e** (134 mg, 0.14 mmol, 5 mol%) and CsF (840 mg, 5.58 mmol, 2.0 equiv). The reaction was stirred at r.t. for 120 h. Purification by flash column chromatography (100:0 to 99.6:0.4, gradient, hexane/Et_2O) afforded colorless solid (0.99 g, 83% yield, *e.r.* = 89:11).

^{1}H NMR (400 MHz, $CDCl_3$): δ 7.42–7.07 (m, 10H), 4.55 (dddd, J = 50.7, 10.4, 10.4, 4.8 Hz, 1H), 3.75 (d, J = 13.9 Hz, 2H), 3.67 (d, J = 14.0 Hz, 2H), 2.68–2.54 (m, 1H), 2.12–1.97 (m, 1H), 1.91–1.76 (m, 1H), 1.67–1.52 (m, 2H), 1.35–1.17 (m, 2H), 1.15–0.93 (m, 2H); **^{19}F NMR** (377 MHz, $CDCl_3$): δ -174.71 (d, J = 50.6 Hz); **^{13}C NMR** (101 MHz, $CDCl_3$): δ 140.7, 128.6, 128.1, 126.6, 93.3 (d, J_{CF} = 177.9 Hz), 61.2 (d, J_{CF} = 14.8 Hz), 54.4, 32.6 (d, J_{CF} = 17.5 Hz), 27.7 (d, J_{CF} = 8.8 Hz), 24.9 (d, J_{CF} = 2.2 Hz), 24.0 (d, J_{CF} = 11.4 Hz); **HRMS** (ESI+) = *m/z* calculated for $C_{20}H_{25}FN^+$ $[M+H]^+$ 298.1966, found 298.1969; **HPLC separation**: DAICEL CHIRALPAK® IB-3, Heptane:*i*PrOH = 99.5:0.5, 1 mL/min; t_1 = 3.52 min (major), t_2 = 4.69 min (minor). Spectroscopic data were in agreement with the ones previously reported in literature.[1]

The value for optical rotation of (*R*,*R*)-*N*,*N*-dibenzyl-2-fluorocyclohexan-1-amine ((*R*,*R*)-**2**) was previously reported as $[\alpha]_D^{25℃}$ = –9.0 (*c* 0.2, $CHCl_3$, *e.r.* = 85.5:14.5.[1] The optical rotation of the obtained product above was $[\alpha]_D^{20℃}$ = +11.2 (*c* 1.0, $CHCl_3$, *e.r.* = 88.8:11.2) indicating (*S*,*S*) configuration. The configuration of the other cyclic products was assigned by analogy.

(*S*,*S*)-*N*,*N*-Dibenzyl-2-fluorocyclopentan-1-amine (*S*,*S*-3b)

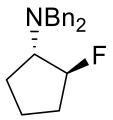

S,S-**3b**

S,*S*-**3b** was prepared from *rac*-**2b** (344 mg, 1.00 mmol, 1 equiv.) according to *General Procedure VI* using catalyst **1e** (48 mg, 0.05 mmol, 5 mol%). The reaction was stirred at r.t. for 96 h. Residue was purified via flash column chromatography (100:0 to 99.6:0.4 (v/v) hexane:Et_2O) giving title product as a white solid (244 mg, 86%, *e.r.* = 81:19).

^{1}H NMR (400 MHz, $CDCl_3$): δ 7.41–7.36 (m, 4H), 7.34–7.28 (m, 4H), 7.27–7.19 (m, 2H), 5.15 (ddt, J = 54.0, 6.9, 3.6 Hz, 1H), 3.70 (d, J = 14.1 Hz, 2H), 3.63 (d, J = 14.1 Hz, 2H), 3.37 (dtd, J = 28.5, 8.5, 3.9 Hz, 1H), 2.01–1.72 (m, 3H), 1.72–1.48 (m, 3H); **^{19}F NMR** (377 MHz, $CDCl_3$): δ -172.99 (dtd, J = 53.5, 28.3, 23.5 Hz); **^{13}C NMR** (101 MHz,

$CDCl_3$): δ 139.9, 128.8, 128.3, 127.0, 97.1 (d, J_{CF} = 176.9 Hz), 68.1 (d, J_{CF} = 22.3 Hz), 55.5, 32.8 (d, J_{CF} = 22.8 Hz), 27.4 (d, J_{CF} = 5.8 Hz), 22.0 (d, J_{CF} = 2.1 Hz); **HRMS** (ESI+) = *m/z* calculated for $C_{19}H_{22}FNK^+$ $[M+K]^+$ 322.1368, found 322.1375; $[\alpha]_D^{20℃}$ = +9.4 (*c* 1.0, $CHCl_3$, *e.r.* = 82:18) [lit.[1] $[\alpha]_D^{25℃}$ = –10.0 (*c* 0.5, $CHCl_3$, *e.r.* = 74.5:25.5, for (*R*,*R*) configuration)]; **HPLC separation**: DAICEL CHIRALPAK® OJ-H, Heptane:EtOH = 98:2, 1 mL/min; t_1 = 8.09 min (major), t_2 = 10.13 min (minor). Spectroscopic data were in agreement with the ones previously reported in literature.[1]

(3*S*,4*R*)-*N*,*N*-Dibenzyl-4-fluorotetrahydrofuran-3-amine (3*S*,4*R*-3c)

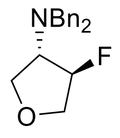

3*S*,4*R*-**3c**

3*S*,4*R*-**3c** was prepared from *rac*-**2c** (345 mg, 1.00 mmol, 1 equiv.) according to *General Procedure VI* using catalyst **1e** (48 mg, 0.05 mmol, 5 mol%). The reaction was stirred at r.t. for 96 h. Residue was purified via flash column chromatography (95:5 (v/v) hexane:Et_2O) giving the title product as a colorless oil (263 mg, 92%, *e.r.* = 85:15).

^{1}H NMR (400 MHz, $CDCl_3$): δ 7.25–7.16 (m, 10H), 5.22 (br d, *J* = 55.0 Hz, 1H), 3.94–3.84 (m, 3H), 3.70–3.47 (m, 6H) ppm; **^{19}F NMR** (377 MHz, $CDCl_3$): δ –177.43 (appt sx, *J* = 27.5 Hz); **^{13}C NMR** (101 MHz, $CDCl_3$): δ 138.8, 128.7, 128.4, 127.3, 94.8 (d, $^1J_{CF}$ = 182 Hz), 73.3 (d, $^2J_{CF}$ = 26 Hz), 70.0 (d, $^3J_{CF}$ = 3 Hz), 67.5 (d, $^2J_{CF}$ = 24 Hz), 55.6 ppm; **IR** (thin layer film): *v* 3064, 2805, 1494, 1454, 1368, 1255, 1209, 1093, 1028, 976, 925, 751. 699, 650 cm^{-1}; **HRMS** (ESI+) = *m/z* calculated for $C_{18}H_{21}FNO^+$ $[M+H]^+$ 286.1602, found 286.1604; $[\alpha]_D^{20℃}$ = –4.2 (*c* 1.0, $CHCl_3$, *e.r.* = 85:15); **HPLC separation**: DAICEL CHIRALPAK® IB-3, Heptane:*i*PrOH = 99.5:0.5, 1 mL/min; t_1 = 13.07 min (major), t_2 = 17.90 min (minor).

Crystallization: 3*S*,4*R*-**3c** (290 mg, 85:15 e.r., 1 equiv.) and trichloroacetic acid (TCA, 150 mg, 1 equiv.) were dissolved in EtOH (2.0 mL) at 60 °C and volatiles were removed in vacuo giving 3*S*,4*R*-**3c**·TCA as a white solid (442 mg, *quantitative* yield). **^{1}H NMR** (600 MHz, $CDCl_3$): δ 9.21 (br s, 1H), 7.47–7.42 (m, 10H), 5.93 (ddt, $^1J_{HF}$ = 53.8 Hz, J_{HH} = 5.2, 2.3 Hz 1H), 4.26 (d, *J* = 13.9 Hz, 2H), 4.19–4.02 (m, 6H), 3.86 (dtd, $^3J_{HF}$ = 25.8 Hz, J_{HH} = 7.4, 2.2 Hz, 1H) ppm; **^{19}F NMR** (565 MHz, $CDCl_3$): δ −176.62 (ddt, 53.8, 30.0, 24.9 Hz, 1F) ppm; **^{13}C NMR** (151 MHz, $CDCl_3$): δ 165.4, 130.7, 130.0 (br s), 129.9, 129.5, 94.9, 93.8 (d, $^1J_{CF}$ = 184 Hz), 73.2 (d, $^2J_{CF}$ = 26 Hz), 69.1 (d, $^2J_{CF}$ = 26 Hz), 68.4 (d, $^3J_{CF}$ = 3 Hz), 55.8 ppm; **m.p.** 107−108 °C.

3*S*,4*R*-**3c**·TCA (442 mg) and *i*PrOH (0.5 ml) were heated to 65 °C until complete dissolution of the solid. The solution was allowed to cool down step-wise to 35 °C over 4 h, and further crystallization for 3 h at 35 °C. The mother liquor was removed at 35 °C and the obtained crystals were washed with cold *i*PrOH (0.1 mL x 2), affording colorless crystals of near-racemic material (145 mg, 33%, e.r. 60:40) as well as enantioenriched product after evaporation of the mother liquor (264 mg, 60%, *e.r.* 98:2). All the *e.r.* were determined of the free amine base by neutralizing **3c**·TCA samples with 1.0 M NaOH (aq) and extracting with Et_2O. The solvent was evaporated *in vacuo* and *e.r.* was measured by chiral HPLC.

***tert*-Butyl (3*S*,4*S*)-3-(dibenzylamino)-4-fluoropyrrolidine-1-carboxylate (*S*,*S*-3d)**

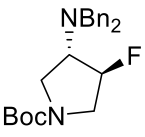

S,S-**3d**

S,*S*-**3d** was prepared from *rac*-**2d** (89 mg, 0.20 mmol, 1 equiv.) according to *General Procedure VI* using catalyst **1e** (19 mg, 0.02 mmol, 10 mol%). The reaction was stirred at r.t. for 72 h. Purification by flash column chromatography (90:10 (v/v), hexane/Et_2O) afforded white solid (67 mg, 87% yield, *e.r.* = 76:24).

^{1}H NMR (400 MHz, $CDCl_3$): δ 7.36–7.24 (m, 10H), 5.23 (br d, J = 52.2 Hz, 1H), 3.72–3.40 (m, 9H), 1.45 (s, 9H) ppm; **^{19}F NMR** (377 MHz, $CDCl_3$, two conformers): δ –179.31 to −179.63 (m), –179.68 to −180.00 (m) ppm; **^{13}C NMR** (101 MHz, $CDCl_3$, two conformers): δ 154.3, 139.0, 128.7, 128.5, 127.4, 93.1 (d, $^1J_{CF}$ = 182 Hz), 92.0 (d, $^1J_{CF}$ = 182 Hz), 80.0, 64.7 (d, $^2J_{CF}$ = 24 Hz), 63.9 (d, $^2J_{CF}$ = 24 Hz), 55.1, 50.9 (t, $^2J_{CF}$ = 27 Hz), 47.1, 46.2, 28.6 ppm; **HRMS** (ESI+) = *m/z* calculated for $C_{23}H_{30}FN_2O_2^+$ [M+H]$^+$ 385.2286, found 385.2277; **IR** (thin layer film): v 3029, 2975, 2885, 1698, 1454, 1403, 1366, 1171, 1142, 1118, 882, 745, 698 cm^{-1}; $[\alpha]_D^{20℃}$ = 11.2 (*c* 1.0, $CHCl_3$, *e.r.* = 76:24); **HPLC separation**: DAICEL CHIRALPAK® IB-3, Heptane:*i*PrOH = 99.0:1.0, 1 mL/min; t_1 = 7.60 min (major), t_2 = 11.42 min (minor).

(1*S*,2*S*)-*N*,*N*-Dibenzyl-2-fluorocycloheptan-1-amine (*S*,*S*-3e)

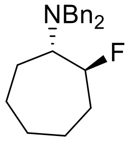

S,S-**3e**

S,*S*-**3e** was prepared from *rac*-**2e** (69 mg, 0.18 mmol, 1 equiv) according to *General Procedure VI* catalyst **1e** (16 mg, 0.018 mmol, 10 mol%). The reaction was stirred at r.t. for 72 h. Purification by flash column chromatography (99.5:0.5 (v/v) hexane/Et_2O) afforded white waxy solid (40 mg, 72% yield, *e.r.* = 81:19).

^{1}H NMR (400 MHz, $CDCl_3$): δ 7.42 (br d, J = 7.2 Hz, 4H), 7.30 (t, J = 7.2 Hz, 4H), 7.22 (br t, J = 7.2 Hz, 2H), 4.88 (dddd, J = 48.3, 7.7, 5.4, 3.9 1H), 3.78 (d, J = 14.0 Hz, 2H), 3.58 (d, J = 14.0 Hz, 2H), 2.89 (dddd, J = 20.8, 10.3, 7.7, 2.0 Hz, 1H), 2.00–1.61 (m, 5H), 1.50–1.21 (m, 5H) ppm; **^{19}F NMR** (377 MHz, $CDCl_3$): δ –167.64 (dddd, J = 48.3, 20.8, 15.5, 2.9 Hz); **^{13}C NMR** (101 MHz, $CDCl_3$): δ 140.1, 128.7, 128.3, 126.9, 95.3 (d, $^1J_{CF}$ = 169 Hz), 64.0 (d, $^2J_{CF}$ = 20 Hz), 54.4, 33.4 (d, $^2J_{CF}$ = 22 Hz), 29.5, 27.5, 26.1 (d, $^3J_{CF}$ = 10 Hz), 21.7 (d, $^3J_{CF}$ = 4 Hz) ppm; **HRMS** (ESI+) = *m/z* calculated for $C_{21}H_{27}FN^+$ $[M+H]^+$ 312.2122, found 312.2115; **IR** (thin layer film): ν 3061, 3028, 2930, 2856, 2800, 1494, 1454, 1369, 1120, 1023, 973, 741, 698 cm^{-1}; $[\alpha]_D^{20°C}$ = 6.0 (c 1.0, $CHCl_3$, *e.r.* = 82:18); **HPLC separation**: DAICEL CHIRALPAK® IC-3, Heptane:*i*PrOH = 99.5:0.5, 1 mL/min; t_1 = 3.37 min (major), t_2 = 3.76 min (minor).

2-((1*R*,2*R*)-2-Fluoro-1,2-diphenylethyl)-1,2,3,4-tetrahydroisoquinoline (*R*,*R*-3f)

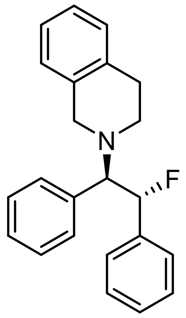

R,R-**3f**

R,*R*-**3f** was prepared from *rac*-**2f** (52 mg, 0.15 mmol, 1 equiv.) according to *General Procedure VI* using catalyst **1e** (14 mg, 0.015 mmol, 10 mol%). The reaction was stirred at r.t. for 72 h. Residue was purified *via* silica plug filtration with CH_2Cl_2 giving product as a colorless oil (47 mg, 94%, *e.r.* 24:76).

^{1}H NMR (400 MHz, $CDCl_3$): δ 7.19–7.10 (m, 13H), 7.01 (br d, J = 6.1 Hz, 1H), 6.04 (br dd, J = 47.5, 7.0 Hz, 1H), 4.16 (dd, J = 14.3, 8.3 Hz, 1H), 3.99–3.89 (m, 2H), 3.16 (br s, 1H), 3.01–2.85 (m, 3H) ppm; **^{19}F{^{1}H} NMR** (377 MHz, $CDCl_3$): δ −172.25 (s, 1F) ppm; **^{13}C NMR** (101 MHz, $CDCl_3$): δ 138.0 (d, J_{CF} = 21 Hz), 136.4 (d, J_{CF} = 6 Hz), 135.3, 134.7, 129.5, 128.7, 128.2 (d, J_{CF} = 2 Hz), 128.1, 128.0, 127.7, 126.9 (d, J_{CF} = 6 Hz), 126.8, 126.2, 125.6, 94.8 (d, J_{CF} = 179 Hz), 73.5 (d, J_{CF} = 22 Hz), 53.7 (d, J_{CF} = 3 Hz), 48.3 (br s), 29.1 ppm; **HRMS** (ESI+) = *m/z* calculated for $C_{23}H_{23}FN^+$ $[M+H]^+$ 332.1809, found 332.1799; **HPLC separation**: DAICEL CHIRALPAK® IF-3, Heptane:*i*PrOH = 99:1, 1 mL/min; t_1 = 4.97 min (minor), t_2 = 6.09 min (major). Spectroscopic data were in agreement with the ones previously reported in literature.[1]

The (*R*,*R*) configuration was assigned based on HPLC conditions reported previously for (*S*,*S*) product (*e.r.* 96:4).[1] HPLC separation: DAICEL CHIRALPAK® IF-3, Heptane:iPrOH = 99:1, 1 mL/min; t_1 = 4.77 min (major), t_2 = 5.83 min (minor). The configuration of the other slibene-based products was assigned by analogy.

1-((1*R*,2*R*)-2-Fluoro-1,2-diphenylethyl)piperidine (*R*,*R*-3g)

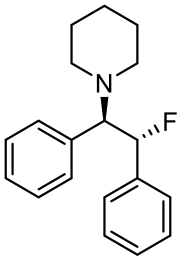

R,*R*-**3g**

R,*R*-**3g** was prepared from *rac*-**2g** (45 mg, 0.15 mmol) according to *General Procedure VI* using catalyst **1e** (14 mg, 0.015 mmol, 10 mol%). The reaction was stirred at r.t. for 24 h. Residue was purified *via* silica plug filtration with CH_2Cl_2 giving product as a white solid (44 mg, 99%, *e.r.* 24:76).

^{1}H NMR (400 MHz, $CDCl_3$): δ 7.21–7.18 (m, 8H), 7.09 (br d, J = 6.6 Hz, 2H), 5.93 (dd, J = 47.4, 8.6 Hz, 1H), 3.97 (dd, J = 13.3, 8.5 Hz, 1H), 2.67–2.52 (m, 4H), 1.73–1.58 (m, 4H), 1.45–1.39 (m, 2H) ppm; **^{19}F{^{1}H} NMR** (377 MHz, $CDCl_3$): δ -174.18 (s, 1F) ppm; **^{13}C NMR** (101 MHz, $CDCl_3$): δ 138.4 (d, J_{CF} = 20 Hz), 135.7 (d, J_{CF} = 6 Hz), 129.6, 128.1 (d, J_{CF} = 2 Hz), 127.9, 127.8, 127.3, 127.0 (d, J_{CF} = 6 Hz), 93.7 (d, J_{CF} = 178 Hz), 74.6 (d, J_{CF} = 22 Hz), 51.5, 26.4, 24.6 ppm; **HRMS** (ESI+) = *m/z* calculated for $C_{19}H_{23}FN^+$ $[M+H]^+$ 284.1809, found 284.1810. **HPLC separation**: DAICEL CHIRALPAK® IC-3, Heptane:EtOH = 99:1, 1 mL/min; t_1 = 6.18 min (major), t_2 = 6.70 min (minor). Spectroscopic data were in agreement with the ones previously reported in literature.[1]

(1*R*,2*R*)-2-Fluoro-*N*,*N*-dimethyl-1,2-diphenylethan-1-amine (*R*,*R*-3h)

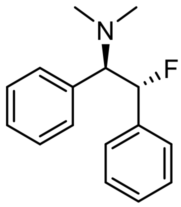

R,*R*-**3h**

R,*R*-**3h** was prepared from *rac*-**2h** (39 mg, 0.15 mmol, 1 equiv.) according to *General Procedure VI* using catalyst **1e** (14 mg, 0.015 mmol, 10 mol%). The reaction was stirred at r.t. for 72 h. Residue was purified *via* silica plug filtration with Et_2O giving product as a colorless oil (33 mg, 92%, *e.r.* 26:74).

^{1}H NMR (400 MHz, $CDCl_3$): δ 7.21–7.15 (m, 8H), 7.08–7.06 (m, 2H), 5.89 (dd, J = 47.2, 8.8 Hz, 1H), 3.93 (dd, J = 12.2, 8.8 Hz, 1H), 2.42 (s, 6H) ppm; **^{19}F{^{1}H} NMR** (377 MHz, $CDCl_3$): δ −172.48 (s, 1F) ppm; **^{13}C NMR** (101 MHz, $CDCl_3$): δ 137.0 (d, J_{CF} = 20 Hz), 134.8 (d, J_{CF} = 7 Hz), 129.5, 128.3 (d, J_{CF} = 2 Hz), 128.0, 127.9, 127.6, 127.0 (d, J_{CF} = 6 Hz), 94.1 (d, J_{CF} = 178 Hz), 74.3 (d, J_{CF} = 22 Hz), 42.7 (d, J_{CF} = 2 Hz) ppm; **HRMS** (ESI+) = *m/z* calculated for $C_{16}H_{19}FN^+$ $[M+H]^+$ 244.1496, found 244.1504; **HPLC separation**: DAICEL CHIRALPAK® IC-3, Heptane:*i*PrOH = 99:1, 1 mL/min; t_1 = 5.43 min (minor), t_2 = 10.44 min (major). Spectroscopic data were in agreement with the ones previously reported in literature.[1]

5.3. Comparison with catalyst U2

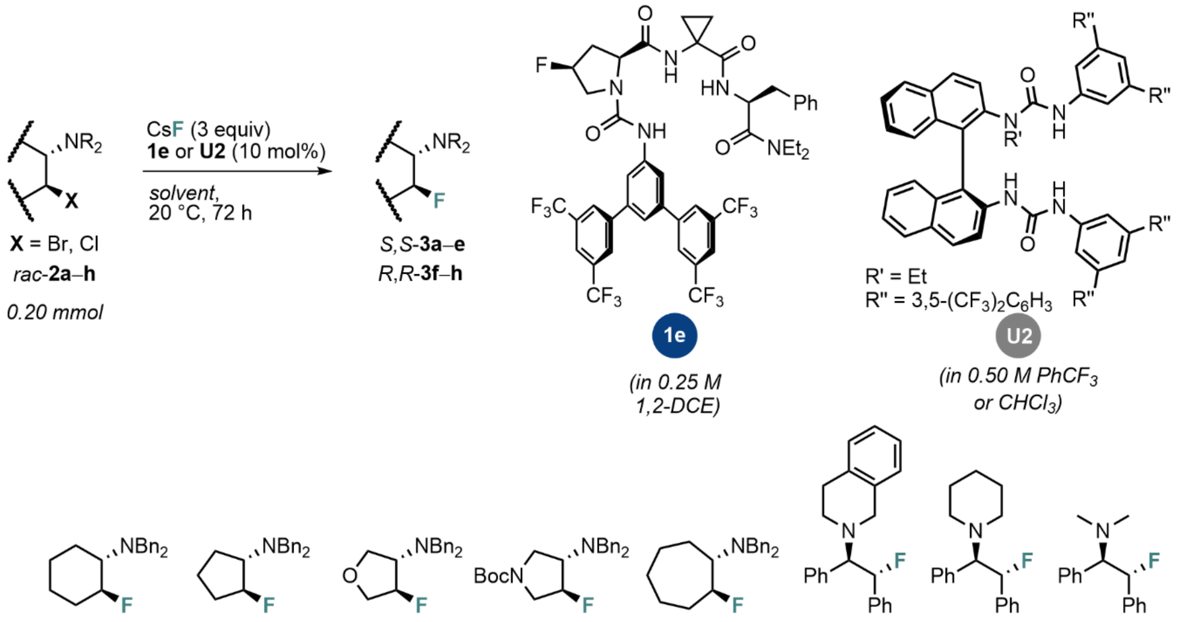

	X = Br<br>*S,S*-**3a**	X = Br<br>*S,S*-**3b**[d]	X = Br<br>*3S,4R*-**3c**	X = Br<br>*S,S*-**3d**	X = Br<br>*S,S*-**3e**	X = Cl<br>*R,R*-**3f**	X = Cl<br>*R,R*-**3g**	X = Cl<br>*R,R*-**3h**
**1e**	*85% yield*<br>*89:11 e.r.*[a]	*96% yield*<br>*81:19 e.r.*[a]	*94% yield*<br>*85:15 e.r.*[a]	*87% yield*<br>*76:24 e.r.*[a]	*72% yield*<br>*81:19 e.r.*[a]	*94% yield*[d]<br>*24:76 e.r.*[a]	*99% yield*[d]<br>*25:75 e.r.*[a]	*92% yield*[d]<br>*26:74 e.r.*[a]
**U2**	*69% yield*[b,c]<br>*14.5:85.5 e.r.*[a]	*71% yield*[b,c]<br>*25.5:74.5 e.r.*[a]	*quant. yield*[c]<br>*29:71 e.r.*[a]	*94% yield*[c]<br>*35:65 e.r.*[a]	*45% yield*[c]<br>*17:83 e.r.*[a]	*91% yield*[b,e]<br>*96:4 e.r.*[a]	*56% yield*[b,e]<br>*96:4 e.r.*[a]	*65% yield*[b,e]<br>*95:5 e.r.*[a]

Figure S1. Catalytic performance of peptidic catalyst **1e** *versus* BINAM *bis*-urea catalyst **U2**. [a]enantiomeric ratios (*e.r.*; (*S,S*):(*R,R*)) measured by HPLC with chiral stationary phase; [b]from ref[1]; [c]in $PhCF_3$; [d]0.15 mmol scale; [e]$CHCl_3$, at -10, 0 or 5°C (see ref[1]).

6. NMR studies

6.1. Solubility determination for catalyst **1b**, **1c** and **1d**.

Catalyst (~0.0055 mmol; 3.5 mg for **1b,** 5.0 mg for **1c**, 5.4 mg for **1d**) and 1,2-DCE (0.5 mL, ~0.0011 mM) were sonicated for 10 min. The resulting suspension was allowed to settle, and the supernatant was filtered through a PTFE syringe filter (0.2 μm pore size). An aliquot (100 μL) of the filtrate was added to NMR tube, followed by addition of 4-fluoroanisole (5.0 μL, 0.044 mmol) with a Hamilton™ syringe, and $CDCl_3$ (0.4 mL). The exact concentrations of catalysts **1b** and **1c** were determined by $^{19}F\{^{1}H\}$ qNMR, integrating the $-CF_3$ peak (−62.96, −62.73 ppm, respectively), and 4-fluoroanisole peak (−124.55 ppm).

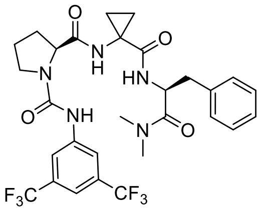

1b

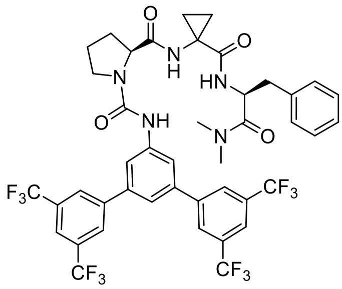

1c

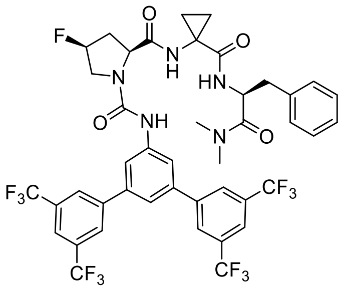

1d

Catalyst	**Solubility** in 1,2-DCE at 20 ̊C (mg/mL)
**1b**	4.4
**1c**	1.1
**1d**	2.1

The data were collected on an AVIII HD 500 at 298 K using the following parameters:

Parameter	Value
TD (SI)	131072 (262144) pt
SW	100 ppm
o1p	−93.7 ppm
NS (DS)	16 (2)
relaxation delay d_1	35 s

6.2. ^{1}H and ^{13}C NMR spectra assignments for **1e**

3,5-[3,5-$(CF_3)_2C_6H_3]_2C_6H_3$NHC(O)-flp-Acpc-Phe-NEt_2 (1e)

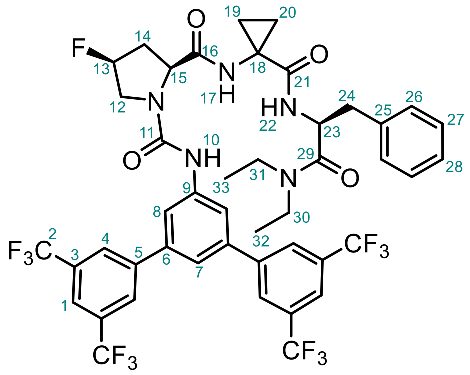

^{1}H (^{1}H-^{1}H COSY, ^{1}H-^{13}C multiplicity-edited HSQC, ^{1}H-^{1}H NOESY) NMR (500 MHz, CD_2Cl_2): δ 8.10 (s, 4H, H^{4}), 7.95 (s, 2H, H^{1}), 7.92 (s, 2H, H^{8}), 7.65 (br s, 1H, H^{10}), 7.48 (s, 1H, H^{7}), 7.32 (s, 1H, H^{17}), 7.20 (d, J = 8.2 Hz, 1H, H^{22}), 7.15–7.14 (m, 3H, H^{27}+H^{28}), 7.00–6.98 (m, 2H, H^{26}), 5.46 (dt, J = 52.4, 3.5 Hz, 1H, H^{13}), 4.87 (q, J = 7.9 Hz, 1H, H^{23}), 4.75 (d, J = 9.6 Hz, 1H, H^{15}), 4.16 (dd, J = 22.4, 11.6 Hz, 1H, H^{12a}), 3.93 (ddd, J = 35.2, 11.8, 3.7 Hz, 1H, H^{12b}), 3.44 (sx, J = 7.0 Hz, 1H, H^{31a}), 3.00–2.91 (m, 3H, H^{31b}+H^{30}), 2.82–2.76 (m, 2H, H^{14a}+H^{24a}), 2.71 (dd, J = 13.3, 6.5 Hz, 1H, H^{24b}), 2.47 (dddd, J = 41.0, 14.7, 10.0, 3.8 Hz, 1H, H^{14b}), 1.49–1.40 (m, 2H, H^{19}), 1.09 (ddd, J = 10.2, 7.2, 4.1 Hz, 1H, H^{20a}), 0.98 (ddd, J = 10.2, 6.5, 3.2 Hz, 1H, H^{20b}), 0.91 (appt q, J = 7.3 Hz, 6H, H^{32}+H^{33}) ppm.

^{13}C (^{1}H-^{13}C multiplicity-edited HSQC, ^{1}H-^{13}C HMBC) NMR (151 MHz, CD_2Cl_2): δ 172.3 (C^{16}), 170.6 (C^{21}), 170.3 (C^{29}), 155.2 (C^{11}), 142.6 (C^{5}), 140.6 (C^{6}), 139.9 (C^{9}), 136.5 (C^{25}), 132.0 (q, J = 33 Hz, C^{3}), 129.3 (C^{26}), 128.1 (C^{27}), 127.5 (br d, J = 3 Hz, C^{4}), 126.6 (C^{28}), 122.3 (q, J = 273 Hz, C^{2}), 121.5 (br t, J = 4 Hz, C^{1}), 121.1 (C^{8}), 119.6 (C^{7}), 92.4 (d, J = 173 Hz, C^{13}), 59.9 (C^{15}), 53.9 (C^{12}, *overlapping with solvent peak*), 50.6 (C^{23}), 41.5 (C^{31}), 40.3 (C^{30}), 39.6 (C^{24}), 35.6 (br d, J = 2 Hz, C^{14}), 34.6 (C^{18}), 16.5 (C^{20}), 16.3 (C^{19}), 13.6 (C^{33}), 12.3 (C^{32}) ppm.

Note: Additional 2D NMR experiments that were used for spectral assignment are given in parenthesis.

^{1}H NMR (600 MHz, CD_2Cl_2): 3,5-[3,5-$(CF_3)_2C_6H_3]_2C_6H_3$NHC(O)-flp-Acpc-Phe-NEt_2 (**1e**)

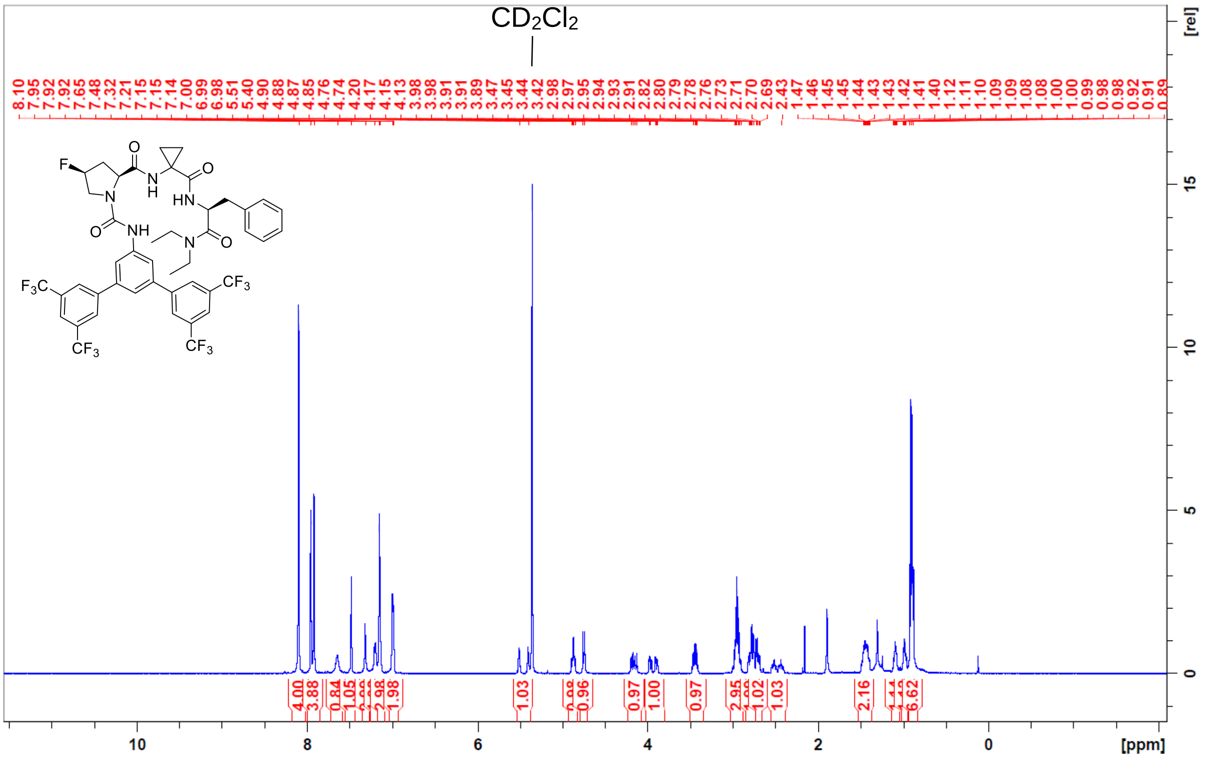

^{13}C NMR (151 MHz, CD_2Cl_2): 3,5-[3,5-$(CF_3)_2C_6H_3]_2C_6H_3$NHC(O)-flp-Acpc-Phe-NEt_2 (**1e**)

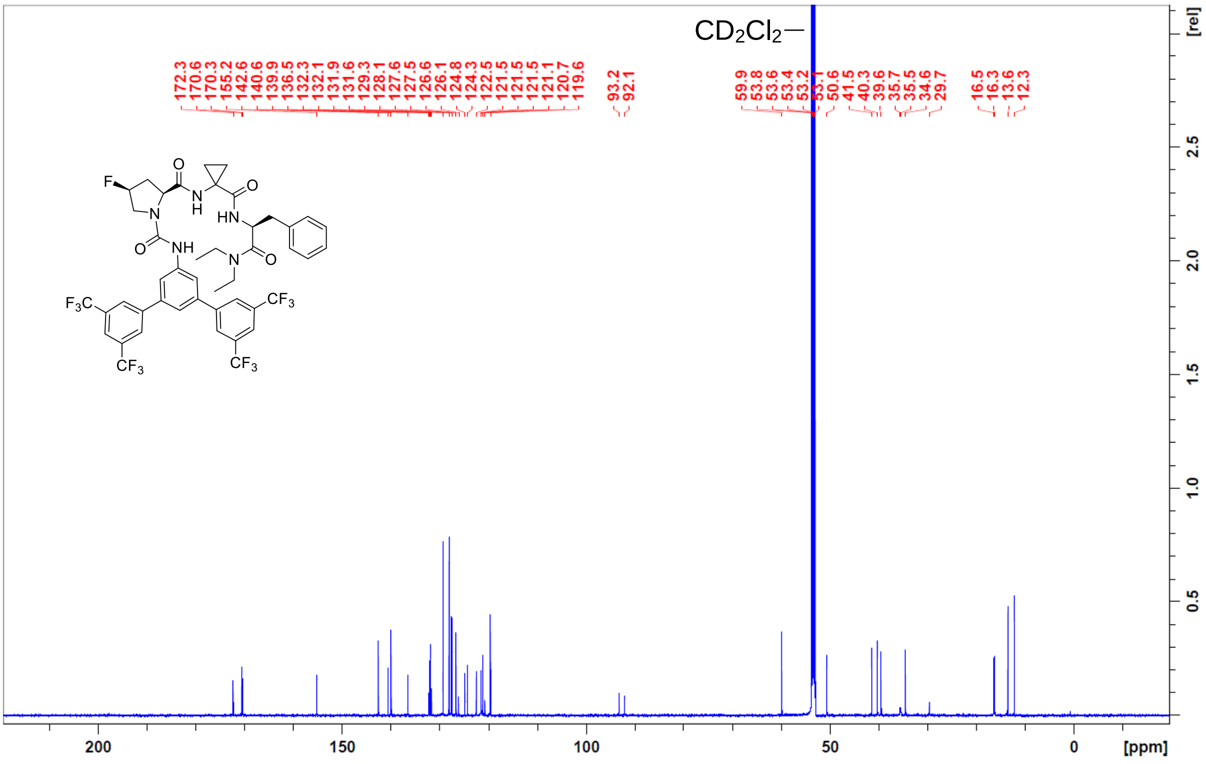

^{1}H-^{1}H COSY NMR (500 MHz, CD_2Cl_2): 3,5-[3,5-$(CF_3)_2C_6H_3]_2C_6H_3NHC(O)$-flp-Acpc-Phe-$NEt_2$ (**1e**)

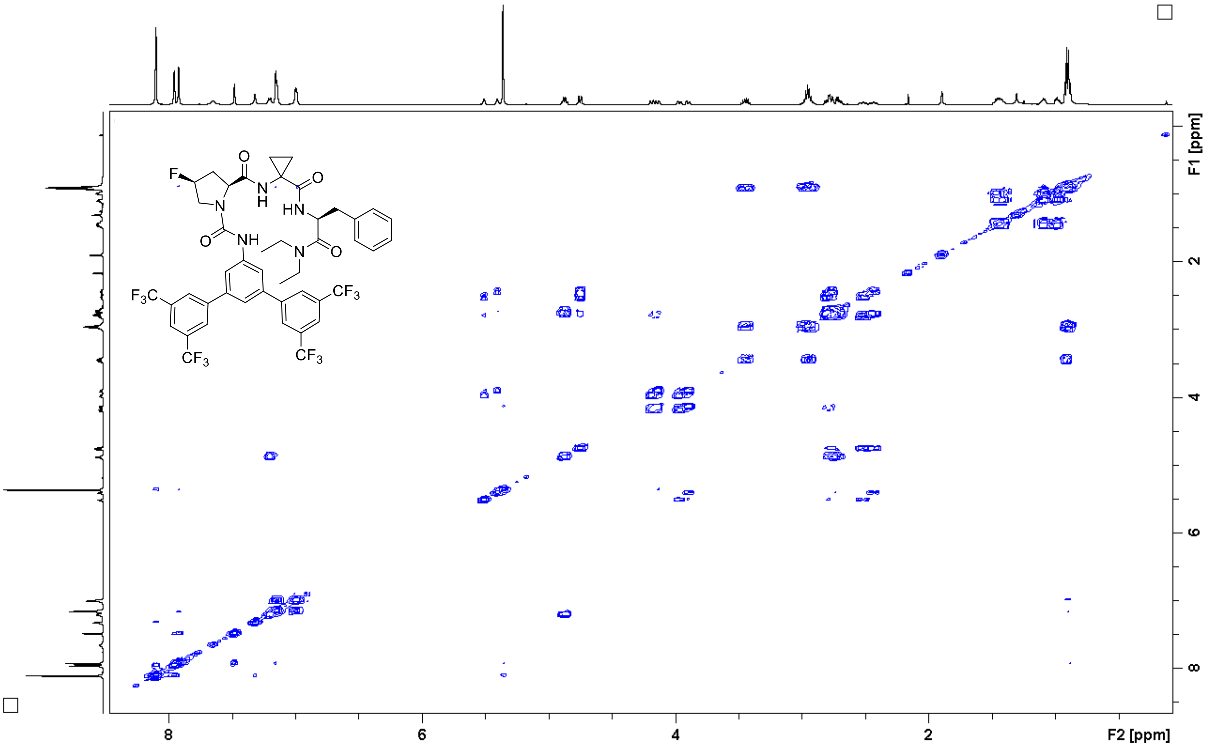

^{1}H-^{13}C multiplicity-edited HSQC NMR (500 MHz, CD_2Cl_2): 3,5-[3,5-$(CF_3)_2C_6H_3]_2C_6H_3NHC(O)$-flp-Acpc-Phe-$NEt_2$ (**1e**)

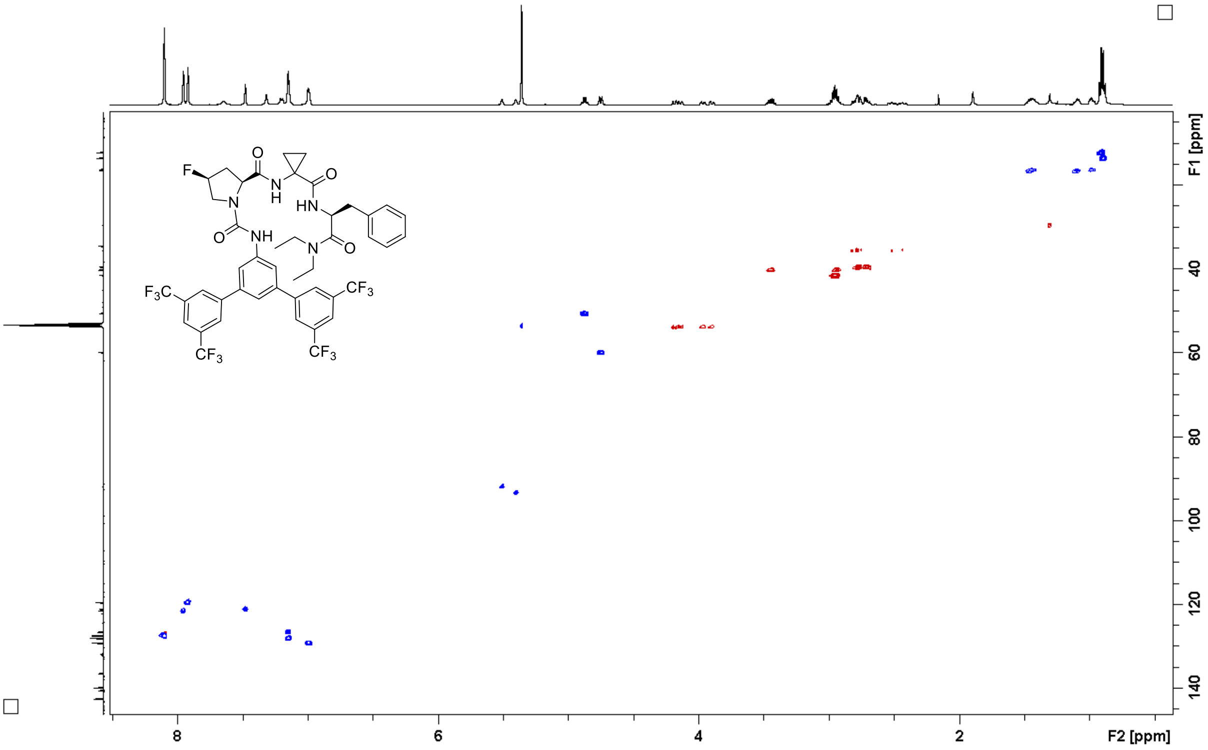

^{1}H-^{13}C HMBC NMR (500 MHz, CD_2Cl_2): 3,5-[3,5-$(CF_3)_2C_6H_3]_2C_6H_3NHC(O)$-flp-Acpc-Phe-$NEt_2$ (**1e**)

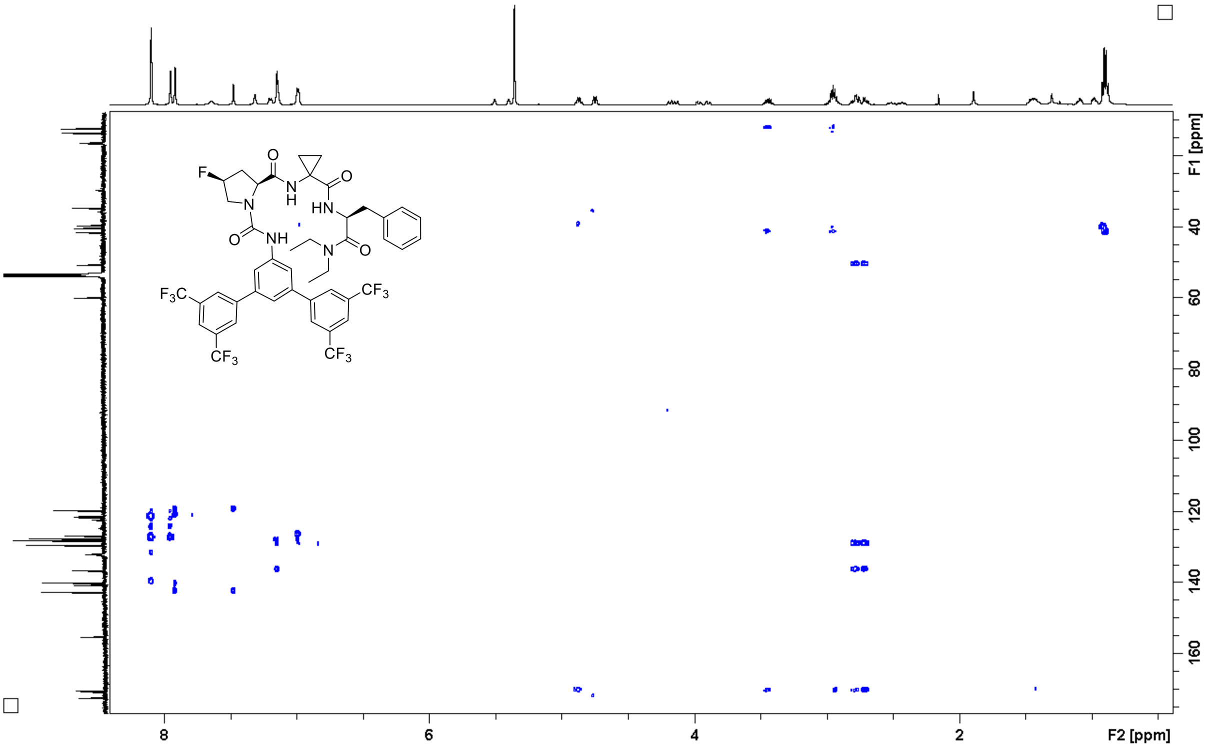

^{1}H-^{1}H NOESY NMR (500 MHz, CD_2Cl_2, 800 ms): 3,5-[3,5-$(CF_3)_2C_6H_3]_2C_6H_3NHC(O)$-flp-Acpc-Phe-$NEt_2$ (**1e**)

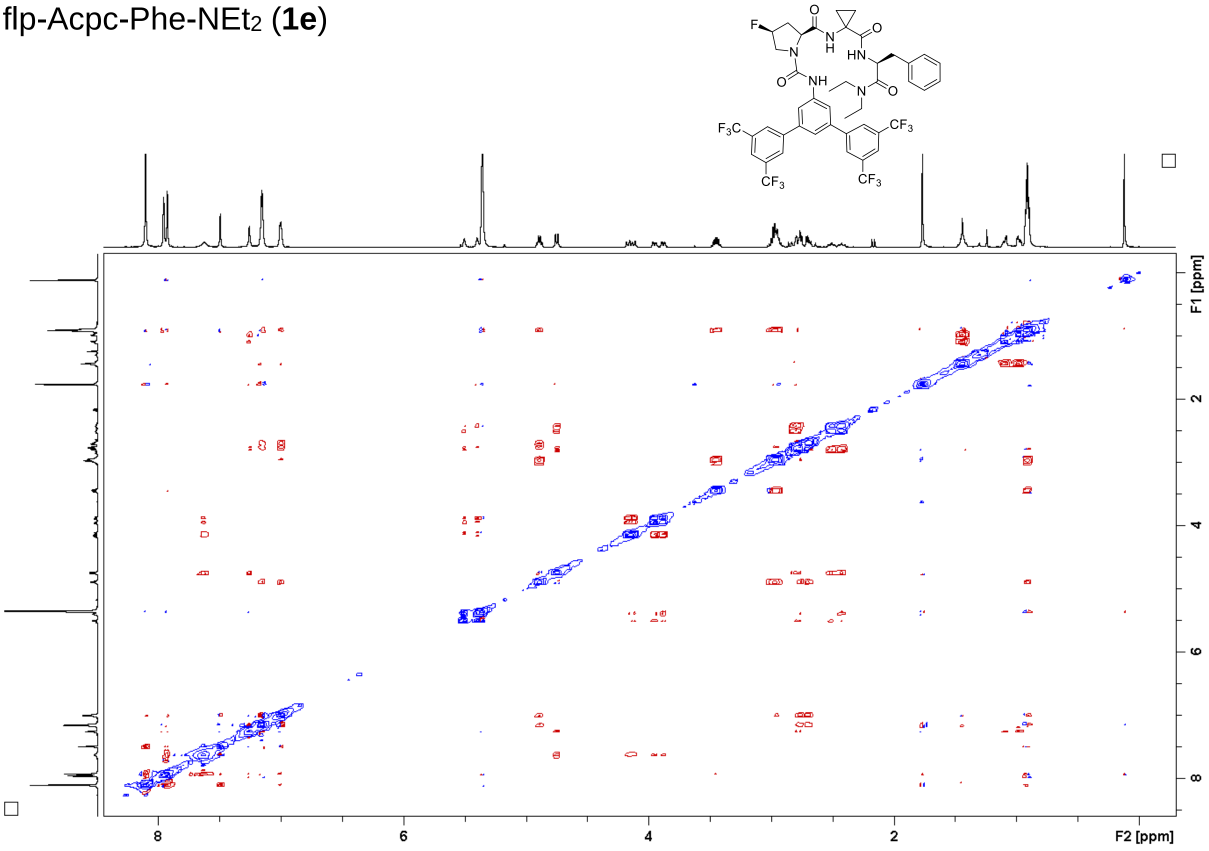

6.3. NMR titrations of catalysts **1a−1e**

Titrations were performed by following the procedure of Ibba *et al.*[17]

A known amount of catalyst was weighted into an NMR tube and dissolved in 0.50 mL of CD_2Cl_2 with 5.0 µL 4-fluoroanisole. A TBAF stock solution was prepared by dissolving ~50 mg of TBAF·$3H_2O$ (Merck, tetrabutylammonium fluoride trihydrate 98%) and 5.0 µL of 4-fluoranisole in CD_2Cl_2 made up to 1.0 mL in a volumetric flask.

The exact concentrations of fluoride and catalysts **1b-e** were determined by ^{19}F qNMR, integrating the F^- peak (–118.9 ppm for TBAF) or -CF_3 peak (around –63 ppm for catalysts), and 4-fluoroanisole peak (–125 ppm). Catalyst **1a** was quantified *via* ^1H qNMR, integrating the 4-fluoroanisole peak (6.89 ppm) and α-protons (5.05, 4.50 and 4.43 ppm, averaged). Data were collected on an AVIII HD 500 at 298 K using the following parameters:

^{19}F qNMR for TBAF·$3H_2O$ and **1b**-**1d** solutions

Parameter	Value
TD (SI)	131072 (262144) pt
SW	20 and 100 ppm
o1p	-122 and -94 ppm
NS (DS)	8 (2)
relaxation delay d_1	35 s

^1H qNMR for **1a** solution

Parameter	Value
TD (SI)	131072 (262144) pt
SW	20 ppm
o1p	-122 ppm
NS (DS)	8 (4)
relaxation delay d_1	75 s

The TBAF solution was added in aliquots from 0 to 3 or 0 to 5 equivalents for **1b**-**1e** and 0 to 10 equivalents for **1a**. After each addition, ^1H NMR spectra were acquired on an AVIIIHD 500 or AVIIIHD 600. The chemical shifts of selected proton signals were plotted against the concentration of added fluoride. The association constants ($K_{a,1:1}$) were determined from the titration data points by non-linear least squares regression using DynaFit 4.08.161.[18] 1:1, 1:2 and 2:1 binding modes were considered, with 1:1 giving superior fitting results, while attempts to fit the data to another binding mode resulted in poor fit and nonsensical data.

The following shows a typical input file for the determination of $K_{a,1:1}$:

```
[task]
   task = fit
   data = equilibria

[mechanism]
   U + F <==> UF : Ka equilibrium

[constants]
Ka = 1000 ?

[responses]
intensive

[data]
variable F, U
plot titration

set NMR.a | resp U = 4.9007, UF = 4.7206 ?
set NMR.b | resp U = 4.7560, UF = 5.0680 ?
set NMR.c | resp U = 7.1660,  UF = 8.640 ?

[set:NMR.a]
0           0.006811111     4.9007
0.000292018     0.006796009     4.8924
0.000872185     0.006766004     4.8663
0.002017287     0.006706783     4.8188
0.002582353     0.00667756      4.8000
0.003142516     0.00664859      4.7844
0.003973707     0.006605603     4.7637
0.004794218     0.006563169     4.7500
0.005604255     0.006521277     4.7380
0.006668354     0.006466245     4.7311
0.007714644     0.006412134     4.7271
0.008743568     0.006358921     4.7260
0.009755556     0.006306584     4.7257
0.01075102      0.006255102     4.7253
0.01317   0.00613   4.7235
0.015494118     0.006009804     4.7239
0.017728846     0.005894231     4.7224
0.019879245     0.005783019     4.7221
0.023945455     0.005572727     4.7206
0.027726316     0.005377193     4.7206

[set:NMR.b]
0           0.006811111     4.7560
0.000292018     0.006796009     4.7642
0.000872185     0.006766004     4.7994
0.002017287     0.006706783     4.8669
0.002582353     0.00667756      4.9002
0.003142516     0.00664859      4.9325
0.003973707     0.006605603     4.9774
0.004794218     0.006563169     5.0131
0.005604255     0.006521277     5.0367
0.006668354     0.006466245     5.0575
0.007714644     0.006412134     5.0570
0.008743568     0.006358921     5.0594
0.009755556     0.006306584     5.0640
0.01075102      0.006255102     5.0650
0.01317   0.00613   5.0640
0.015494118     0.006009804     5.0650
0.017728846     0.005894231     5.0631
0.019879245     0.005783019     5.0660
0.023945455     0.005572727     5.0660
0.027726316     0.005377193     5.0680

[set:NMR.c]
0           0.006811111     7.1660
0.000292018     0.006796009     7.2000
0.000872185     0.006766004     7.3290
0.001447253     0.006736264     7.4610
0.002017287     0.006706783     7.6040
0.002582353     0.00667756      7.7430
0.003142516     0.00664859      7.8920
0.003973707     0.006605603     8.1270
0.004794218     0.006563169     8.3260
0.005604255     0.006521277     8.4860
0.006668354     0.006466245     8.6230
0.007714644     0.006412134     8.6860
0.008743568     0.006358921     8.7040
0.009755556     0.006306584     8.7110
0.01075102      0.006255102     8.7150
0.01317   0.00613   8.7040
0.015494118     0.006009804     8.7000
0.017728846     0.005894231     8.6840
0.019879245     0.005783019     8.6790
0.023945455     0.005572727     8.6630
0.027726316     0.005377193     8.6400

[output]
   directory ./output
[end]
```

Table S8. Difference in chemical shifts of NH protons between free and TBAF·3H_2O bound catalysts ($\Delta\delta$ NH), and association constants (K_a) for the formation of 1:1 catalyst-TBAF·3H_2O complexes.

Catalyst	$\Delta\delta$ NH(i−1)	$\Delta\delta$ NH(i+1)	$\Delta\delta$ NH(i+2)	$K_{a\ (1:1)\ TBAF}$ (M^{-1})	Mean Square Error	log($K_{a,\ 1:1\ TBAF}$)
**1a** (exp 1)	0.20[a]	3.51[a]	0.03[a]	103	5.8×10^{-6}	2.013
**1a** (exp 2)	0.17[a]	3.22[a]	0.01[a]	101	1.9×10^{-6}	2.003
**1a** (average)	0.19[a]	3.37[a]	0.02[a]	102 ± 1		2.008 ± 0.005
**1b** (exp 1)	4.68	2.25	1.24	7 400	3.3×10^{-5}	3.89
**1b** (exp 2)	4.58	2.18	1.27	6 900	7.8×10^{-5}	3.84
**1b** (average)	4.63	2.22	1.26	7 400 ± 500		3.87 ± 0.03
**1c** (exp 1)	4.26	2.47	0.89	7 900	1.2×10^{-4}	3.90
**1c** (exp 2)	4.19	2.49	0.95	9 100	3.1×10^{-5}	3.96
**1c** (average)	4.19	2.48	0.92	8 500 ± 600		3.93 ± 0.03
**1d** (exp 1)	4.96	1.49	1.68	31 000	4.4×10^{-4}	4.49
**1d** (exp 2)	5.01	1.49	1.71	23 000	4.0×10^{-5}	4.36
**1d** (average)	4.98	1.49	1.70	27 000 ± 4 000		4.43 ± 0.06
**1e** (exp 1)	4.62	1.42	1.83	47 000	2.6×10^{-4}	4.66
**1e** (exp 2)	4.70	1.45	1.78	34 100	2.8×10^{-4}	4.53
**1e** (average)	4.66	1.44	1.81	40 000 ± 6 000		4.60 ± 0.07

[a]$\Delta\delta$ NH = δ NH (TBAF 10 equiv) − δ NH (TBAF 0 equiv).

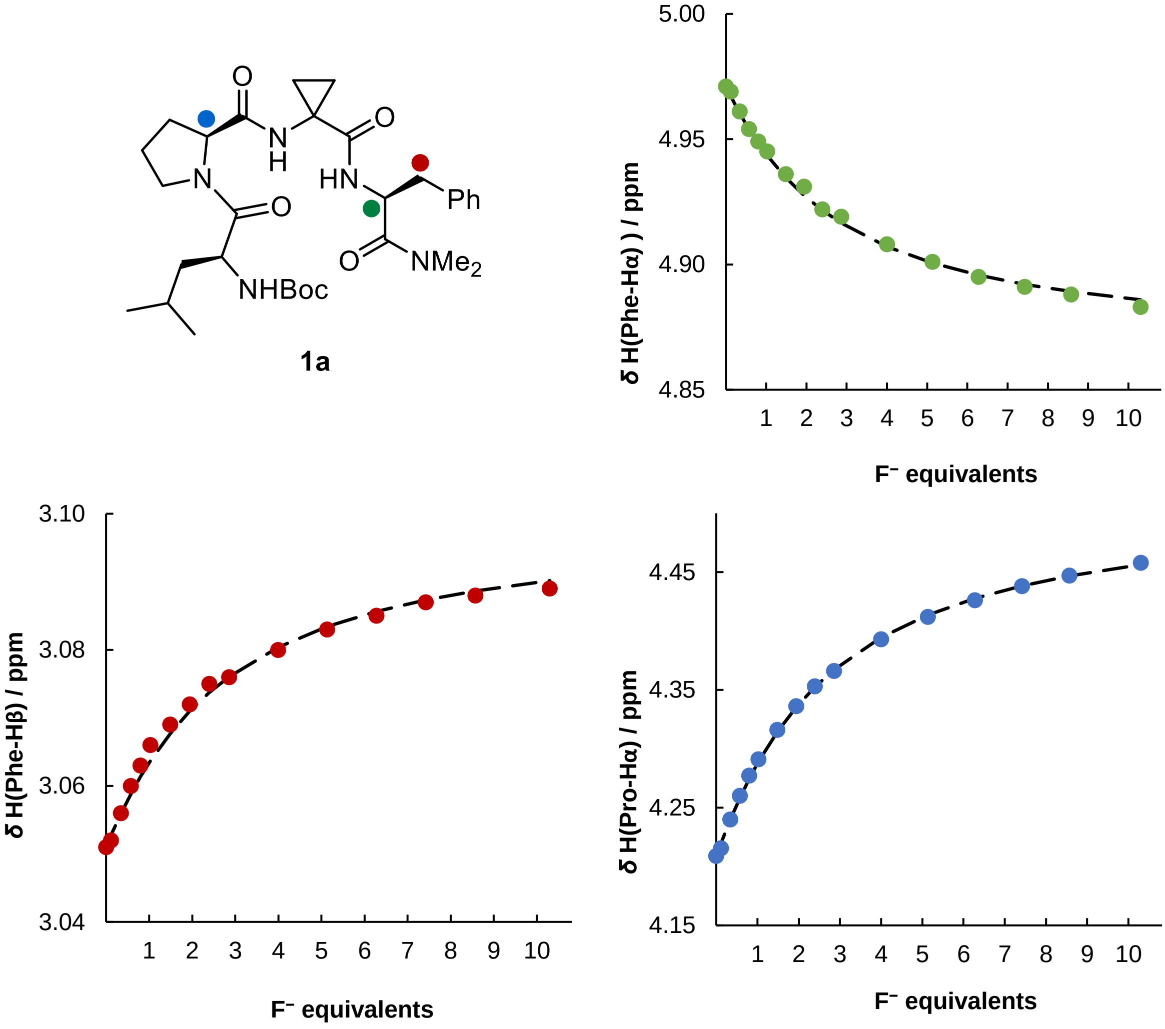

Figure S2. Titration profiles of selected protons for the titration of **1a** (2.8 mM) with TBAF·3H_2O in CD_2Cl_2 (144 mM) at 298 K; experimental points (colored circles) and fitted function (dashed lines).

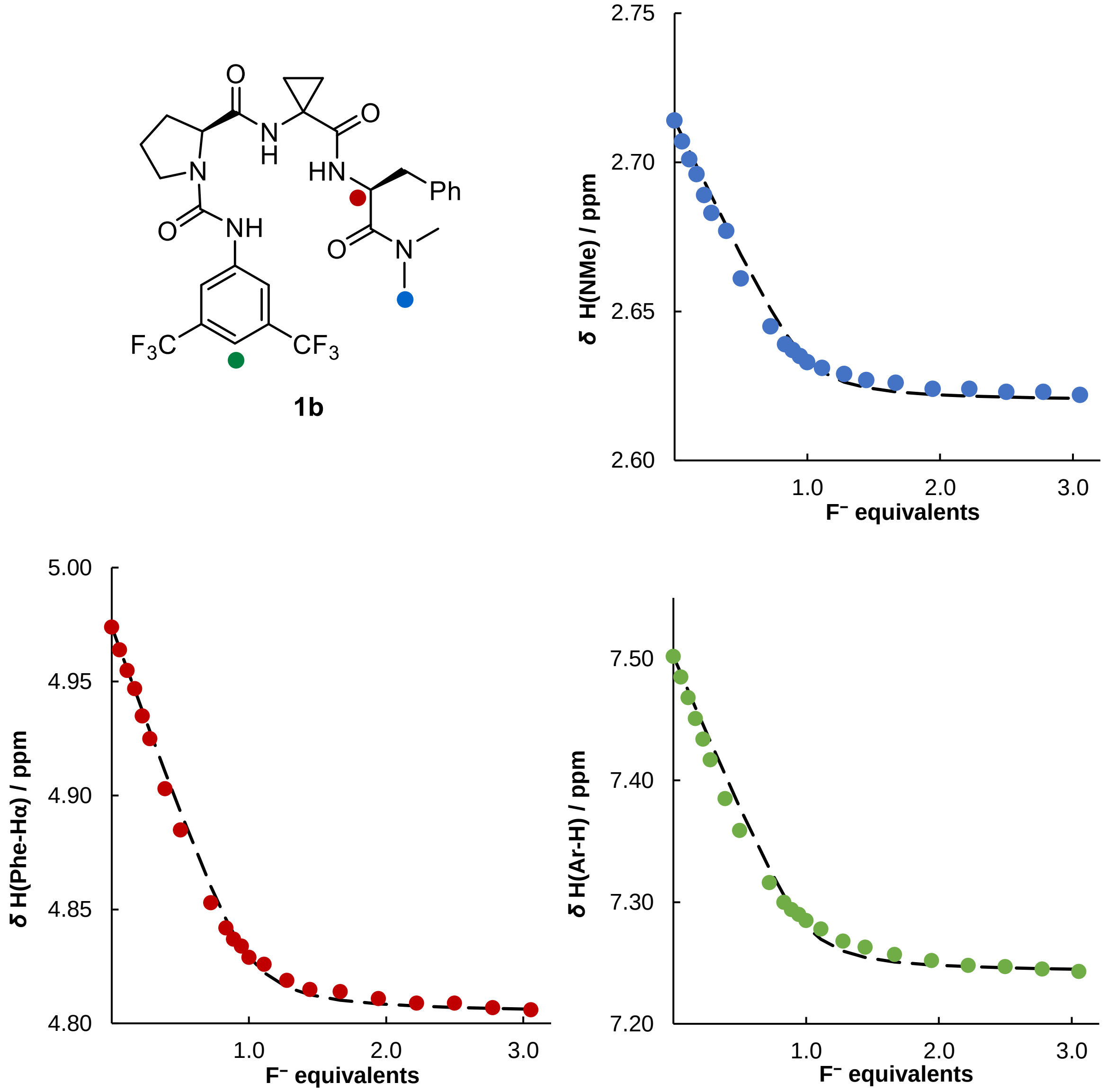

Figure S3. Titration profiles of selected protons for the titration of **1b** (5.1 mM) with TBAF·3H_2O in CD_2Cl_2 (142 mM) at 298 K; experimental points (colored circles) and fitted function (dashed lines).

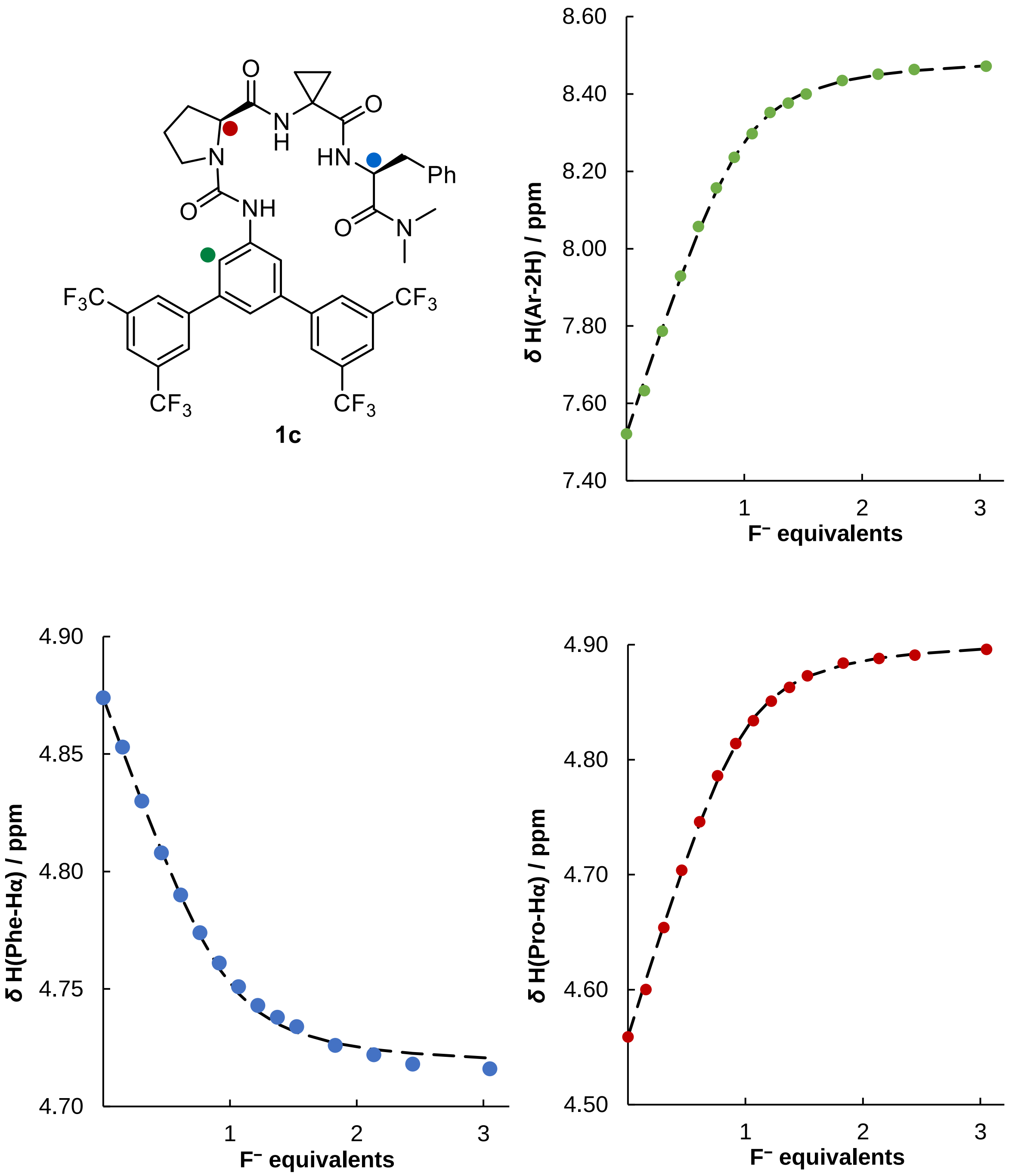

Figure S4. Titration profiles of selected protons for the titration of **1c** (1.6 mM) with TBAF·3H_2O in CD_2Cl_2 (108 mM) at 298 K; experimental points (colored circles) and fitted function (dashed lines).

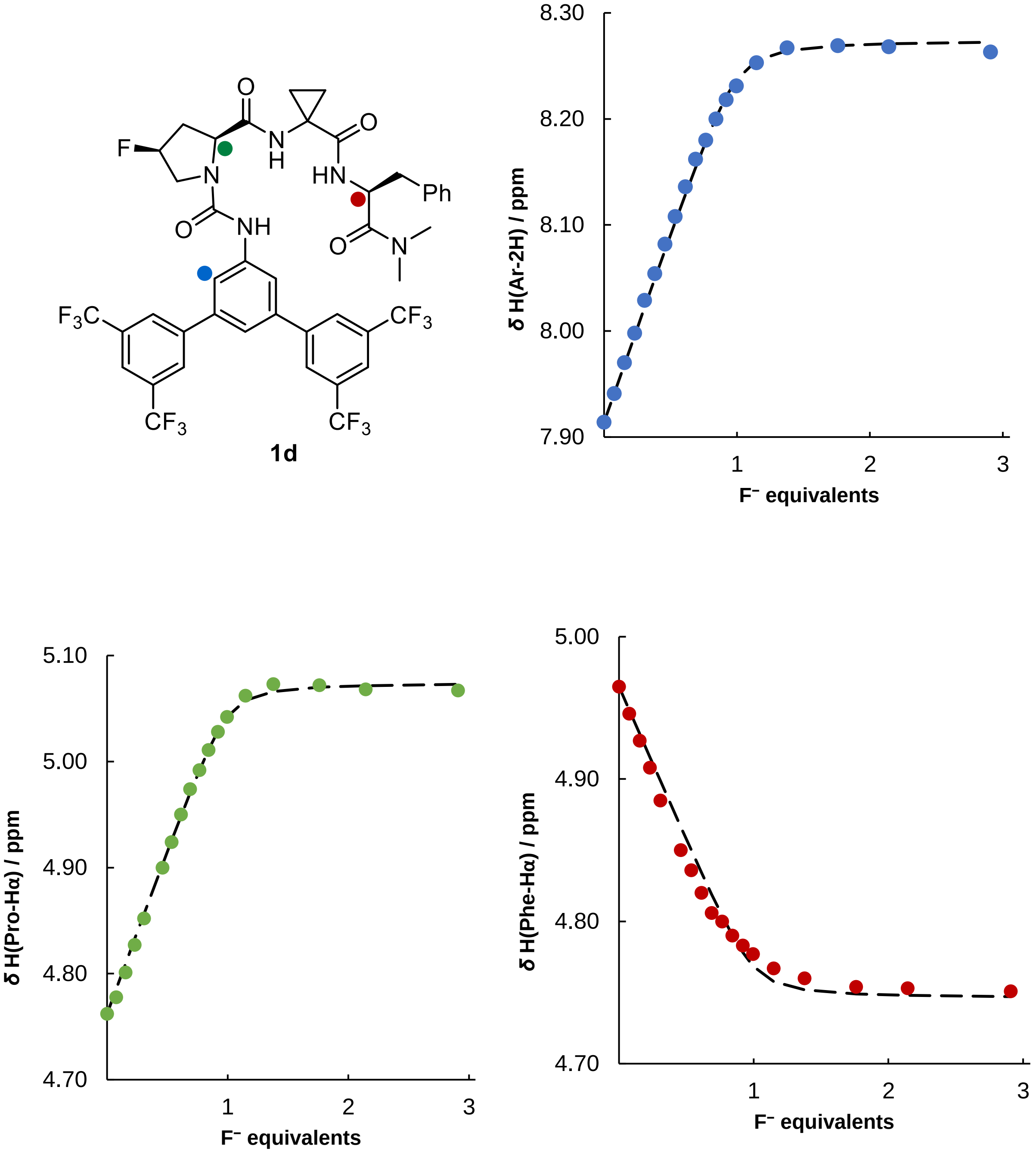

Figure S5. Titration profiles of selected protons for the titration of **1d** (3.9 mM) with TBAF·3H_2O in CD_2Cl_2 (134 mM) at 298 K; experimental points (colored circles) and fitted function (dashed lines).

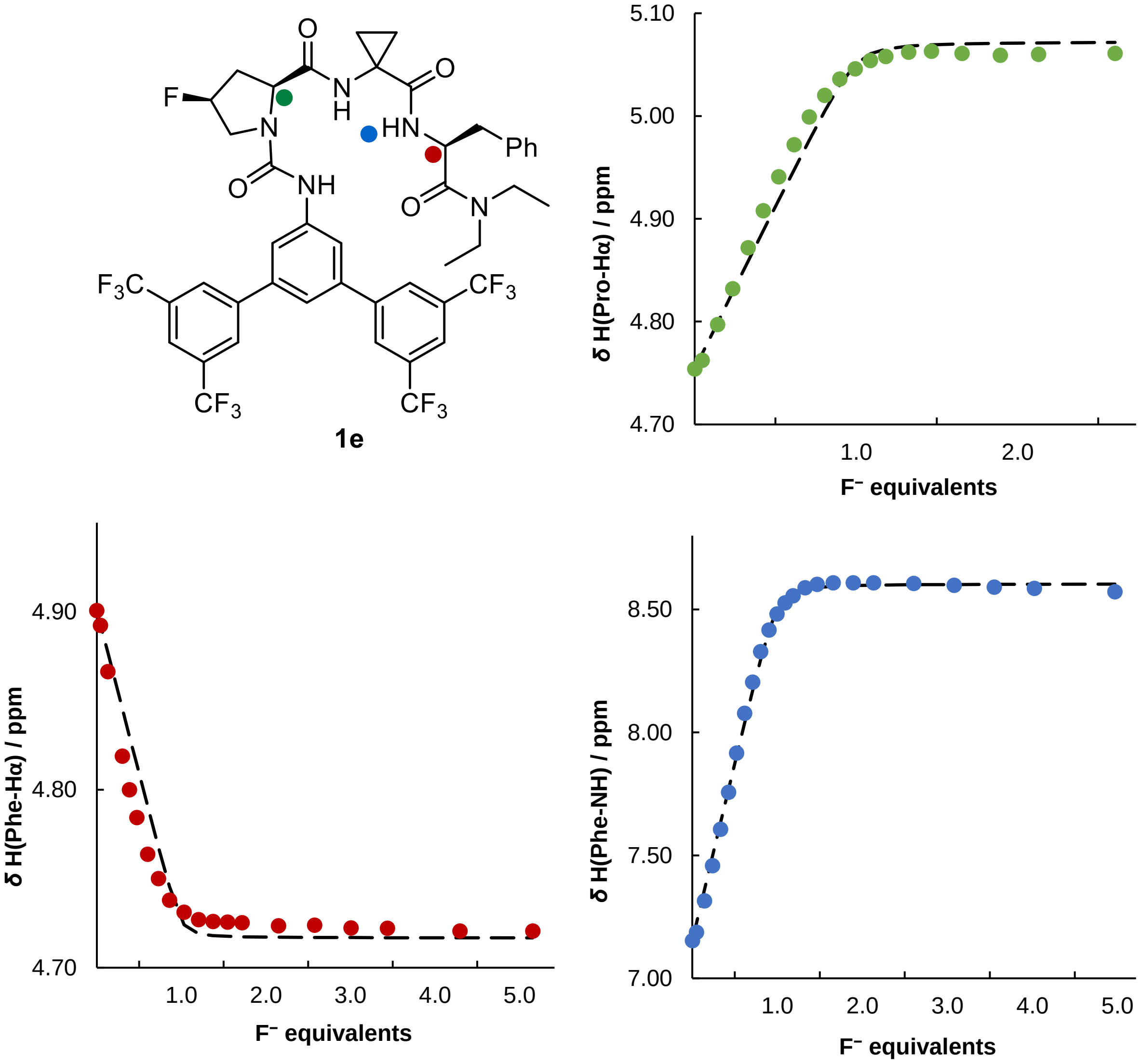

Figure S6. Titration profiles of selected protons for the titration of **1e** (5.9 mM) with TBAF·3H_2O in CD_2Cl_2 (154 mM) at 298 K; experimental points (colored circles) and fitted function (dashed lines).

6.4. **1e**–TBAF Complex: HOESY NMR

1D heteronuclear ^{19}F–^{1}H nOe experiments, utilizing ^{19}F inversion and ^{1}H detection, were acquired on AVIIHD 500 using a previously developed sequence (*hoesyfhgh1d*)[15] and the parameters reported below.

Parameter	Value
TD	32768 pt
SW	20 ppm
o2p	-110.85 ppm
NS (DS)	1280 (16)
relaxation delay d_1	3 s
mixing time d_8	600 ms

The ^{19}F – ^{1}H HOESY NMR experiment was acquired for **1e**:TBAF complex prepared using the titration sample preparation procedure, in absence of 4-fluoroanisole. Peak assignment was achieved during NMR titrations described above, by tracking chemical shifts.

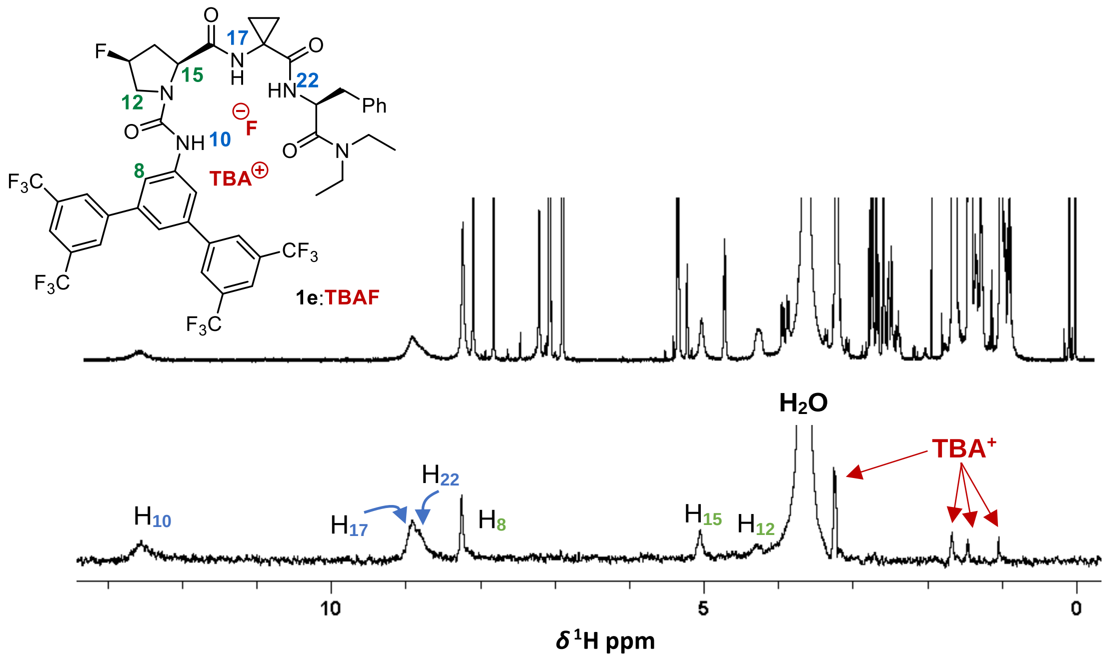

Figure S7. ^{1}H NMR (top) and ^{1}H-^{19}F HOESY (bottom) of **1e**:TBAF (3 mM + 1 equiv. of TBAF·$3H_2O$) at 298 K.

6.5. **1e**–CsF Complex: NMR at low temperature

Spectra were recorded in CD_2Cl_2 due to its noncoordinating nature, favorable melting point for low temperature experiments, and CH_2Cl_2 being a suitable reaction solvent (see **Table S4**). The concentration of all samples was set to 25 mM to reflect the reaction conditions applied to the fluorination reaction under HB-PTC.

Catalyst (**1e**, **1e-^{15}NH(*i*–1)**, **1e-^{15}NH(*i*+2)**) (0.0125 mmol) and CsF (100 mg, 0.66 mmol) were suspended in 0.5 mL of CD_2Cl_2 (25 mM) inside a sealed NMR tube. The sample was sonicated for 1 hour and NMR spectra were recorded on an AVIII HD 500 equipped with liquid nitrogen exchanger allowing for variable temperature studies. The samples can be stored at room temperature without signs of degradation.

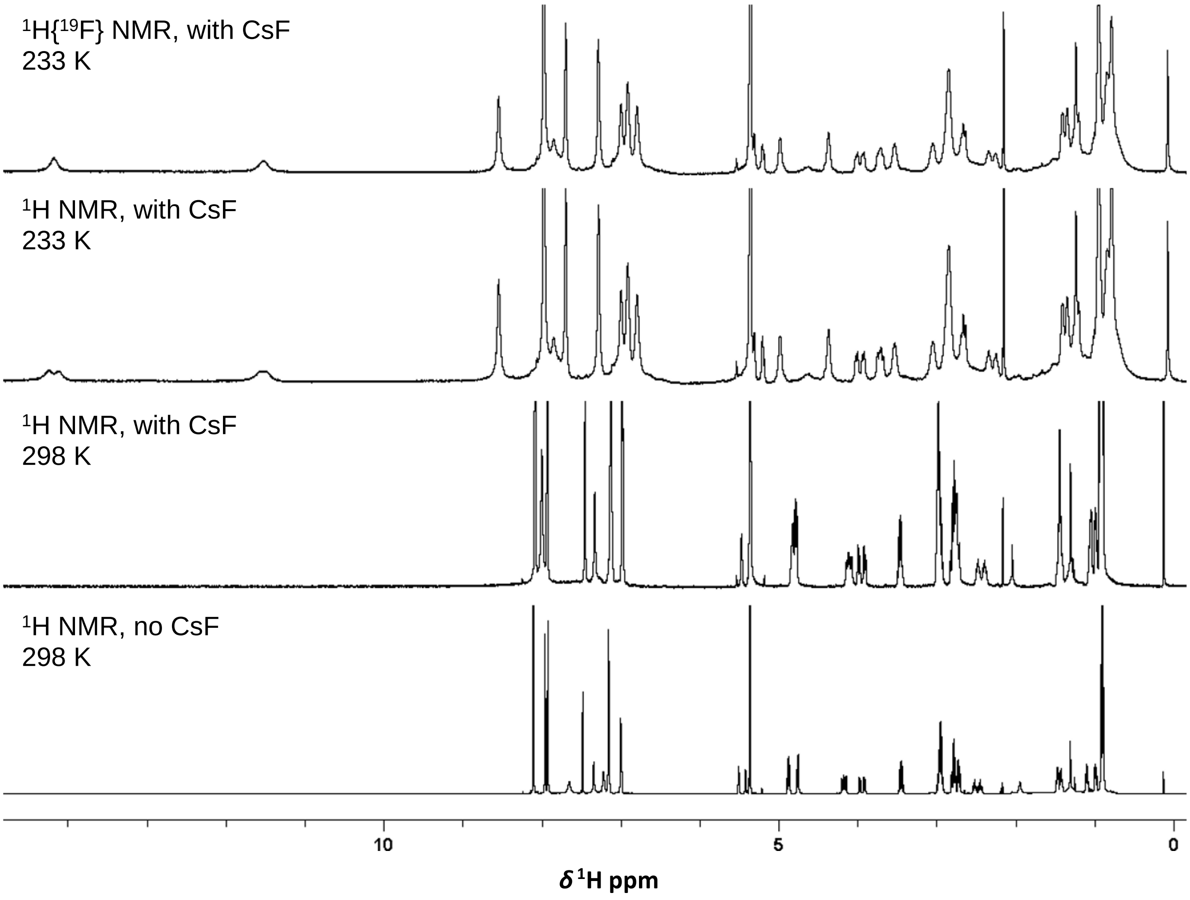

Figure S8. ^{1}H and ^{1}H{^{19}F} NMR of **1e** and **1e**:CsF at 298K and 233 K. For decoupling, ^{19}F δ (o2p) selected at -63 ppm.

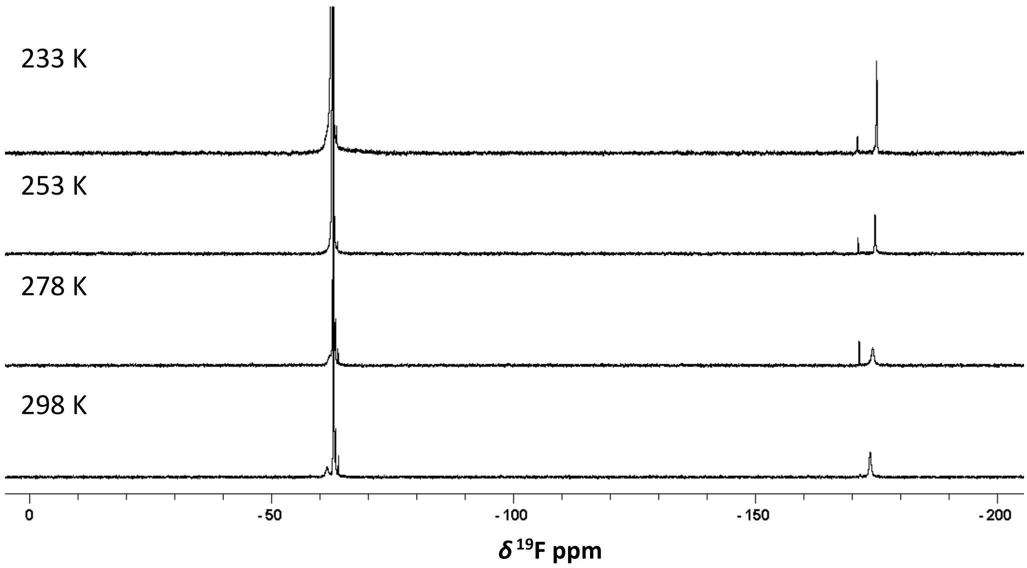

Figure S9. $^{19}F\{^{1}H\}$ NMR of **1e**:CsF 298–233 K.

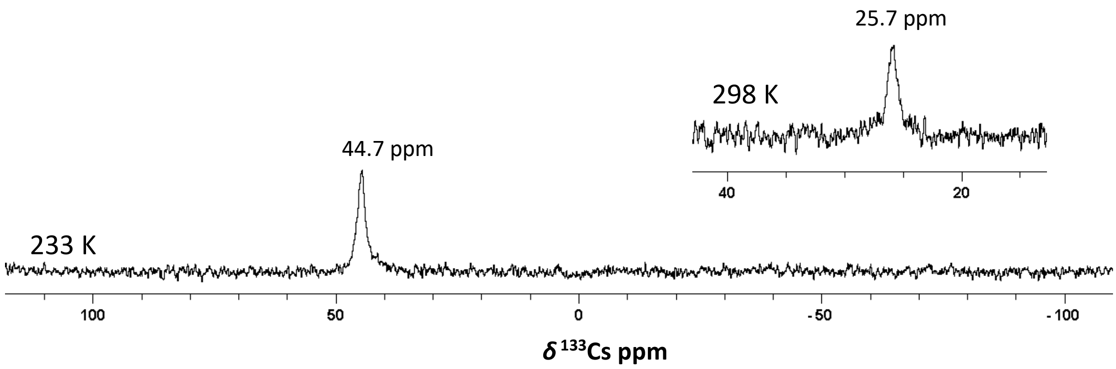

Figure S10. ^{133}Cs NMR of **1e**:CsF at 298 and 233 K.

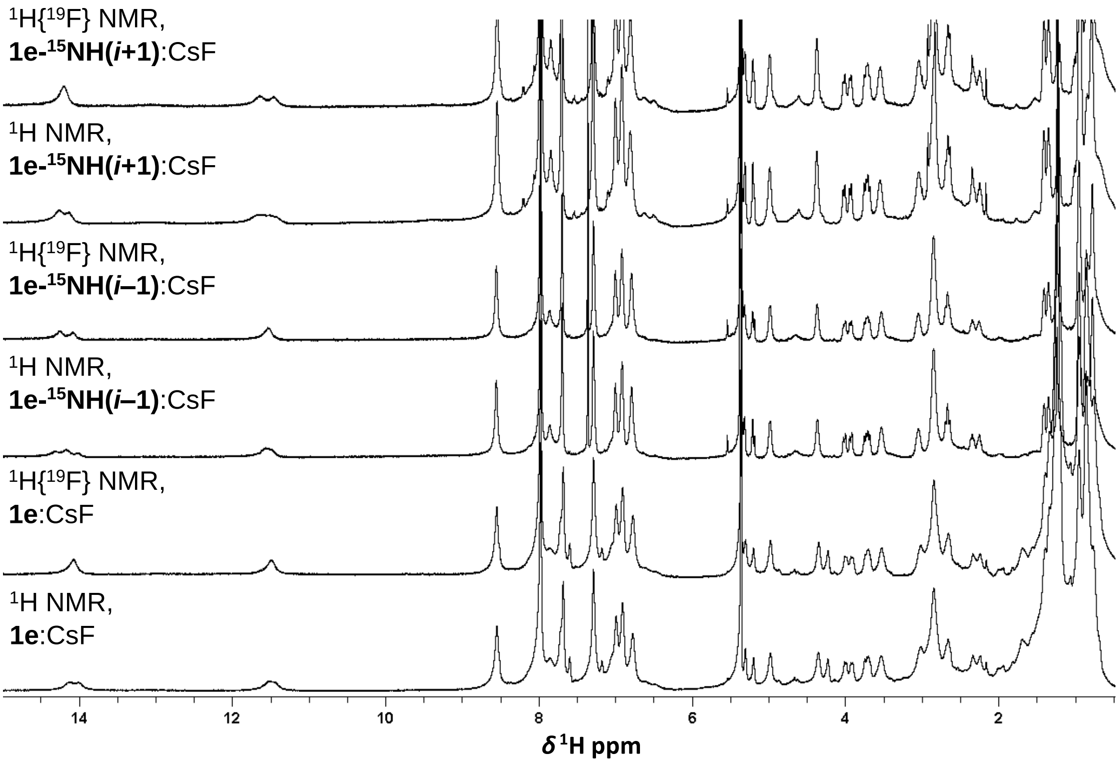

Figure S11. ^1H and $^1H\{^{19}F\}$ NMR of **1e, 1e-^{15}NH(*i*–1), 1e-^{15}NH(*i*+1)**:CsF at 233 K. For decoupling, ^{19}F δ (o2p) selected at −60 ppm.

6.6. CsF Complexation with **1a−1d**

Protocol for preparation of samples can be found in the section above. All samples can be stored at room temperature without signs of degradation.

When peptide **1a** (25 mM) was saturated with CsF in CD_2Cl_2, the ^{1}H spectrum broadened, indicating solubilization and complexation of CsF. While NH(*i*−1) and NH(*i*+2) shifts were unperturbed, the resonance of NH(*i*+1) disappeared at 298 K, but was observed upon cooling to 188 K as a broadened singlet (NH(*i*+1), 6.91 ppm; **Figure S12** *top*). At 188 K, additional low intensity peaks were observed in the ^{1}H spectrum, and by ^{1}H−^{15}N HSQC the presence of two sets of **1a**:CsF complexes was determined (**Figure S13**). Both complexes featured chelation by one NH at 12.72 ppm (minor species, $^{1h}J_{NH\cdots F^-}$ = 62 Hz) and 12.84 ppm (major species, $^{1h}J_{NH\cdots F^-}$ = 64 Hz) (**Figure S12** *bottom*). This data provides indirect evidence of NH(*i*+1) as the key HBD involved in fluoride complexation, in line with monodentate binding of TBAF·$3H_2O$ (*vide supra*). The ^{133}Cs NMR spectrum at 298 K exhibited a singlet at 37.5 ppm, which resolved into two doublets at 188 K at 56.9 and 50.5 ppm ($^1J_{Cs–F}$ = 104 and 102 Hz, respectively; **Figure S14**). This confirmed CsF solubilization in organic solvent medium and Cs–F through-bond scalar coupling as indicated by ^{19}F, ^{19}F{^{133}Cs}, and ^{133}Cs{^{19}F} NMR spectra (**Figure S14** and **S15**). The unusually sharp peaks in ^{133}Cs NMR spectra, considering its nuclear spin (I = 7/2), suggested a Cs^+ counterion in a locally ordered environment. These results serve as spectroscopic evidence of **1a** acting as a phase-transfer agent for CsF, but poor chelating ability for fluoride (monodentate binding). **1a**:CsF complex was further investigated computationally (GOAT-DFT; *section 7.3*). Conformation of the lowest energy conformer was in line with spectroscopic observations and, in addition, revealed a bidentate chelation of Cs^+ via C=O(*i*) and C=O(*i*+2) carbonyls (**Figure S26**).

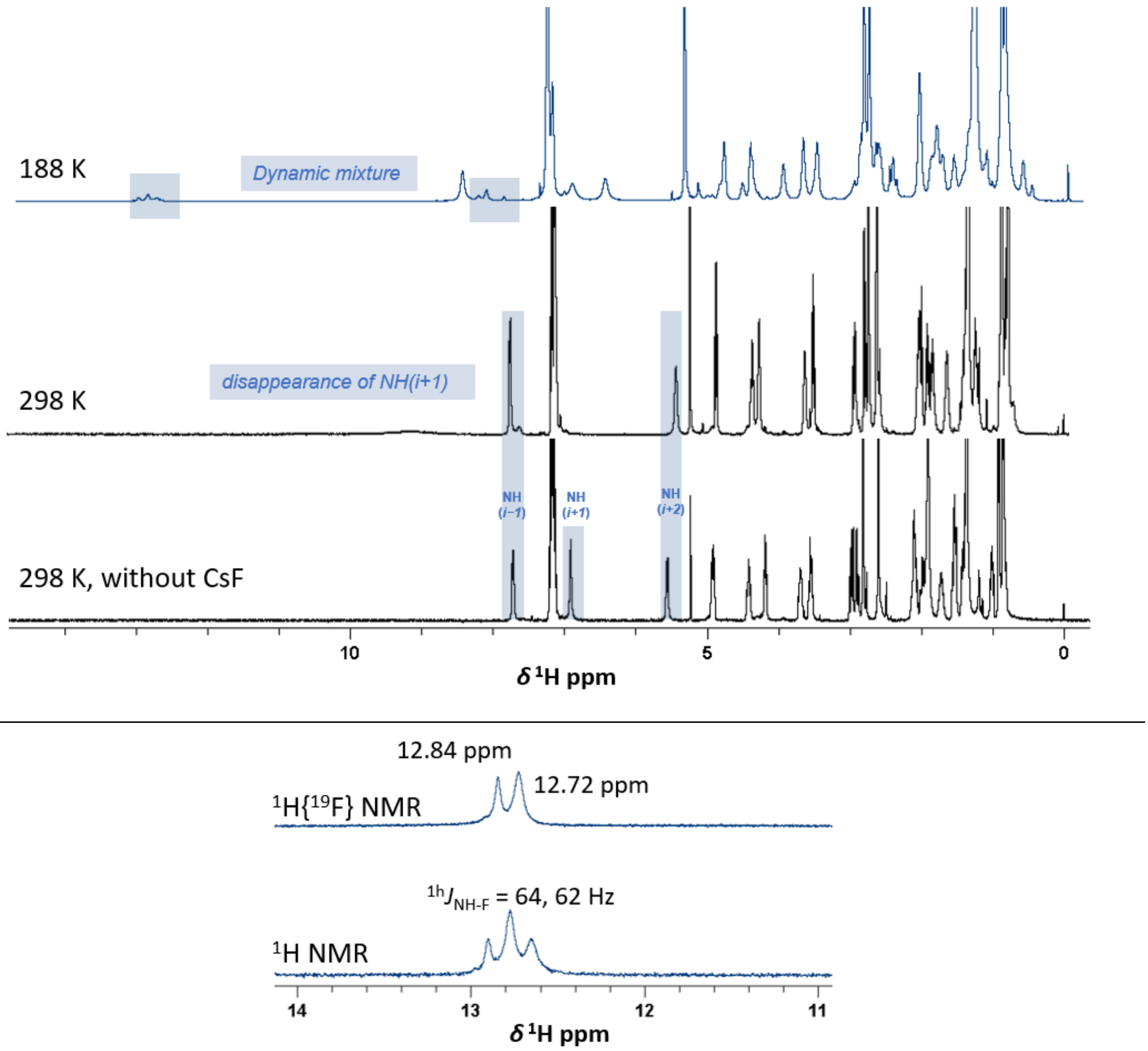

Figure S12. ^{1}H NMR of **1a** and **1a**:CsF at 298 K and 188 K (*top*); and ^{1}H{^{19}F} NMR 14–12 ppm expanded region of **1a**:CsF at 188 K (*bottom*). For decoupling, ^{19}F δ (o2p) selected at −80 ppm.

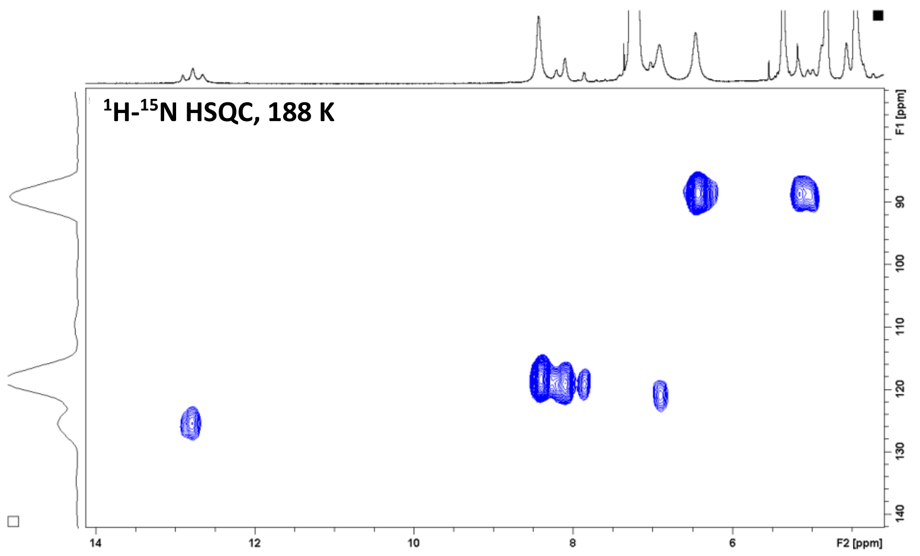

Figure S13. ^{1}H−^{15}N NMR spectrum of **1a** with CsF at 188 K. Adiabatic 180° on ^{15}N; NS = 800.

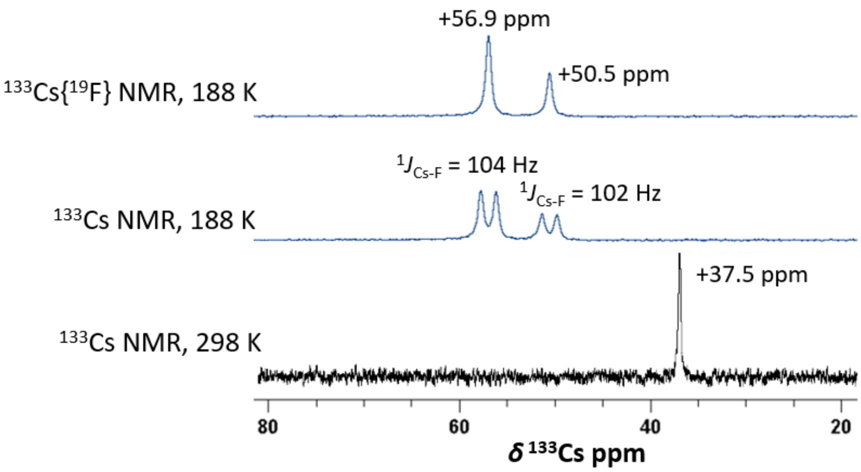

Figure S14. ^{133}Cs and ^{133}Cs{^{19}F} NMR of **1a**:CsF at 188 K and 298 K. ^{133}C{^{19}F} was achieved using GARP decoupling, ^{19}F δ (o2p) selected at −80 ppm.

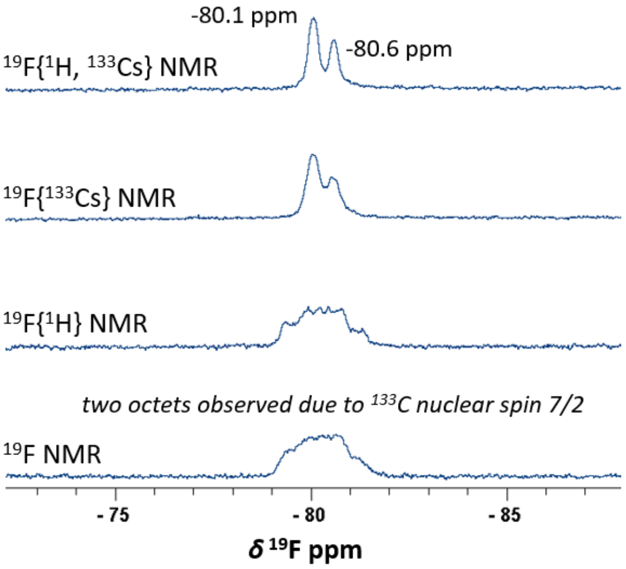

Figure S15. ^{19}F NMR with and without ^{1}H/^{133}Cs decoupling of **1a**:CsF at 188 K. For ^{133}Cs - CW decoupling (o2p = 53.6 ppm), for ^{1}H (o2p/o3p = 6.175 ppm) - CP decoupling.

The CsF binding studies for **1b** indicated tridentate binding at room temperature, as suggested by disappearance of all NH protons upon addition of CsF (**Figure S16**). At 298 K with CsF a broad peak appeared at 8.00 ppm – it was identified as H(4) *via* 2D ^{1}H-^{13}C HSQC experiment (**Figure S17**). At temperatures below 243 K, catalyst precipitates out of solution and, hence, is not observable via NMR.

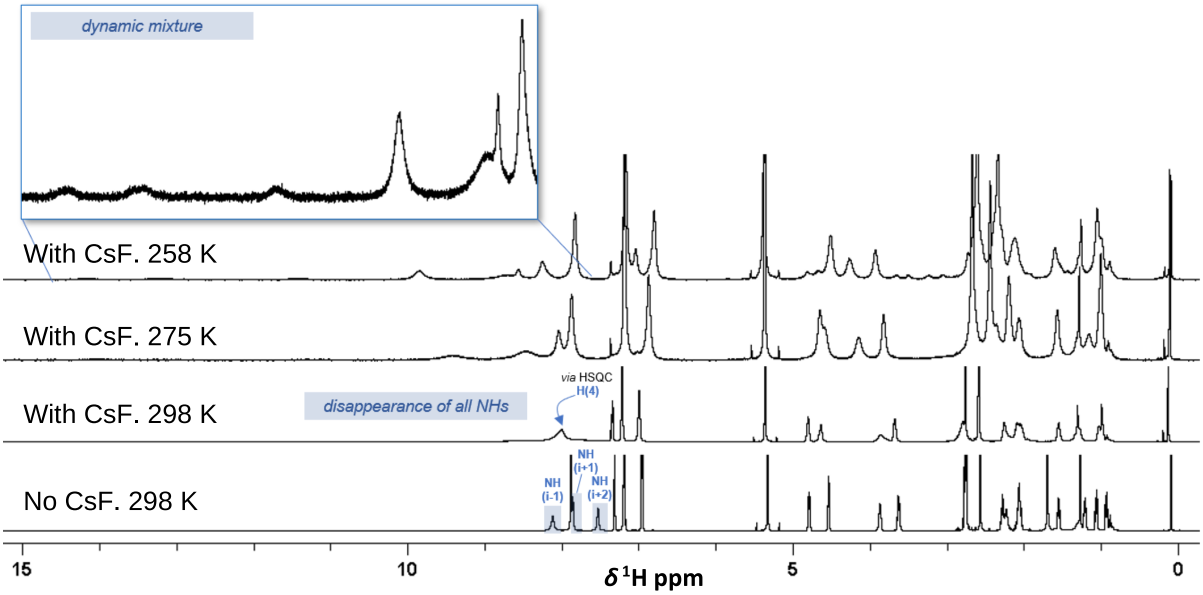

Figure S16. ^{1}H NMR of **1b** at 298 K, and **1b**:CsF at varying temperatures.

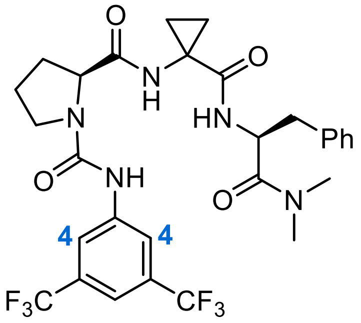

1b

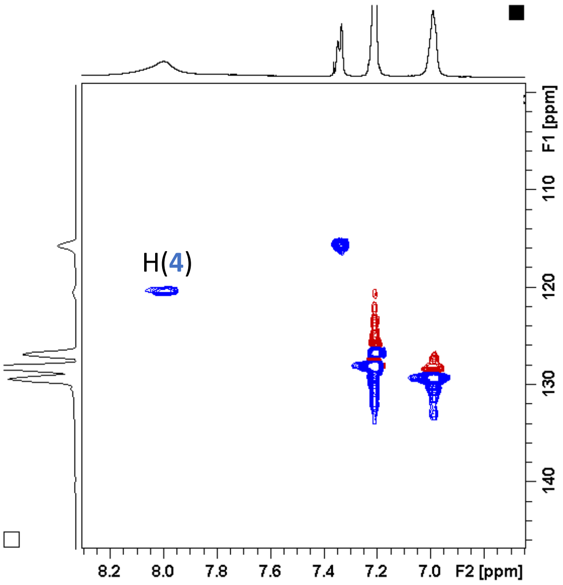

Figure S17. ^{1}H-^{13}C HSQC NMR spectrum expansion of **1b**:CsF at 298 K.

The CsF binding studies for **1c** indicated binding at room temperature (**Figure S18**). NMR experiments at temperatures below 273 K proved unsuccessful as catalyst precipitates out of solution.

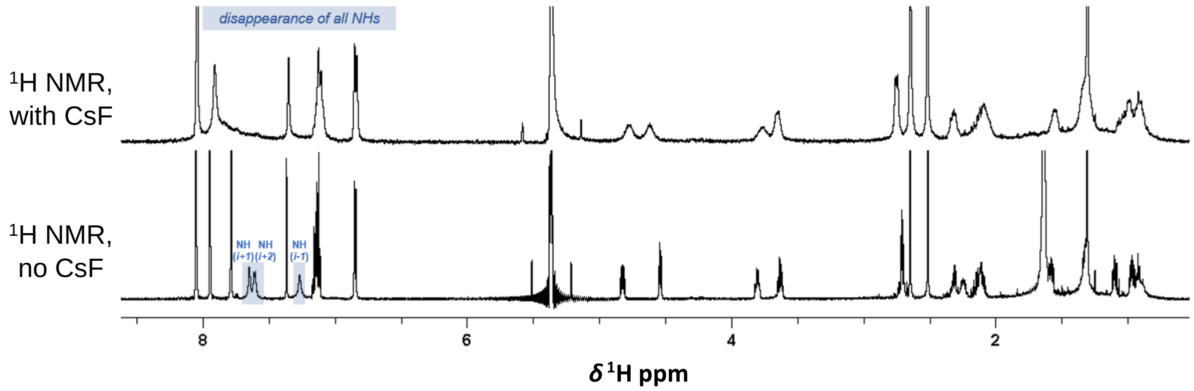

Figure S18. ^{1}H NMR spectra of **1c** with and without CsF at 298 K.

The CsF binding studies for **1d** indicated bidentate binding at room temperature (**Figure S19**). NMR experiments at 233 K showcased similar doublets as for **1e**:CsF complex.

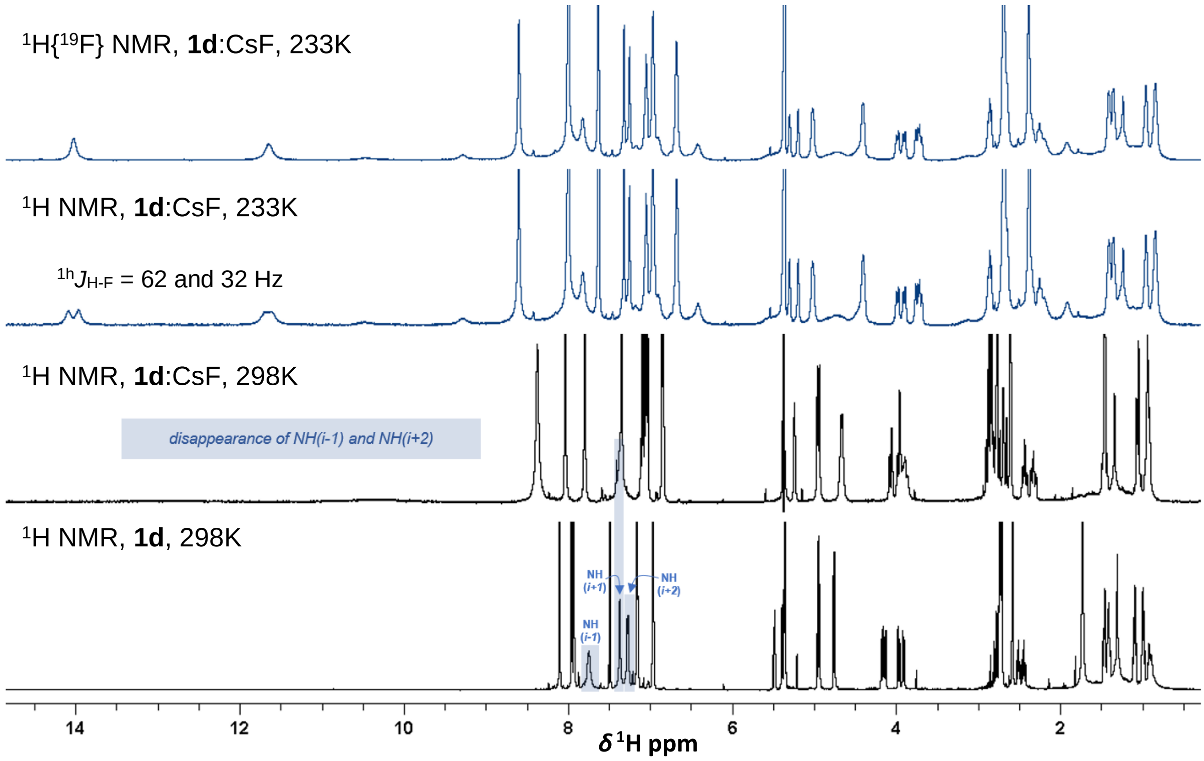

Figure S19. ^{1}H NMR of **1d** and **1d**:CsF at 298 K. And ^{1}H and $^{1}H\{^{19}F\}$ spectra of **1d**:CsF at 233 K. For decoupling, ^{19}F δ (o2p) selected at −60.9 ppm.

7. Computational studies

7.1. Computational methods

Geometry optimization and vibrational frequency calculations were performed using *Gaussian 16, revision C.01* at the M06-L-D3/def2-SVP;def2-TZVPPD[Cs^+, $F^–$] level of theory, where the double-zeta def2-SVP basis set was used for all atoms, except for Cs^+ and $F^–$ where the triple-zeta def2-TZVPPD basis set was used.[19] Solvation in dichloromethane was modeled using the universal solvation model (SMD).[20] Ground state geometries were identified by the absence of imaginary frequency vibrational modes. Single-point energy calculations were performed using *ORCA 6.0.0* software package at the M06-2X-D3/def2-TZVP; def2-TZVPPD[Cs^+, $F^–$] level of theory, where the triple-zeta def2-TZVP basis set was used for all atoms except for Cs^+ and $F^–$ where the triple-zeta def2-TZVPPD was applied.[21] All bond lengths are reported in Angstroms (Å). Thermochemistry was evaluated at 298.15 K and a concentration of 0.25 mol·$L^{–1}$ using Paton's *GoodVibes* script.[22] The entropic contribution of low vibrational modes was corrected using a frequency cutoff value of 100 $cm^{–1}$.[23]

Conformational sampling was performed prior to geometry optimization of key structures using a Global Optimizer Algorithm (GOAT) featured in *ORCA 6.0.0*, based on the GFN2-XTB method.[24] Conformational sampling was performed in dichloromethane using the extended conductor-like polarizable continuum model (CPCM-X).[25] A 6 kcal·$mol^{–1}$ energy window was applied during sampling.

7.2. Binding modes of **1d:CsF**

A total of 487 unique conformers of the **1d:CsF** complex were identified after optimization, ranging from tridentate H-bonding mode to no chelation of fluoride. Analysis of the NH···F⁻ bond distance revealed a large fraction of geometries with NH···F⁻ ≤ 2.0 Å, consistent with effective hydrogen bonding interactions (**Figure S20**).

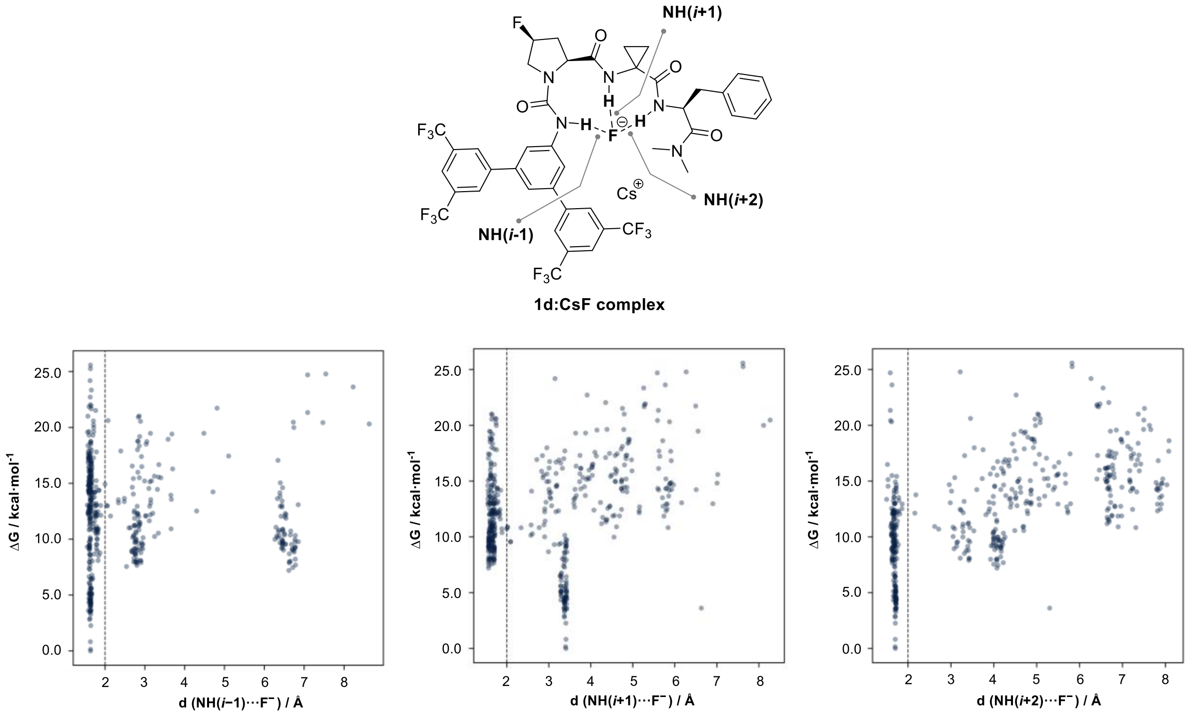

Figure S20. Relative Gibbs free energy plotted against NH–F⁻ bond length.

Conformers were further grouped into eight classes (**A–H**) based on the number of H-bonding between **1d** and F⁻, using an NH···F⁻ ≤ 2.0 Å criterion (**Figure S21**). **Class C** in which NH(*i*–1) and NH(*i*+2) bind to fluoride, was the most stable ensemble, followed by the other two bidentate binding modes (**Class D** and **Class B**). The tridentate mode (**Class A**) was ranked the fifth, whereas structures lacking of fluoride chelation formed the least stable ensemble (**Class H**).

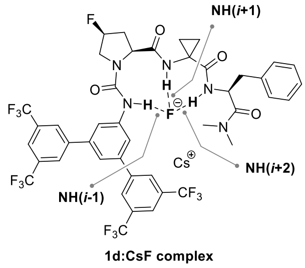

Class	NH(*i*-1)	NH(*i*+1)	NH(*i*+2)
A	✓	✓	✓
B	✓	✓	X
C	✓	X	✓
D	X	✓	✓
E	✓	X	X
F	X	✓	X
G	X	X	✓
H	X	X	X

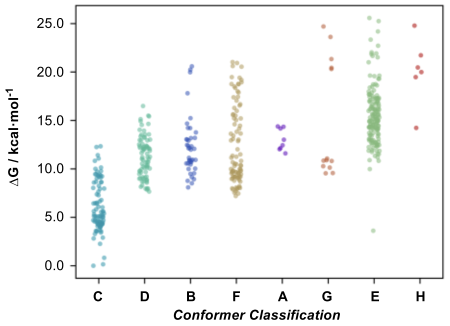

Figure S21. Conformer classification of **1d:CsF** complex.

The structure with the lowest global Gibbs free energy (conformer in **Class C**) features bidentate H-bonding to fluoride with NH(i−1)···F$^-$ (1.64 Å) < NH(i+2)···F$^-$ (1.71 Å), bidentate chelation to cesium via two carbonyls (2.98 and 3.01 Å) and a proximal Cs$^+$···F$^-$ ion pairing (2.82 Å) (**Figure S22**). In contrast, maintaining tridentate binding in **Class A** requires Cs$^+$ and F$^-$ to separate (4.73 Å), which reduces stability.

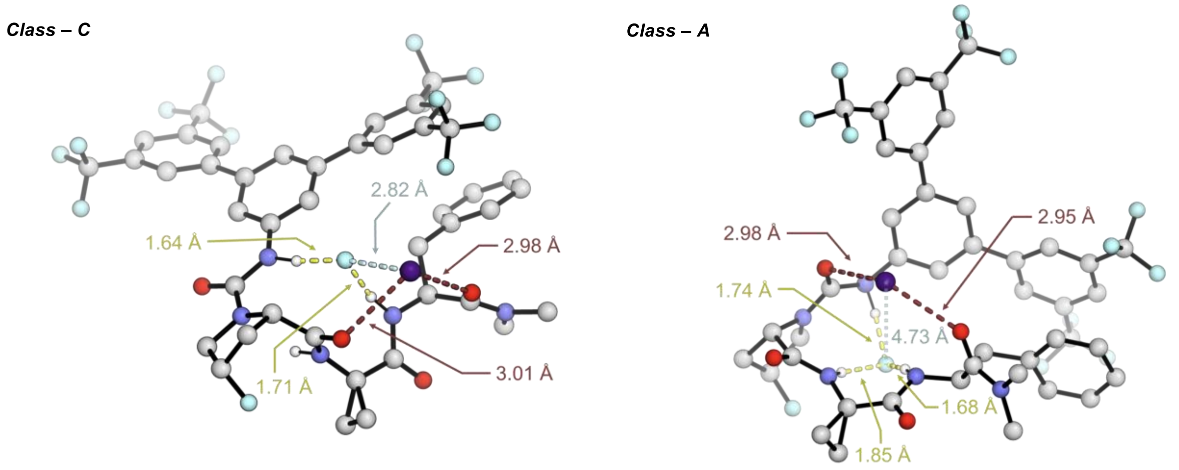

**1d:CsF (C)**	**1d:CsF (A)**
ΔΔG = 0.0 kcal/mol	ΔΔG = 11.6 kcal/mol

Figure S22. Structures with the lowest Gibbs free energies in **Class C** and **A**.

7.3. Binding modes of **1a:CsF**

A total of 351 unique conformers of the **1a:CsF** complex were identified after optimization, ranging from bidentate H-bonding mode to no chelation of fluoride. Analysis of the NH···F⁻ bond distance revealed a large fraction of geometries with NH···F⁻ ≤ 2.0 Å, consistent with effective hydrogen bonding interactions (**Figure S23**).

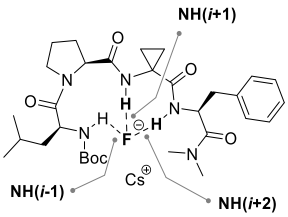

1a:CsF complex

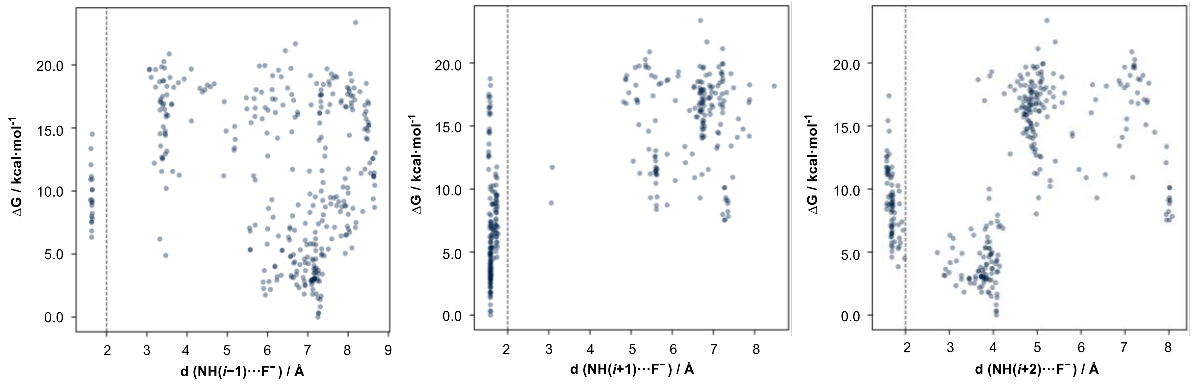

Figure S23. Relative Gibbs free energy plotted against NH–F⁻ bond length.

Conformers were further categorized based on the number of H-bonding between **1a** and F⁻, using an NH···F⁻ ≤ 2.0 Å criterion (**Figure S24**). **Class F** in which only NH(*i*+1) binds to fluoride, was the most stable ensemble. **Class A**, the tridentate H-bonding mode and **Class C**, in which NH(*i*–1) and NH(*i*+2) bind to fluoride, were not observed. A closer inspection of **Class F** revealed that NH(*i*+2) forms intramolecular H-bond with C=O^{A}(*i*−1) in the lower energy conformers (**Figure S25**).

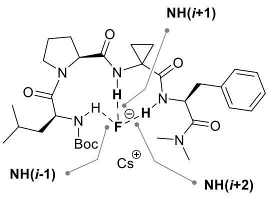

1a:CsF complex

Class	NH(*i*-1)	NH(*i*+1)	NH(*i*+2)
A	√	√	√
B	√	√	X
C	√	X	√
D	X	√	√
E	√	X	X
F	X	√	X
G	X	X	√
H	X	X	X

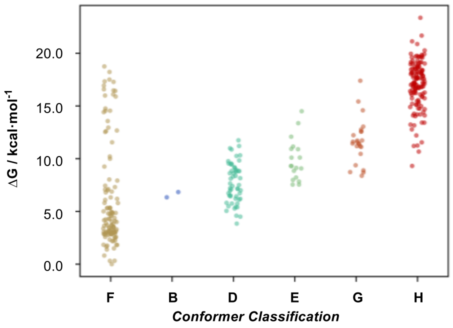

Figure S24. Conformer classification of **1a:CsF** complex.

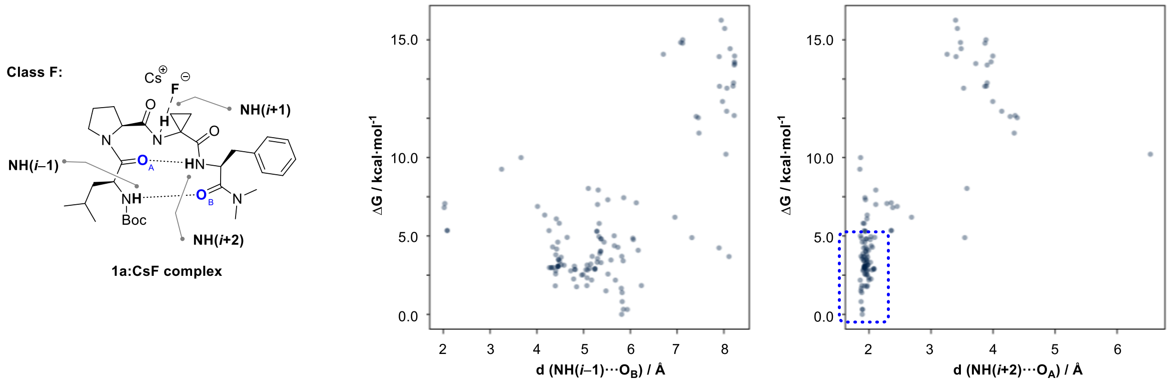

Figure S25. Relative Gibbs free energy plotted against NH···O$^{A/B}$ bond lengths in **Class F**. Effective hydrogen bonding interaction between NH(i+2) and C=O^{A}(i−1) for lower energy conformers in **Class F** (ΔG ≤ 5 kcal·mol^{-1}) were observed.

The structure with the lowest global Gibbs free energy (conformer in **Class F**) for complex **1a:CsF** features monodentate H-bonding to fluoride with NH(i+1)···F^{-} (1.58 Å), bidentate chelation to cesium via two carbonyls (3.02 and 3.04 Å), a proximal Cs^{+}···F^{-} ion pairing (2.82 Å), and intramolecular hydrogen-bonding between NH(i+2) and C=O(i−1) (**Figure S26**).

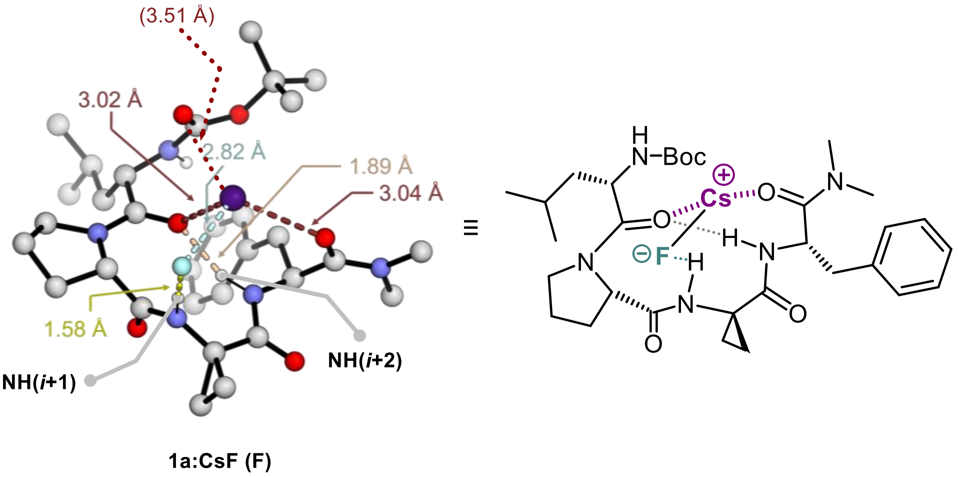

Figure S26. Structure with the lowest Gibbs free energy for complex **1a:CsF** (conformer in **Class F**).

7.4. Cartesian coordinates of **1d:CsF (C)**, **1d:CsF (A)**, and **1a:CsF (F)**

1d:CsF (C)

C -1.400289 -1.498300 0.564901

C -2.775794 -1.598658 0.306953

C -3.581663 -0.447298 0.322726

C -3.015000 0.806929 0.590454

C -1.642167 0.919687 0.856527

C -0.857382 -0.233533 0.842530

H -3.201754 -2.569900 0.065656

H -3.653908 1.691527 0.639524

H 0.219111 -0.187115 1.014441

C -0.994868 2.220786 1.128214

C -1.524463 3.422596 0.639171

C 0.215336 2.280772 1.837234

C -0.858031 4.632228 0.837058

H -2.451999 3.413053 0.062265

C 0.881894 3.488164 2.020944

H 0.651044 1.368326 2.249615

C 0.354014 4.678926 1.522626

H 0.885171 5.622185 1.654676

C -5.032958 -0.566021 0.060721

C -5.753528 0.487618 -0.521840

C -5.735761 -1.735553 0.386190

C -7.121187 0.374246 -0.765033

H -5.237653 1.405971 -0.812513

C -7.103903 -1.842769 0.141321

H -5.213328 -2.571798 0.856776

C -7.812127 -0.790706 -0.435960

H -8.881587 -0.877579 -0.627414

C -1.474481 5.890951 0.302356

C 2.176712 3.487695 2.777606

C -7.837962 1.544730 -1.369965

C -7.795619 -3.117321 0.525031

F -2.484038 6.318155 1.085685

F -0.596410 6.900066 0.216823

F -1.997206 5.713404 -0.923208

F 2.914684 4.578397 2.534260

F 1.982771 3.432841 4.108794

F 2.939580 2.417498 2.467846

F -7.198340 2.016648 -2.454355

F -7.922365 2.573406 -0.504699

F -9.088042 1.246125 -1.745471

F -9.102674 -3.102406 0.233321

F -7.687086 -3.363784 1.843531

F -7.261724 -4.180811 -0.102275

H 0.490656 -2.237865 0.735913

N -0.491055 -2.552722 0.563320

C -0.750668 -3.868204 0.264564

O -1.843013 -4.310733 -0.073830

C 1.606053 -4.445593 1.051071

C 0.151162 -6.126877 0.094375

C 1.971759 -5.829387 1.590266

H 1.462403 -3.719469 1.865362

C 1.462179 -6.749116 0.507915

H -0.082650 -6.337761 -0.959521

H -0.681576 -6.522280 0.698347

H 3.044178 -5.946947 1.786873

H 1.424843 -6.029993 2.521974

H 1.372467 -7.803700 0.802021

N 0.350249 -4.700908 0.343041

F 2.357061 -6.693925 -0.575954

C 2.740240 -3.889629 0.186324

O 3.857521 -3.726062 0.668180

H 1.478988 -3.853532 -1.397698

N 2.426135 -3.635375 -1.101989

C 3.529500 -1.757508 -2.357780

O 4.212363 -1.356403 -3.301621

H 2.441780 -1.221803 -0.636713

N 2.881015 -0.912815 -1.526891

C 2.981741 0.506145 -1.793455

H 2.651997 0.683928 -2.827419

C 4.420475 0.995487 -1.595396

O 5.008374 0.709510 -0.547349

N 4.958924 1.803784 -2.537661

C 6.226130 2.452166 -2.285003
H 6.137633 3.536896 -2.448925
H 6.530251 2.270558 -1.250091
H 7.012138 2.070002 -2.954811
C 4.337212 2.069077 -3.821404
H 3.482178 2.760385 -3.743739
H 5.079660 2.533771 -4.479925
H 4.006964 1.139093 -4.301271
C 2.054599 1.277396 -0.848149
H 1.051063 0.832837 -0.931615
H 2.394323 1.102422 0.186118
C 1.994039 2.748185 -1.145256
C 2.986651 3.622248 -0.677070
C 0.957979 3.276533 -1.926921
C 2.946364 4.981709 -0.983513
H 3.798825 3.228038 -0.057788
C 0.913939 4.636426 -2.236246
H 0.171522 2.607734 -2.292584
C 1.908948 5.493610 -1.765213
H 3.724827 5.647326 -0.600497
H 0.094218 5.029438 -2.842927
H 1.870923 6.560534 -1.999247
F 1.900352 -1.436049 0.966050
Cs 4.557484 -1.034993 1.829748
C 3.361568 -3.229234 -2.098284
C 3.483052 -4.126497 -3.305764
C 4.580200 -4.091319 -2.311998
H 3.631670 -3.626110 -4.264165
H 2.824372 -4.998061 -3.316414
H 4.697173 -4.933838 -1.628242
H 5.501196 -3.566717 -2.574059

1d:CsF (A)

C 0.786488 0.801393 -2.022645
C 2.130780 0.755141 -1.626723
C 2.691314 -0.440228 -1.157790
C 1.910421 -1.603095 -1.115214
C 0.571403 -1.580391 -1.531408
C 0.023988 -0.373759 -1.983344
H 2.742386 1.652902 -1.695267
H 2.348537 -2.530919 -0.741437
H -1.028999 -0.314244 -2.267501
C -0.279022 -2.785160 -1.429516
C -1.407226 -2.950658 -2.246033
C -0.013741 -3.773132 -0.470587
C -2.238140 -4.059975 -2.101468
H -1.642800 -2.206496 -3.010070
C -0.848346 -4.879513 -0.331574
H 0.833919 -3.660053 0.208898
C -1.968872 -5.036479 -1.144569
H -2.631423 -5.893811 -1.022412
C 4.083109 -0.452740 -0.661312
C 4.616817 0.676785 -0.025141
C 4.896623 -1.588741 -0.775509
C 5.913695 0.667714 0.484934
H 4.001545 1.572002 0.098137
C 6.195571 -1.590196 -0.268680
H 4.517204 -2.480071 -1.281520
C 6.717320 -0.464897 0.368125
H 7.731153 -0.471404 0.768345
C -3.426307 -4.190103 -3.008362
C -0.528706 -5.890467 0.729055
C 6.405016 1.895622 1.193023
C 7.026466 -2.827723 -0.443007
F -4.087416 -3.026525 -3.137086
F -3.064601 -4.557903 -4.252244
F -4.307522 -5.101026 -2.573131
F -0.162032 -5.301961 1.883040
F -1.565411 -6.691812 1.006233
F 0.496613 -6.685609 0.368466
F 7.687981 1.804547 1.557506
F 5.691316 2.135802 2.313170
F 6.281859 2.994788 0.428343
F 6.389470 -3.920253 0.014916
F 8.199256 -2.755113 0.198290
F 7.298078 -3.064181 -1.739218
H -0.863830 1.874511 -2.572693

N 0.164774 1.973070 -2.451378

C 0.443282 3.204349 -1.879345

O 1.244256 3.386542 -0.959653

C -0.389857 5.458959 -1.604546

C -1.091188 4.248866 -3.603417

C -1.108935 6.409808 -2.566302

H 0.613520 5.829287 -1.350978

C -1.918970 5.508978 -3.472760

H -1.740381 3.368926 -3.708157

H -0.430823 4.303400 -4.485096

H -1.730887 7.161204 -2.063236

H -0.368016 6.949148 -3.172977

H -2.163825 5.962071 -4.443561

N -0.312163 4.224997 -2.368933

F -3.138029 5.201502 -2.851384

C -1.113713 5.286858 -0.260649

O -0.849398 6.044864 0.664587

H -2.174348 3.698628 -1.054803

N -2.046697 4.298463 -0.225218

C -3.155552 2.804431 1.473686

O -3.724242 2.690770 2.558589

H -2.513793 1.815627 -0.228714

N -2.671467 1.726745 0.800355

C -3.102174 0.426427 1.287033

H -4.203757 0.393978 1.288922

C -2.555447 0.175839 2.694212

O -1.348987 0.338588 2.905523

N -3.396815 -0.325574 3.627785

C -4.833980 -0.413681 3.450416

H -5.292005 -0.647284 4.417970

H -5.252063 0.541876 3.107715

H -5.125905 -1.207311 2.744228

C -2.871049 -0.763629 4.900924

H -3.220456 -1.782494 5.126256

H -1.777718 -0.760368 4.866523

H -3.197749 -0.103001 5.719417

C -2.597437 -0.687788 0.362464

H -2.866767 -0.421277 -0.671960

H -1.495557 -0.711688 0.415360

C -3.183887 -2.024183 0.721620

C -2.602957 -2.832977 1.708456

C -4.373545 -2.458643 0.120523

C -3.198213 -4.035779 2.089372

H -1.668253 -2.513733 2.183045

C -4.963891 -3.667858 0.487063

H -4.837652 -1.836888 -0.651216

C -4.379223 -4.458553 1.477662

H -2.727884 -4.654814 2.857855

H -5.882423 -3.995484 -0.006954

H -4.838525 -5.407051 1.767853

F -2.445973 2.071952 -1.883719

Cs 0.688490 2.081685 1.664310

C -3.047010 4.141480 0.787294

C -4.422357 4.744303 0.508124

C -3.544820 5.328521 1.549570

H -5.294397 4.160644 0.811955

H -4.517725 5.264924 -0.446841

H -3.055678 6.280413 1.350378

H -3.796690 5.137486 2.594259

1a:CsF (F)

C 2.971496 3.238972 1.062086

C 0.553846 3.296962 0.819529

C 1.115603 4.709383 0.930885

C 2.417878 4.506522 1.687199

H 3.651926 2.679293 1.719815

H 3.520261 3.473998 0.133953

H -0.021984 3.038397 1.726930

H 1.319303 5.099790 -0.079582

H 0.413275 5.394814 1.422097

H 3.112713 5.352043 1.603578

H 2.216573 4.345318 2.757763

C -0.326033 3.077136 -0.410658

O 0.143502 3.048707 -1.542695

H -1.931616 2.944553 0.908168

C -2.640619 2.681029 -1.082436

C -3.900347 3.499497 -0.989382

C -2.952691 3.749641 -2.102663
H -3.944106 4.216658 -0.167211
H -4.831220 2.975763 -1.213862
H -3.225709 3.405272 -3.102490
H -2.322097 4.639895 -2.069882
N -1.635203 2.932878 -0.103529
C -2.819149 1.263861 -1.557514
O -3.793516 0.922340 -2.225731
H -1.004799 0.687609 -0.718783
C -1.940090 -0.982172 -1.665912
H -2.210193 -0.964864 -2.733384
N -1.844047 0.387329 -1.216616
C -0.626381 -1.736525 -1.483494
H -0.272172 -1.586601 -0.447913
H -0.837837 -2.816079 -1.555280
C 0.487967 -1.407229 -2.439906
C 0.500965 -0.258145 -3.241238
C 1.574344 -2.293448 -2.538665
C 1.561232 -0.007634 -4.115308
H -0.320097 0.460003 -3.185693
C 2.634769 -2.043733 -3.407640
H 1.578624 -3.200002 -1.923902
C 2.630186 -0.897422 -4.205347
H 1.546206 0.895384 -4.730931
H 3.465885 -2.751916 -3.468115
H 3.455019 -0.701506 -4.894823
C -3.028487 -1.710730 -0.867508
O -3.166790 -1.476031 0.334440
C -3.631193 -2.894699 -2.950050
H -4.040280 -2.069003 -3.553056
H -2.590761 -3.069676 -3.254730
H -4.195147 -3.800257 -3.198968
C -4.790154 -3.376556 -0.826270
H -4.612854 -4.460831 -0.884764
H -4.800704 -3.074590 0.224559
H -5.778520 -3.168297 -1.263561
N -3.754767 -2.647454 -1.525139
C 1.652981 1.146614 0.534597
N 1.764447 2.465674 0.764890
O 0.546461 0.620436 0.359189
C 2.926318 0.304384 0.493830
H 3.511803 0.547992 1.398954
H 2.601781 -1.575053 -0.392748
C 3.751569 0.612798 -0.753646
H 3.118795 0.420330 -1.639329
H 3.975883 1.689867 -0.780475
C 5.060259 -0.168577 -0.877884
H 4.808927 -1.240698 -0.985022
C 5.939550 -0.018647 0.353030
H 5.480664 -0.454038 1.252903
H 6.907987 -0.520733 0.213507
H 6.150840 1.042431 0.566908
C 5.794067 0.263527 -2.136779
H 5.160909 0.166376 -3.031256
H 6.105823 1.318467 -2.072454
H 6.700912 -0.335640 -2.304284
C 1.876016 -1.677765 1.494463
N 2.615006 -1.102442 0.507170
O 1.613050 -1.149567 2.562020
O 1.514739 -2.914125 1.106031
C 0.896554 -3.876264 2.027731
C -0.441331 -3.359194 2.525433
H -1.061545 -3.003207 1.688635
H -0.991254 -4.174512 3.016479
H -0.318519 -2.552377 3.257278
C 1.856351 -4.172635 3.164690
H 2.030245 -3.287828 3.789145
H 1.442387 -4.963352 3.805863
H 2.823327 -4.525395 2.779059
C 0.700050 -5.096640 1.148322
H 0.010688 -4.880388 0.319903
H 1.655032 -5.432277 0.721256
H 0.277145 -5.924237 1.733136
Cs -1.637349 0.162295 2.390962
F -2.376452 2.879225 2.426583

8. Crystallographic information: structure of 1a

Compound **1a** was crystalized *via* vapor diffusion using a EtOAc/pentane solvent system. Solid-state data for **1a** was collected using a Rigaku XtaLAB Synergy-R DW diffractometer with a HyPix-Arc 150 detector. Crystals were selected under perfluoropolyether oil, mounted on a MiTeGen Micromount loop and quench-cooled using an Oxford Cryosystems open flow N_2 cooling device.[26] Selected details of the data collection are given in **Table S9**. Data were reduced using the CrysAlisPro package,[27] including unit cell parameter refinement and inter-frame scaling (which was carried out using SCALE3 ABSPACK within CrysAlisPro). Equivalent reflections were merged, and corrected; crystal faces were indexed and used to apply an absorption and illuminated volume correction with the CrysAlisPro suite. Structures were solved *ab initio* from the integrated intensities using SuperFlip[28] and refined using full-matrix least-squares on F2 using CRYSTALS.[29] Hydrogen atoms were included in the refinement with soft restraints.[30]

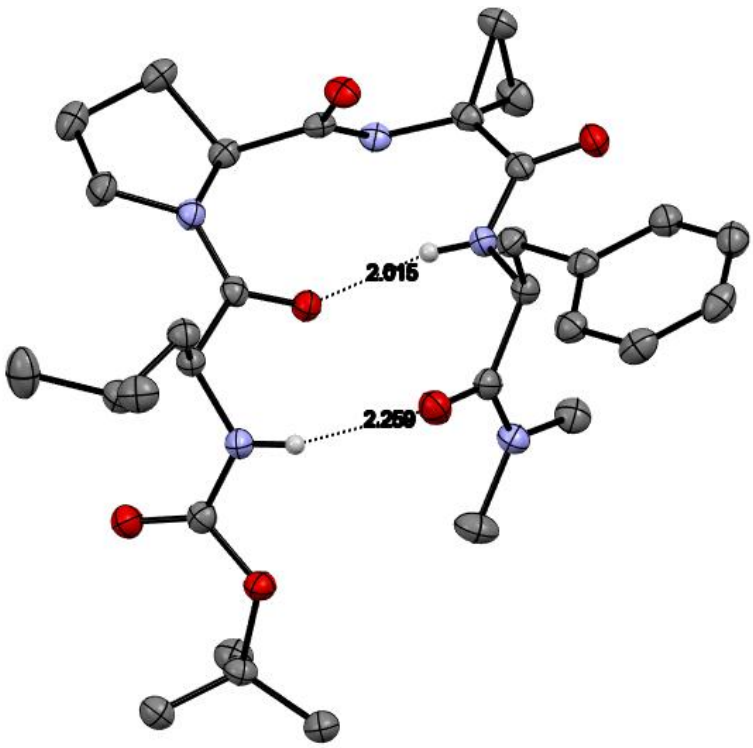

Figure S27. Crystal structure of **1a** with 50% thermal ellipsoids. Most H-atoms have been omitted for clarity, while some are depicted as circles. Dashed lines highlight H-bonding interactions.

Table S9. Selected X-ray data collection and refinement parameters.

**Name**	*tert*-Butyl ((*S*)-1-((*S*)-2-((1-(((*S*)-1-(dimethylamino)-1-oxo-3-phenylpropan-2-yl)carbamoyl)cyclopropyl)carbamoyl)pyrrolidin-1-yl)-4-methyl-1-oxopentan-2-yl)carbamate (**1a**)
**Formula**	$C_{31}H_{47}N_5O_6$
**Fw (g mol^{-1})**	585.74
**Cell system**	monoclinic
**Space group**	$P2_1$
a **(Å)**	10.6739(1)
b **(Å)**	9.4321(1)
c **(Å)**	16.1917(1)
α **(°)**	90
β **(°)**	95.5259(4)
γ **(°)**	90
V **(Å^{-3})**	1622.56(3)
Z	2
**ρ_{calc} (g cm^{-3})**	1.199
**Radiation, λ (Å)**	CuKα (λ = 1.54184)
**Absorption**	Gaussian
**μ (mm^{-1})**	0.679
**$R_{(int)}$**	0.0265
**Parameters**	392
**R_1 (all data/*I* >2σ(I))**	0.0265 / 0.0259
**Ωr_2 (all data/*I* >2σ(I))**	0.0687 / 0.0682
**GooF**	0.9852
T **(K)**	100.15
**Largest diff. peak/hole (e Å^{-3})**	0.14 / −0.15
**Flack parameter**	-0.04(9)
**CCDC Deposition No.**	2492827

9. NMR spectra

9.1. NMR spectra of catalysts and peptide intermediates

^{1}H NMR (400 MHz, $CDCl_3$): Boc-Phe-NEt_2 (**S1**)

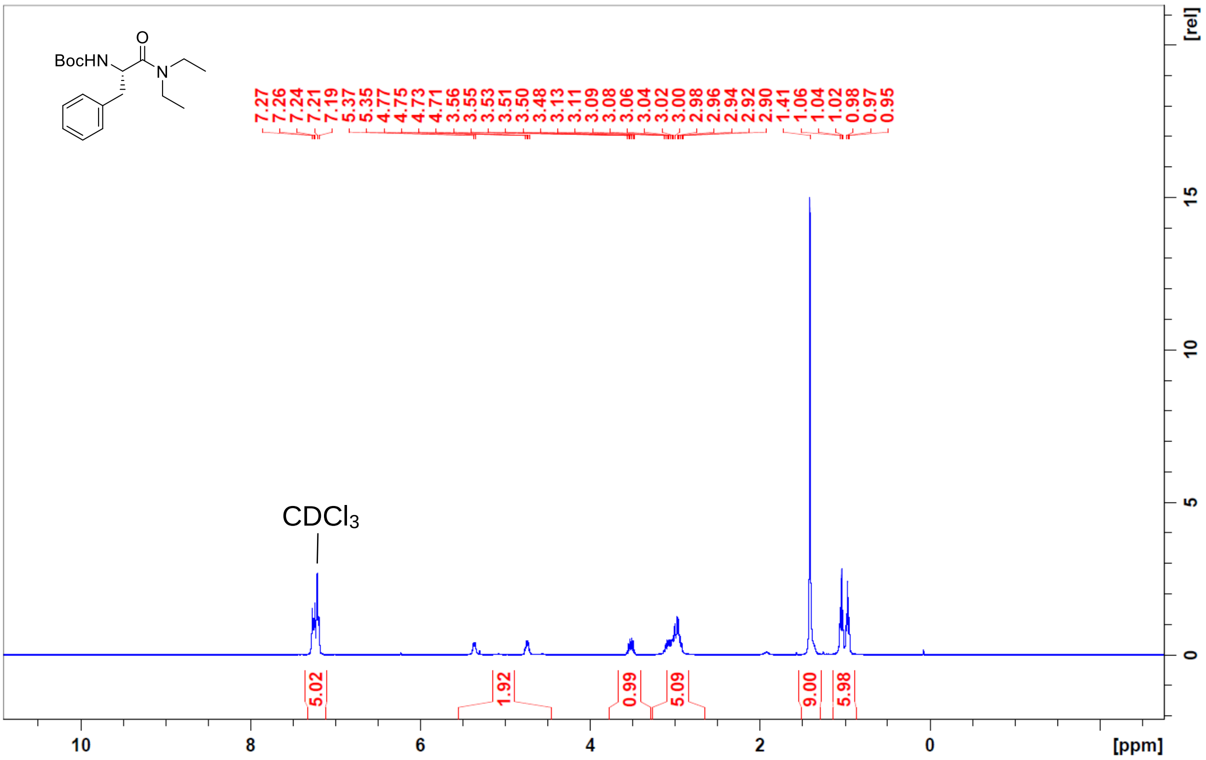

^{13}C NMR (101 MHz, $CDCl_3$): Boc-Phe-NEt_2 (**S1**)

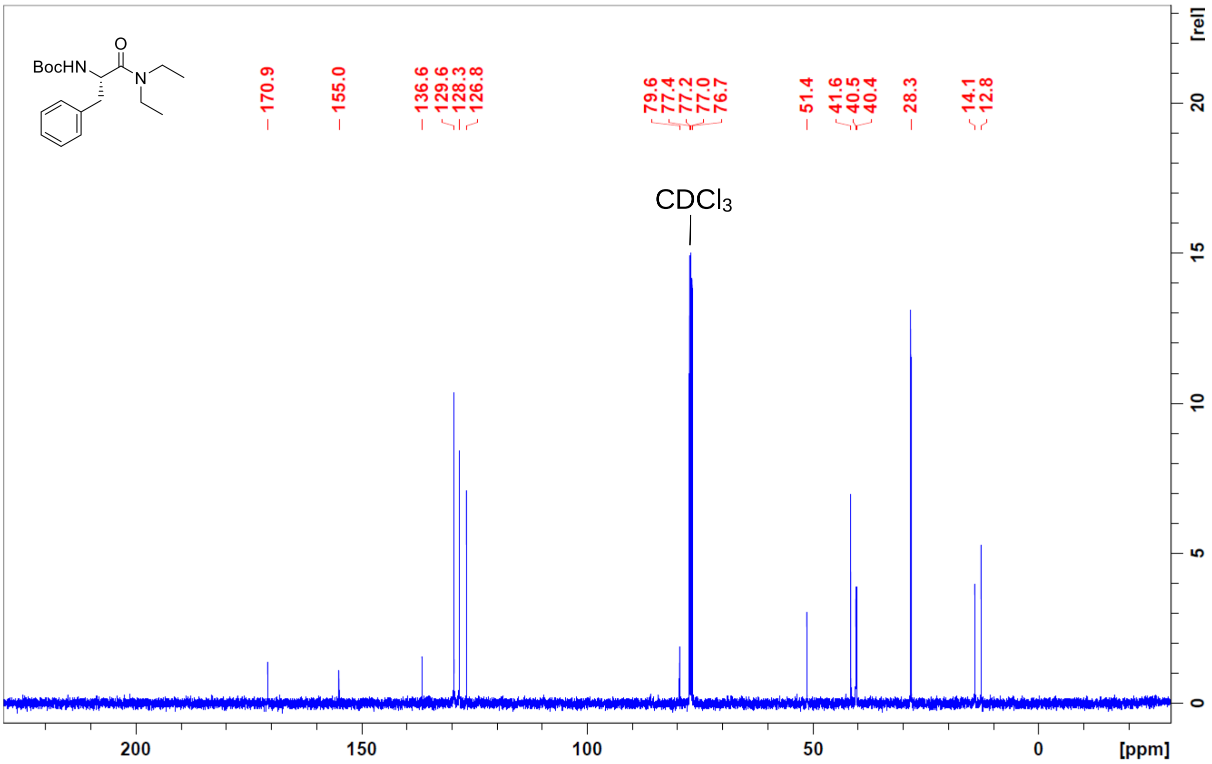

^{1}H NMR (400 MHz, $CDCl_3$): H-Phe-NEt_2·HCl (**S2**)

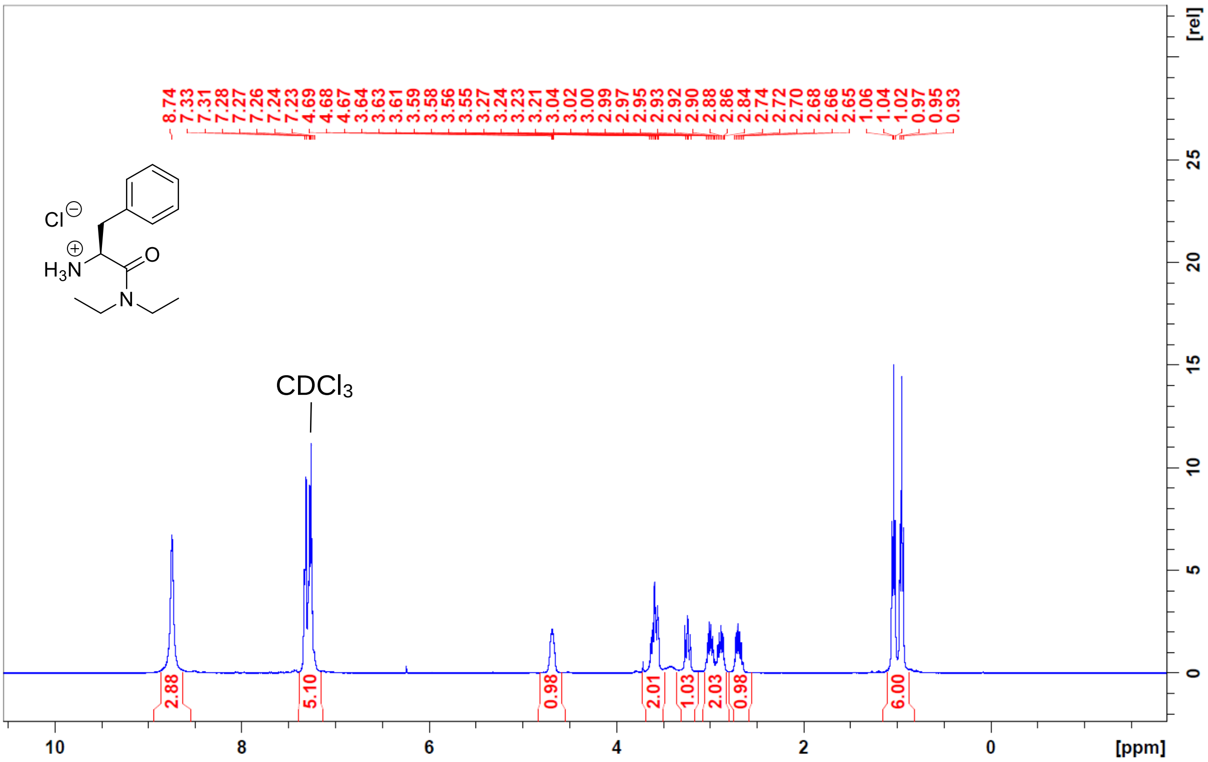

^{13}C NMR (101 MHz, $CDCl_3$): H-Phe-NEt_2·HCl (**S2**)

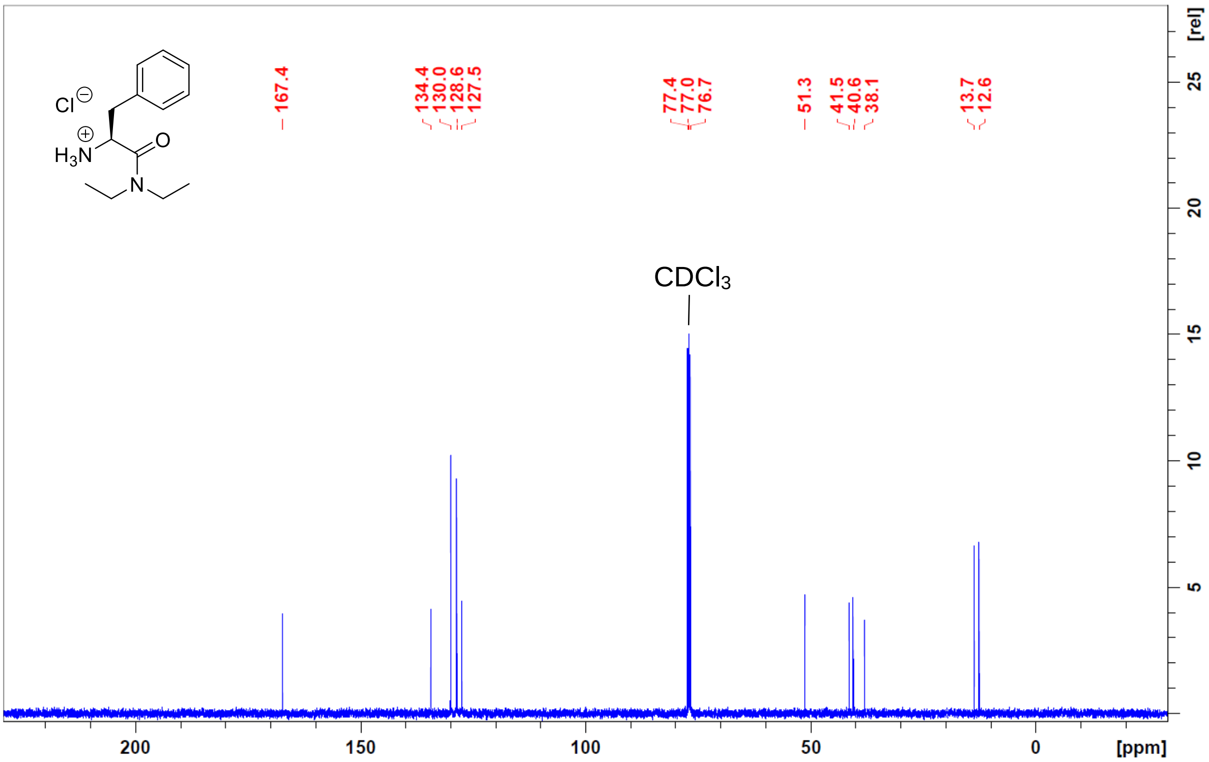

^{1}H NMR (400 MHz, $CDCl_3$): Boc-Acpc-Phe-NEt_2 (**S3**)

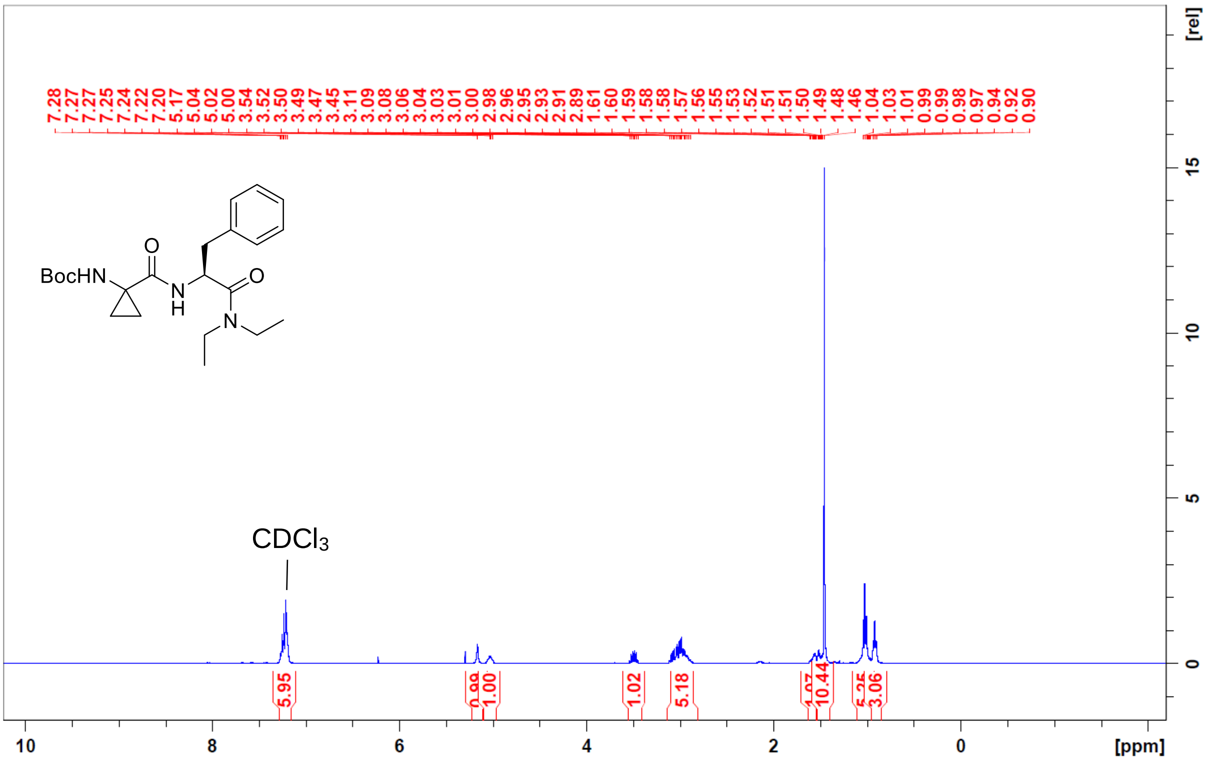

^{13}C NMR (101 MHz, $CDCl_3$): Boc-Acpc-Phe-NEt_2 (**S3**)

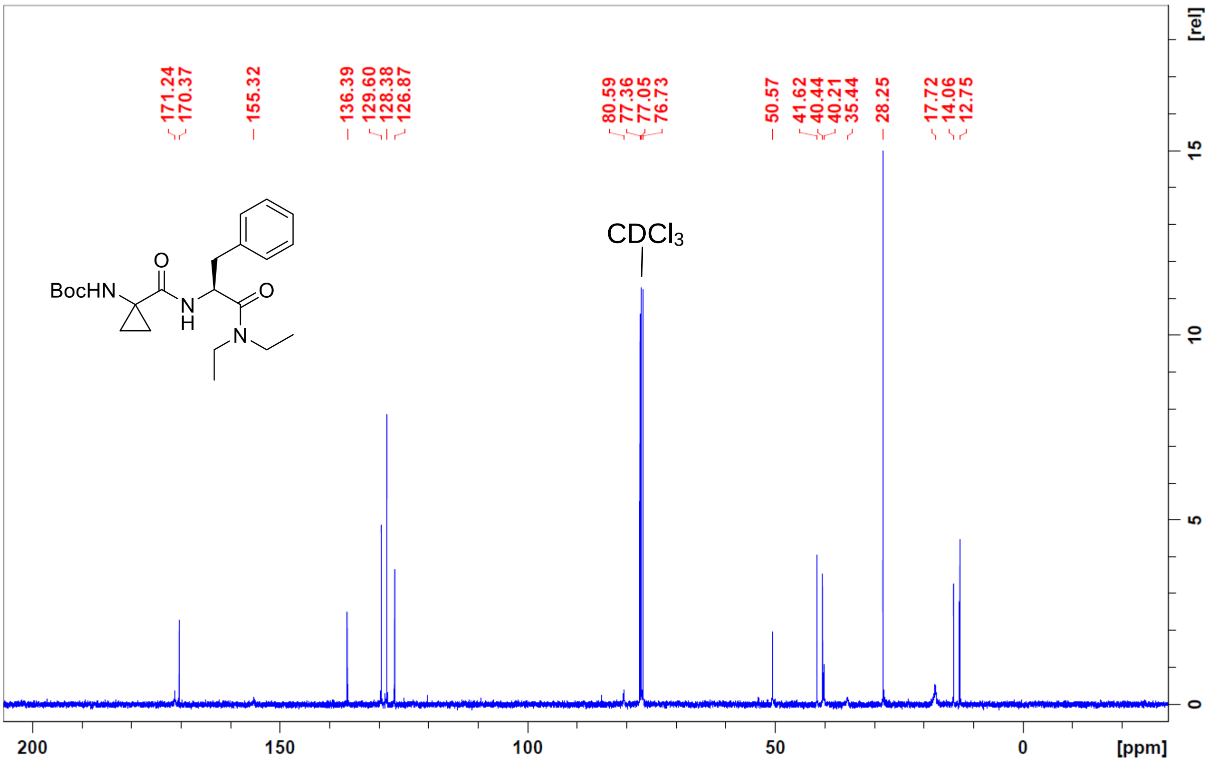

^{1}H NMR (400 MHz, D_2O): H-Acpc-Phe-NEt_2·HCl (**S4**)

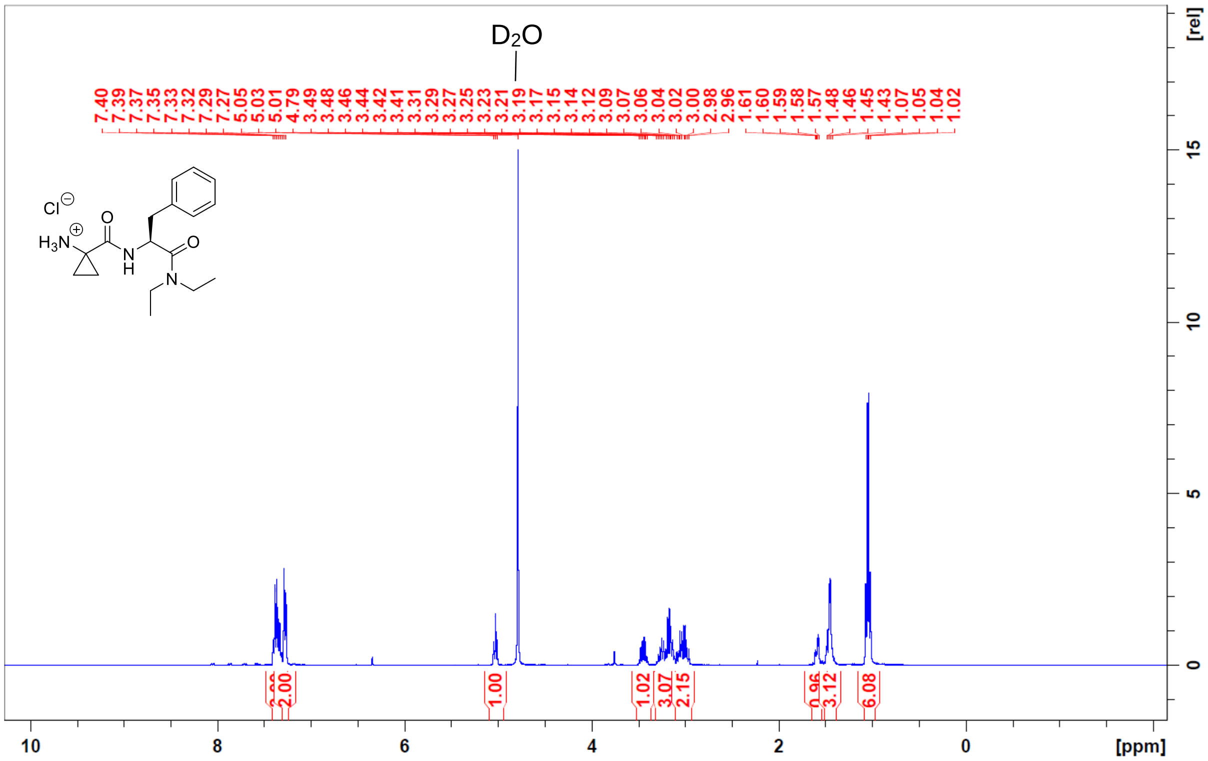

^{13}C NMR (101 MHz, D_2O): H-Acpc-Phe-NEt_2·HCl (**S4**)

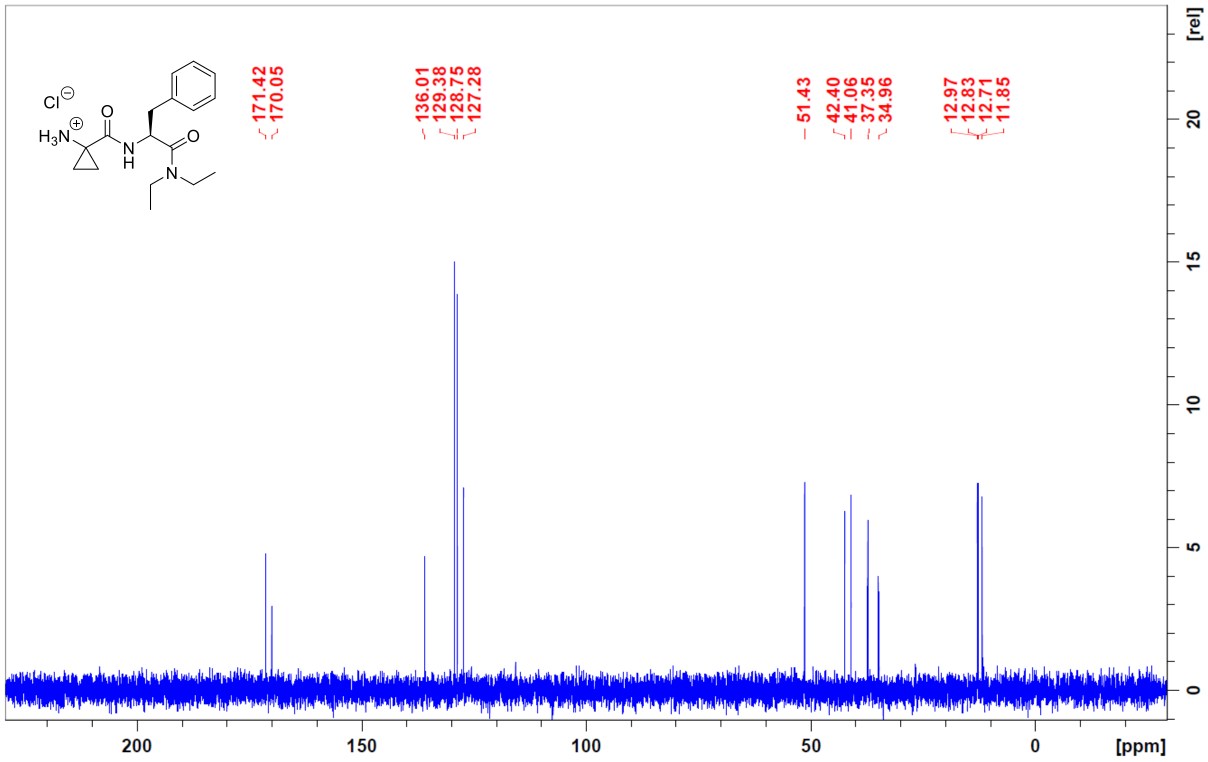

^{1}**H NMR** (500 MHz, $CDCl_3$, **at 333 K**): Boc-flp-Acpc-Phe-NEt_2 (**S5**)

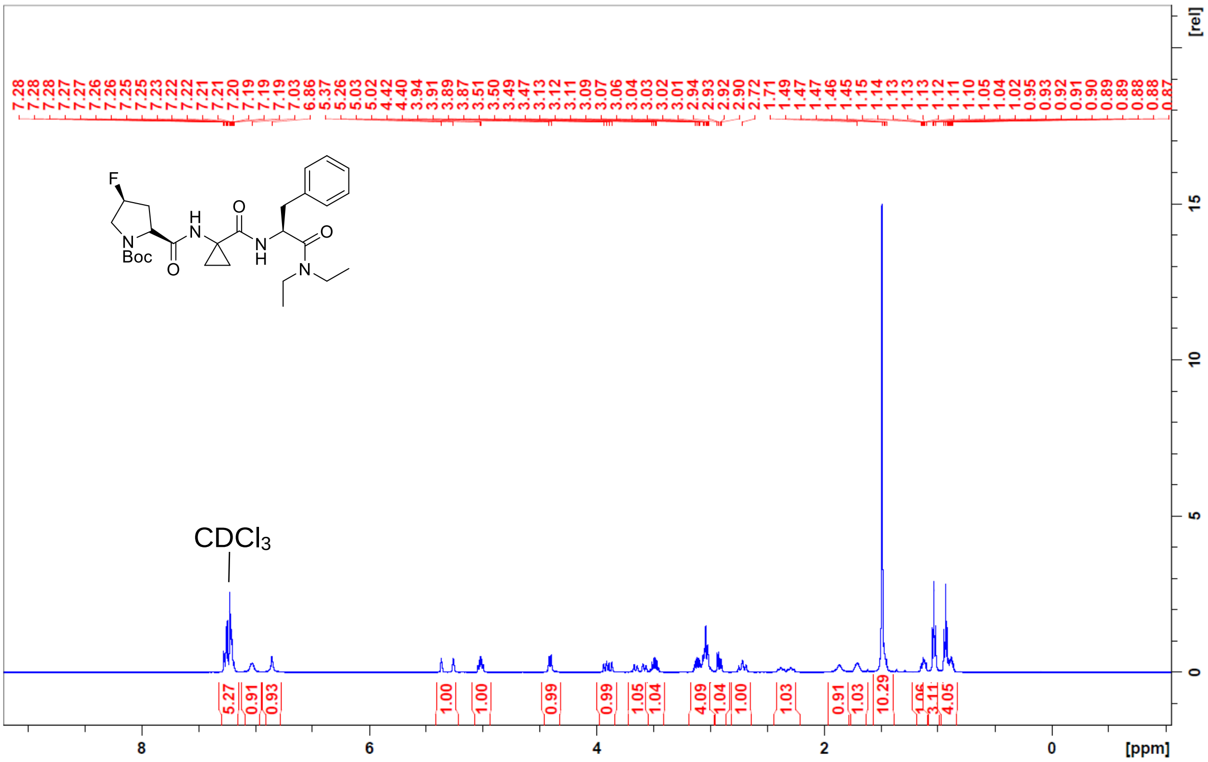

^{13}**C NMR** (126 MHz, $CDCl_3$, **at 333 K**): Boc-flp-Acpc-Phe-NEt_2 (**S5**)

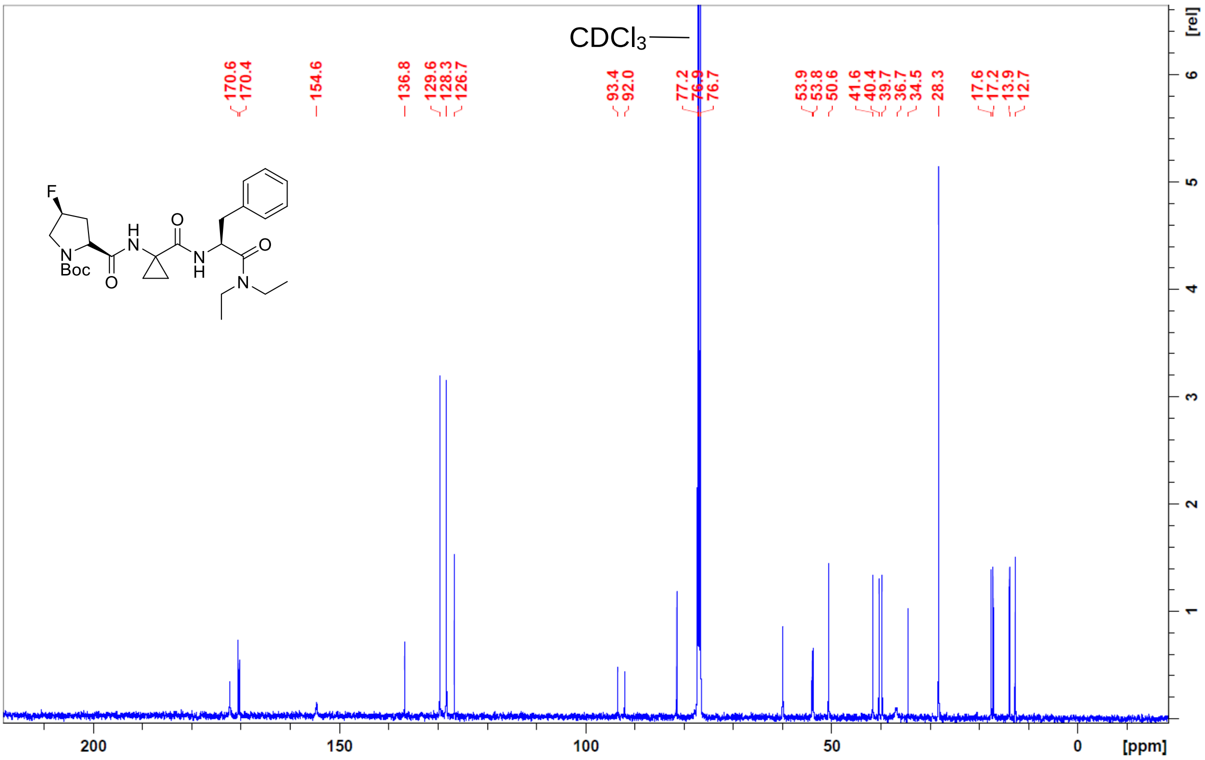

^{19}F NMR (377 MHz, CDCl3, at 298 K): Boc-flp-Acpc-Phe-NEt2 (**S5**)

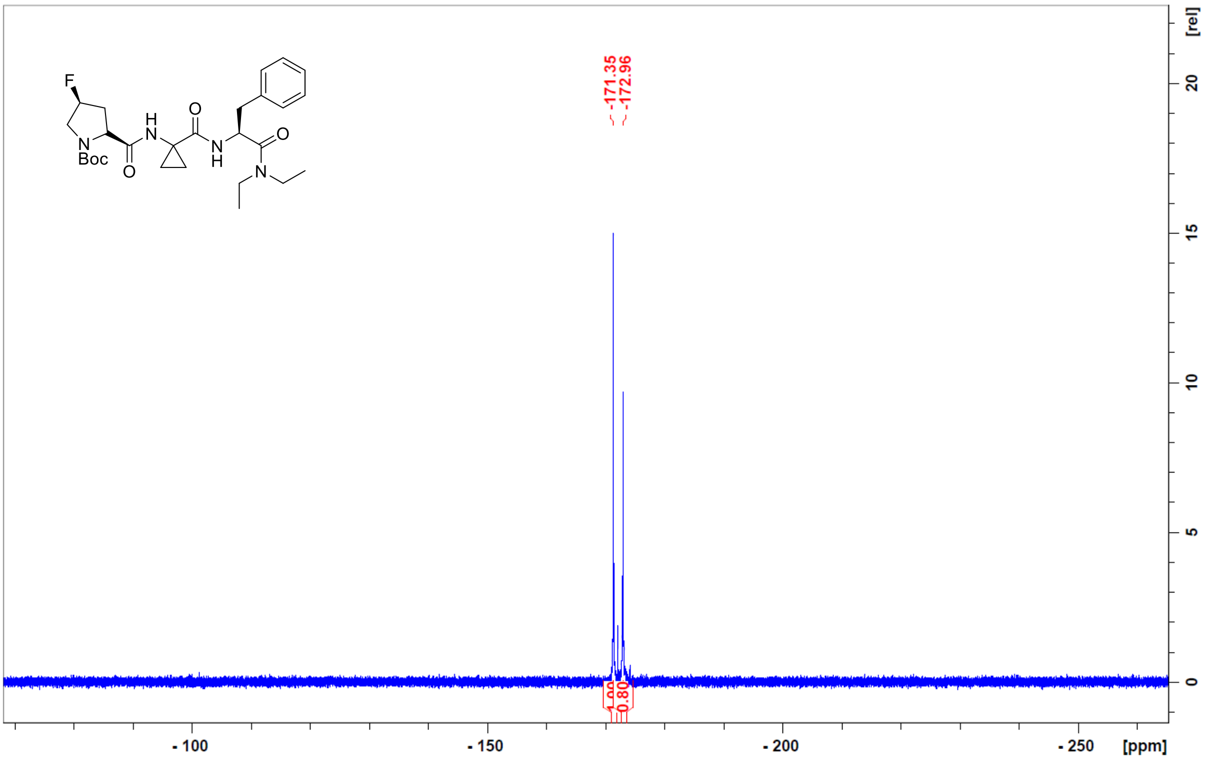

^{1}H NMR (500 MHz, CDCl3): H-flp-Acpc-Phe-NEt2·HCl (**S6**)

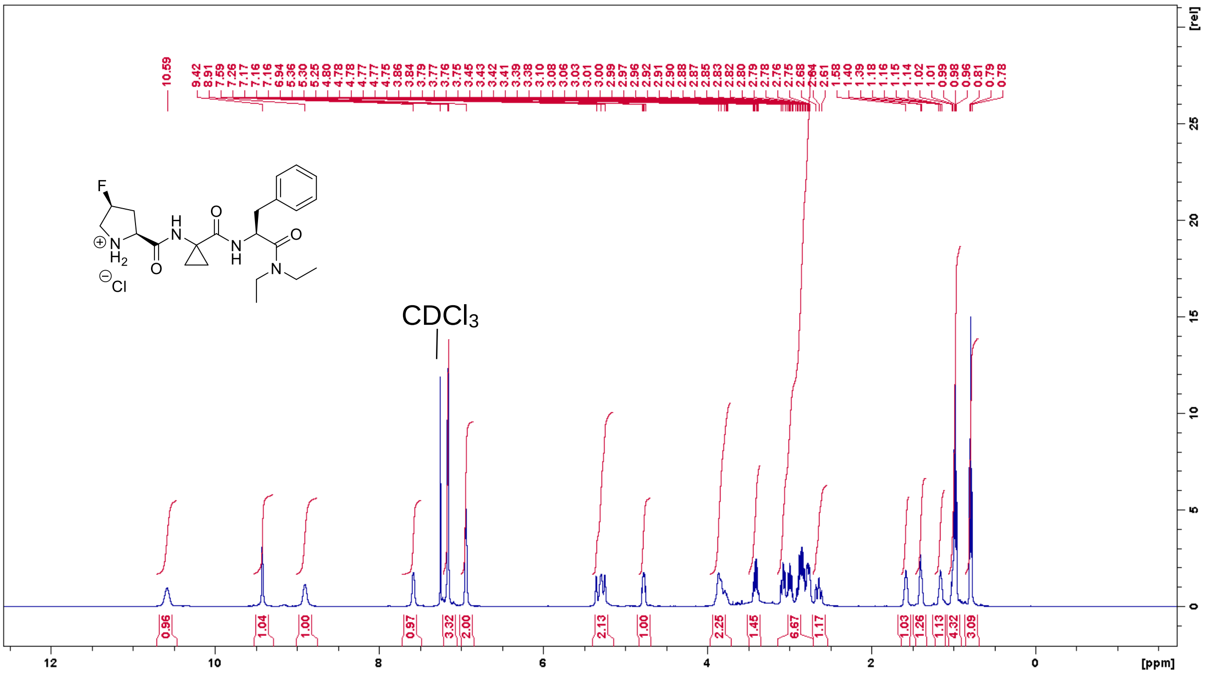

^{13}C NMR (126 MHz, $CDCl_3$): H-flp-Acpc-Phe-NEt_2·HCl (**S6**)

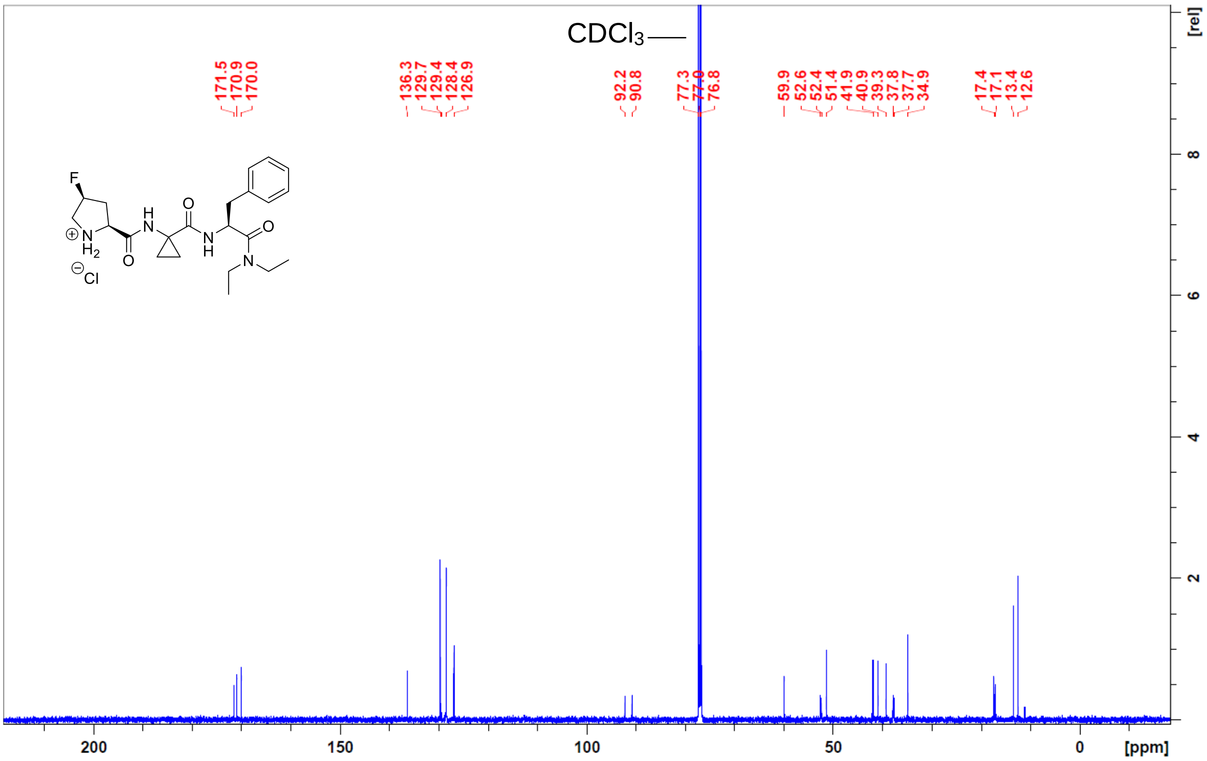

^{19}F{^{1}H} NMR (471 MHz, $CDCl_3$): H-flp-Acpc-Phe-NEt_2·HCl (**S6**)

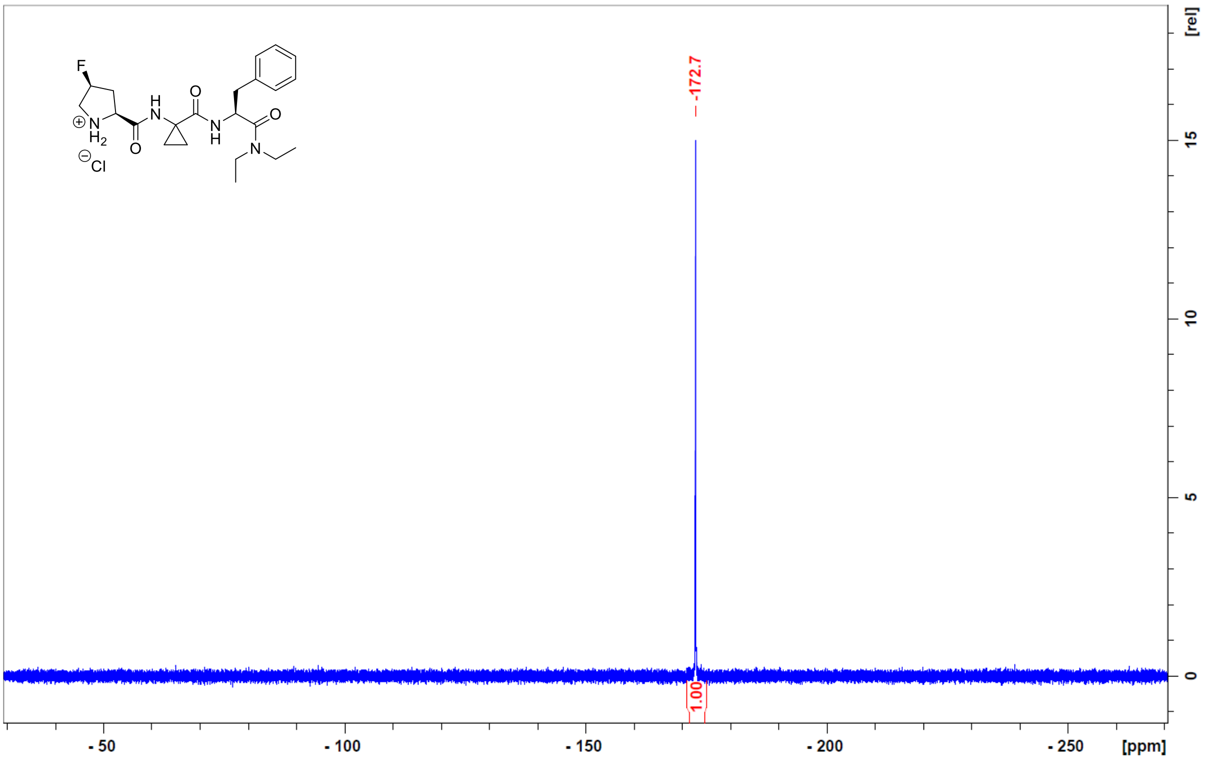

^{1}H NMR (500 MHz, CD_2Cl_2): Boc-Leu-Pro-Acpc-Phe-NMe$_2$ (**1a**)

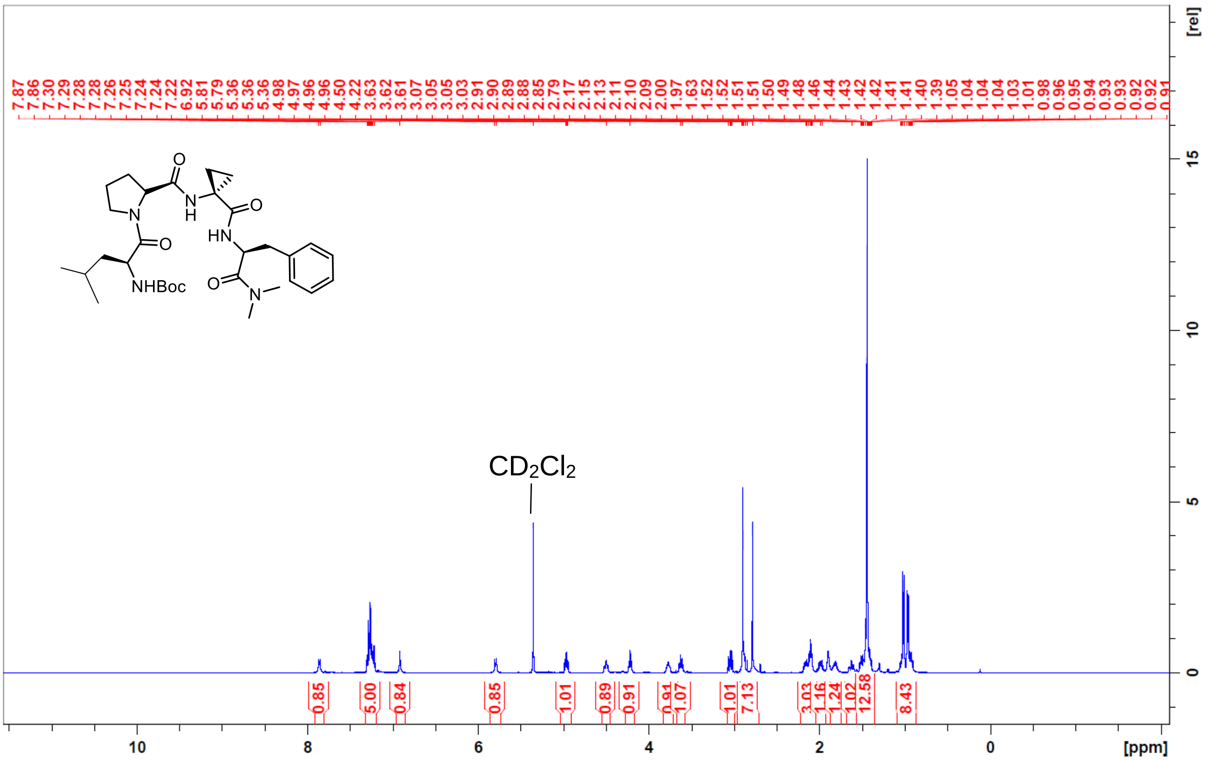

^{13}C NMR (126 MHz, CD_2Cl_2): Boc-Leu-Pro-Acpc-Phe-NMe$_2$ (**1a**)

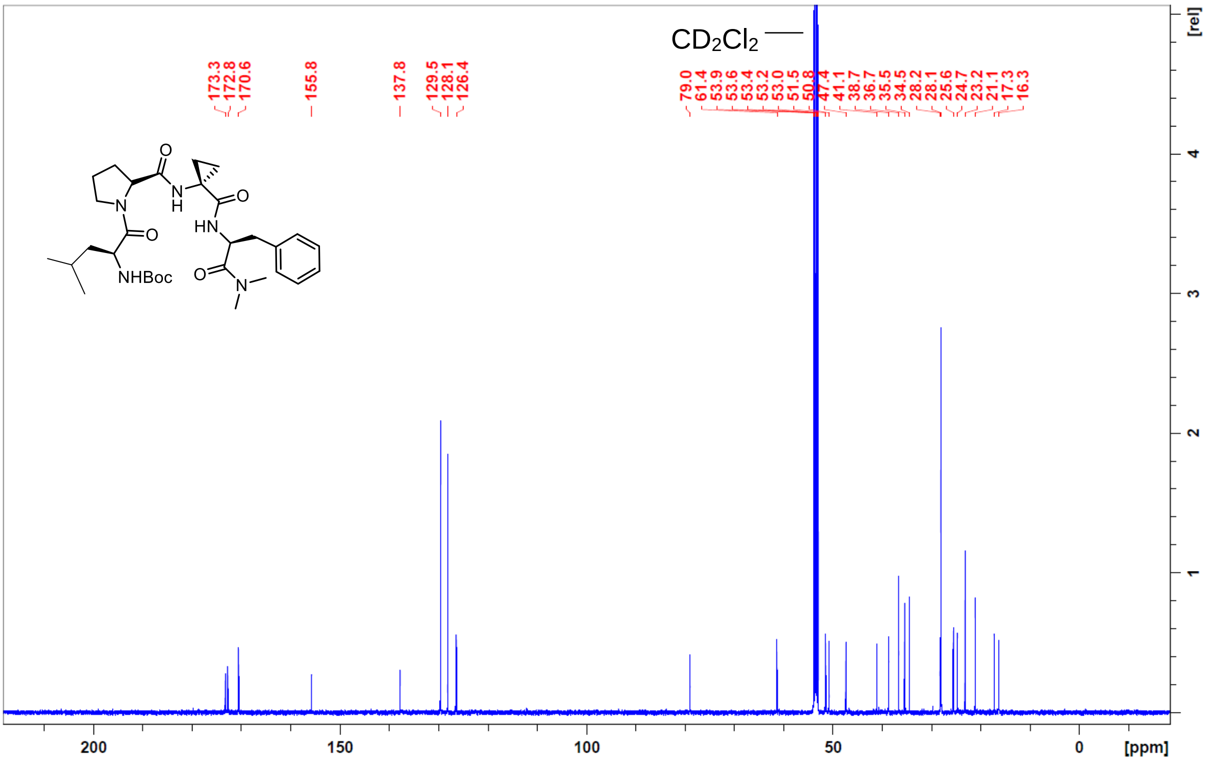

^{1}H NMR (500 MHz, CD_3OD): 3,5-$(CF_3)_2C_6H_3$NHC(O)-Pro-Acpc-Phe-NMe_2 (**1b**)

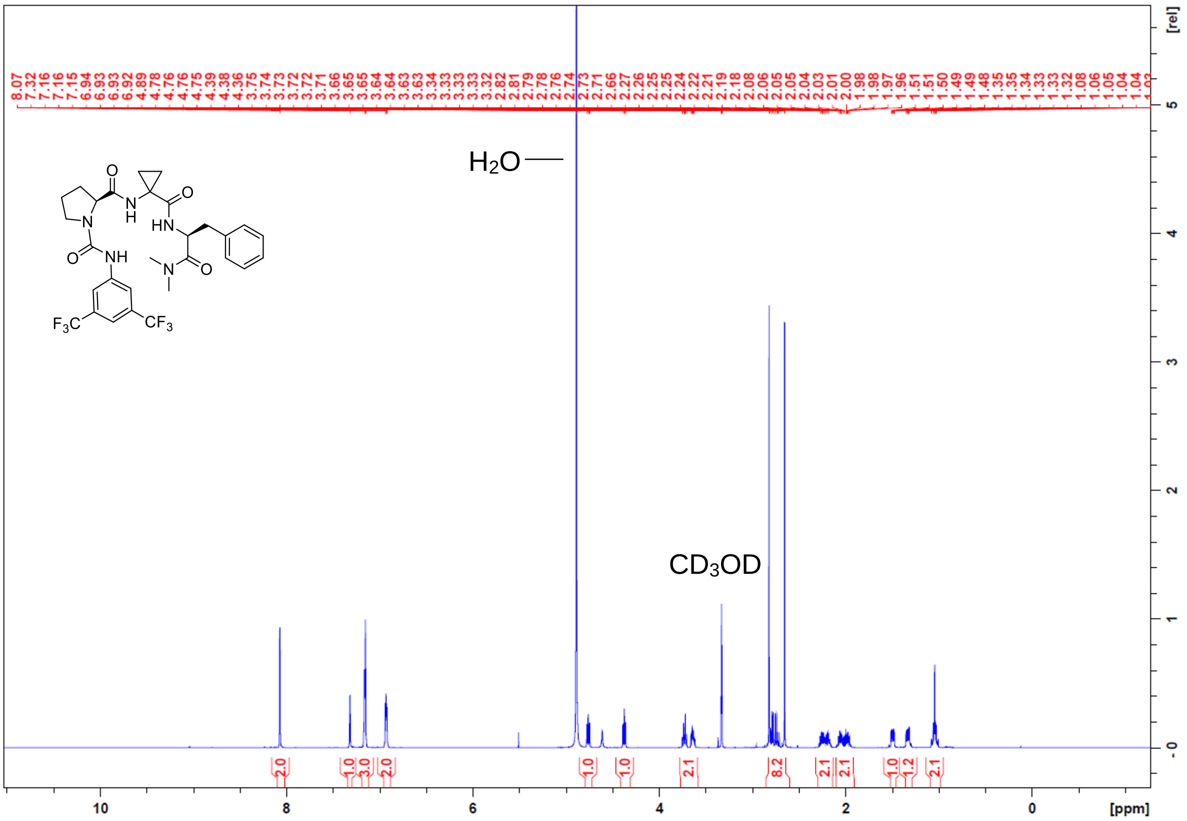

^{13}C NMR (126 MHz, CD_3OD): 3,5-$(CF_3)_2C_6H_3$NHC(O)-Pro-Acpc-Phe-NMe_2 (**1b**)

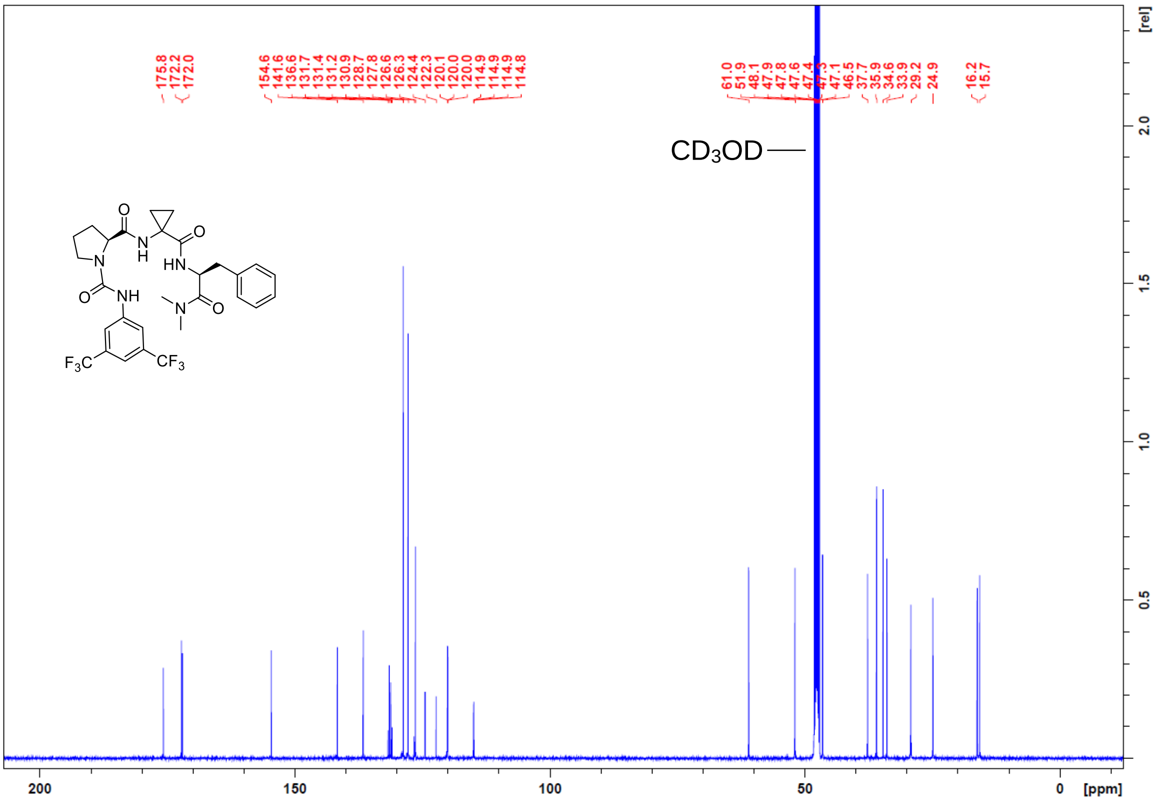

^{1}H NMR (500 MHz, $(CD_3)_2SO$): 3,5-[3,5-$(CF_3)_2C_6H_3$]$_2C_6H_3$NHC(O)-Pro-Acpc-Phe-NMe$_2$ (**1c**)

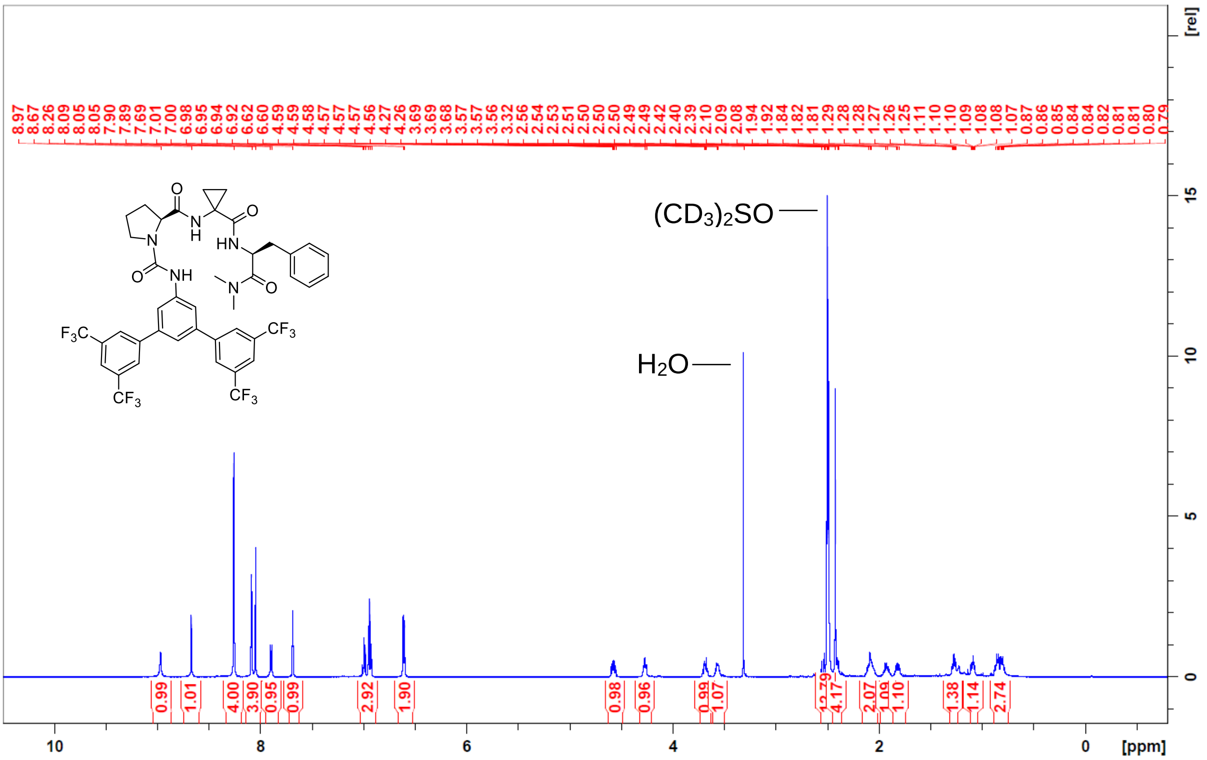

^{13}C NMR (151 MHz, $(CD_3)_2SO$): 3,5-[3,5-$(CF_3)_2C_6H_3$]$_2C_6H_3$NHC(O)-Pro-Acpc-Phe-NMe$_2$ (**1c**)

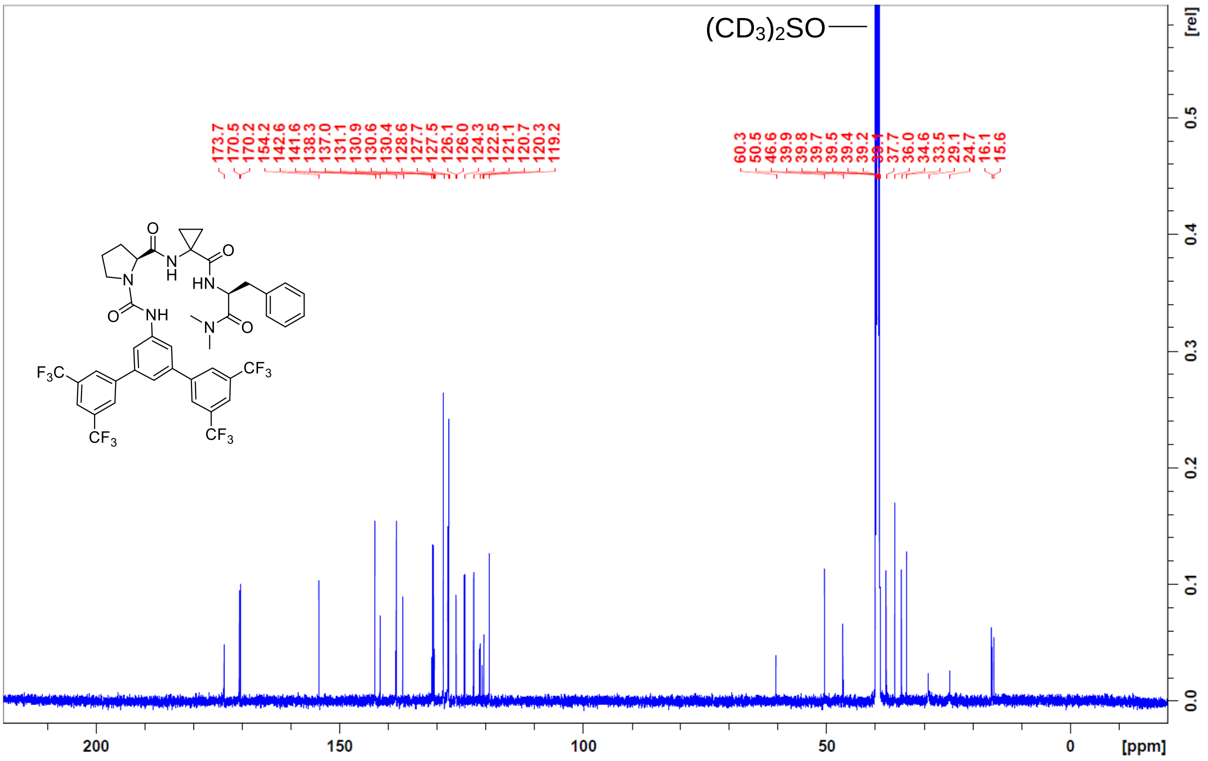

^{19}F NMR (470 MHz, $(CD_3)_2SO$): 3,5-[3,5-$(CF_3)_2C_6H_3]_2C_6H_3$NHC(O)-Pro-Acpc-Phe-NMe_2 (**1c**)

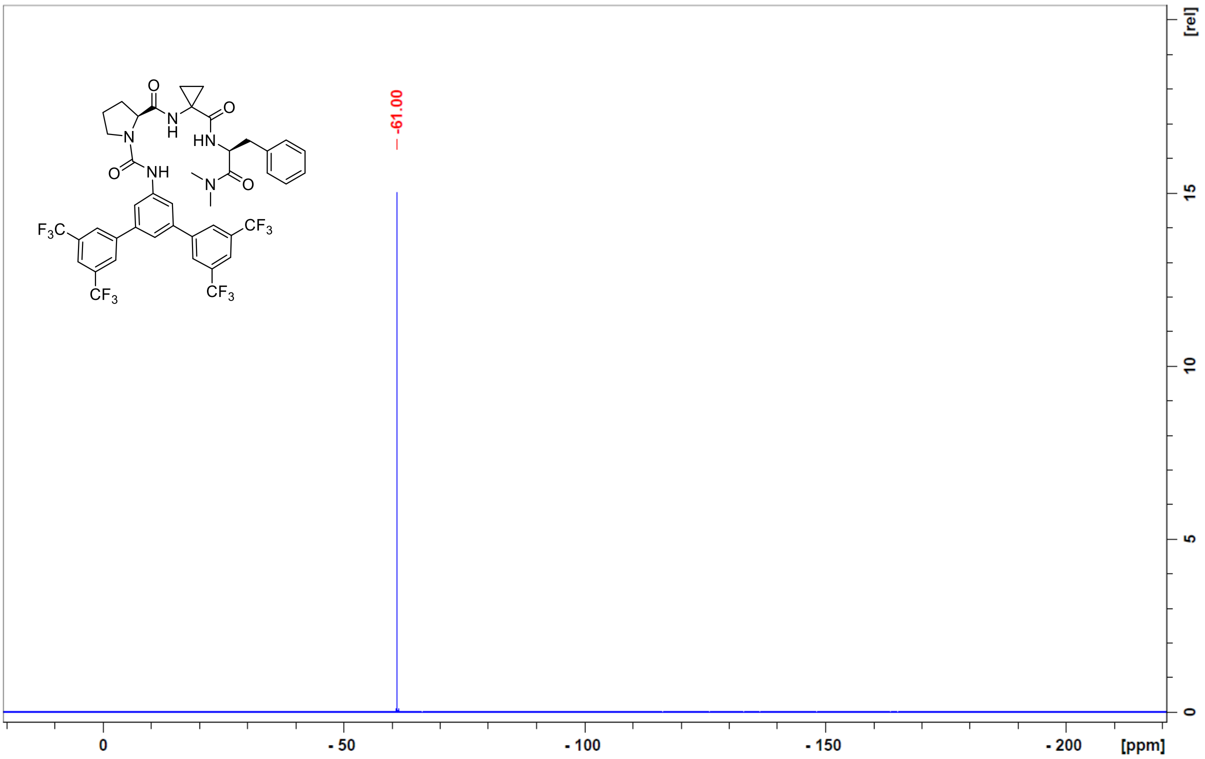

^{1}H NMR (600 MHz, $(CD_3)_2SO$): 3,5-[3,5-$(CF_3)_2C_6H_3]_2C_6H_3$NHC(O)-flp-Acpc-Phe-NMe_2 (**1d**)

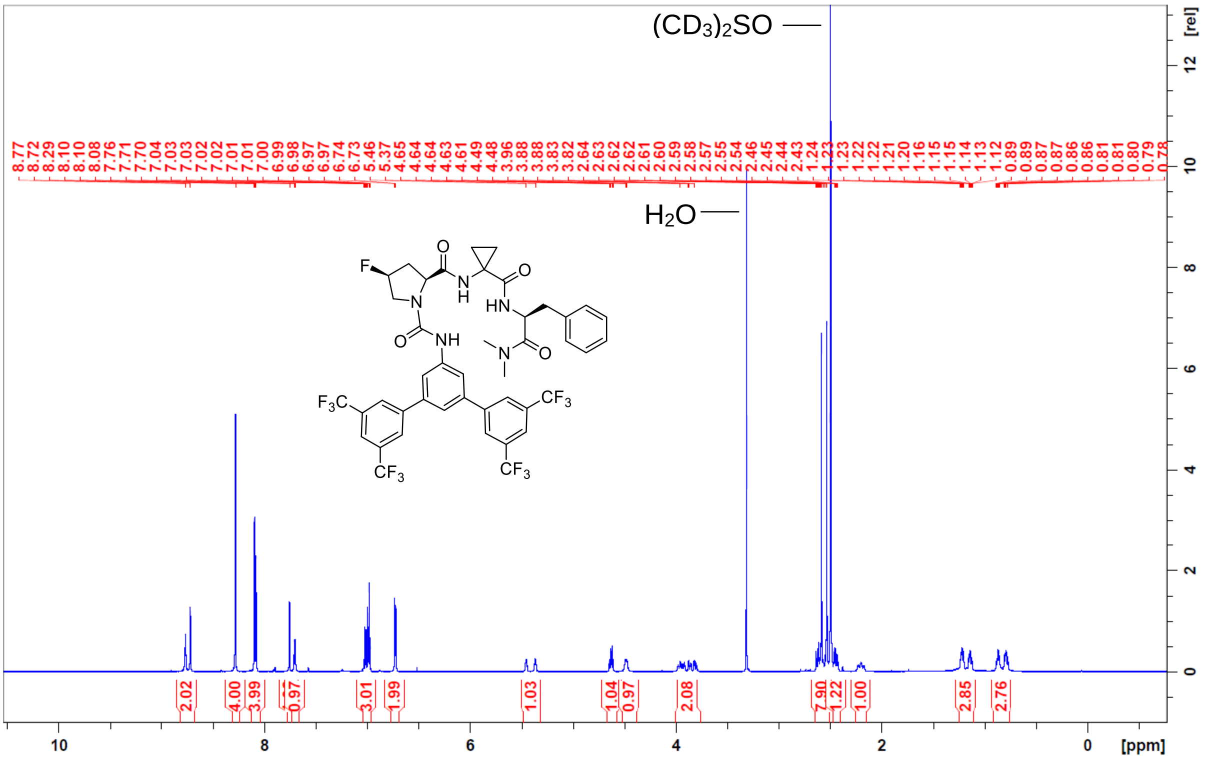

^{13}C NMR (151 MHz, $(CD_3)_2SO$): 3,5-[3,5-$(CF_3)_2C_6H_3$]$_2C_6H_3$NHC(O)-flp-Acpc-Phe-NMe_2 (**1d**)

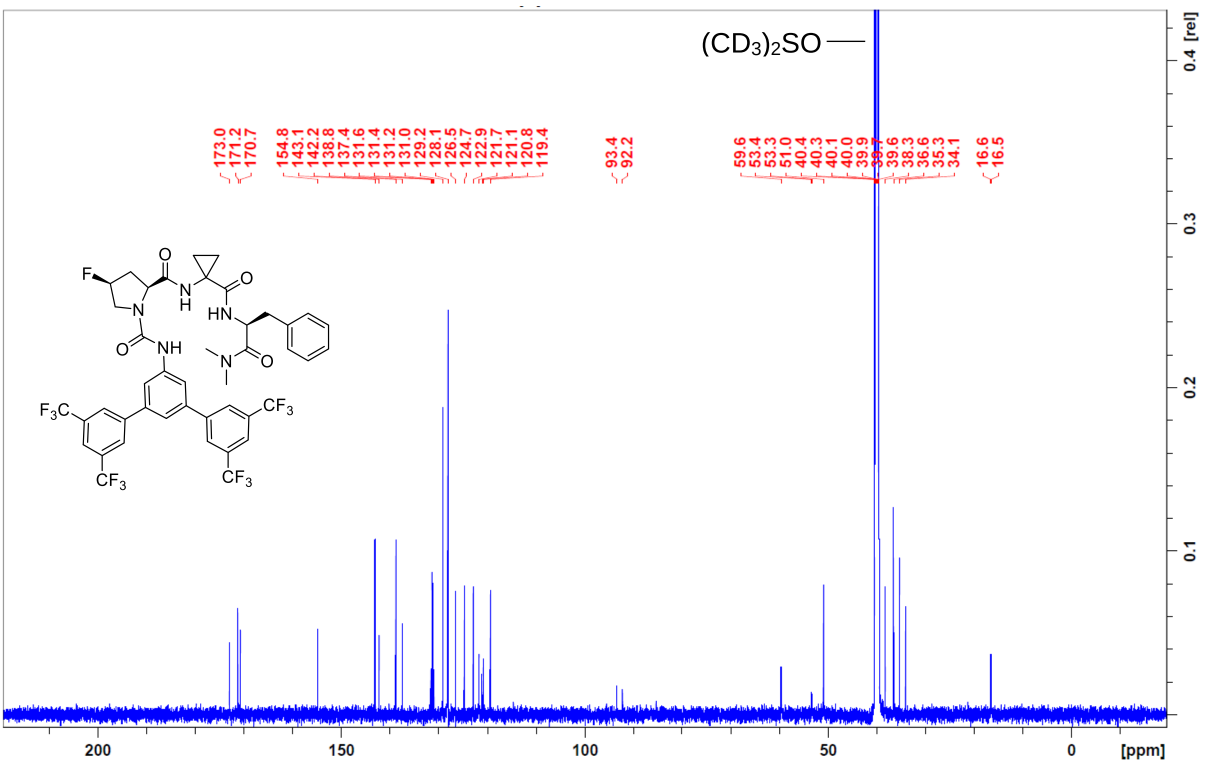

^{19}F NMR (377 MHz, $CDCl_3$): 3,5-[3,5-$(CF_3)_2C_6H_3$]$_2C_6H_3$NHC(O)-flp-Acpc-Phe-NMe_2 (**1d**)

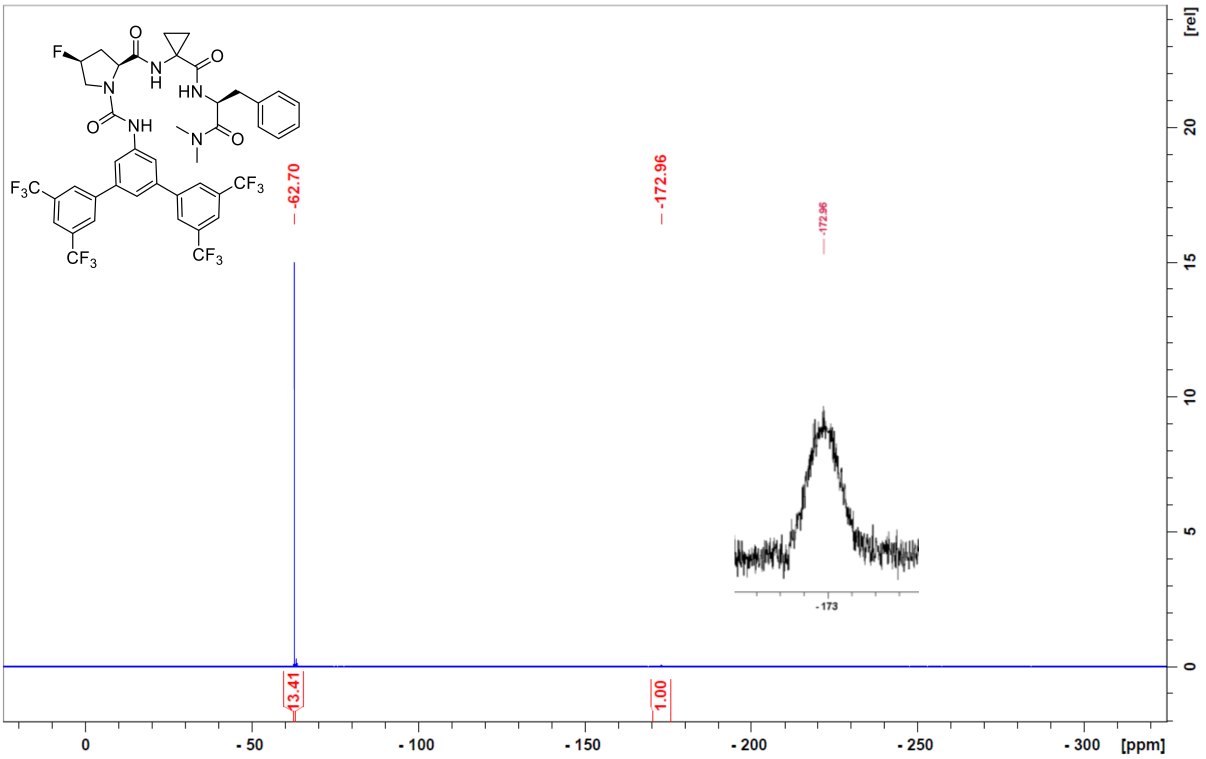

^{1}H **NMR** (600 MHz, CD_2Cl_2): 3,5-[3,5-$(CF_3)_2C_6H_3$]$_2C_6H_3$NHC(O)-flp-Acpc-Phe-NEt_2 (**1e**)

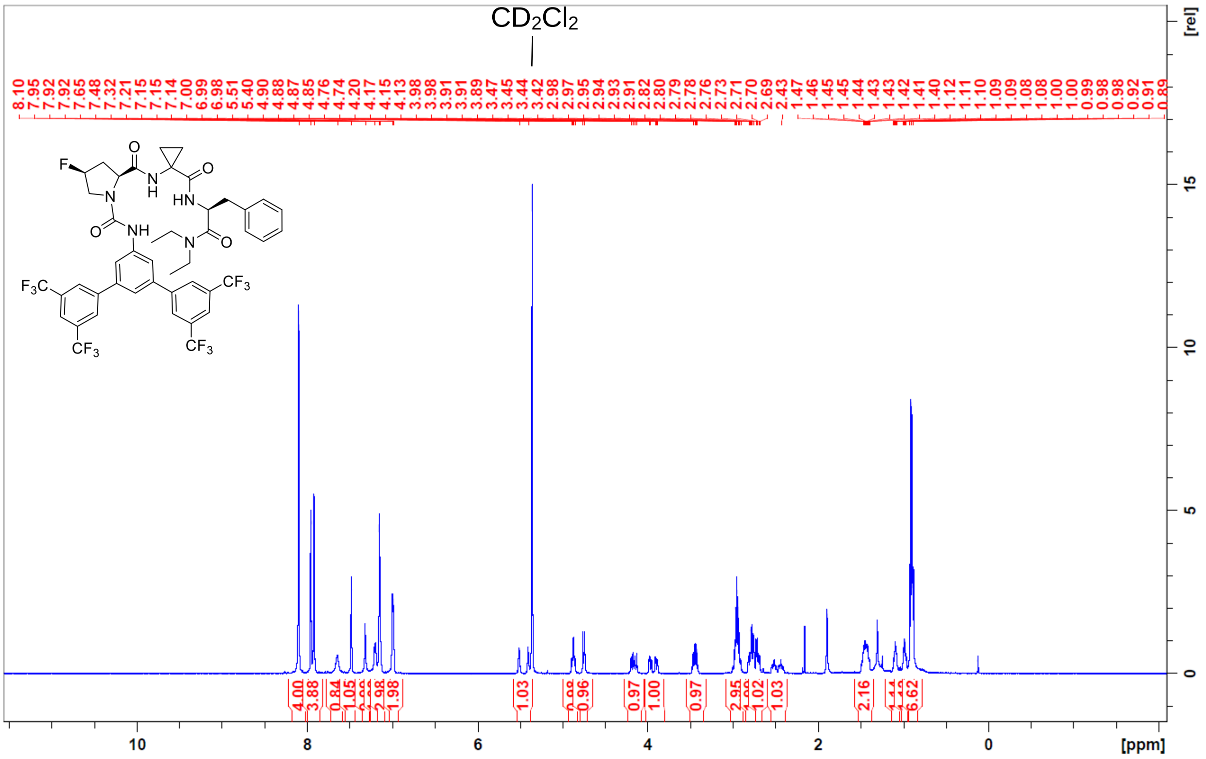

$^{19}F\{^{1}H\}$ **NMR** (471 MHz, CD_2Cl_2): 3,5-[3,5-$(CF_3)_2C_6H_3$]$_2C_6H_3$NHC(O)-flp-Acpc-Phe-NEt_2 (**1e**)

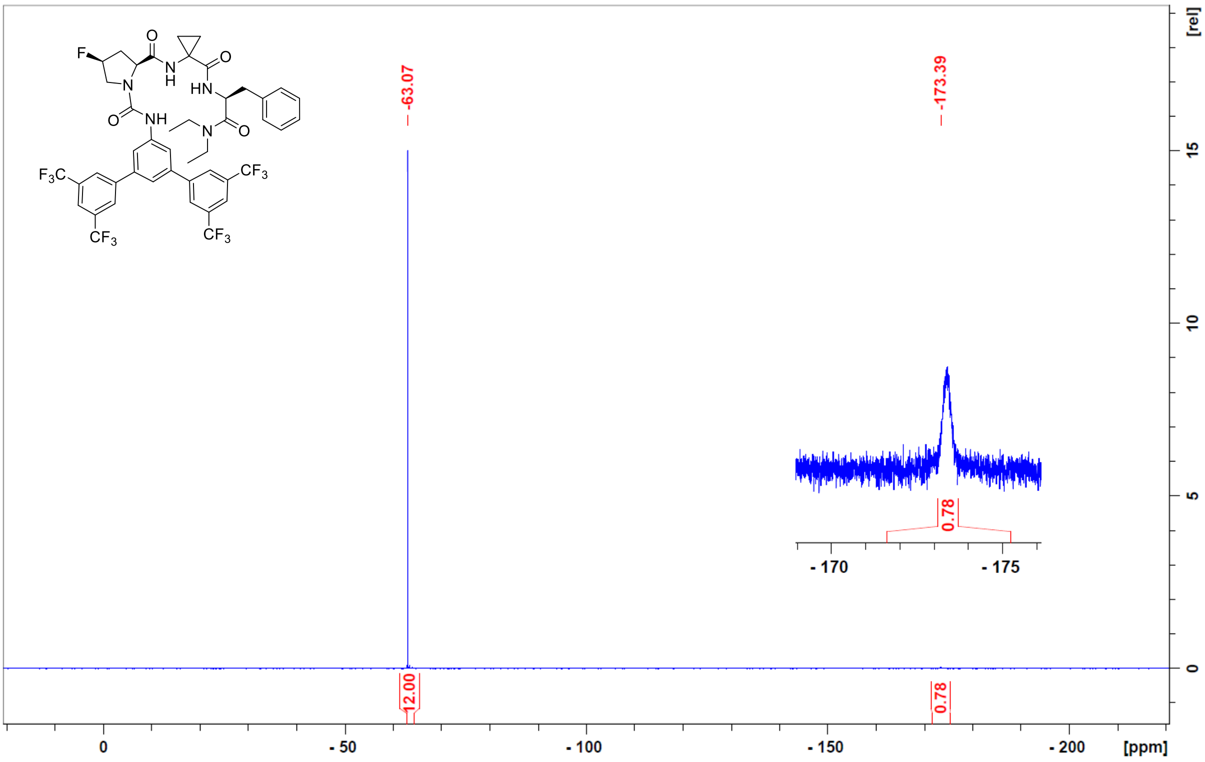

^{13}C NMR (151 MHz, CD_2Cl_2): 3,5-[3,5-$(CF_3)_2C_6H_3]_2C_6H_3NHC(O)$-flp-Acpc-Phe-$NEt_2$ (**1e**)

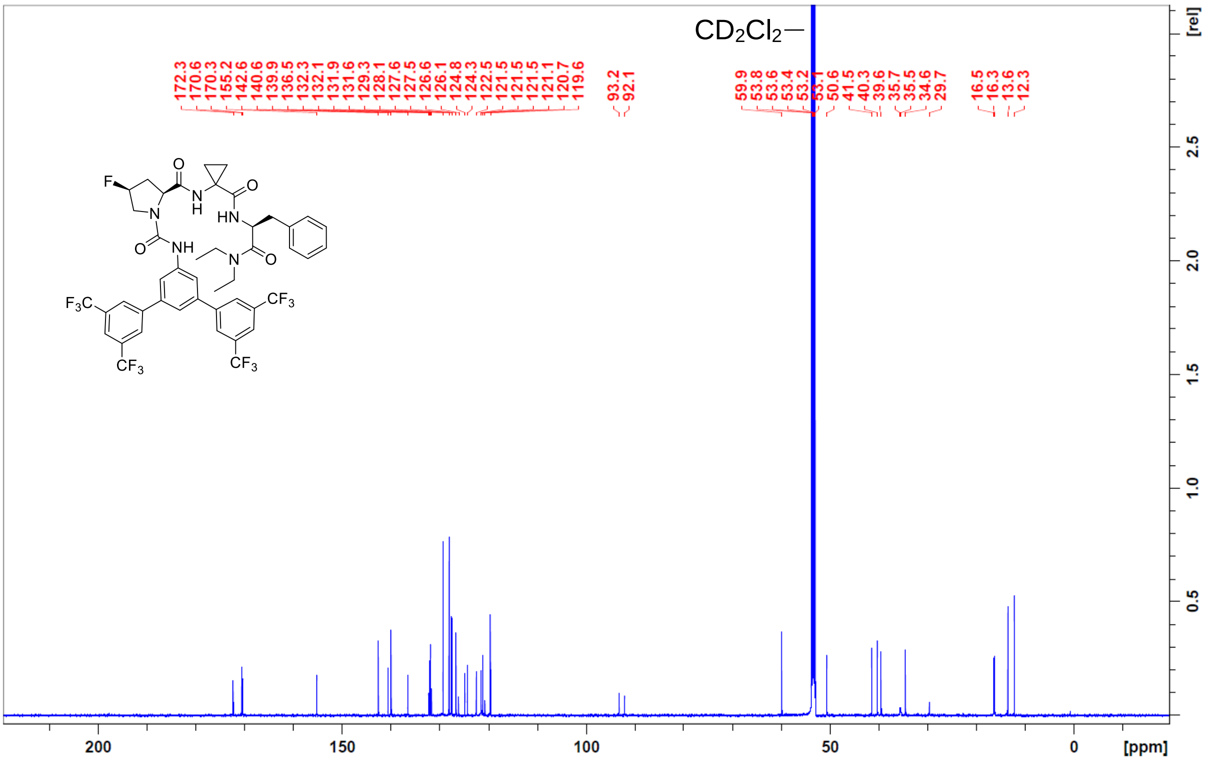

^{1}H NMR (500 MHz, CD_2Cl_2): 3,5-[3,5-$(CF_3)_2C_6H_3]_2C_6H_3NHC(O)$-Pro-Gly-Phe-$NMe_2$ (**1c-Gly(*i*+1)**)

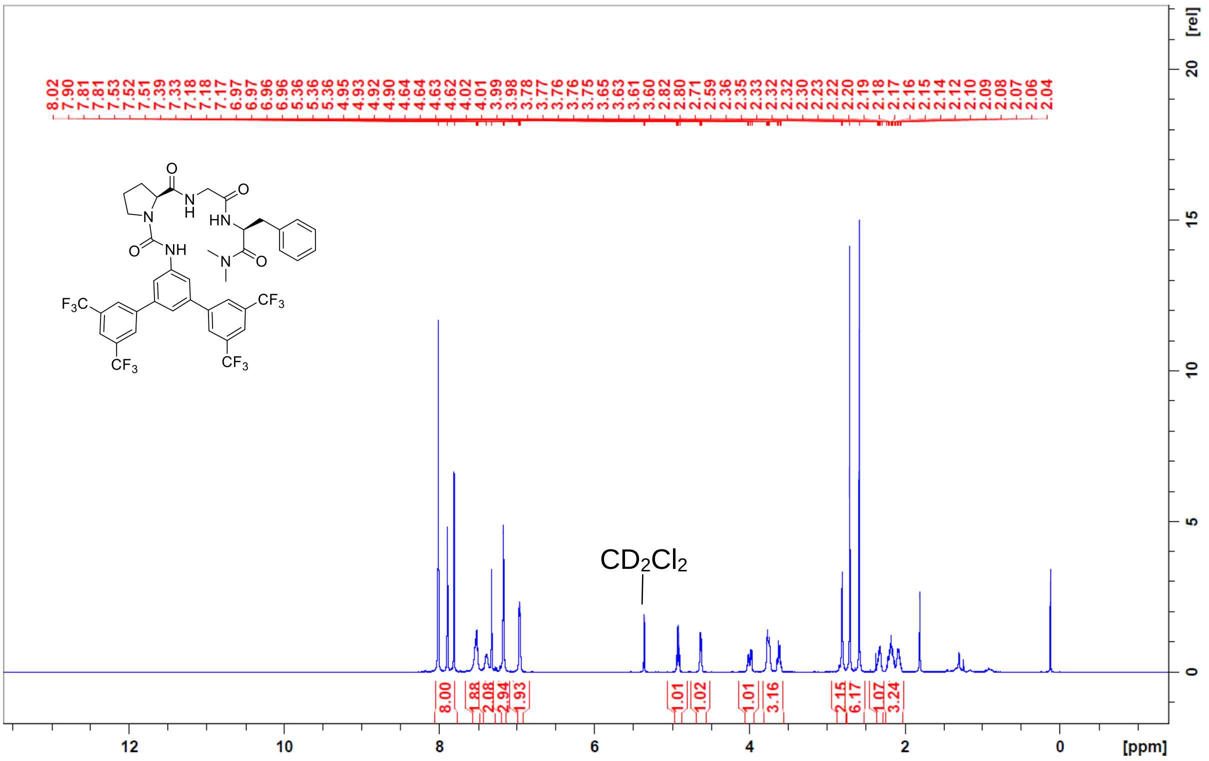

^{13}C NMR (126 MHz, $CDCl_3$): 3,5-[3,5-$(CF_3)_2C_6H_3]_2C_6H_3NHC(O)$-Pro-Gly-Phe-$NMe_2$ (**1c-Gly(*i*+1)**)

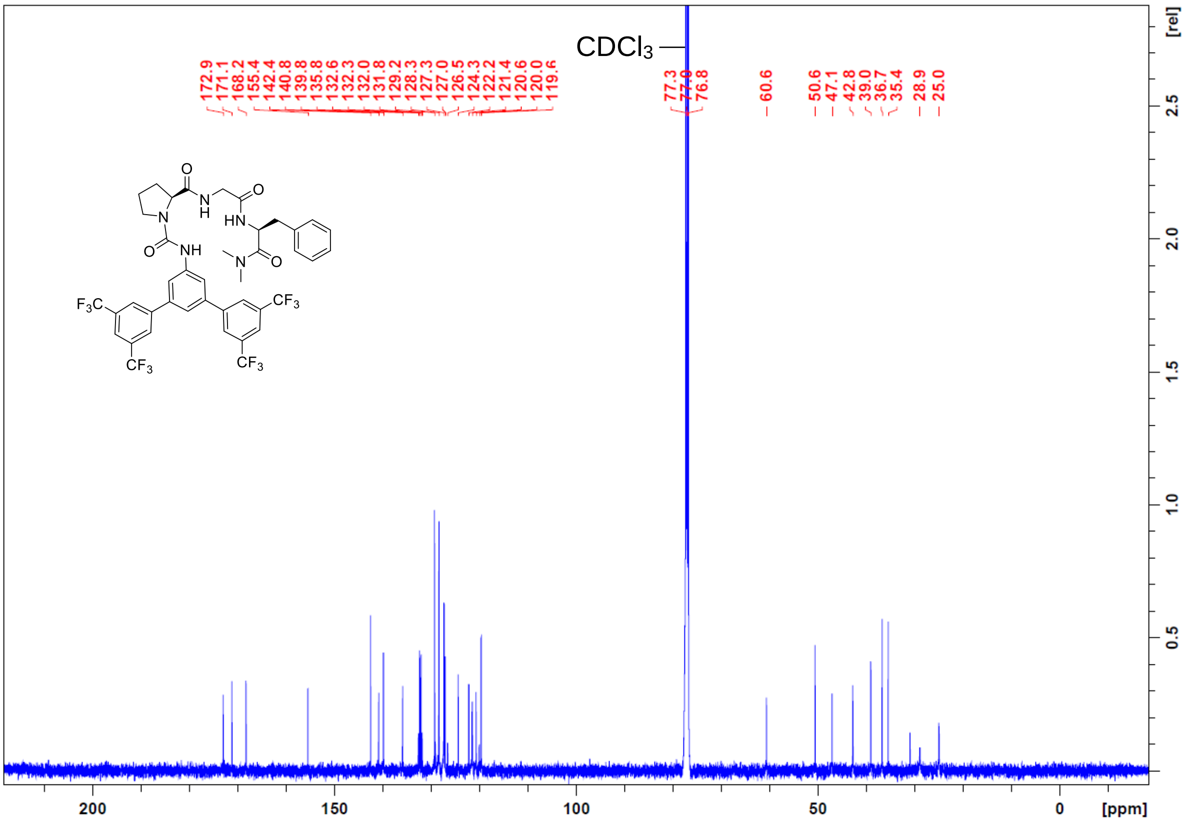

^{19}F NMR (377 MHz, $CDCl_3$): 3,5-[3,5-$(CF_3)_2C_6H_3]_2C_6H_3NHC(O)$-Pro-Gly-Phe-$NMe_2$ (**1c-Gly(*i*+1)**)

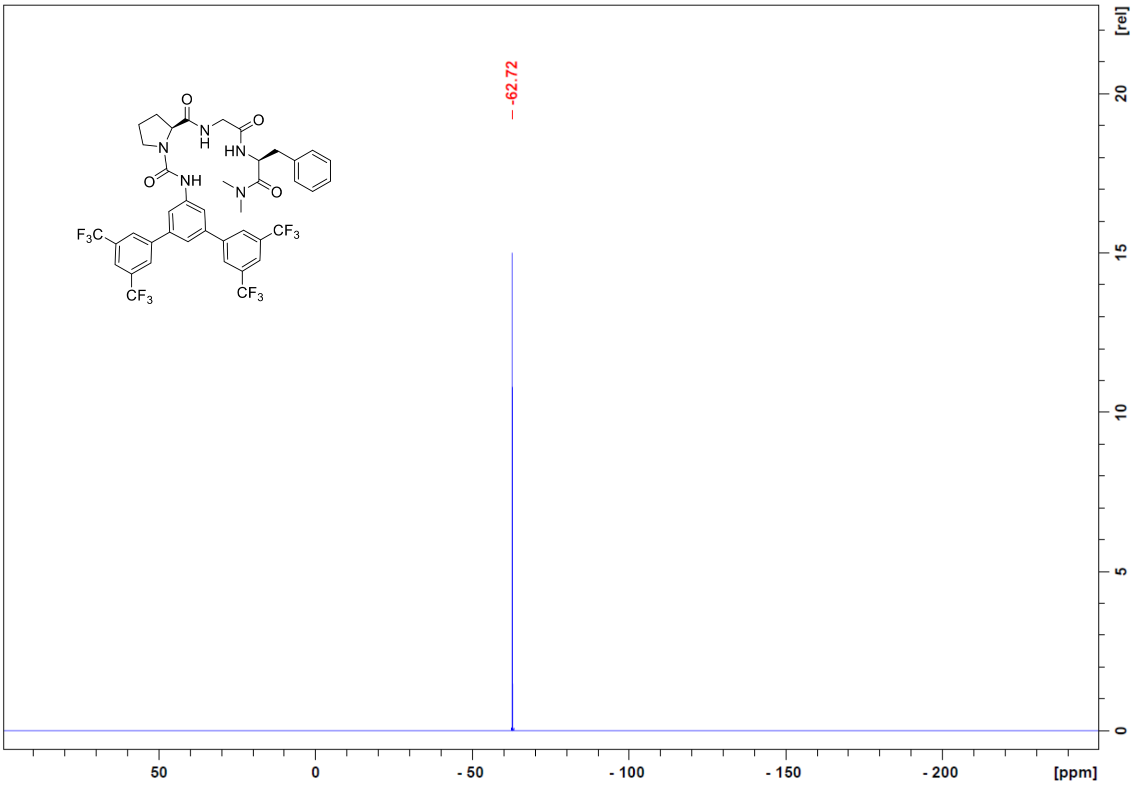

^{1}H NMR (400 MHz, CDCl3): 3,5-[3,5-(CF3)2C6H3]2C6H3NHC(O)-D-Pro-Gly-Phe-NMe2 (**1c-D-Pro(*i*)-Gly(*i*+1)**)

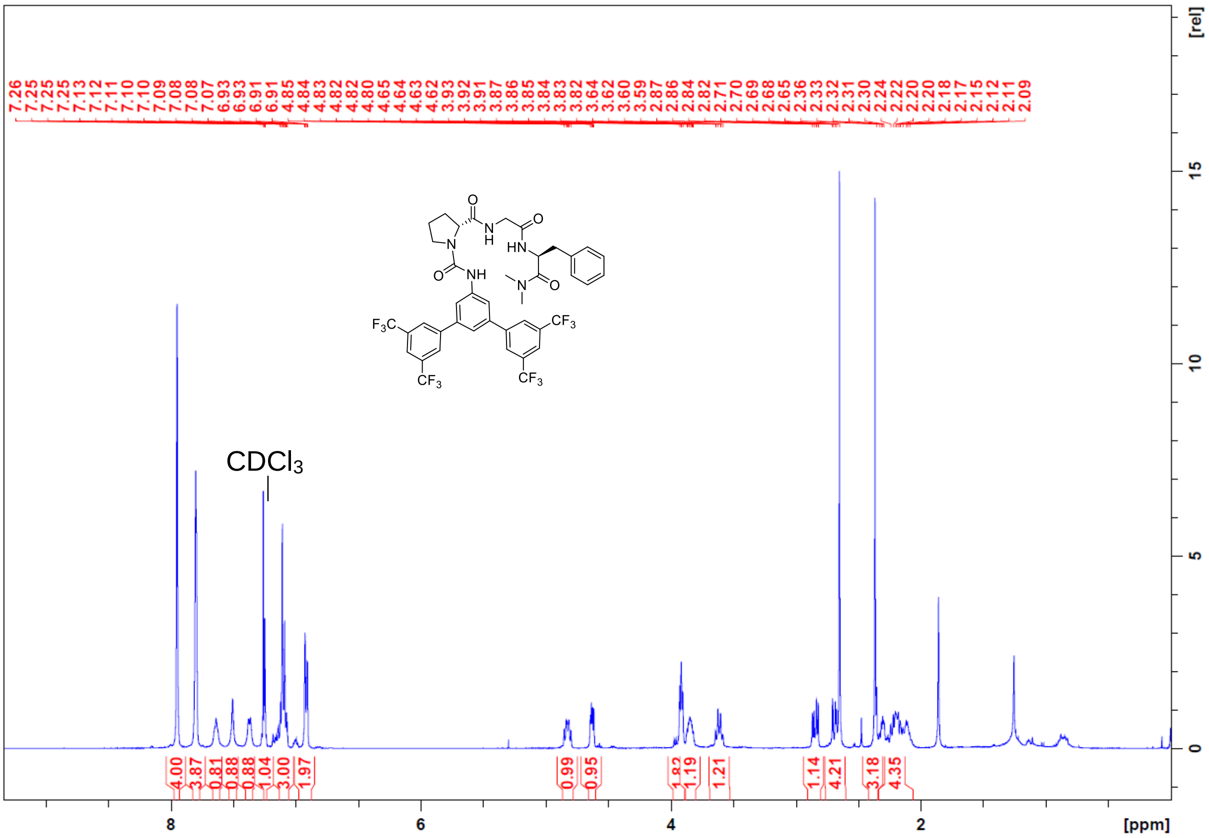

^{13}C NMR (101 MHz, CDCl3): 3,5-[3,5-(CF3)2C6H3]2C6H3NHC(O)-D-Pro-Gly-Phe-NMe2 (**1c-D-Pro(*i*)-Gly(*i*+1)**)

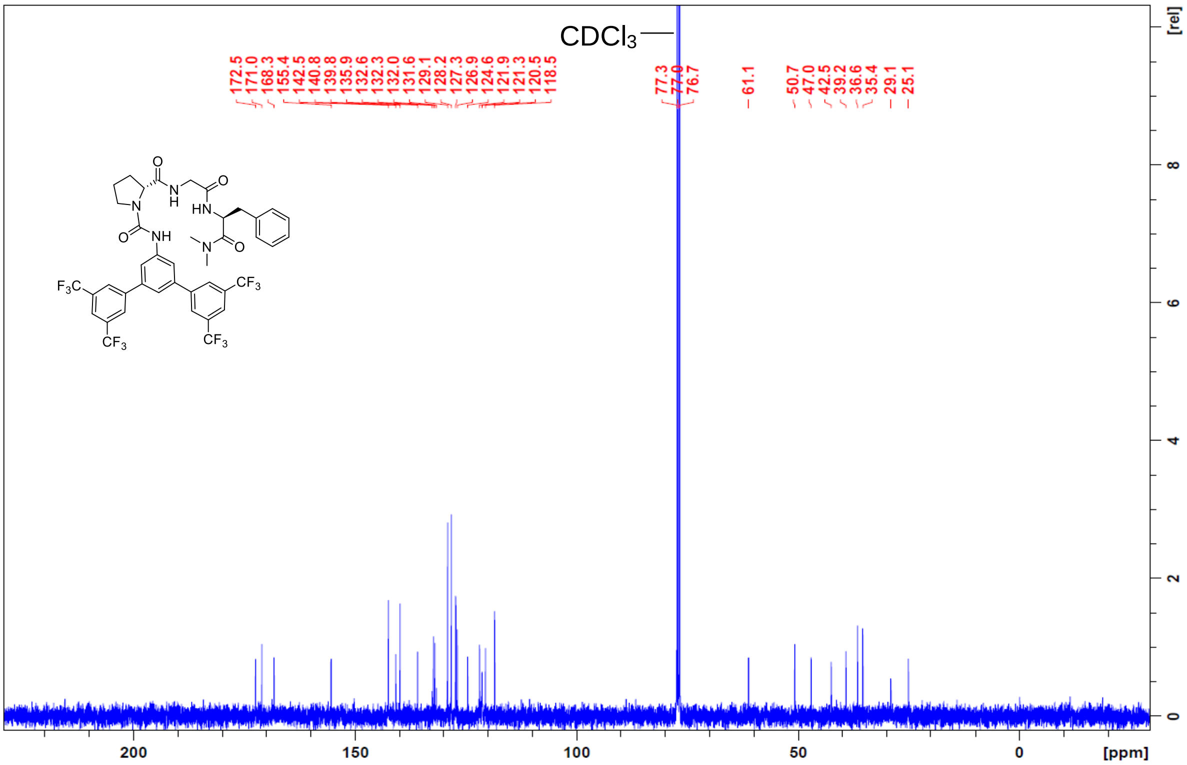

^{19}F NMR (377 MHz, $CDCl_3$): 3,5-[3,5-$(CF_3)_2C_6H_3]_2C_6H_3$NHC(O)-D-Pro-Gly-Phe-NMe_2 (**1c-D-Pro(*i*)-Gly(*i*+1)**)

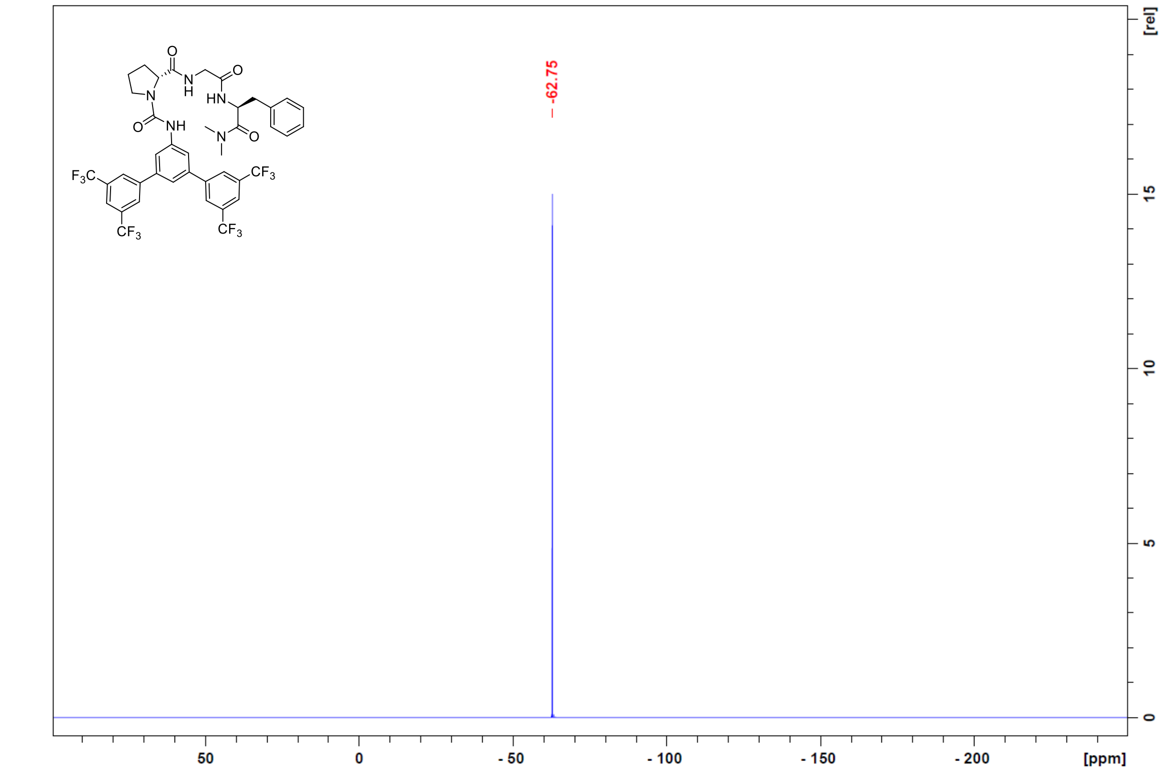

^{1}H NMR (500 MHz, CD_2Cl_2): 3,5-[3,5-$(CF_3)_2C_6H_3]_2C_6H_3$NHC(O)-flp-Gly-Phe-NMe_2 (**1d-Gly(*i*+1)**)

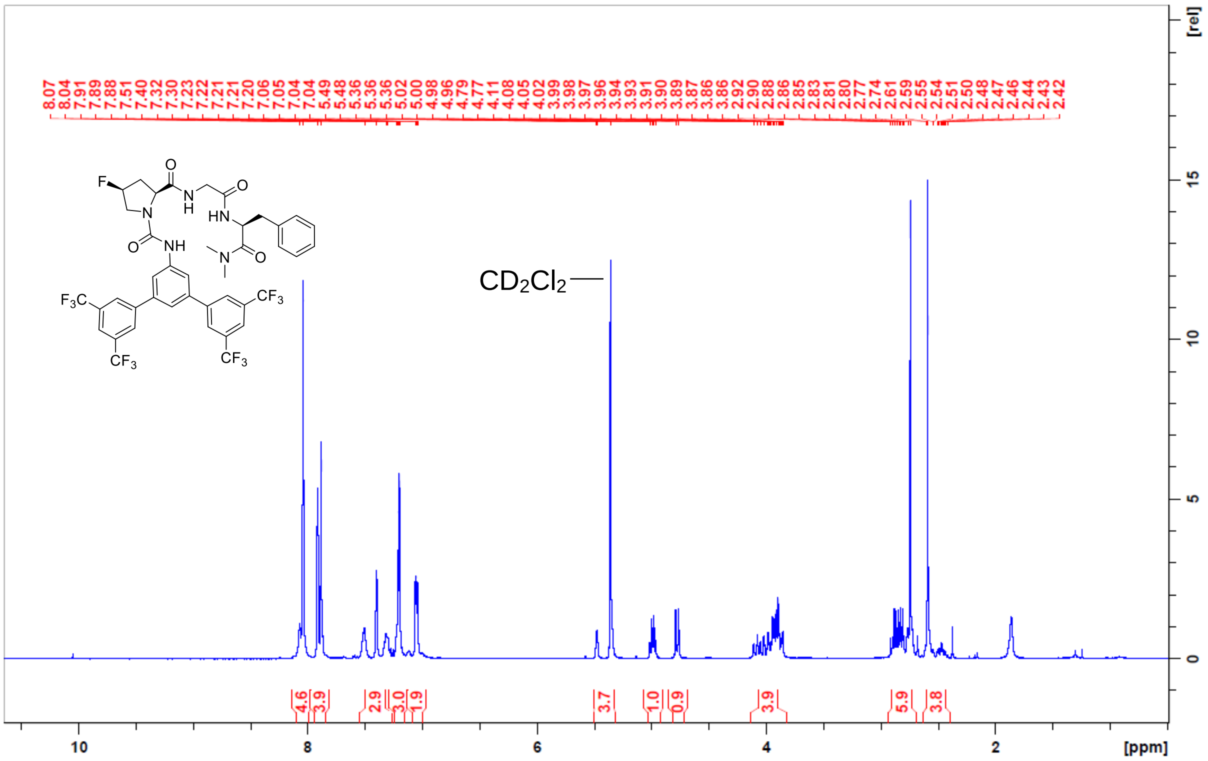

^{13}**C NMR** (126 MHz, CD_2Cl_2): 3,5-[3,5-$(CF_3)_2C_6H_3]_2C_6H_3$NHC(O)-flp-Gly-Phe-NMe_2 (**1d-Gly(*i*+1)**)

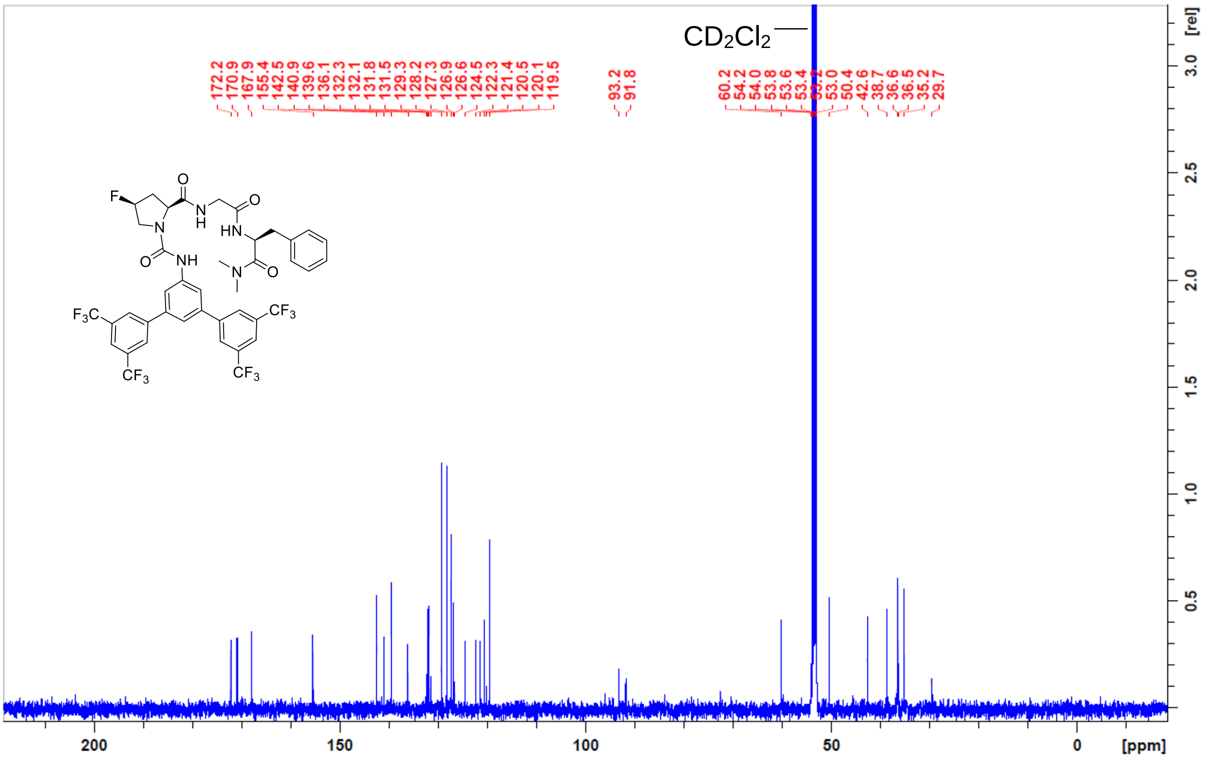

^{19}**F NMR** (470 MHz, CD_2Cl_2): 3,5-[3,5-$(CF_3)_2C_6H_3]_2C_6H_3$NHC(O)-flp-Gly-Phe-NMe_2 (**1d-Gly(*i*+1)**)

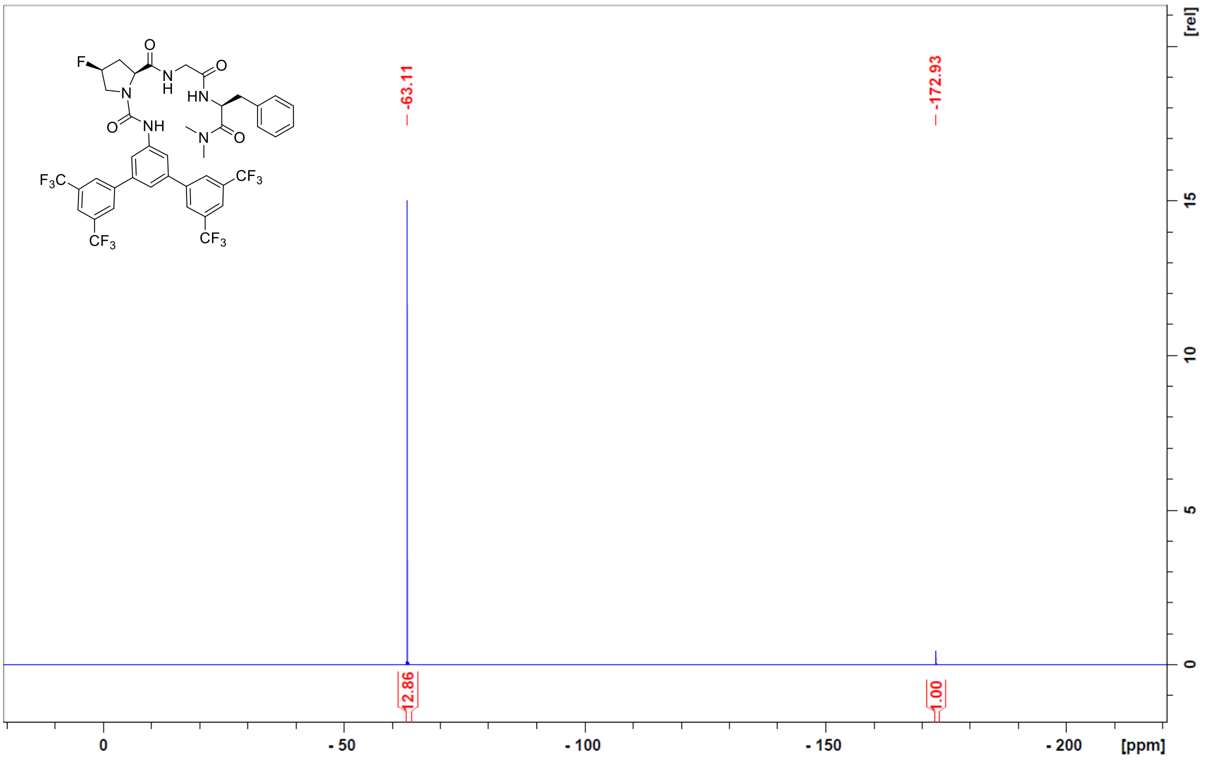

^{1}H NMR (400 MHz, CD_2Cl_2): 3,5-[3,5-$(CF_3)_2C_6H_3$]$_2C_6H_3$NHC(O)-flp-Gly-Phe-NMe_2 (**1d-Flp(*i*)-Gly(*i*+1)**)

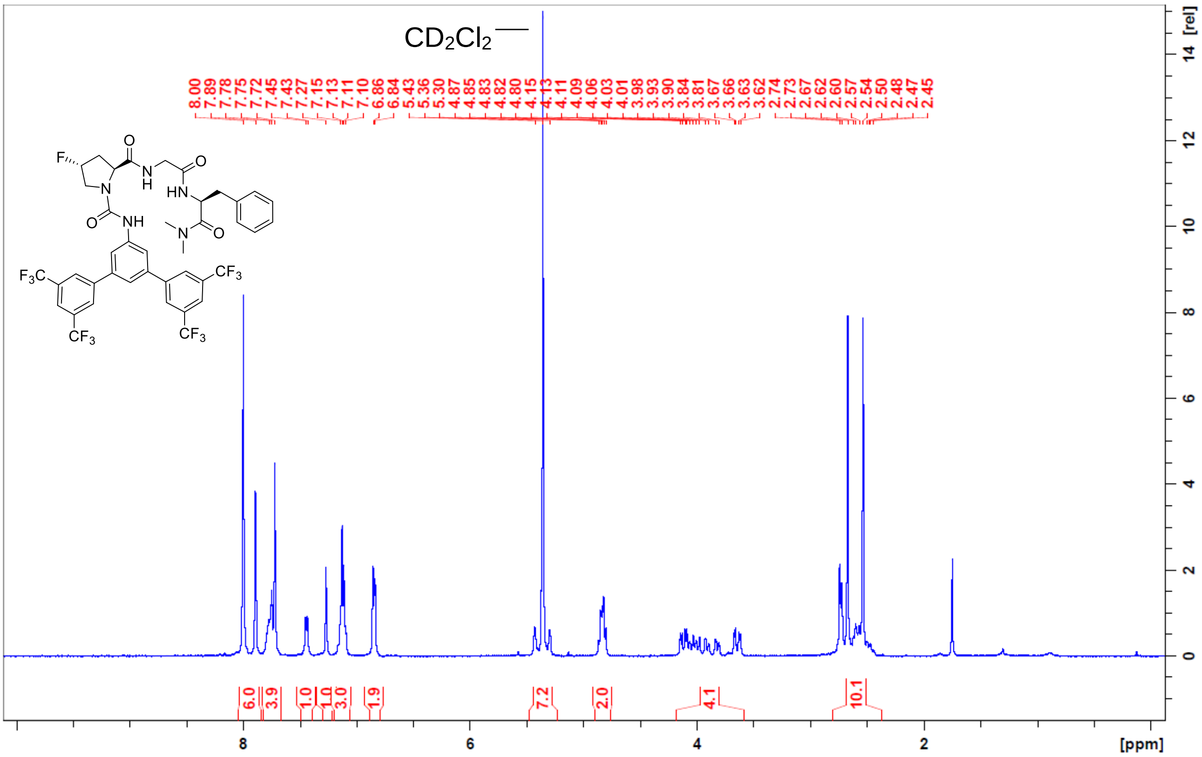

^{13}C NMR (101 MHz, CD_2Cl_2): 3,5-[3,5-$(CF_3)_2C_6H_3$]$_2C_6H_3$NHC(O)-flp-Gly-Phe-NMe_2 (**1d-Flp(*i*)-Gly(*i*+1)**)

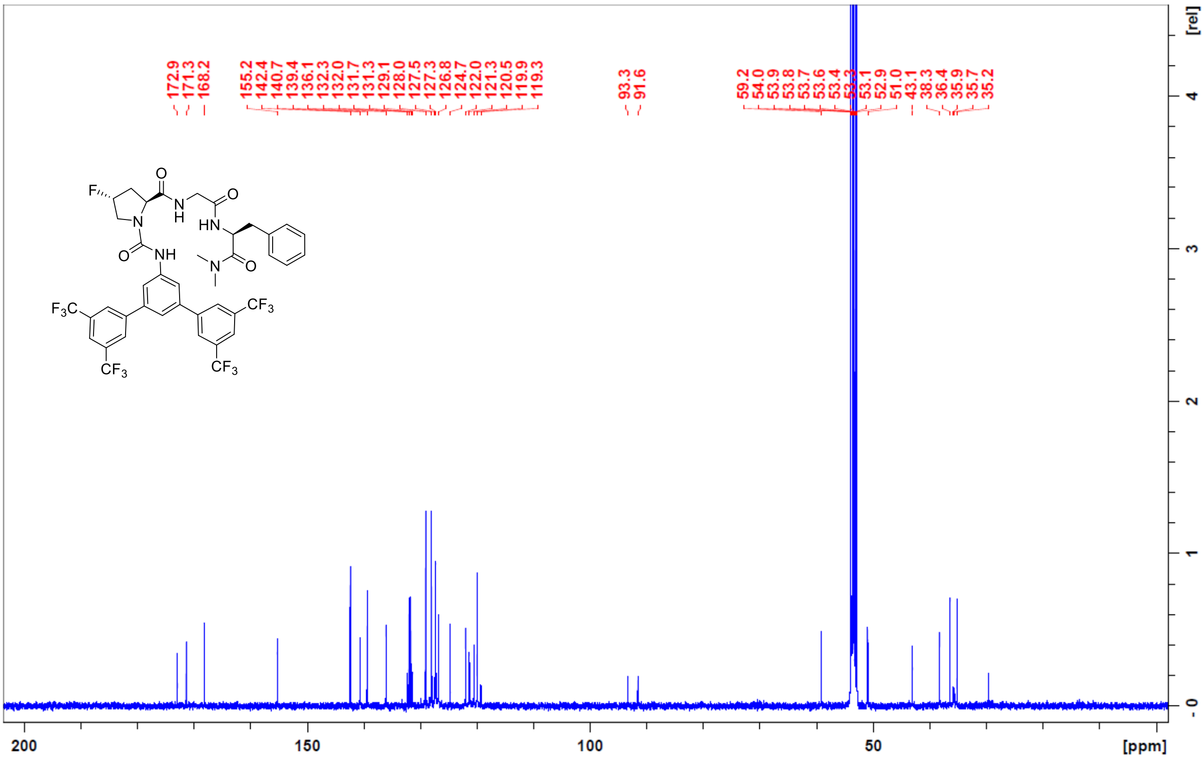

^{19}F NMR (376 MHz, CD_2Cl_2): 3,5-[3,5-$(CF_3)_2C_6H_3]_2C_6H_3$NHC(O)-flp-Gly-Phe-NMe_2 (**1d-Flp(*i*)-Gly(*i*+1)**)

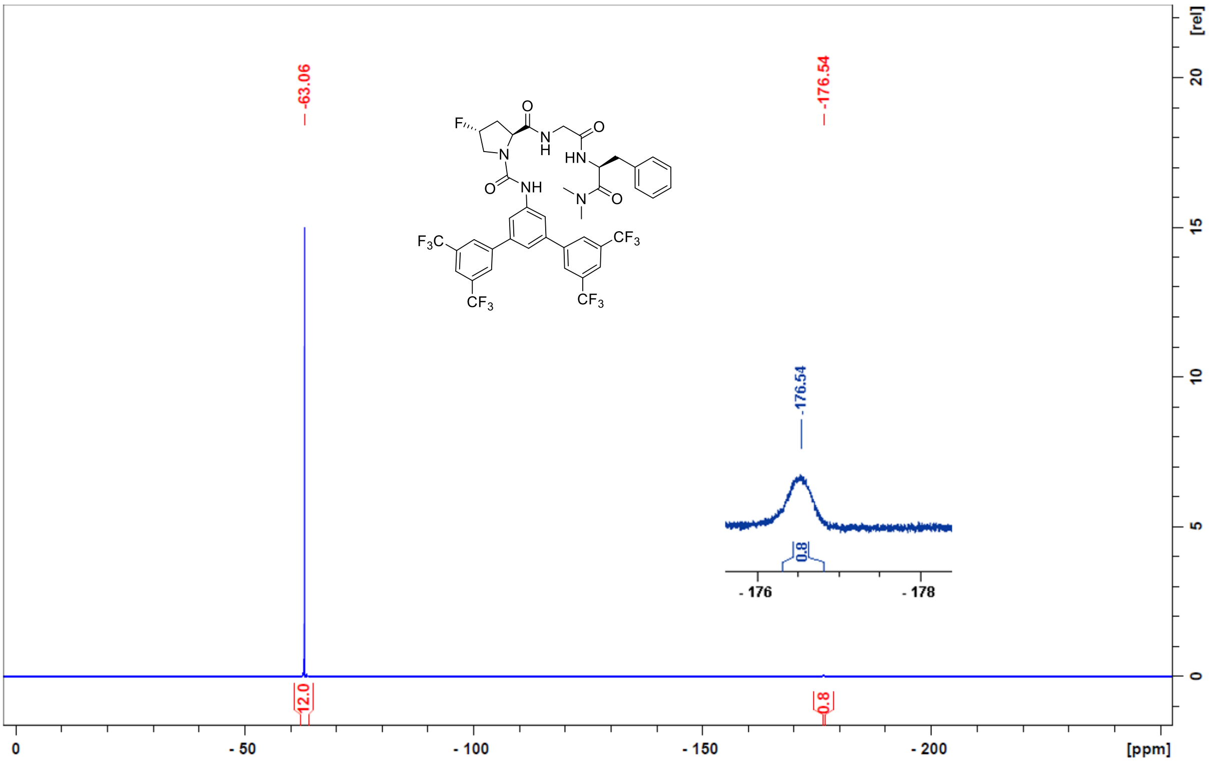

^{1}H NMR (600 MHz, CD_2Cl_2): 3,5-[3,5-$(CF_3)_2C_6H_3]_2C_6H_3{}^{15}$NHC(O)-flp-Acpc-Phe-$NEt_2$ (**1e-^{15}NH(*i*–1)**)

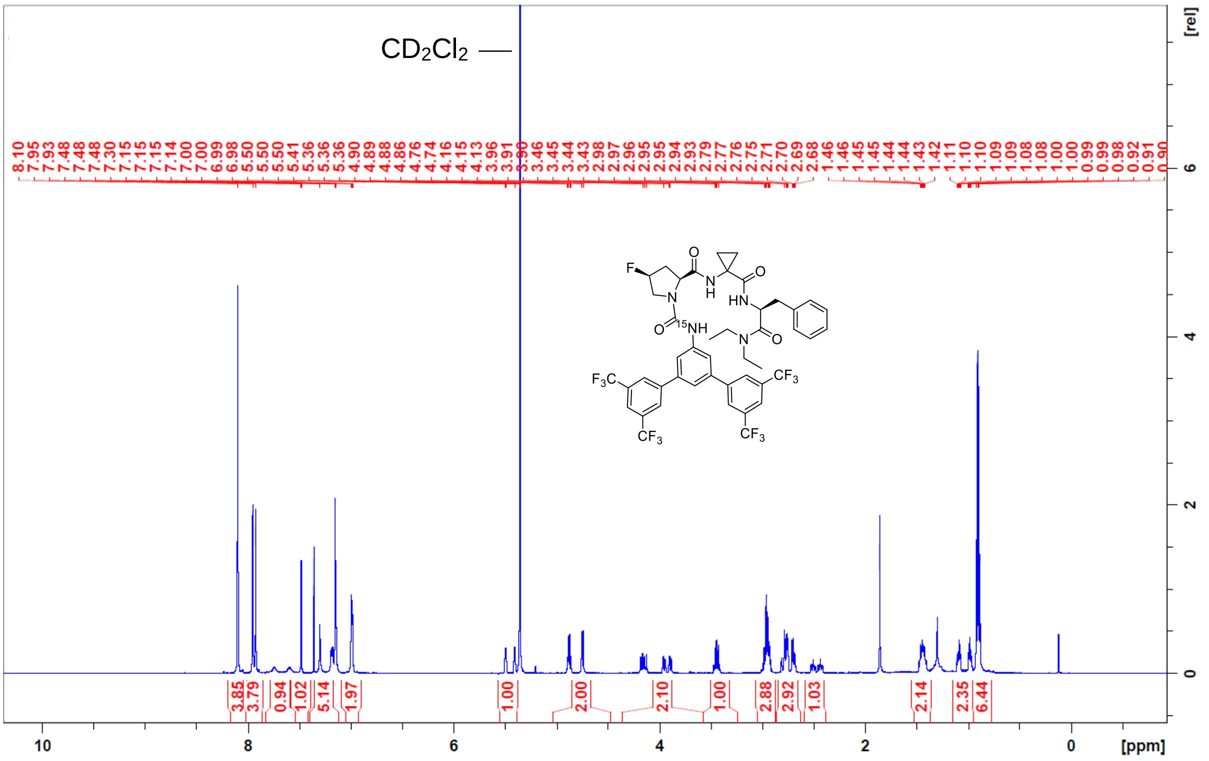

^{13}C NMR (151 MHz, CD_2Cl_2): 3,5-[3,5-$(CF_3)_2C_6H_3]_2C_6H_3{}^{15}$NHC(O)-flp-Acpc-[^{15}N]Phe-NEt_2 (**1e-^{15}NH(*i*–1)**)

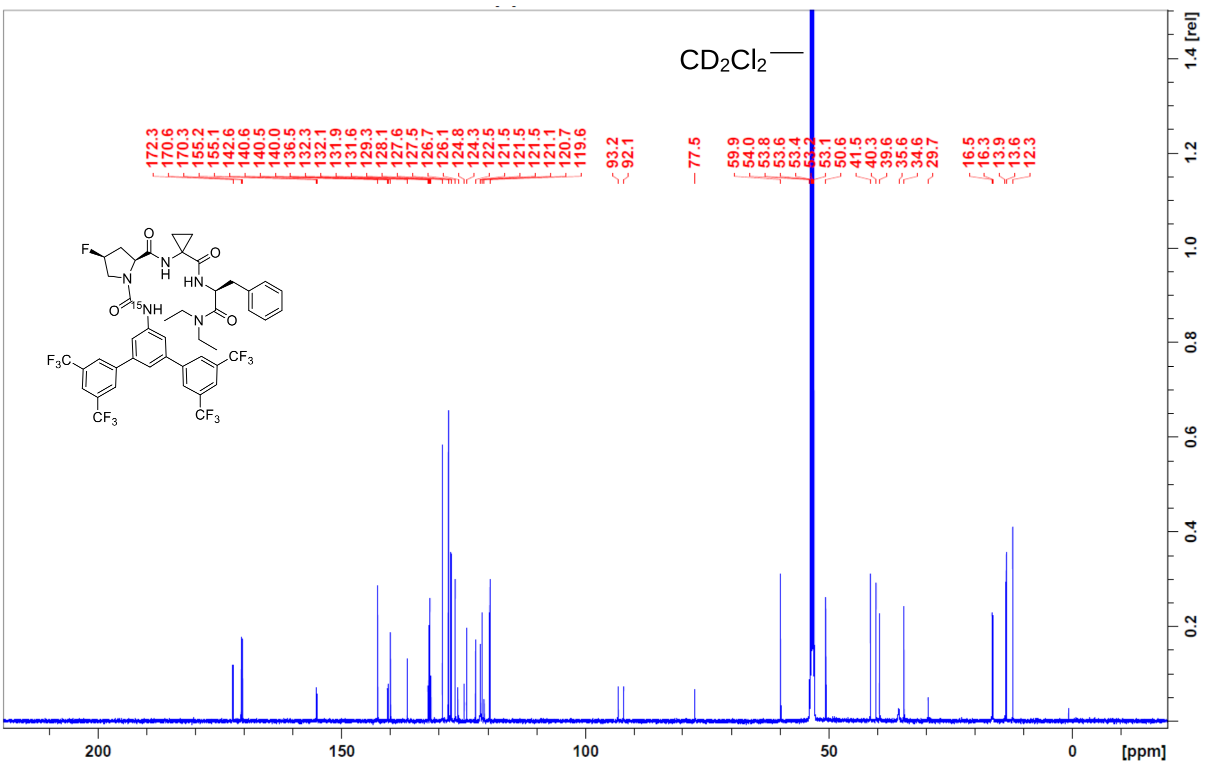

^{15}N NMR (120 MHz, CD_2Cl_2): 3,5-[3,5-$(CF_3)_2C_6H_3]_2C_6H_3{}^{15}$NHC(O)-flp-Acpc-Phe-$NEt_2$ (**1e-^{15}NH(*i*–1)**)

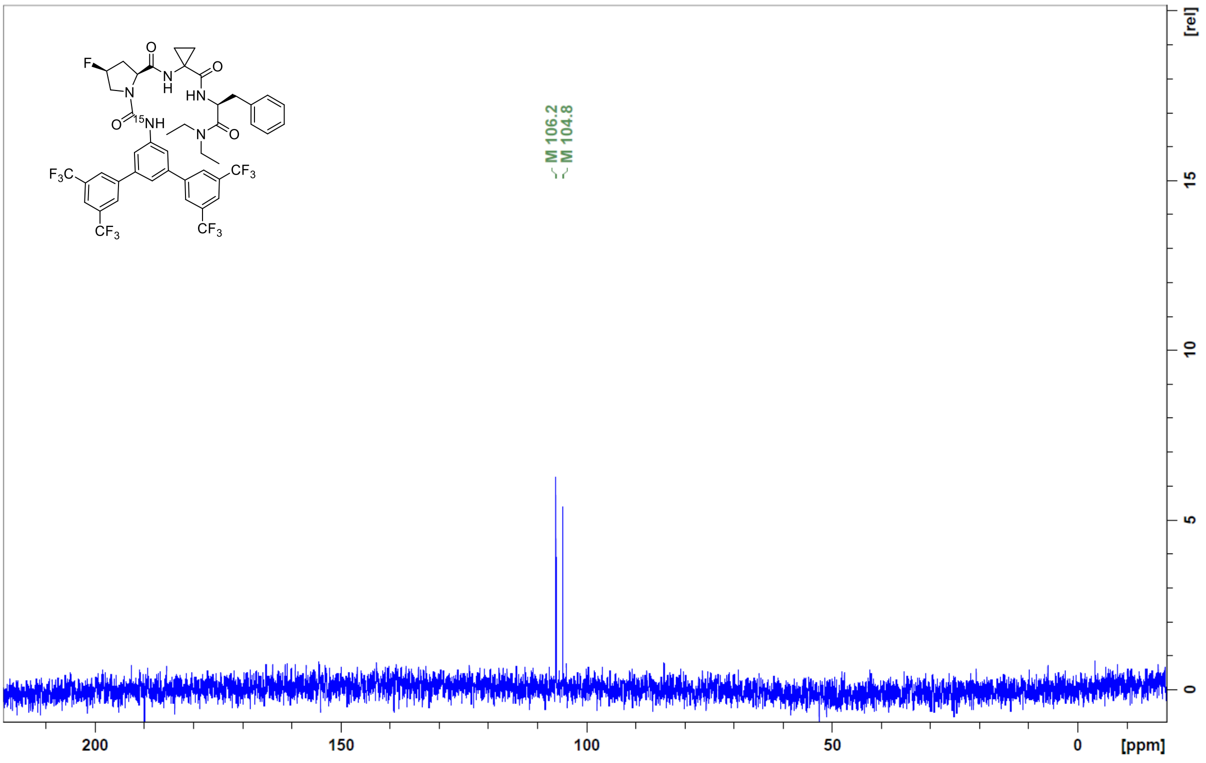

^{19}F{^{1}H} NMR (470 MHz, $CDCl_3$): 3,5-[3,5-$(CF_3)_2C_6H_3]_2C_6H_3{}^{15}$NHC(O)-flp-Acpc-Phe-$NEt_2$ (**1e-^{15}NH(*i*–1)**)

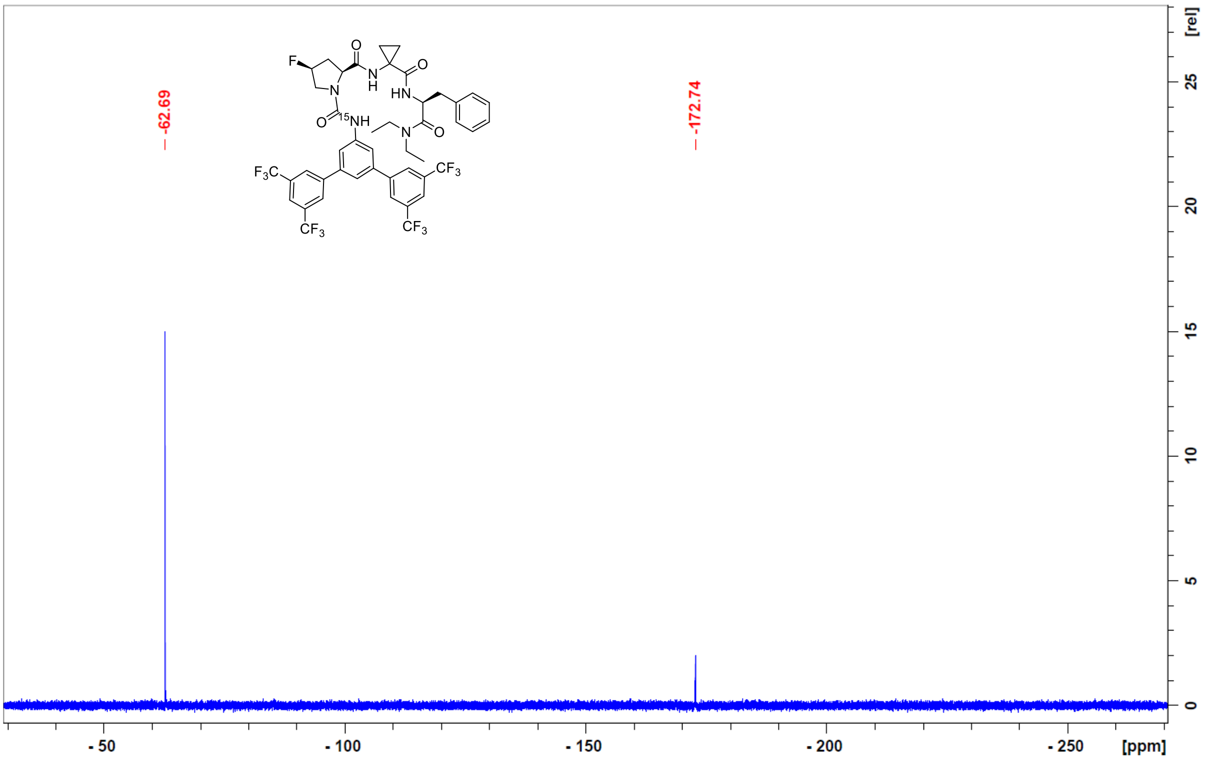

^{1}H NMR (600 MHz, CD_2Cl_2): 3,5-[3,5-$(CF_3)_2C_6H_3]_2C_6H_3$NHC(O)-flp-Acpc-[^{15}N]Phe-NEt_2 (**1e-^{15}NH(*i*+2)**)

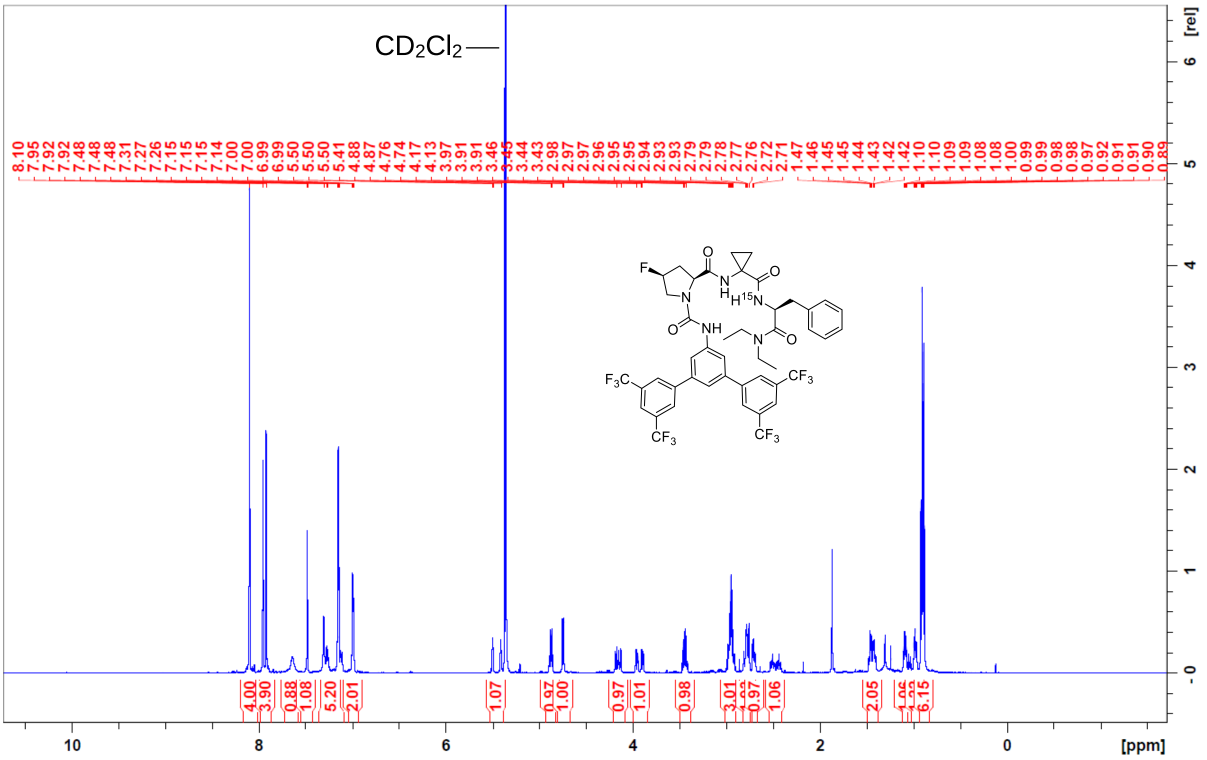

^{13}C NMR (151 MHz, CD_2Cl_2): 3,5-[3,5-$(CF_3)_2C_6H_3]_2C_6H_3$NHC(O)-flp-Acpc-[^{15}N]Phe-NEt$_2$ (**1e-^{15}NH(*i*+2)**)

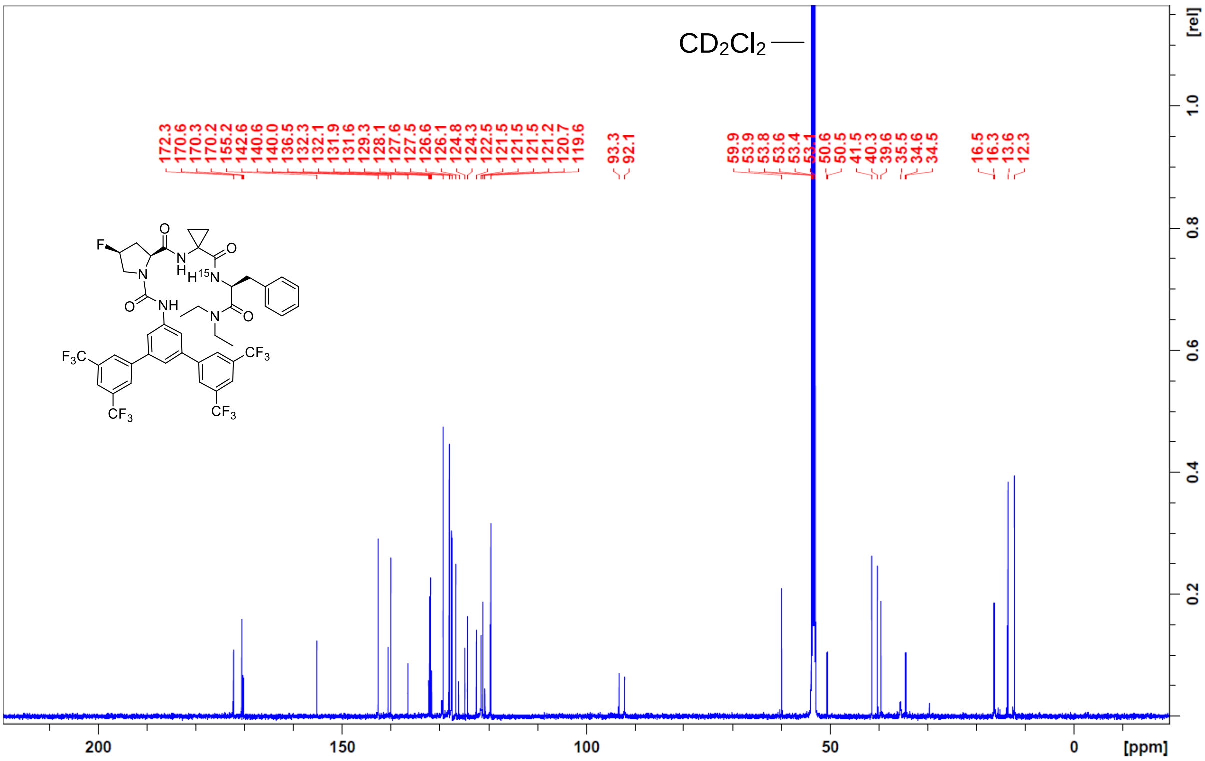

^{15}N NMR (120 MHz, CD_2Cl_2): 3,5-[3,5-$(CF_3)_2C_6H_3]_2C_6H_3$NHC(O)-flp-Acpc-[^{15}N]Phe-NEt$_2$ (**1e-^{15}NH(*i*+2)**)

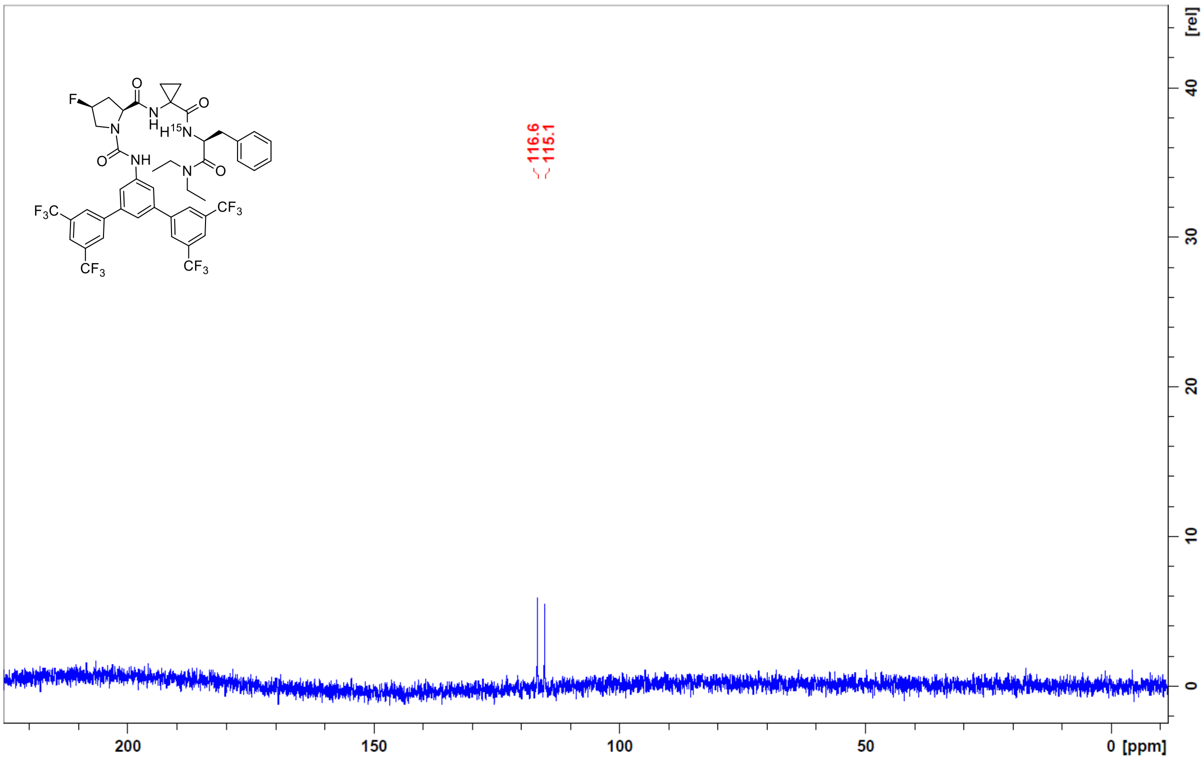

$^{19}F\{^{1}H\}$ NMR (470 MHz, $CDCl_3$): 3,5-[3,5-$(CF_3)_2C_6H_3]_2C_6H_3$NHC(O)-flp-Acpc-[^{15}N]Phe-NEt_2 (**1e-^{15}NH(*i*+2)**)

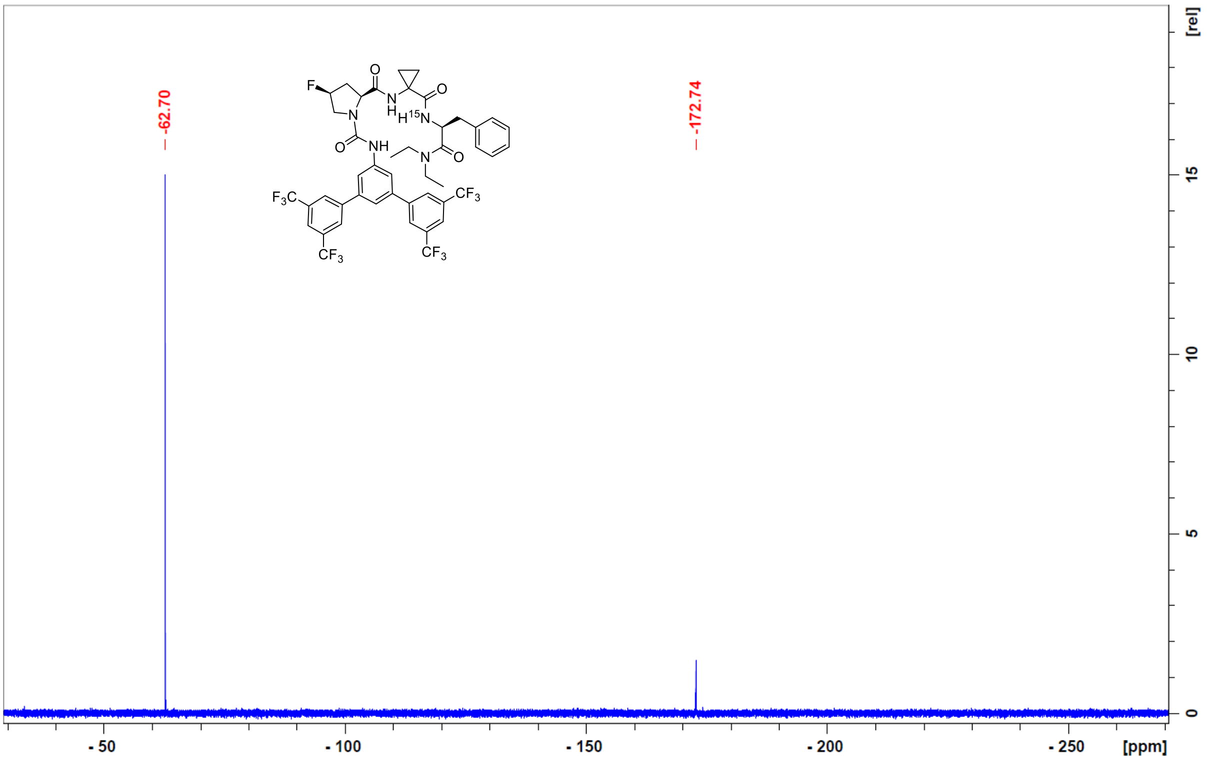

9.2. NMR spectra of substrates, their intermediates and products

^1H NMR (400 MHz, $CDCl_3$): *trans*-4-(dibenzylamino)tetrahydrofuran-3-ol (*rac*-**S10**)

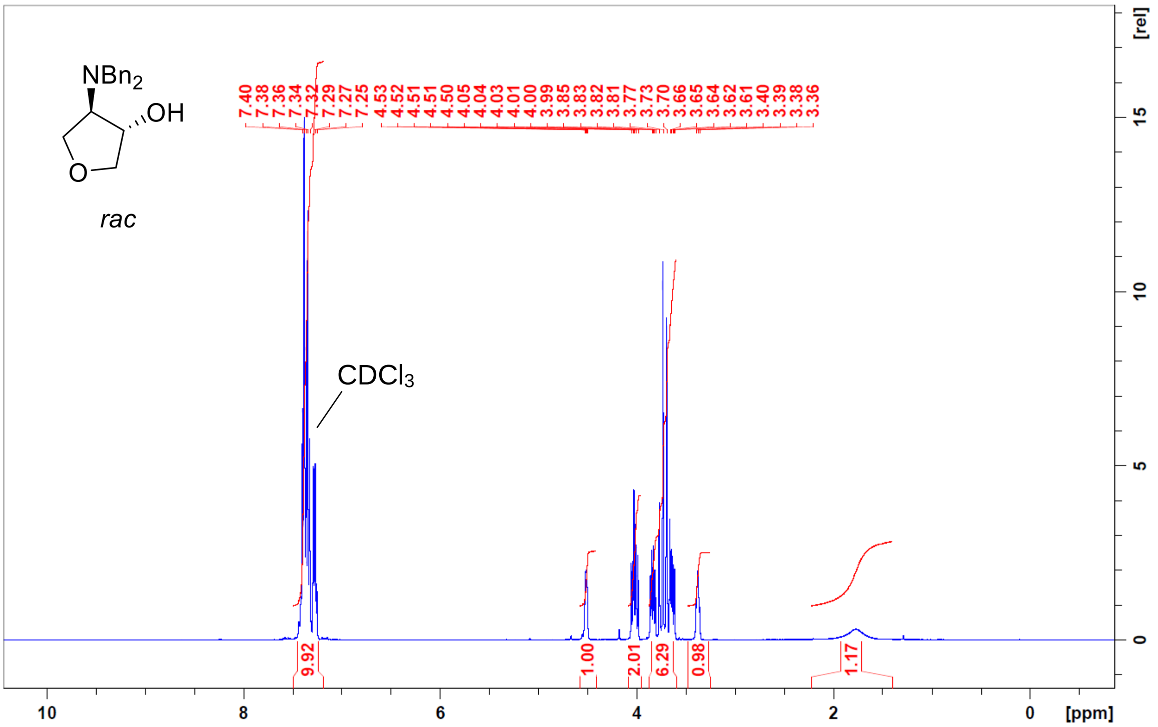

^{13}C NMR (101 MHz, $CDCl_3$): *trans*-4-(dibenzylamino)tetrahydrofuran-3-ol (*rac*-**S10**)

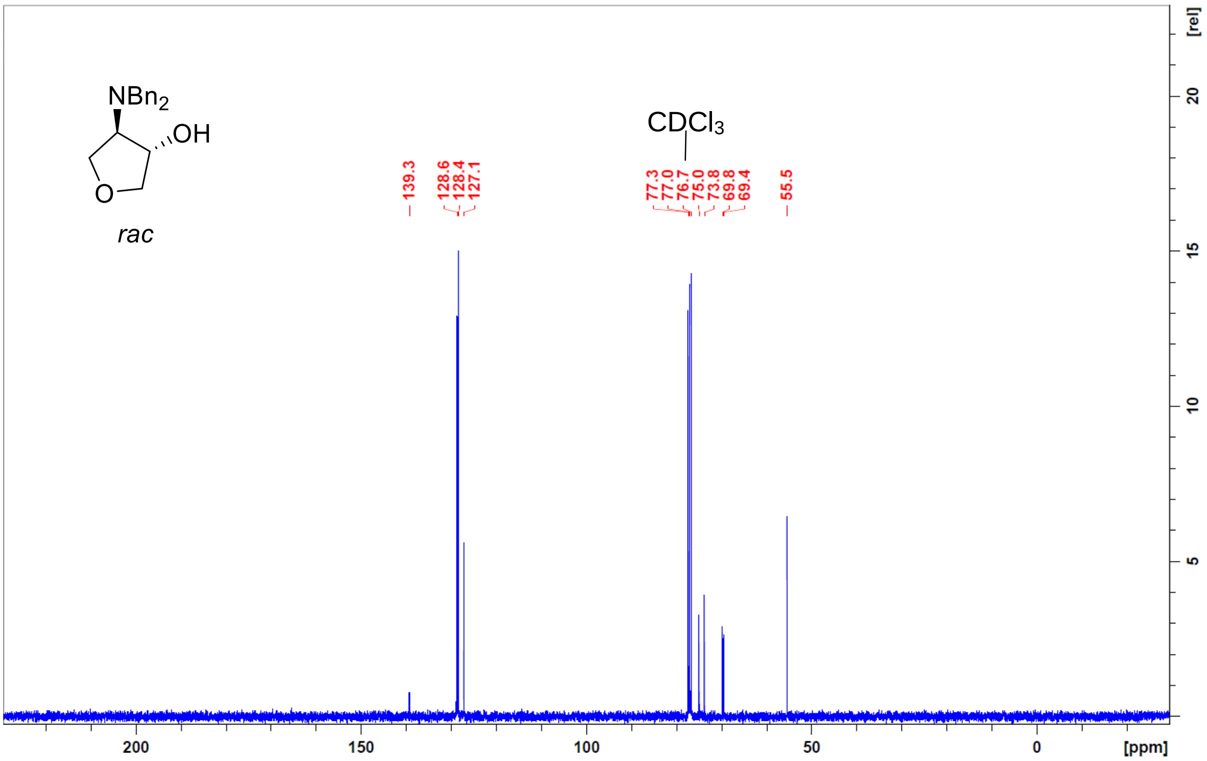

^{1}H NMR (400 MHz, $CDCl_3$): *rac*-2-(dibenzylamino)cycloheptan-1-ol (*rac*-**S12**)

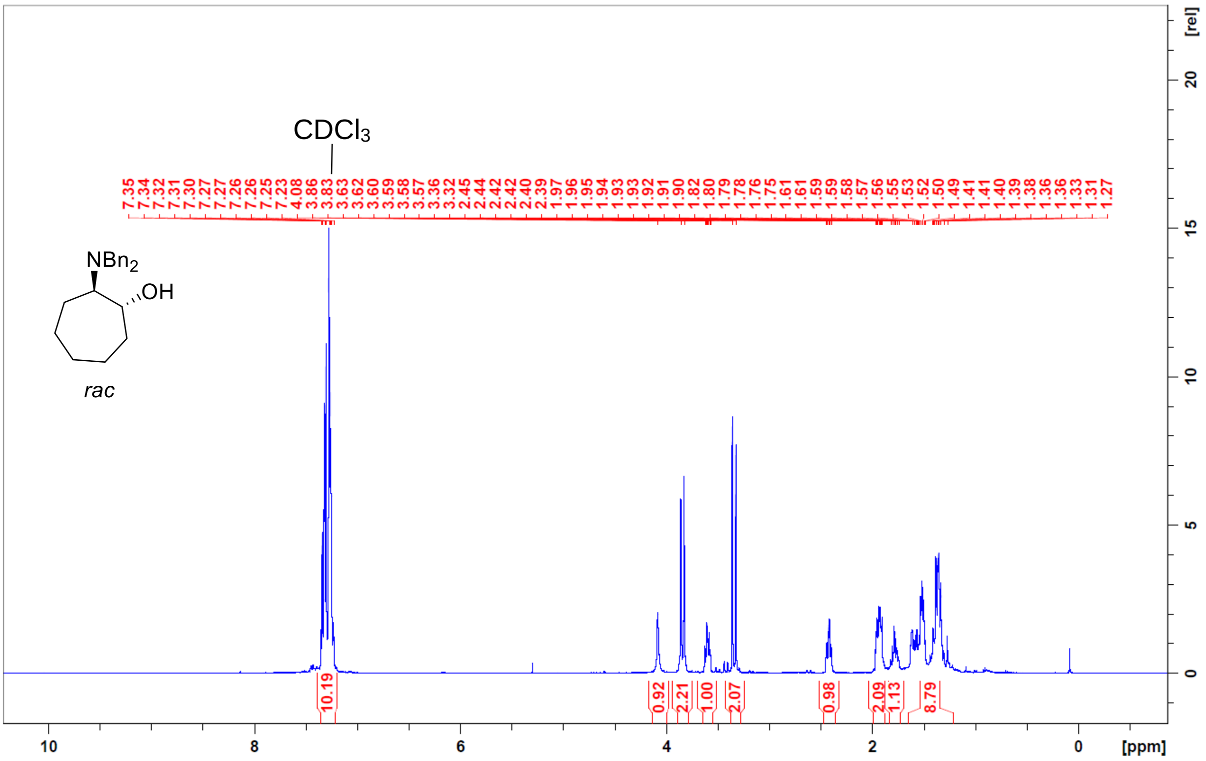

^{13}C NMR (101 MHz, $CDCl_3$): *rac*-2-(dibenzylamino)cycloheptan-1-ol (*rac*-**S12**)

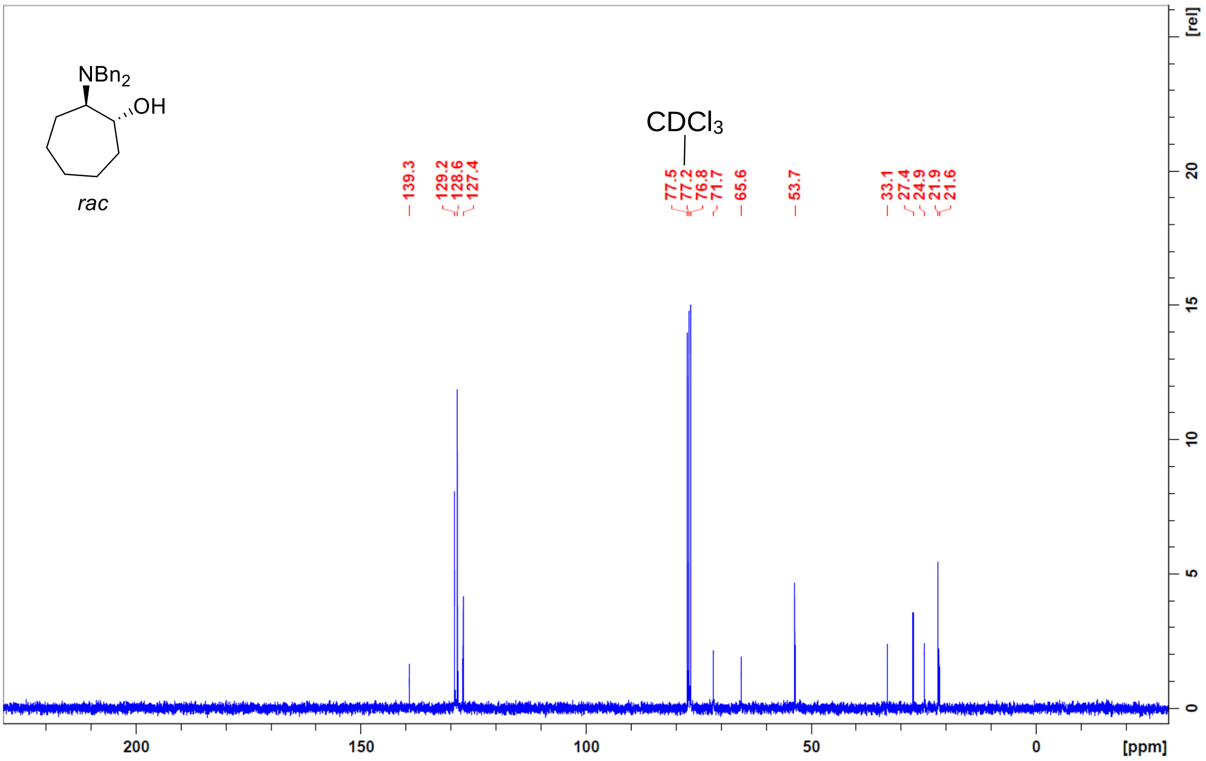

^{1}H NMR (400 MHz, $CDCl_3$): *trans*-*N*,*N*-dibenzyl-4-bromotetrahydrofuran-3-amine (*rac*-**2c**)

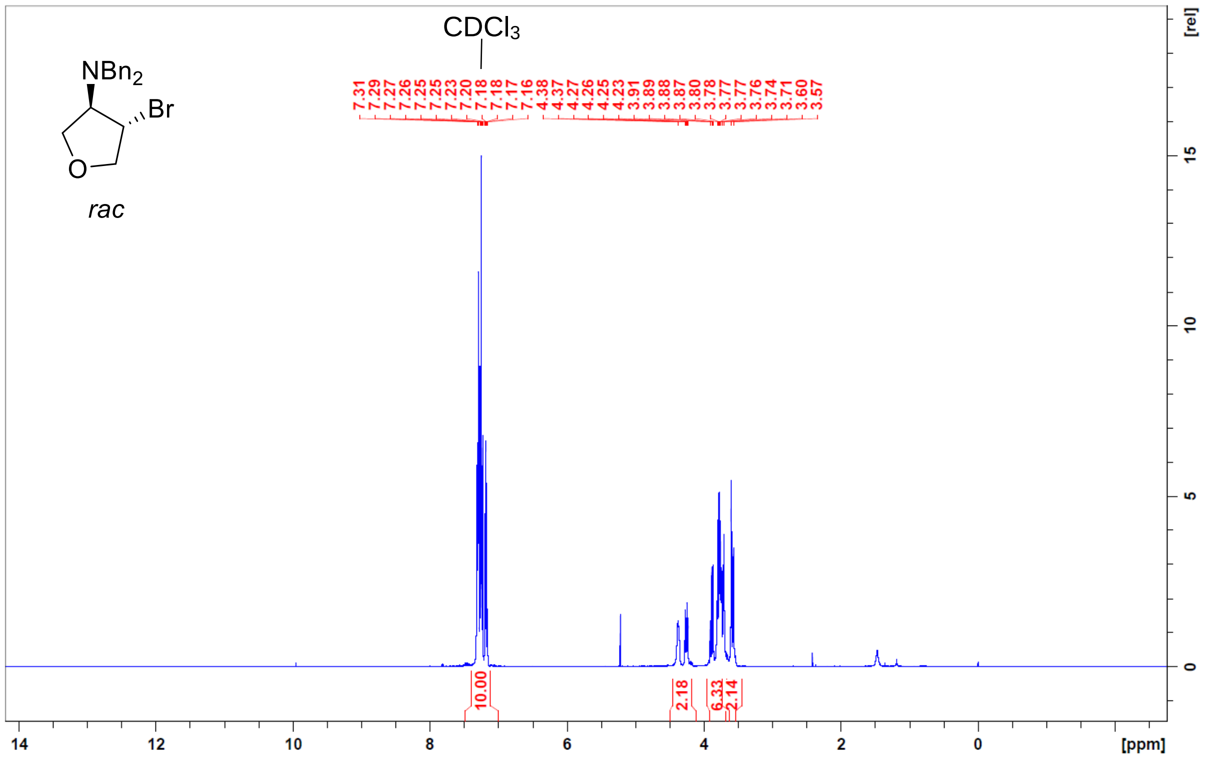

^{13}C NMR (101 MHz, $CDCl_3$): *trans*-*N*,*N*-dibenzyl-4-bromotetrahydrofuran-3-amine (*rac*-**2c**)

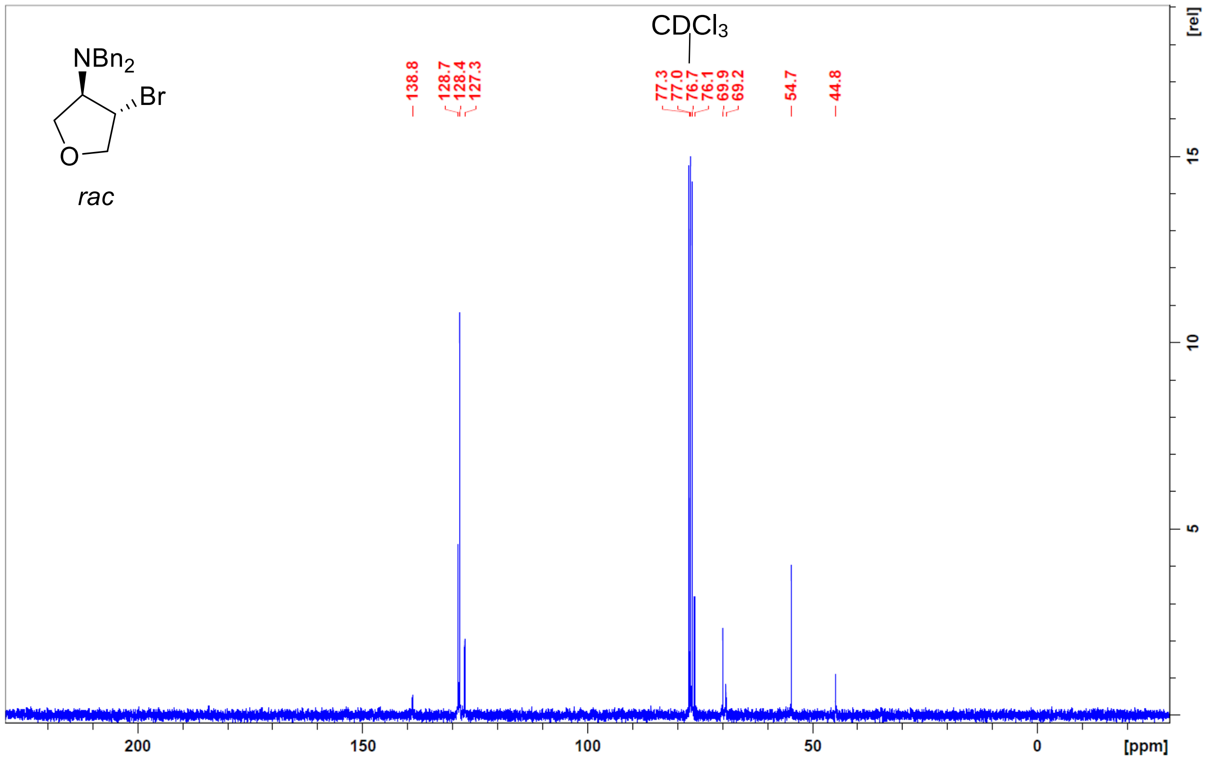

^{1}H NMR (500 MHz, $CDCl_3$): *tert*-butyl *trans*-3-bromo-4-(dibenzylamino)pyrrolidine-1-carboxylate (*rac*-**2d**)

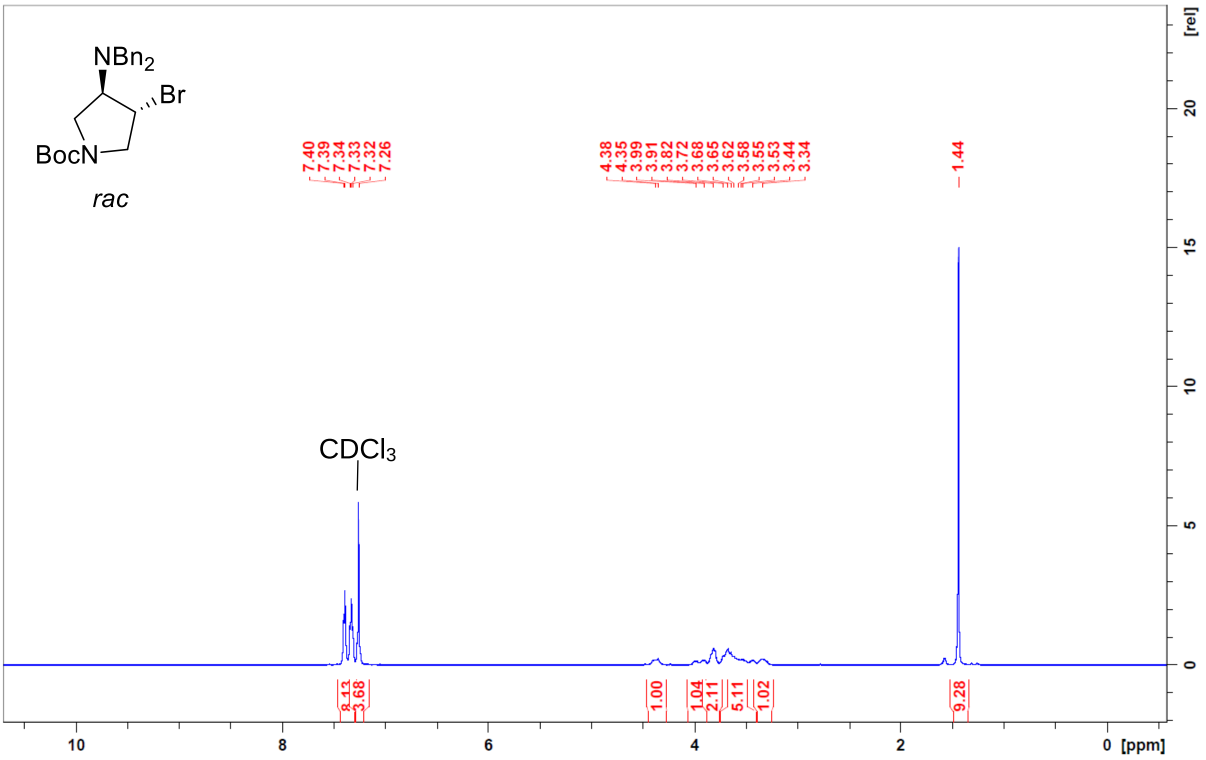

^{13}C NMR (126 MHz, $CDCl_3$): *tert*-butyl *trans*-3-bromo-4-(dibenzylamino)pyrrolidine-1-carboxylate (*rac*-**2d**)

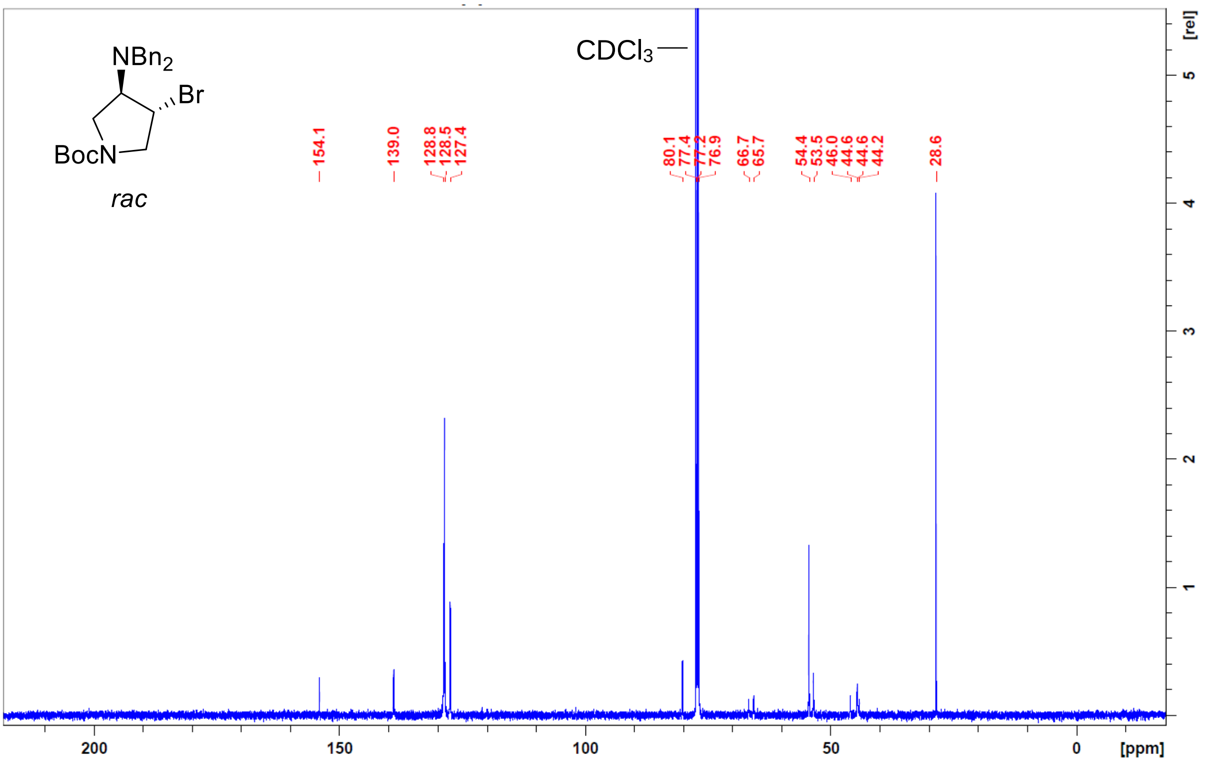

^{1}H NMR (400 MHz, $CDCl_3$): *trans-N,N*-dibenzyl-2-bromocyclohepatn-1-amine (*rac*-**2e**)

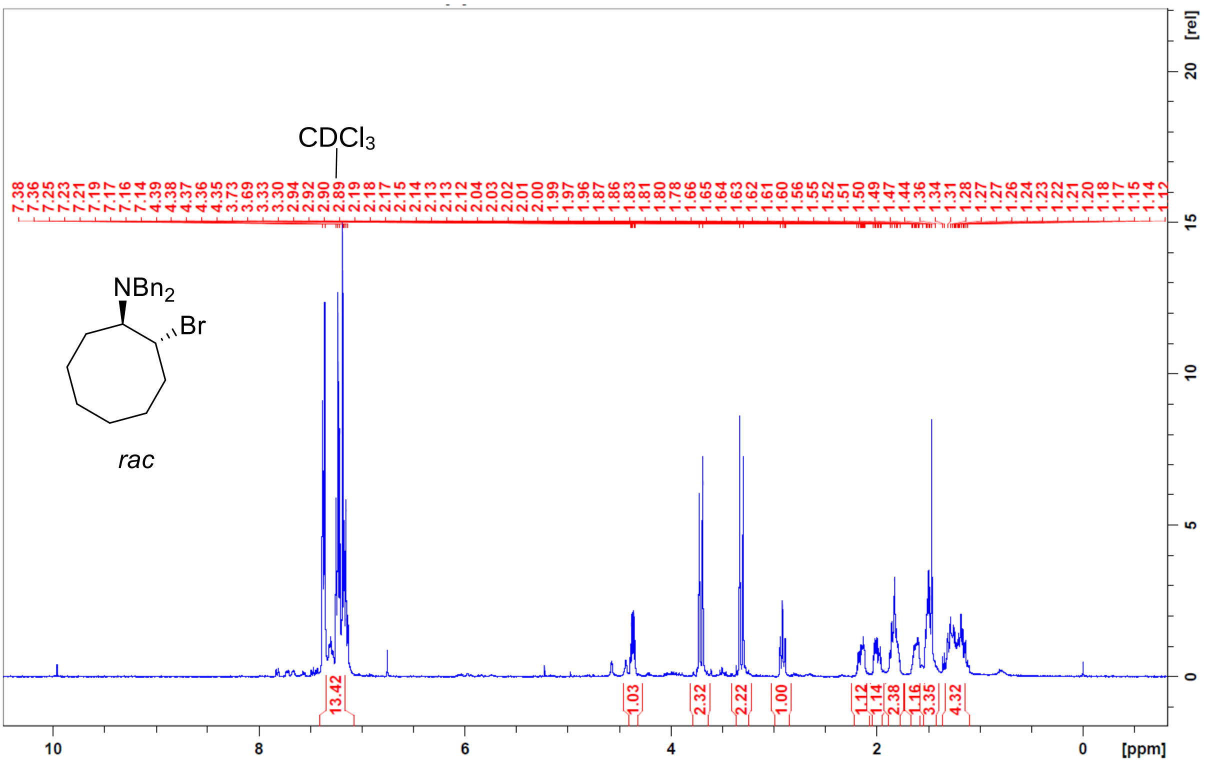

^{13}C NMR (126 MHz, $CDCl_3$): *trans-N,N*-dibenzyl-2-bromocyclohepatn-1-amine (*rac*-**2e**)

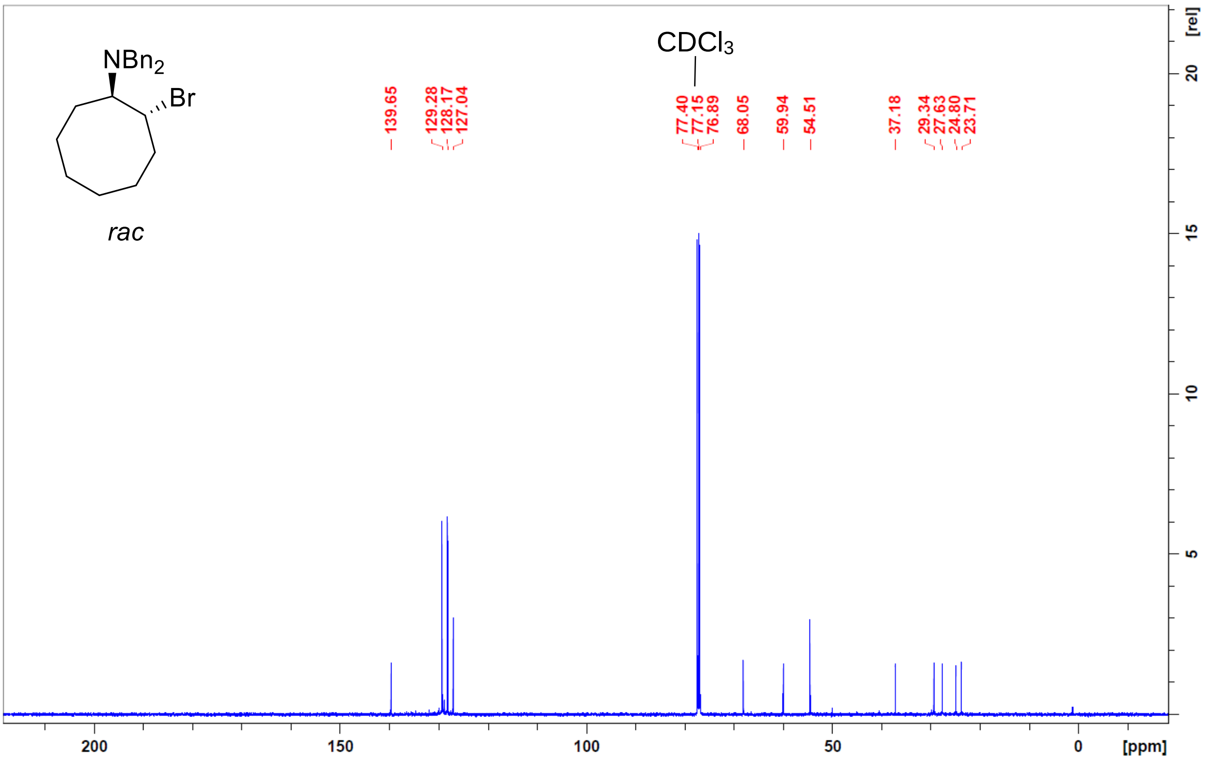

^{1}H NMR (400 MHz, $CDCl_3$): (3*S*,4*R*)-*N*,*N*-dibenzyl-4-fluorotetrahydrofuran-3-amine (3*S*,4*R*-**3c**)

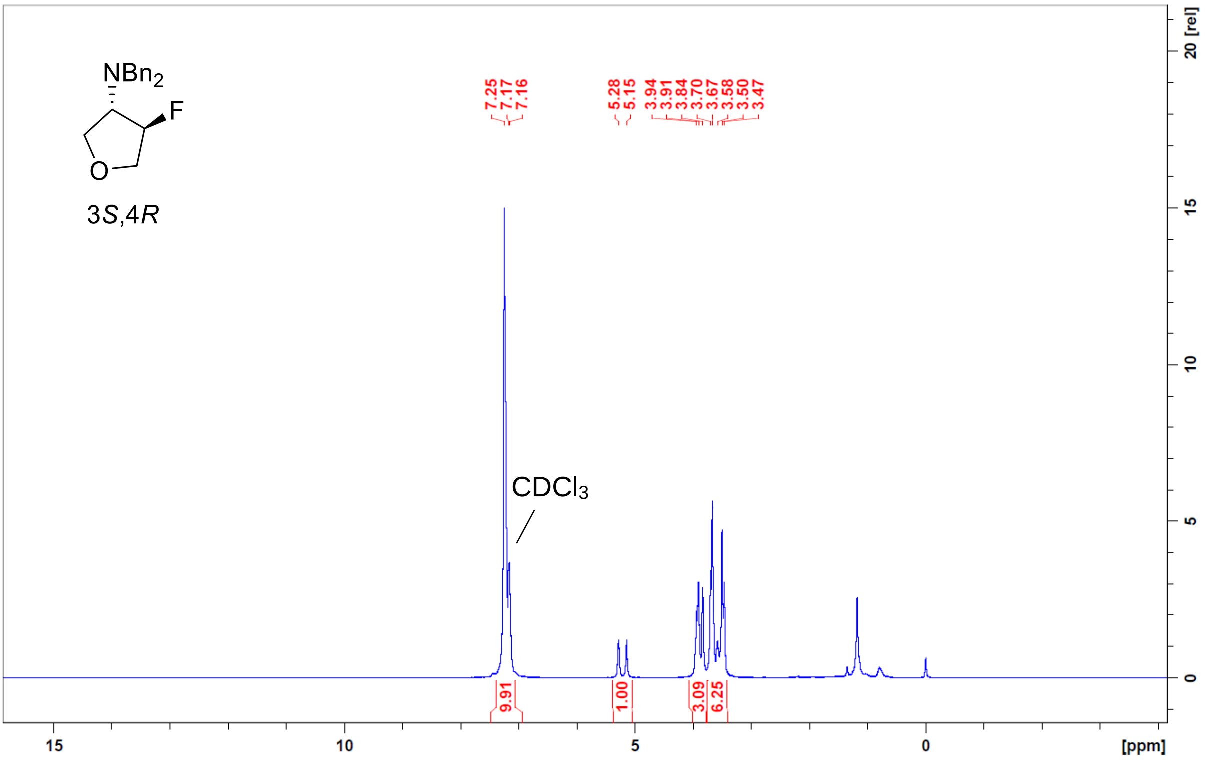

^{13}C NMR (101 MHz, $CDCl_3$): (3*S*,4*R*)-*N*,*N*-dibenzyl-4-fluorotetrahydrofuran-3-amine (**3c**)

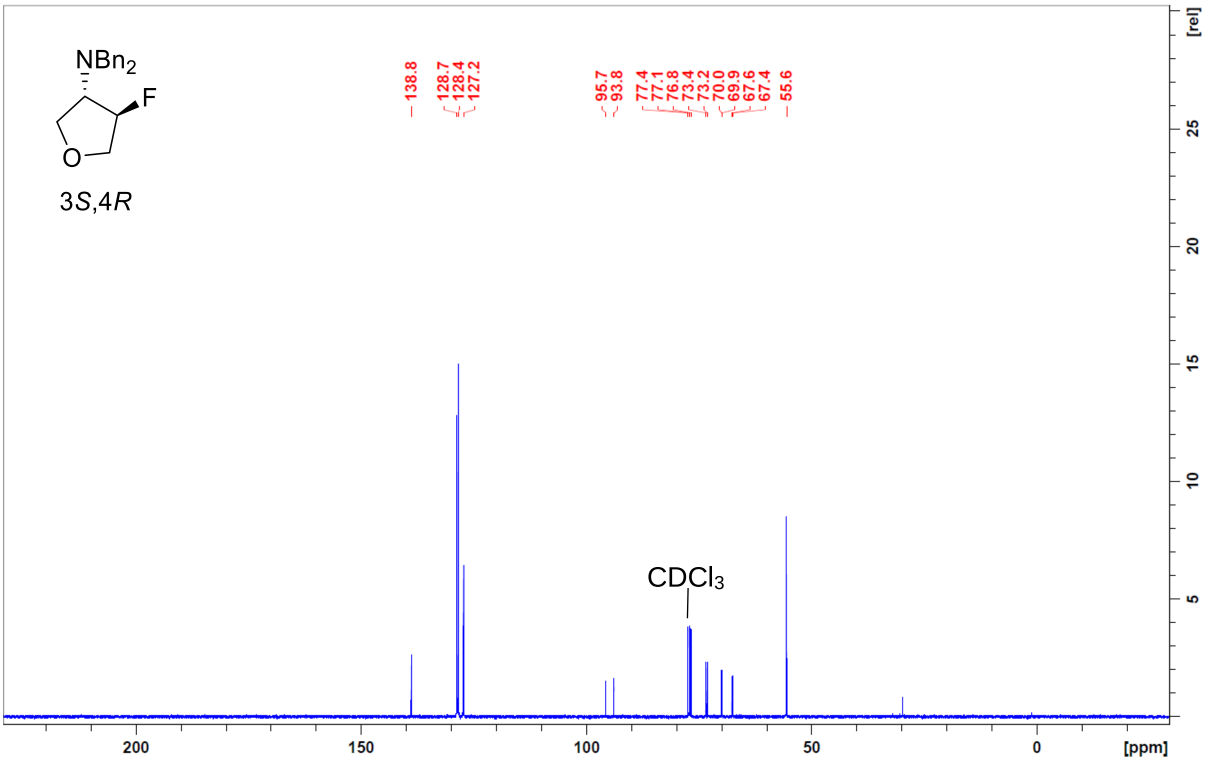

^{19}F NMR (377 MHz, $CDCl_3$): (3*S*,4*R*)-*N*,*N*-Dibenzyl-4-fluorotetrahydrofuran-3-amine (**3c**)

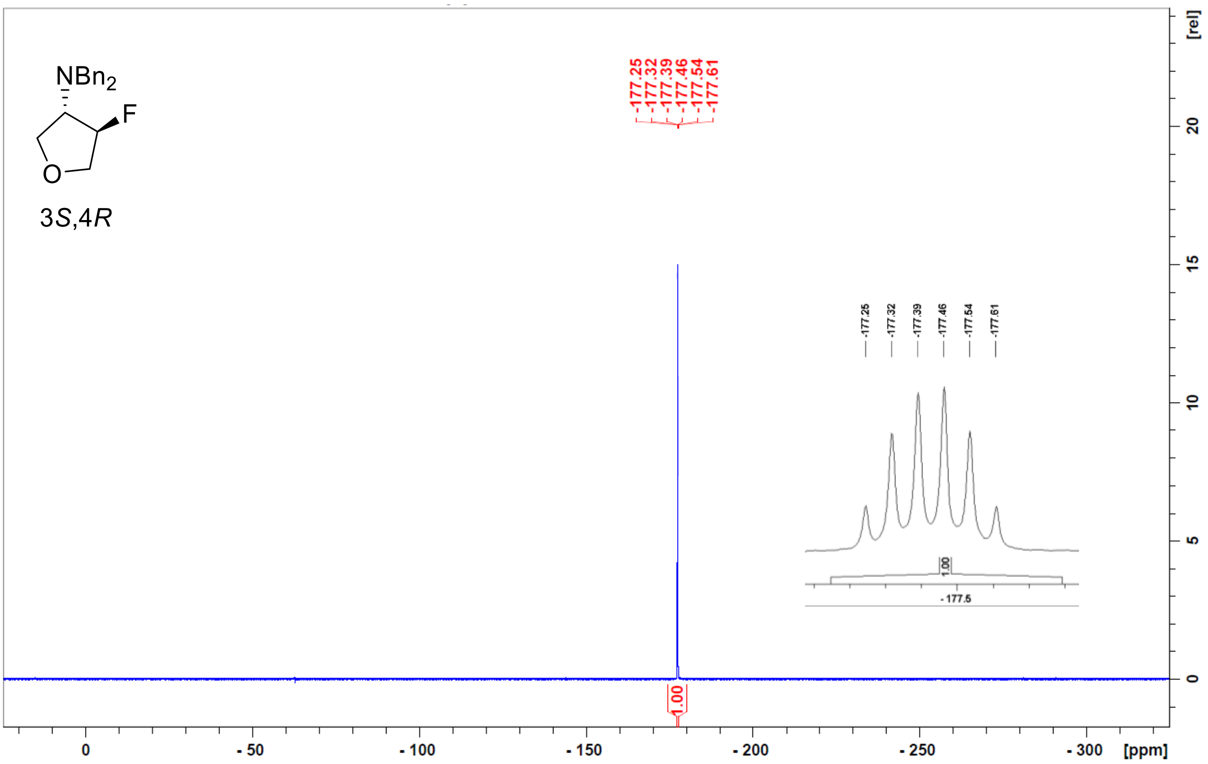

^{1}H NMR (600 MHz, $CDCl_3$): (3*S*,4*R*)-*N*,*N*-dibenzyl-4-fluorotetrahydrofuran-3-amine trichloroacetic acid salt (3*S*,4*R*-**3c**·TCA)

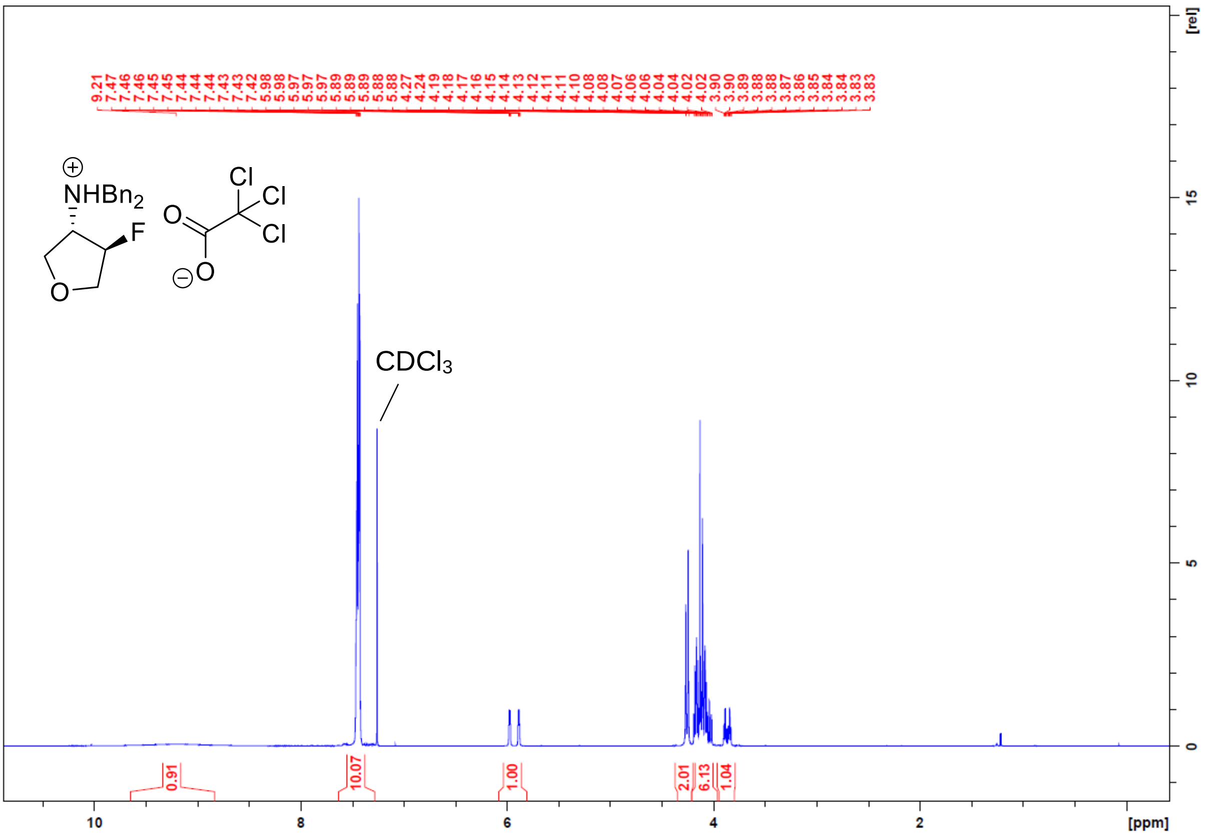

^{19}F NMR (565 MHz, $CDCl_3$): (3*S*,4*R*)-*N*,*N*-dibenzyl-4-fluorotetrahydrofuran-3-amine trichloroacetic acid salt (3*S*,4*R*-**3c**·TCA)

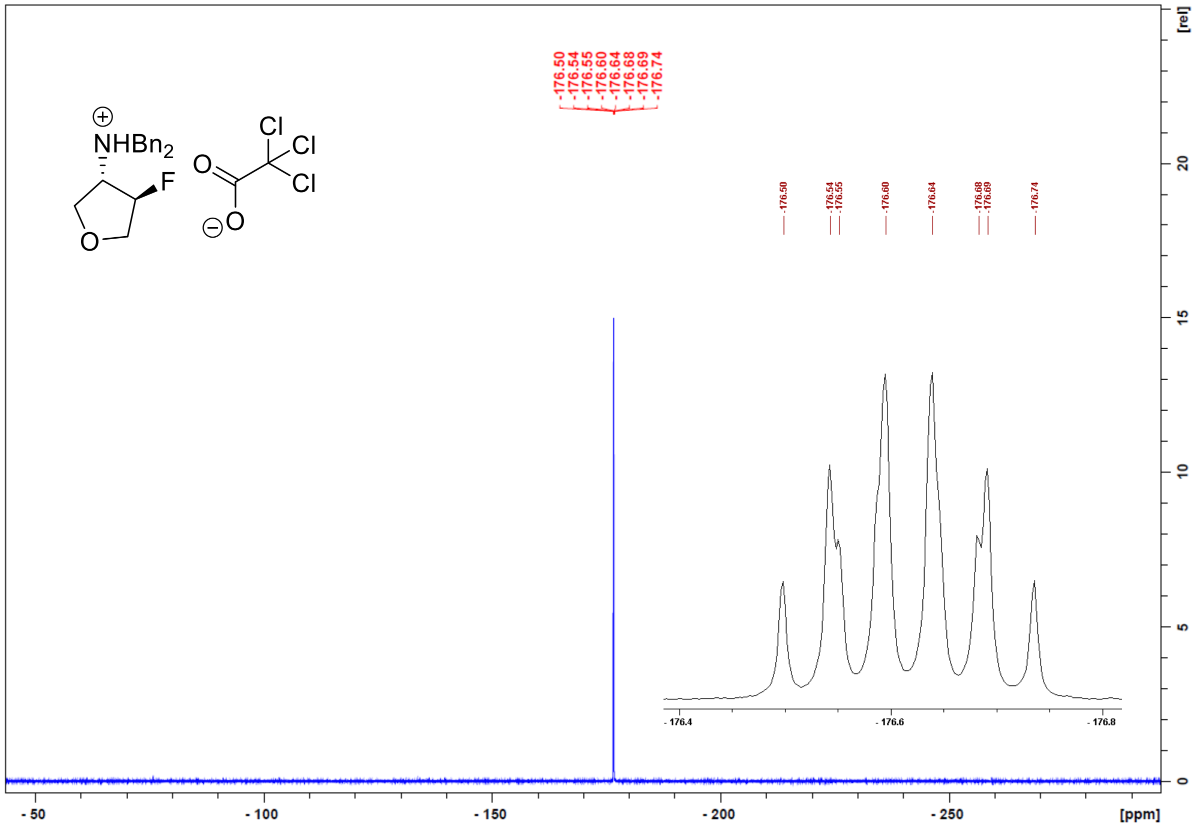

^{13}C NMR (151 MHz, $CDCl_3$): (3*S*,4*R*)-*N*,*N*-dibenzyl-4-fluorotetrahydrofuran-3-amine trichloroacetic acid salt (3*S*,4*R*-**3c**·TCA)

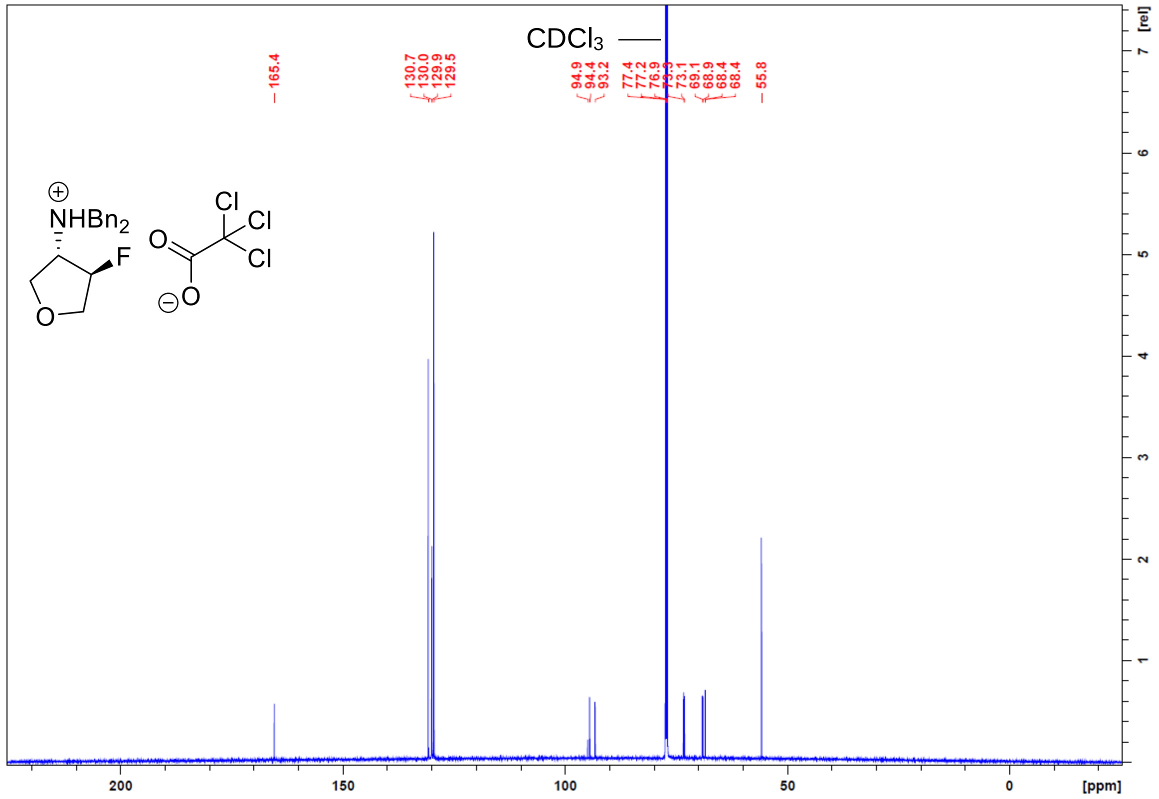

^{1}H NMR (400 MHz, $CDCl_3$): *tert*-Butyl (3*S*,4*S*)-3-(dibenzylamino)-4-fluoropyrrolidine-1-carboxylate (*S*,*S*-**3d**)

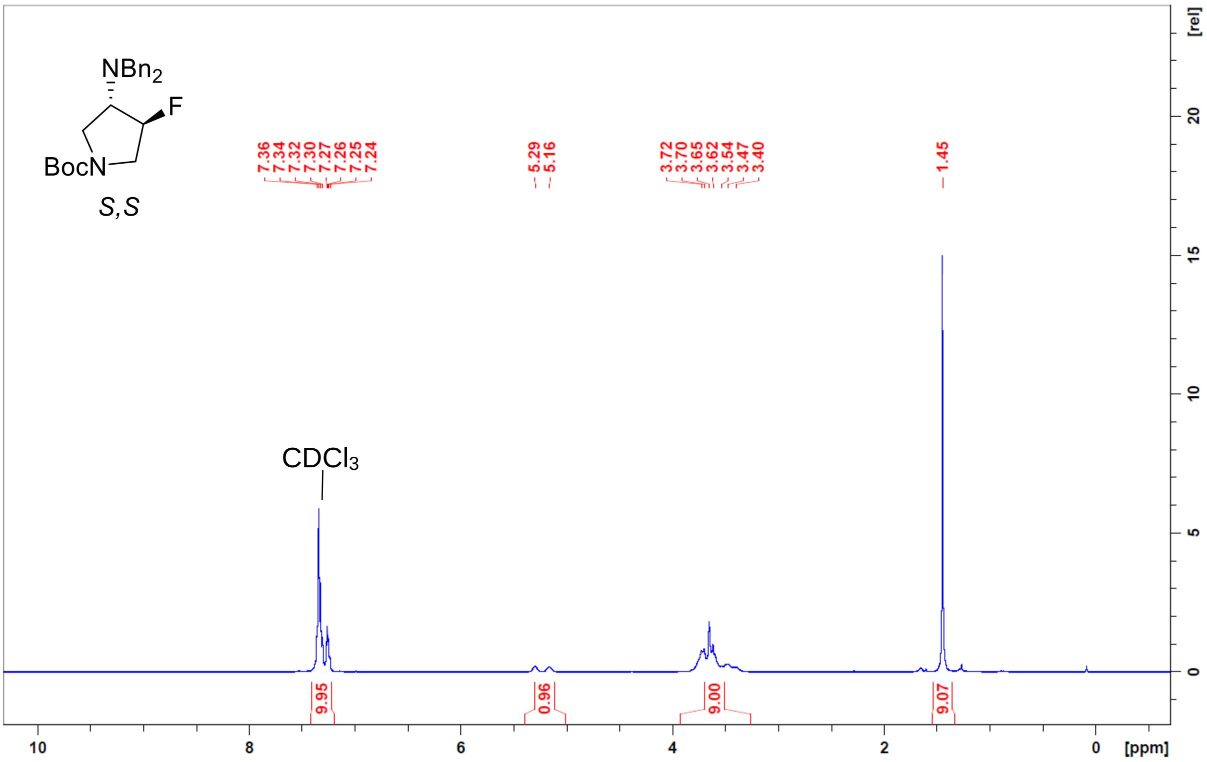

^{19}F NMR (377 MHz, $CDCl_3$): *tert*-Butyl (3*S*,4*S*)-3-(dibenzylamino)-4-fluoropyrrolidine-1-carboxylate (*S*,*S*-**3d**)

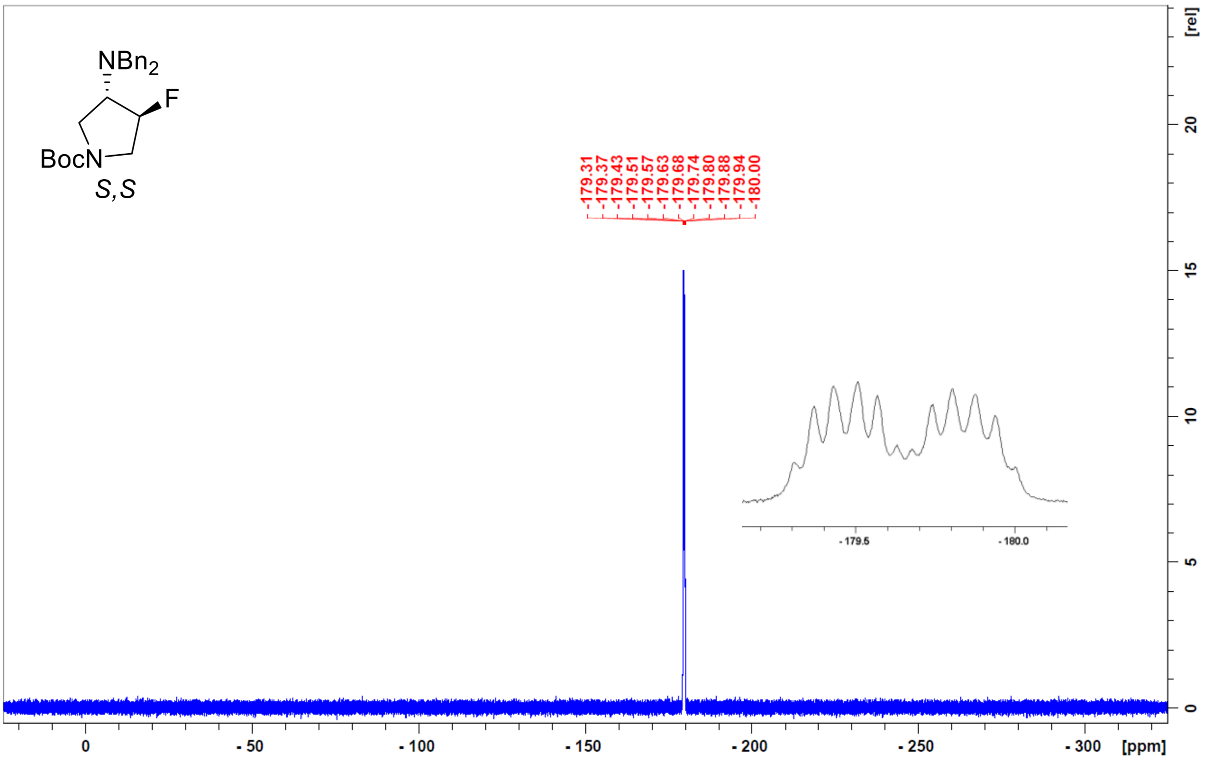

^{13}C NMR (101 MHz, $CDCl_3$): *tert*-Butyl (3*S*,4*S*)-3-(dibenzylamino)-4-fluoropyrrolidine-1-carboxylate (*S*,*S*-**3d**)

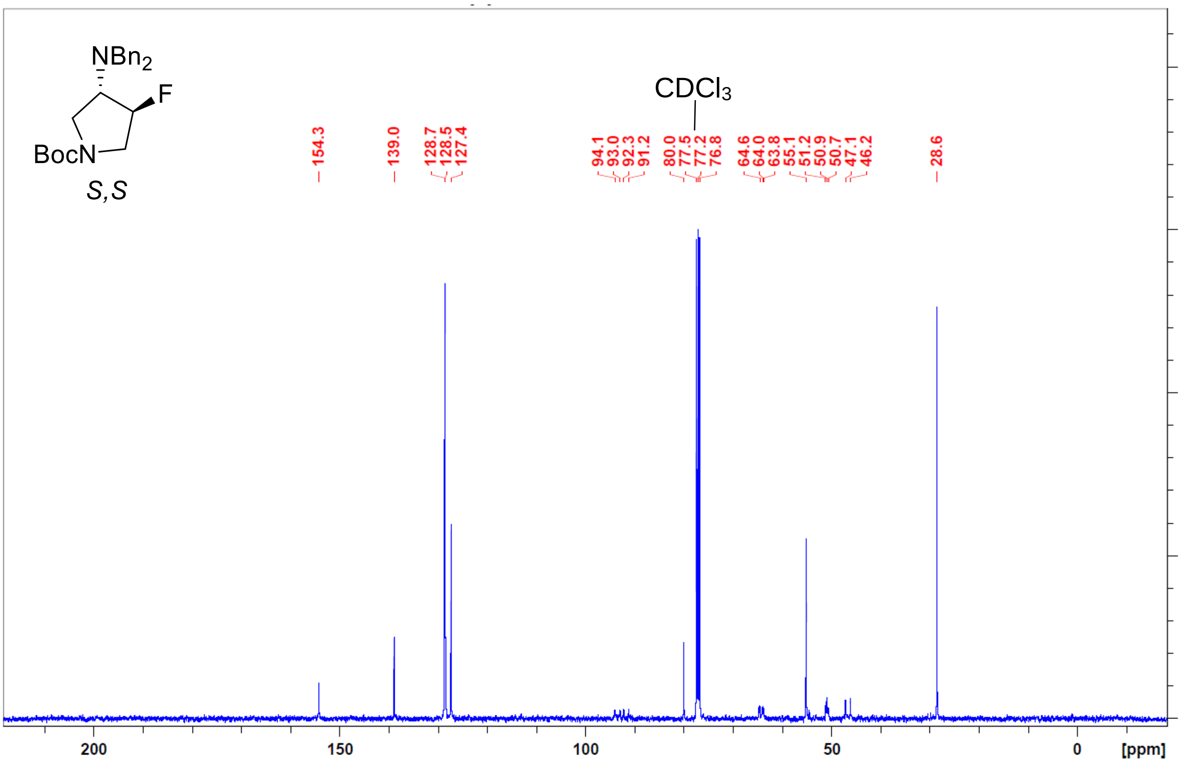

^{1}H NMR (400 MHz, $CDCl_3$): (1*S*,2*S*)-*N*,*N*-dibenzyl-2-fluorocycloheptan-1-amine (*S*,*S*-**3e**)

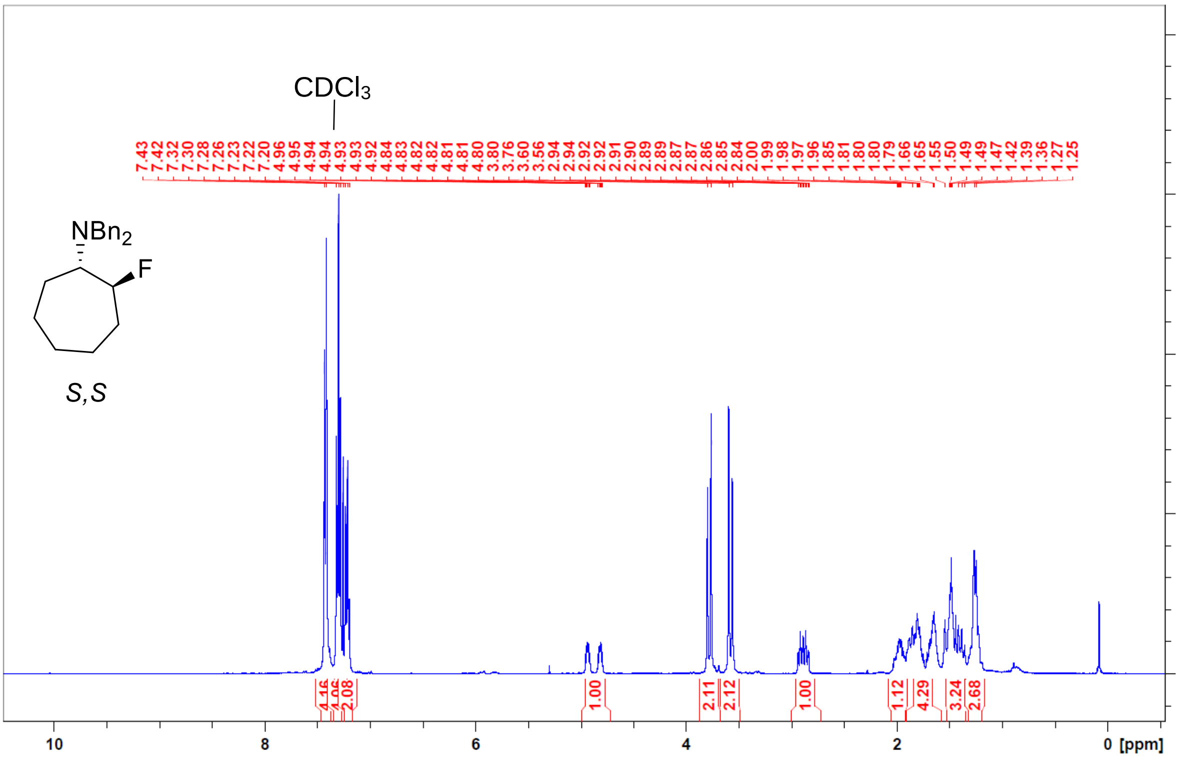

^{13}C NMR (101 MHz, $CDCl_3$): (1*S*,2*S*)-*N*,*N*-dibenzyl-2-fluorocycloheptan-1-amine (*S*,*S*-**3e**)

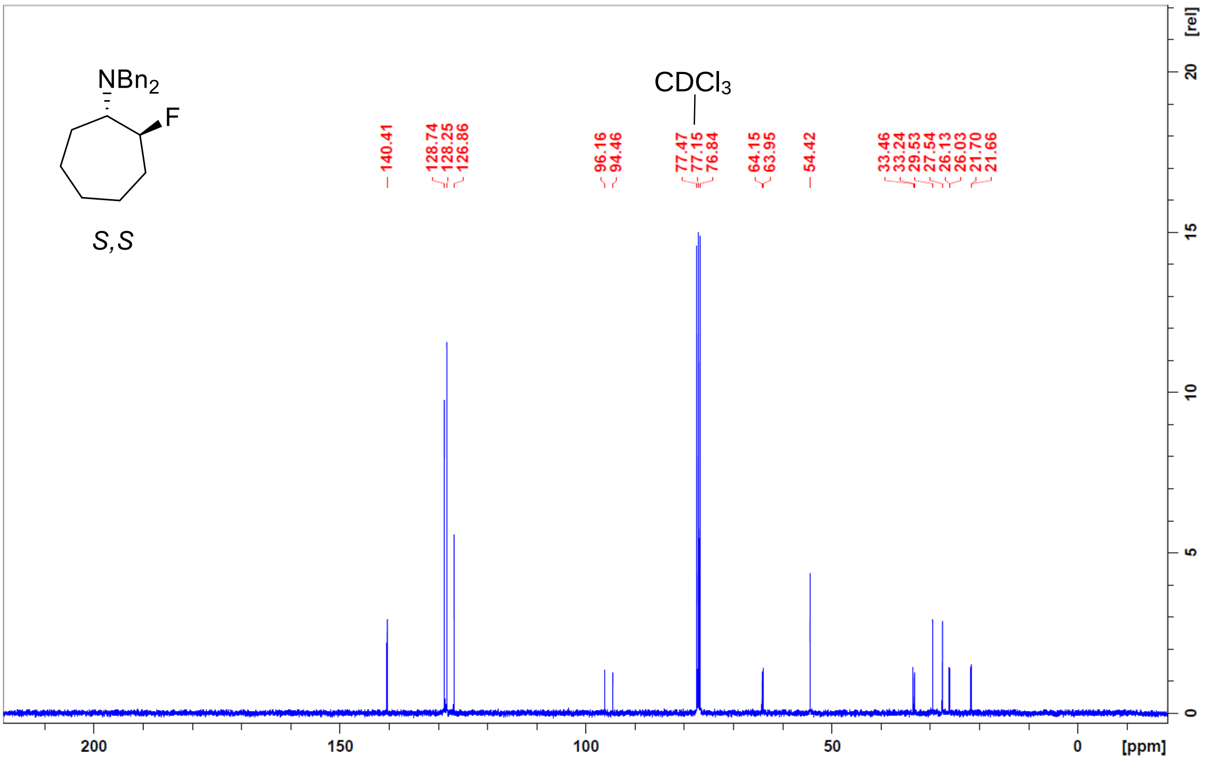

^{19}F NMR (377 MHz, $CDCl_3$): (1*S*,2*S*)-*N*,*N*-dibenzyl-2-fluorocycloheptan-1-amine (*S*,*S*-**3e**)

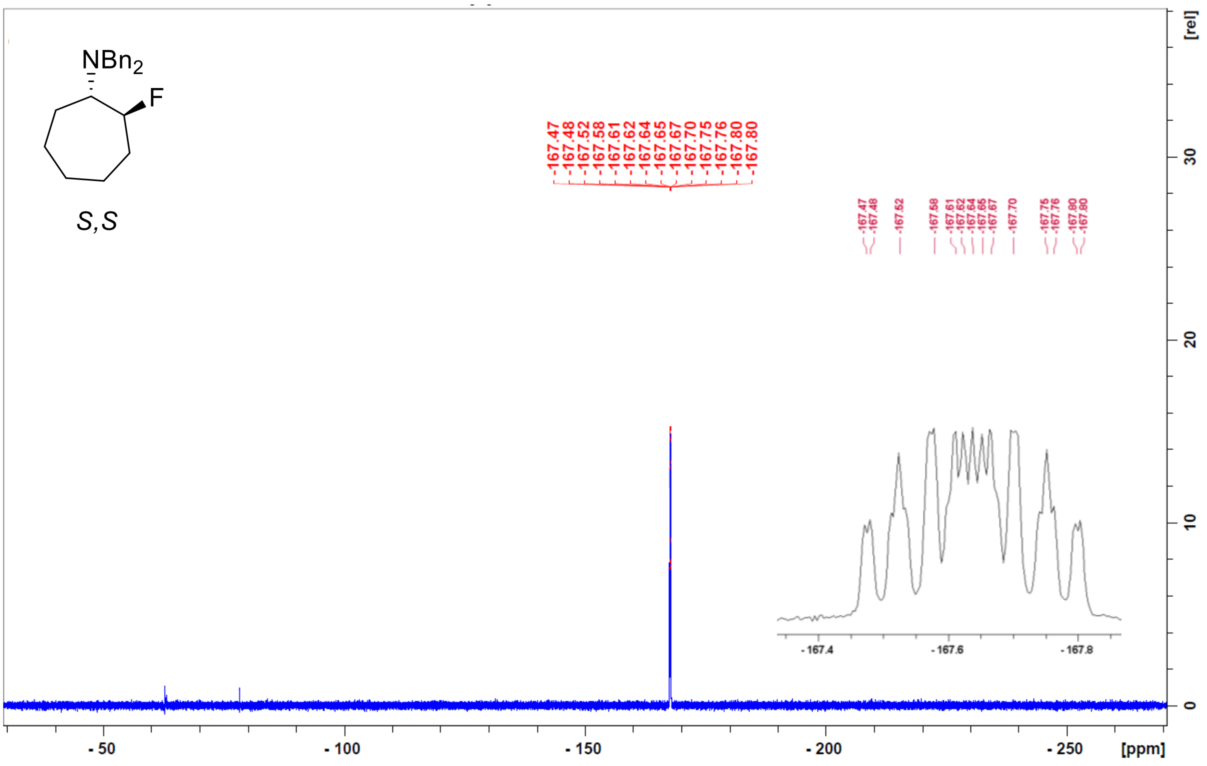

10. HPLC traces

(*S*,*S*)-*N*,*N*-Dibenzyl-2-fluorocyclohexan-1-amine (*S*,*S*-**3a**)

Method: DAICEL CHIRALPAK® IB-3, Heptane:*i*PrOH = 99.5:0.5

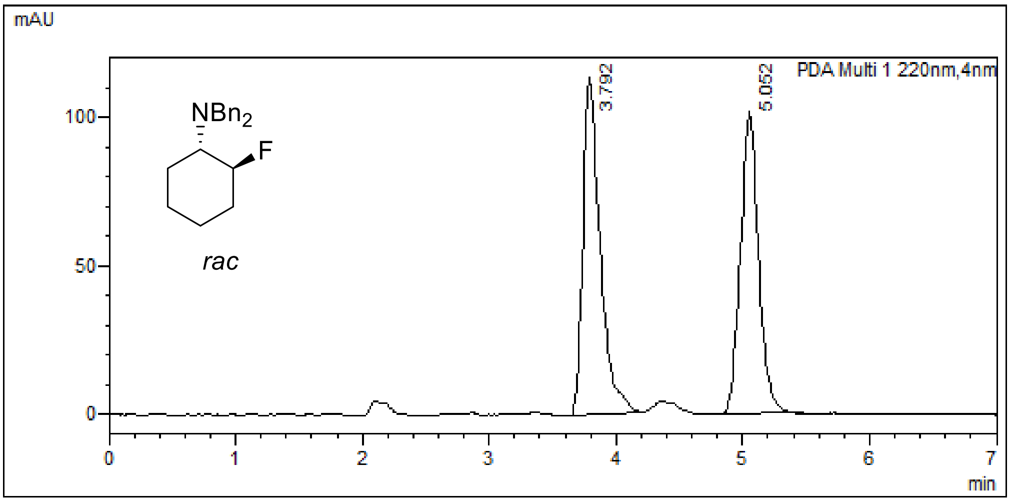

PDA Ch1 220nm

Peak#	Ret. Time	Area	Area%
1	3.792	1057126	51.021
2	5.052	1014807	48.979

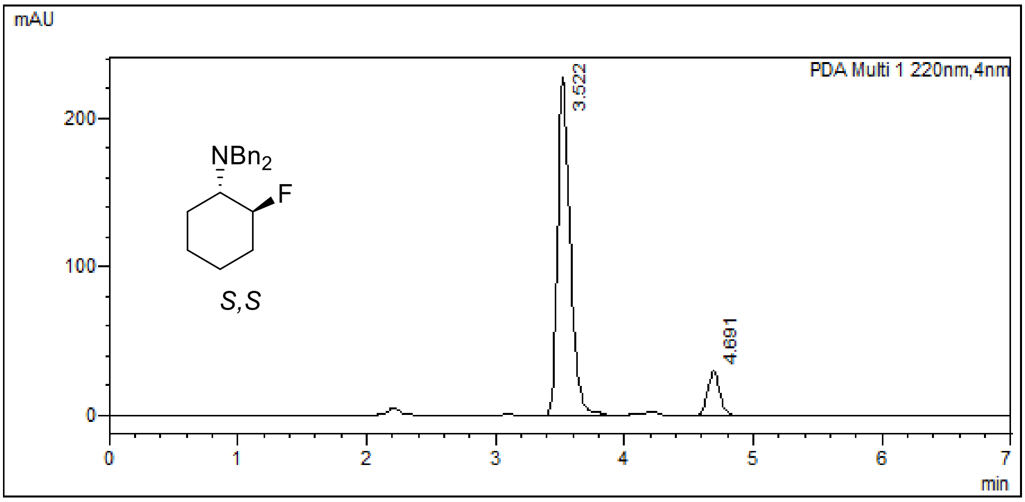

PDA Ch1 220nm

Peak#	Ret. Time	Area	Area%
1	3.522	1542493	88.750
2	4.691	195524	11.250

(*S*,*S*)-*N*,*N*-Dibenzyl-2-fluorocyclopentan-1-amine (*S*,*S*-**3b**)

Method: DAICEL CHIRALPAK® OJ-H, Heptane:EtOH = 98:2

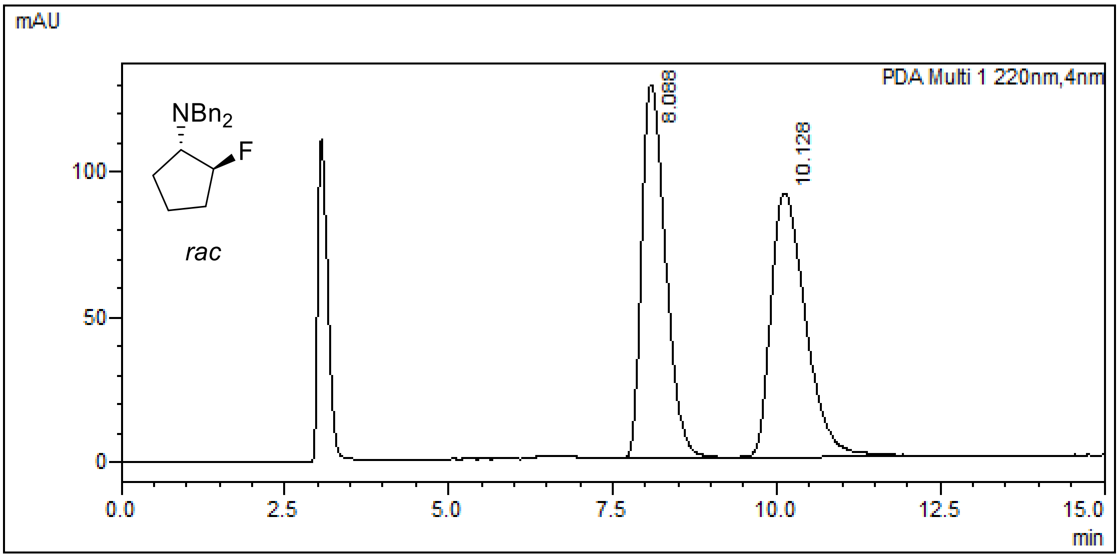

PDA Ch1 220nm

Peak#	Ret. Time	Area%
1	8.088	49.638
2	10.128	50.362
Total		100.000

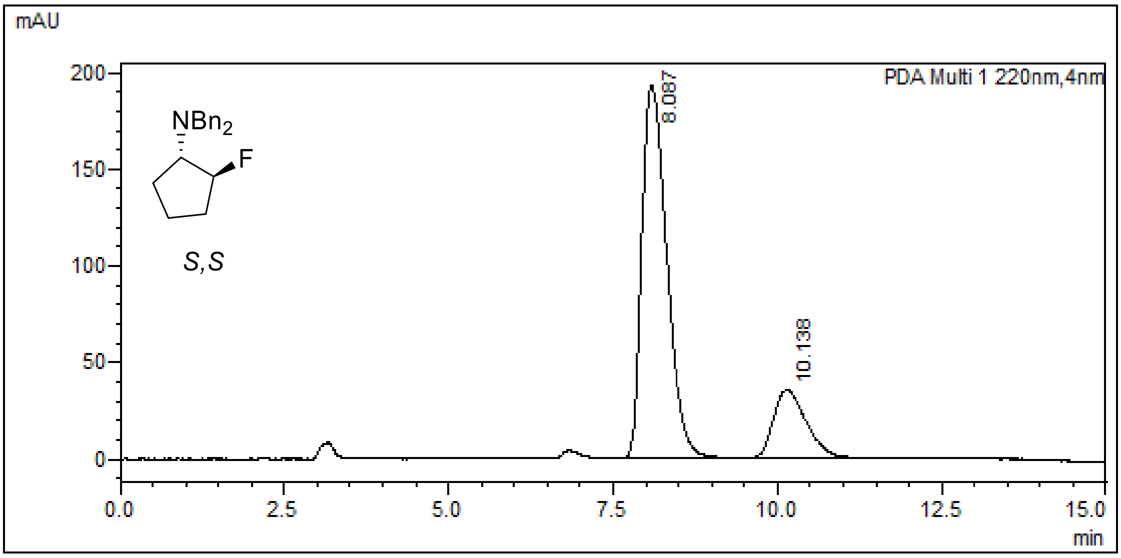

PDA Ch1 220nm

Peak#	Ret. Time	Area%
1	8.087	80.611
2	10.138	19.389
Total		100.000

(3*S*,4*R*)-*N*,*N*-Dibenzyl-4-fluorotetrahydrofuran-3-amine (3*S*,4*R*-**3c**)

Method: DAICEL CHIRALPAK® IB-3, Heptane:*i*PrOH = 99.5:0.5

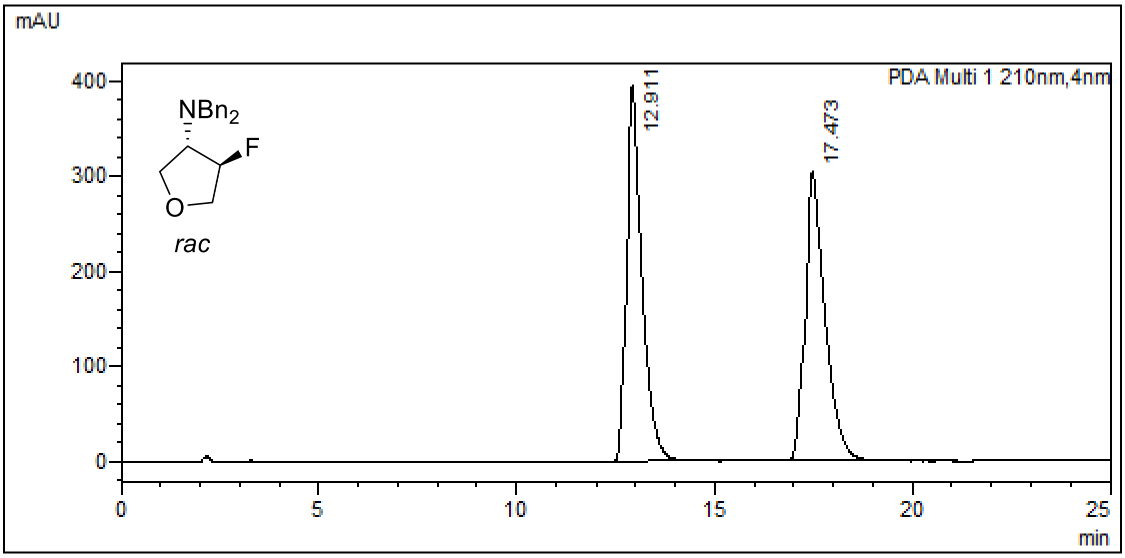

PDA Ch1 210nm

Peak#	Ret. Time	Area%
1	12.911	50.049
2	17.473	49.951
Total		100.000

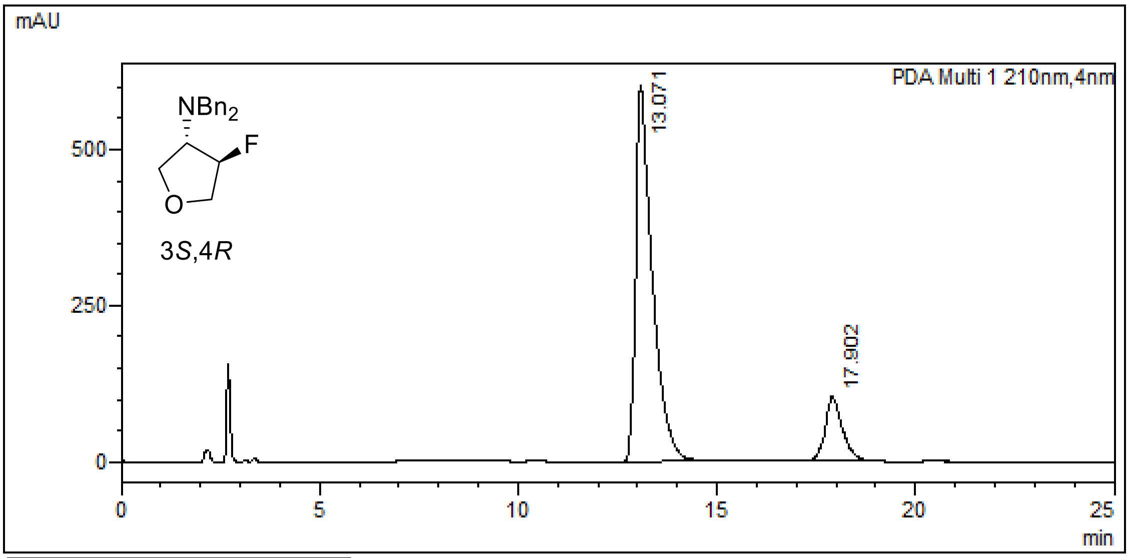

PDA Ch1 210nm

Peak#	Ret. Time	Area%
1	13.071	85.074
2	17.902	14.926
Total		100.000

Crystallization of (3*S*,4*R*)-*N*,*N*-Dibenzyl-4-fluorotetrahydrofuran-3-amine (3*S*,4*R*-**3c**)

Mother liquor:

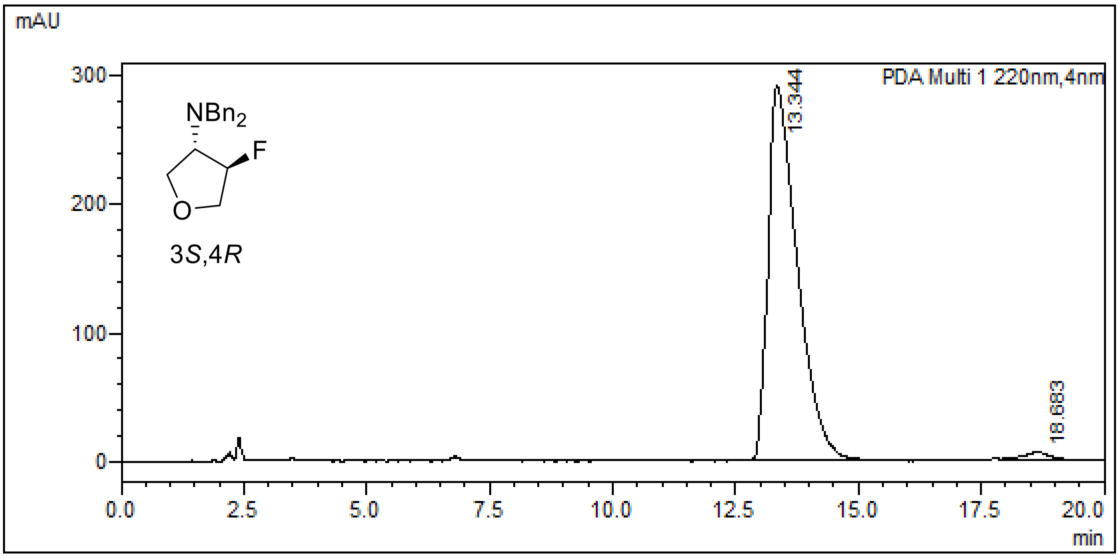

PDA Ch1 220nm

Peak#	Ret. Time	Area%
1	13.344	98.407
2	18.683	1.593

Crystals:

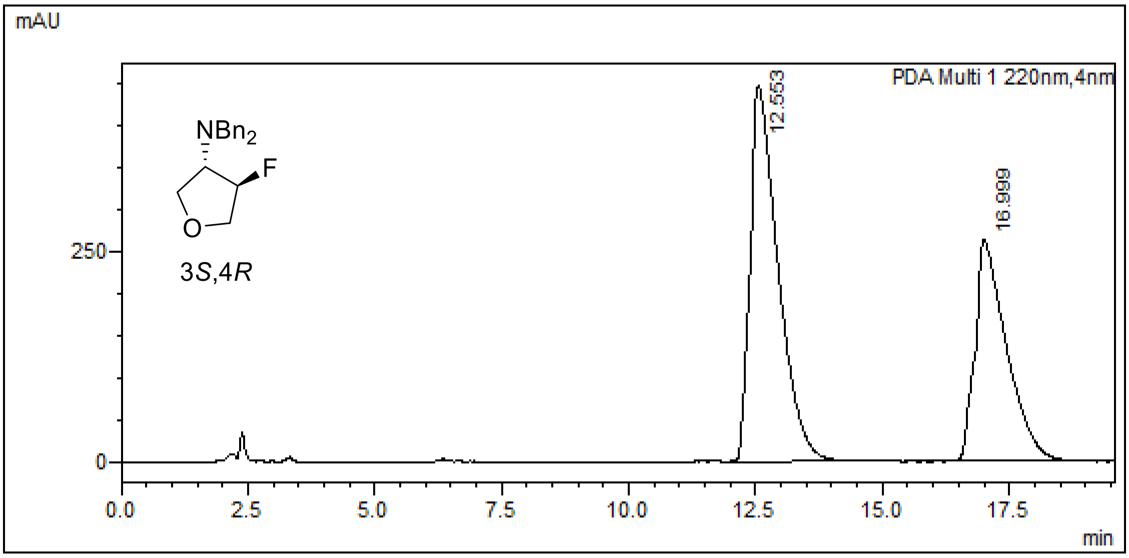

PDA Ch1 220nm

Peak#	Ret. Time	Area%
1	12.553	60.290
2	16.999	39.710

tert-Butyl (3*S*,4*S*)-3-(dibenzylamino)-4-fluoropyrrolidine-1-carboxylate (*S*,*S*-**3d**)

Method: DAICEL CHIRALPAK® IB-3, Heptane:*i*PrOH = 99.0:1.0

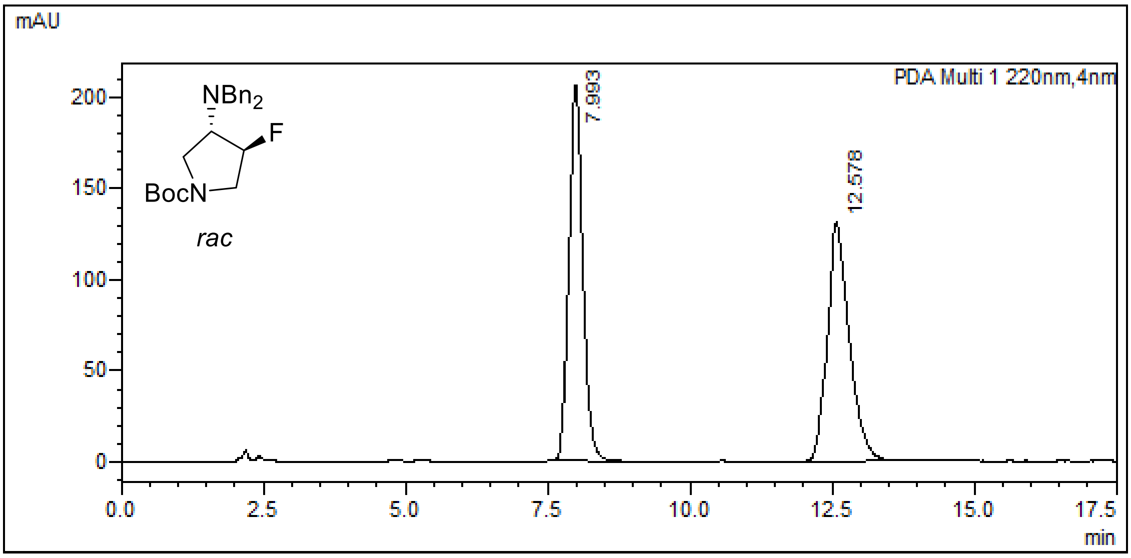

PDA Ch1 220nm

Peak#	Ret. Time	Area%
1	7.993	49.709
2	12.578	50.291
Total		100.000

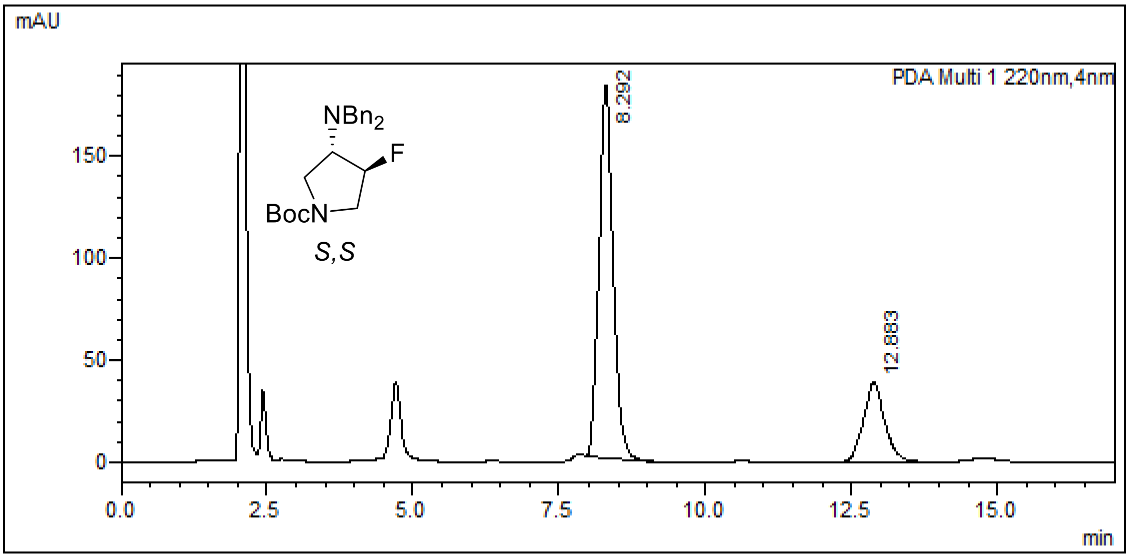

PDA Ch1 220nm

Peak#	Ret. Time	Area%
1	8.292	75.597
2	12.883	24.403
Total		100.000

(1*S*,2*S*)-*N*,*N*-Dibenzyl-2-fluorocycloheptan-1-amine (*S*,*S*-**3e**)

Method: DAICEL CHIRALPAK® IC-3, Heptane:*i*PrOH = 99.5:0.5, 1 mL/min

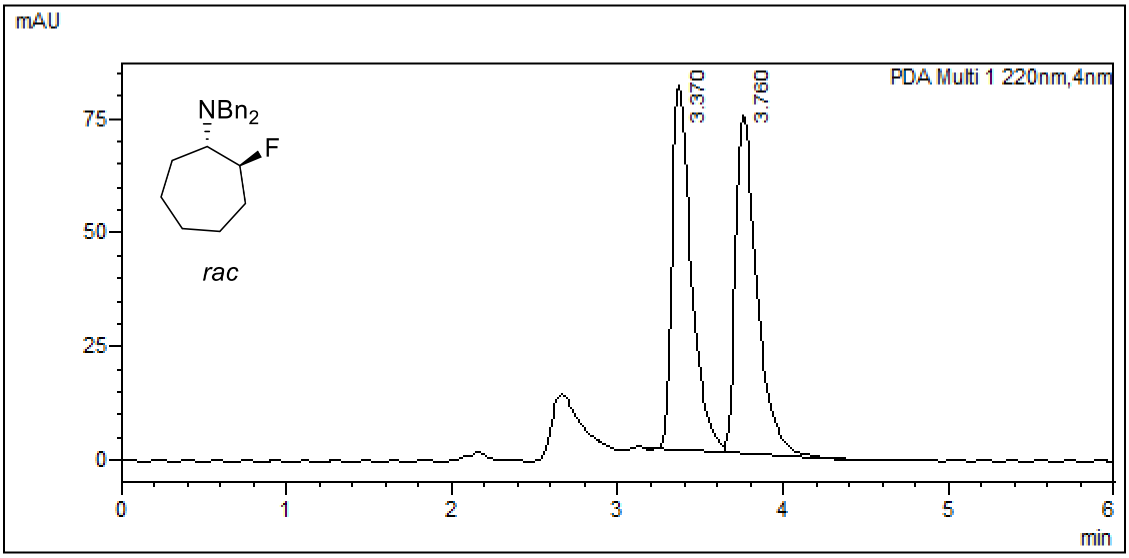

PDA Ch1 220nm

Peak#	Ret. Time	Area%
1	3.370	49.325
2	3.760	50.675
Total		100.000

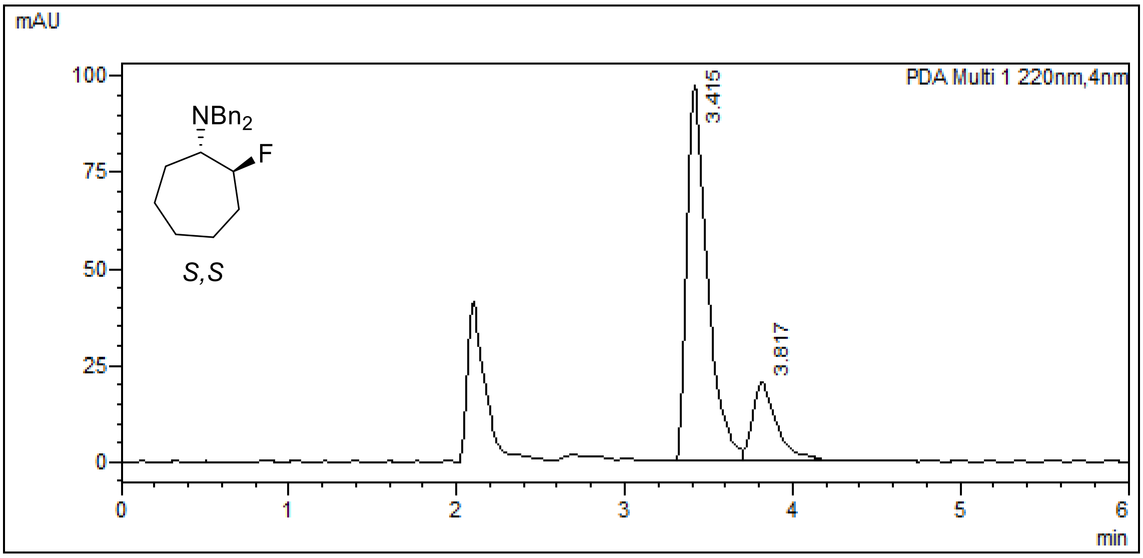

PDA Ch1 220nm

Peak#	Ret. Time	Area%
1	3.415	80.856
2	3.817	19.144
Total		100.000

2-((1*R*,2*R*)-2-fluoro-1,2-diphenylethyl)-1,2,3,4-tetrahydroisoquinoline (*R*,*R*-**3f**)

Method: DAICEL CHIRALPAK® IF-3, Heptane:*i*PrOH = 99:1

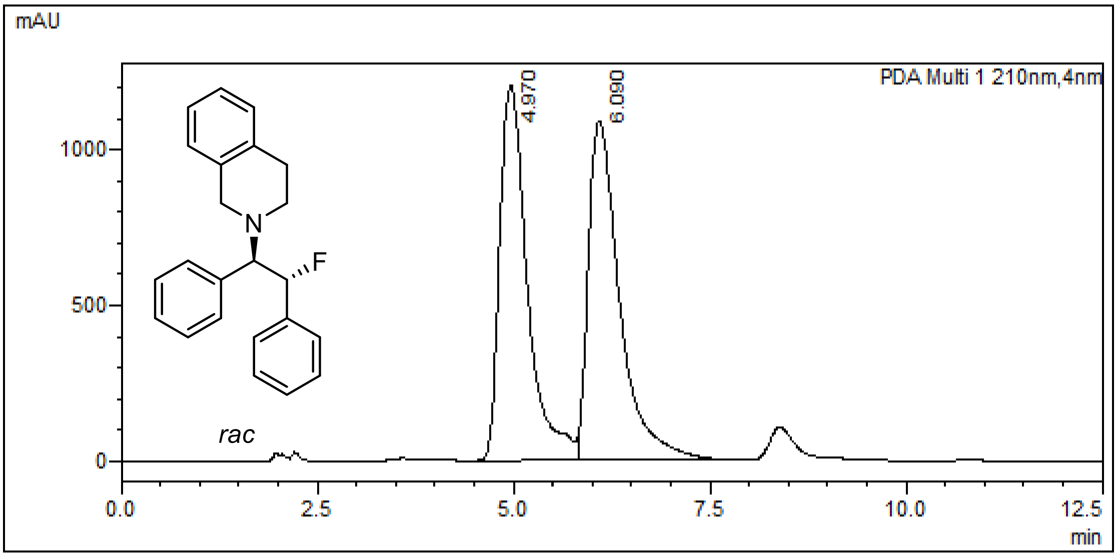

PDA Ch1 210nm

Peak#	Ret. Time	Area%
1	4.970	49.587
2	6.090	50.413

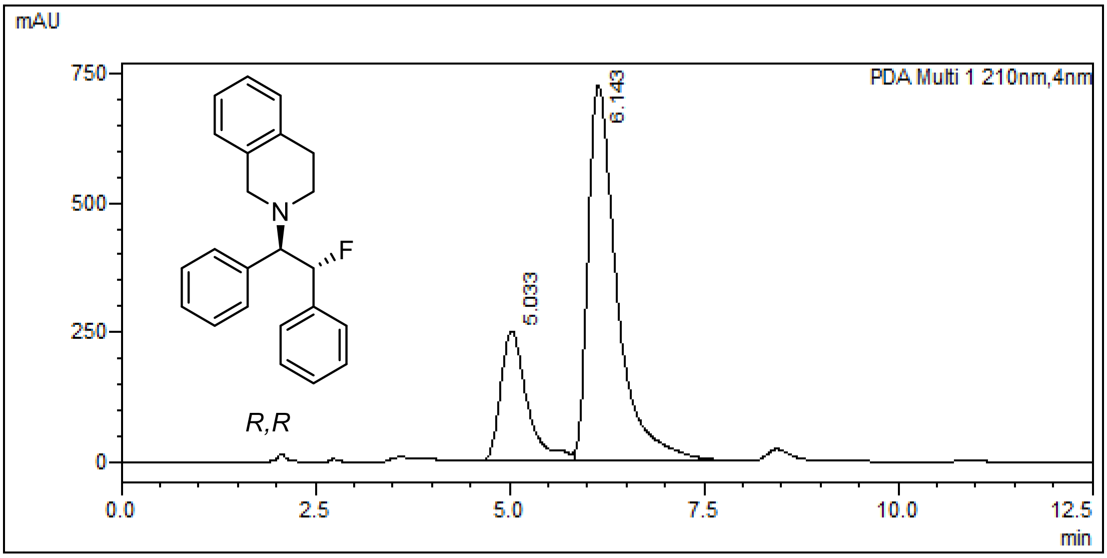

PDA Ch1 210nm

Peak#	Ret. Time	Area%
1	5.033	24.136
2	6.143	75.864

rac-Benzyl(2-bromocyclohexyl)sulfane (*R*,*R*-**3g**)

Method: DAICEL CHIRALPAK® IC-3, Heptane:EtOH = 99:1, 1 mL/min

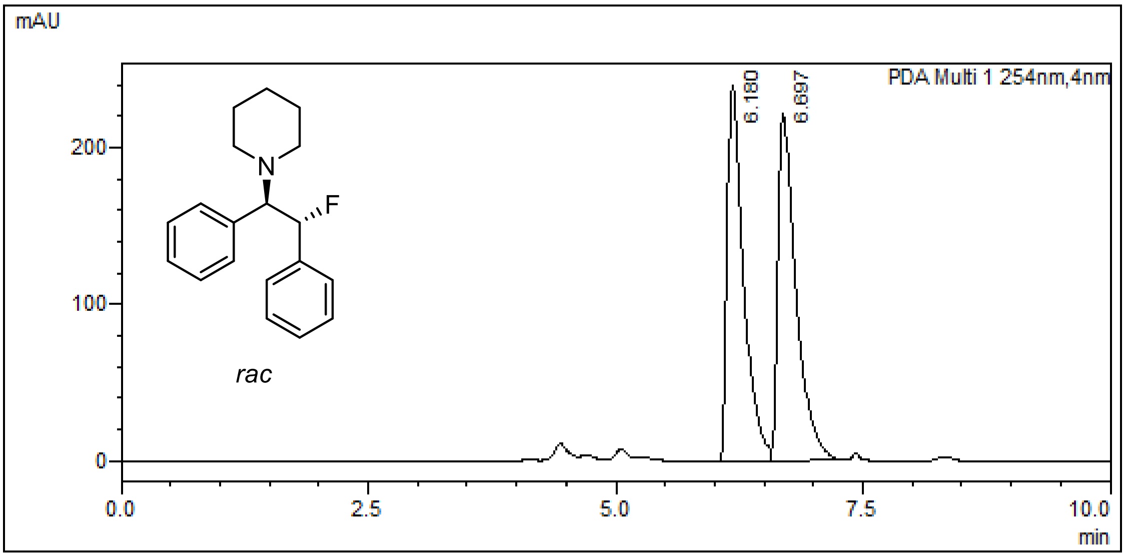

PDA Ch1 254nm

Peak#	Ret. Time	Area%
1	6.180	49.545
2	6.697	50.455

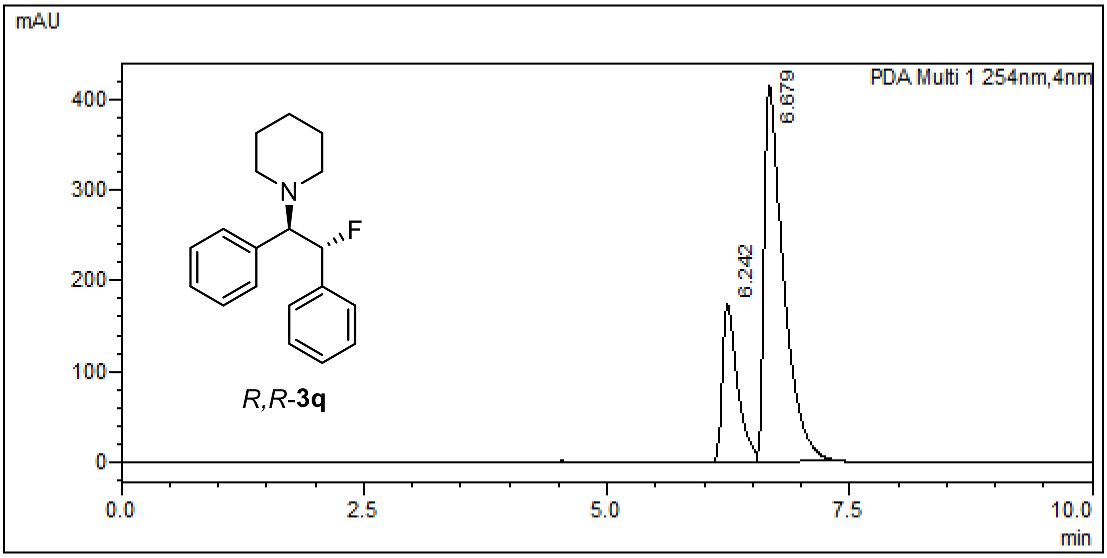

PDA Ch1 254nm

Peak#	Ret. Time	Area%
1	6.242	24.849
2	6.679	75.151

(1*R*,2*R*)-2-fluoro-N,N-dimethyl-1,2-diphenylethan-1-amine (*R*,*R*-**3h**)

Method: DAICEL CHIRALPAK® IC-3, Heptane:*i*PrOH = 99:1, 1 mL/min.

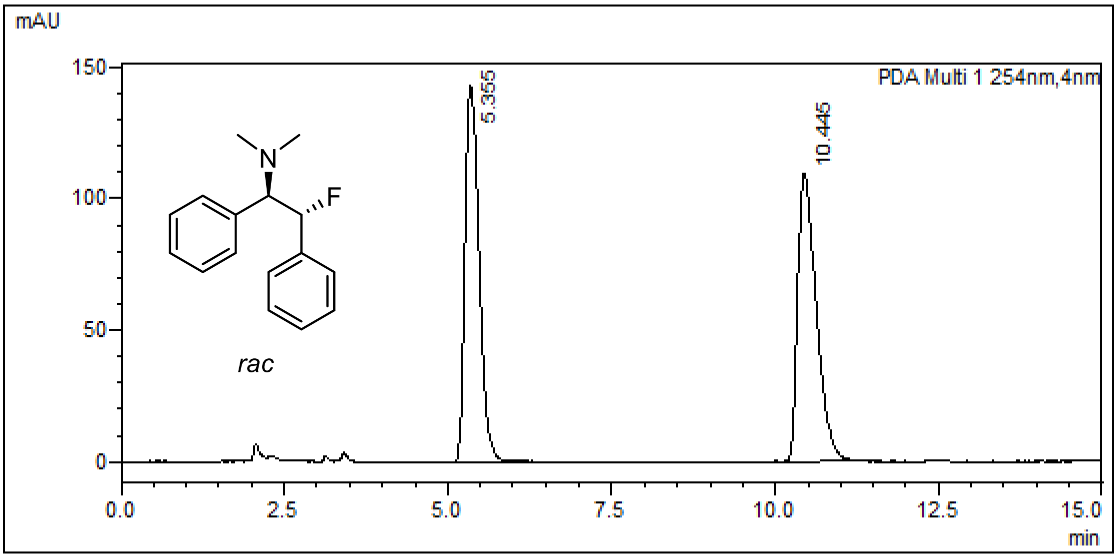

PDA Ch1 254nm

Peak#	Ret. Time	Area%
1	5.355	49.858
2	10.445	50.142

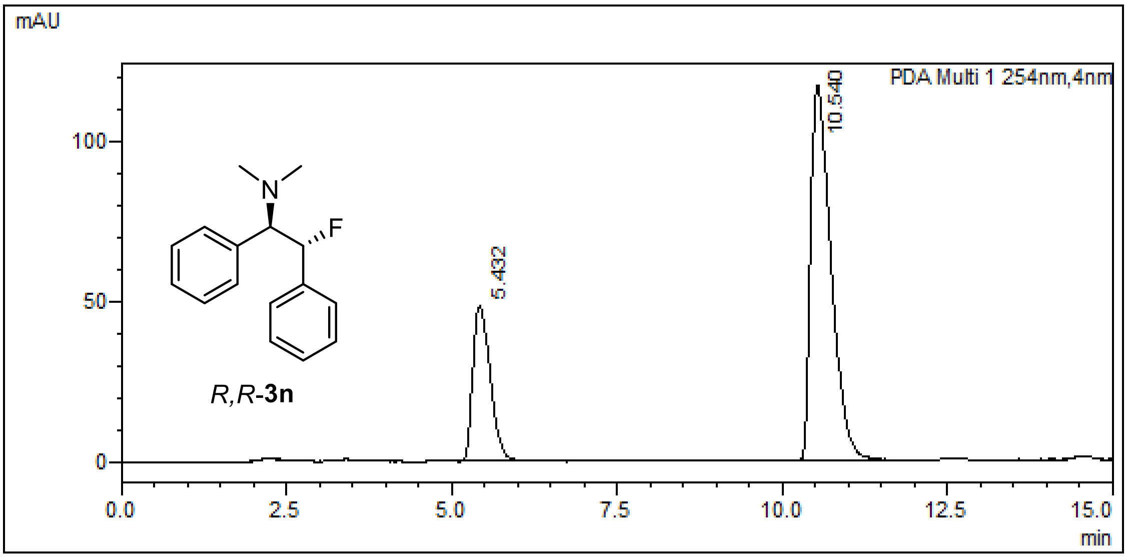

PDA Ch1 254nm

Peak#	Ret. Time	Area%
1	5.432	25.913
2	10.540	74.087

11. References

(1) Pupo, G.; Vicini, A. C.; Ascough, D. M. H.; Ibba, F.; Christensen, K. E.; Thompson, A. L.; Brown, J. M.; Paton, R. S.; Gouverneur, V. Hydrogen Bonding Phase-Transfer Catalysis with Potassium Fluoride: Enantioselective Synthesis of β-Fluoroamines. *Journal of the American Chemical Society* **2019**, *141* (7), 2878–2883. DOI: 10.1021/jacs.8b12568.

(2) Metrano, A. J.; Abascal, N. C.; Mercado, B. Q.; Paulson, E. K.; Hurtley, A. E.; Miller, S. J. Diversity of Secondary Structure in Catalytic Peptides with β-Turn-Biased Sequences. *Journal of the American Chemical Society* **2017**, *139* (1), 492–516. DOI: 10.1021/jacs.6b11348.

(3) Leggio, A.; Belsito, E. L.; De Luca, G.; Di Gioia, M. L.; Leotta, V.; Romio, E.; Siciliano, C.; Liguori, A. One-pot synthesis of amides from carboxylic acids activated using thionyl chloride. *RSC Advances* **2016**, *6* (41), 34468–34475. DOI: 10.1039/C5RA24527C.

(4) Vicini, A. C.; Pupo, G.; Ibba, F.; Gouverneur, V. Multigram synthesis of *N*-alkyl *bis*-ureas for asymmetric hydrogen bonding phase-transfer catalysis. *Nature Protocols* **2021**, *16* (12), 5559–5591. DOI: 10.1038/s41596-021-00625-y.

(5) Flavell, R. R.; von Morze, C.; Blecha, J. E.; Korenchan, D. E.; Van Criekinge, M.; Sriram, R.; Gordon, J. W.; Chen, H.-Y.; Subramaniam, S.; Bok, R. A.; Wang, Z. J.; Vigneron, D. B.; Larson, P. E.; Kurhanewicz, J.; Wilson, D. M. Application of Good's buffers to pH imaging using hyperpolarized ^{13}C MRI. *Chemical Communications* **2015**, *51* (74), 14119–14122. DOI: 10.1039/C5CC05348J.

(6) Degerbeck, F.; Fransson, B.; Grehn, L.; Ragnarsson, U. Synthesis of ^{15}N-labelled chiral Boc-amino acids from triflates: enantiomers of leucine and phenylalanine. *Journal of the Chemical Society, Perkin Transactions 1* **1993**, (1), 11–14. DOI: 10.1039/P19930000011.

(7) (a) Askari, M. S.; Orio, M.; Ottenwaelder, X. Controlled nitrene transfer from a tyrosinase-like arylnitroso–copper complex. *Chemical Communications* **2015**, *51* (56), 11206–11209. DOI: 10.1039/C5CC02806J ; (b) Sharma, V.; De, D.; Saha, R.; Das, R.; Chattaraj, P. K.; Bharadwaj, P. K. A Cu(II)-MOF capable of fixing CO_2 from air and showing high capacity H_2 and CO_2 adsorption. *Chemical Communications* **2017**, *53* (100), 13371–13374. DOI: 10.1039/C7CC08315G ; (c) Rozsar, D.; Farley, A. J. M.; McLauchlan, I.; Shennan, B. D. A.; Yamazaki, K.; Dixon, D. J. Bifunctional Iminophosphorane-Catalyzed Enantioselective Nitroalkane Addition to Unactivated α,β-Unsaturated Esters. *Angewandte Chemie International Edition* **2023**, *62* (21), e202303391. DOI: 10.1002/anie.202303391.

(8) Rasheed, S.; Rao, D. N.; Reddy, A. S.; Shankar, R.; Das, P. Sulphuric acid immobilized on silica gel (H_2SO_4–SiO_2) as an eco-friendly catalyst for transamidation. *RSC Advances* **2015**, *5* (14), 10567–10574. DOI: 10.1039/C4RA16571C.

(9) Bagheri Natanzi, M.; Kazemi, F.; Zand, Z.; Kaboudin, B. Highly chemoselective and fast practical visible photoreduction of nitroaromatic compounds to aromatic amines and amides using a self-assembled triad TiO_2-TEOA-NC (LMCT/EDA) complex system. *Green Chemistry* **2024**, *26* (3), 1637–1652. DOI: 10.1039/D3GC02563B.

(10) Gavinolla, V.; Thangalipalli, S.; Bandalla, S. G.; Panduga, R.; Neella, C. K. A thermo-regulated highly regioselective mono and dihalogenations of phenols and anilines in water employing new Lewis base adducts (LBAs) $[DBUBr]^+Br^-$ and $[DBUI]^+I^-$ as green reagents: a simple approach. *New Journal of Chemistry* **2023**, *47* (45), 20777–20784. DOI: 10.1039/D3NJ03370H.

(11) Li, X.; Deng, X.; Coyne, A. G.; Srinivasan, R. *meta*-Nitration of Arenes Bearing *ortho*/*para* Directing Group(s) Using C−H Borylation. *Chemistry – A European Journal* **2019**, *25* (34), 8018–8023. DOI: 10.1002/chem.201901633.

(12) Martinez, G. E.; Nugent, J. W.; Fout, A. R. Simple Nickel Salts for the Amination of (Hetero)aryl Bromides and Iodides with Lithium Bis(trimethylsilyl)amide. *Organometallics* **2018**, *37* (18), 2941–2944. DOI: 10.1021/acs.organomet.8b00567.

(13) Monaco, M. R.; Prévost, S.; List, B. Organocatalytic Asymmetric Hydrolysis of Epoxides. *Angewandte Chemie International Edition* **2014**, *53* (31), 8142–8145. DOI: 10.1002/anie.201400170.

(14) Hansen, S. U.; Bols, M. 1-Azaribofuranoside analogues as designed inhibitors of purine nucleoside phosphorylase. Synthesis and biological evaluation. *Acta Chemica Scandinavica* **1998**, *52* (10), 1214–1222. DOI: 10.3891/acta.chem.scand.52-1214.

(15) Yamasaki, M.; Lu, P.-P.; Morgan, B. P.; Garard, M.; Collibee, S.; Koganemaru, Y.; Sami, Y.; Negoro, K.; Imada, S.; Kamikubo, T.; Ishikawa, N.; Inagi, Y.; Ohnuki, K.; Sato, S.; Tanaka, H.; Ishida, J.; Abe, T. Slow Skeletal Troponin Activators. WO2023/245137, 2023.

(16) Monaco, M. R.; Poladura, B.; Diaz de Los Bernardos, M.; Leutzsch, M.; Goddard, R.; List, B. Activation of Carboxylic Acids in Asymmetric Organocatalysis. *Angewandte Chemie International Edition* **2014**, *53* (27), 7063–7067. DOI: 10.1002/anie.201400169.

(17) Ibba, F.; Pupo, G.; Thompson, A. L.; Brown, J. M.; Claridge, T. D. W.; Gouverneur, V. Impact of Multiple Hydrogen Bonds with Fluoride on Catalysis: Insight from NMR Spectroscopy. *Journal of the American Chemical Society* **2020**, *142* (46), 19731–19744. DOI: 10.1021/jacs.0c09832.

(18) Kuzmič, P. Program DYNAFIT for the Analysis of Enzyme Kinetic Data: Application to HIV Proteinase. *Analytical Biochemistry* **1996**, *237* (2), 260–273. DOI: 10.1006/abio.1996.0238.

(19) (a) *Gaussian 16 Rev. C.01*; Gaussian Inc.: Wallingford, CT, 2016 ; (b) Zhao, Y.; Truhlar, D. G. A new local density functional for main-group thermochemistry, transition metal bonding, thermochemical kinetics, and noncovalent interactions. *The Journal of Chemical Physics* **2006**, *125* (19). DOI: 10.1063/1.2370993 ; (c) Weigend, F.; Ahlrichs, R. Balanced basis sets of split valence, triple zeta valence and quadruple zeta valence quality for H to Rn: Design and assessment of accuracy. *Physical Chemistry Chemical Physics* **2005**, *7* (18), 3297–3305. DOI: 10.1039/B508541A ; (d) Grimme, S.; Antony, J.; Ehrlich, S.; Krieg, H. A consistent and accurate *ab initio* parametrization of density functional dispersion correction (DFT-D) for the 94 elements H-Pu. *The Journal of Chemical Physics* **2010**, *132* (15). DOI: 10.1063/1.3382344.

(20) Marenich, A. V.; Cramer, C. J.; Truhlar, D. G. Universal Solvation Model Based on Solute Electron Density and on a Continuum Model of the Solvent Defined by the Bulk Dielectric Constant and Atomic Surface Tensions. *The Journal of Physical Chemistry B* **2009**, *113* (18), 6378–6396. DOI: 10.1021/jp810292n.

(21) (a) Neese, F. The ORCA program system. *WIREs Computational Molecular Science* **2012**, *2* (1), 73–78. DOI: 10.1002/wcms.81 ; (b) Neese, F. Software update: The ORCA program system—Version 5.0. *WIREs Computational Molecular Science* **2022**, *12* (5), e1606. DOI: 10.1002/wcms.1606 ; (c) Zhao, Y.; Truhlar, D. G. The M06 suite of density functionals for main group thermochemistry, thermochemical kinetics, noncovalent interactions, excited states, and transition elements: two new functionals and systematic testing of four M06-class functionals and 12 other functionals. *Theoretical Chemistry Accounts* **2008**, *120* (1), 215–241. DOI: 10.1007/s00214-007-0310-x.

(22) Luchini, G.; Alegre-Requena, J. V.; Funes-Ardoiz, I.; Paton, R. S. GoodVibes: automated thermochemistry for heterogeneous computational chemistry data *F1000Research* **2020**, *9* (291). DOI: 10.12688/f1000research.22758.1.

(23) Grimme, S. Supramolecular Binding Thermodynamics by Dispersion-Corrected Density Functional Theory. *Chemistry – A European Journal* **2012**, *18* (32), 9955–9964. DOI: 10.1002/chem.201200497.

(24) (a) de Souza, B. GOAT: A Global Optimization Algorithm for Molecules and Atomic Clusters. *Angewandte Chemie International Edition* **2025**, *64* (18), e202500393. DOI: 10.1002/anie.202500393 ; (b) Bannwarth, C.; Ehlert, S.; Grimme, S. GFN2-xTB—An Accurate and Broadly Parametrized Self-Consistent Tight-Binding Quantum Chemical Method with Multipole Electrostatics and Density-Dependent Dispersion Contributions. *Journal of Chemical Theory and Computation* **2019**, *15* (3), 1652–1671. DOI: 10.1021/acs.jctc.8b01176.

(25) Stahn, M.; Ehlert, S.; Grimme, S. Extended Conductor-like Polarizable Continuum Solvation Model (CPCM-X) for Semiempirical Methods. *The Journal of Physical Chemistry A* **2023**, *127* (33), 7036–7043. DOI: 10.1021/acs.jpca.3c04382.

(26) Cosier, J.; Glazer, A. M. A nitrogen-gas-stream cryostat for general X-ray diffraction studies. *Journal of Applied Crystallography* **1986**, *19* (2), 105–107. DOI: 10.1107/S0021889886089835.

(27) Rigaku Oxford Diffraction. **2021**.

(28) Palatinus, L.; Chapuis, G. SUPERFLIP - a computer program for the solution of crystal structures by charge flipping in arbitrary dimensions. *Journal of Applied Crystallography* **2007**, *40* (4), 786–790. DOI: 10.1107/S0021889807029238.

(29) Betteridge, P. W.; Carruthers, J. R.; Cooper, R. I.; Prout, K.; Watkin, D. J. CRYSTALS version 12: software for guided crystal structure analysis. *Journal of Applied Crystallography* **2003**, *36* (6), 1487. DOI: 10.1107/S0021889803021800.

(30) Cooper, R. I.; Thompson, A. L.; Watkin, D. J. CRYSTALS enhancements: dealing with hydrogen atoms in refinement. *Journal of Applied Crystallography* **2010**, *43*, 1100–1107. DOI: 10.1107/S0021889810025598.
